# Rh-Catalyzed Cycloaddition Cascade of Allenynes and Maleimides: A Powerful Strategy for Constructing Complex Pentacyclic Structures with a Bicyclo[2.2.2]octene Core

**DOI:** 10.1021/acs.orglett.5c04524

**Published:** 2025-12-22

**Authors:** Elias A. Romero-Cavagnaro, Albert Artigas, Anna Pla-Quintana, Anna Roglans

**Affiliations:** † Institut de Química Computacional i Catàlisi (IQCC) and Departament de Química, 16738 Universitat de Girona (UdG), Facultat de Ciències, C/Maria Aurèlia Capmany, 69, 17003 Girona, Catalunya, Spain

## Abstract

Herein, we report that 1,5- and 1,6-allenynes react with two equivalents of maleimide to afford pentacyclic frameworks featuring a bicyclo[2.2.2]­octene core in a fully diastereoselective fashion. DFT calculations and deuterium-labeling studies reveal an unconventional mechanism initiated by a noncanonical [2 + 2 + 2] cycloaddition that, through intramolecular hydrogen shifts, diverges from the classical pathway to generate a conjugated diene. A subsequent thermal Diels–Alder reaction with a second maleimide completes the cascade, unveiling a distinct reactivity mode.

## Graphical abstract

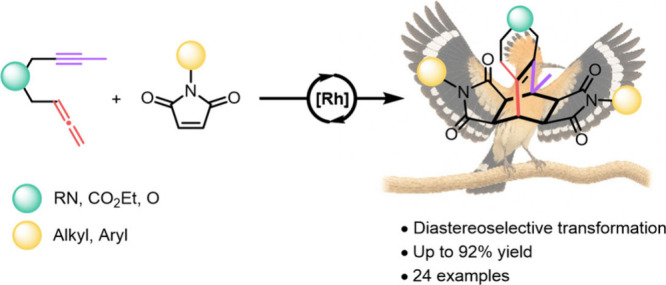

The bicyclo[2.2.2]­octene framework is a rigid, strain-free polycyclic hydrocarbon with a well-defined three-dimensional architecture that enables precise spatial arrangement of functional groups.^1^ This versatile scaffold is a key structural motif in a range of complex targets, including biomolecules and natural products (Figure 1A). Examples of the latter include eremolactone,^2^ a naturally occurring diterpene; kopsidasine,^3^ a member of the *Kopsia* alkaloid family; and kingianin A,^4^ a compound isolated from the bark of *Endiandra kingiana*, that exhibits an affinity for the antiapoptotic protein BcL-xL.^5^ Beyond natural products, the bicyclo[2.2.2]­octene skeleton is also featured in tecovirimat,^6^ an ST-246 class antiviral drug used to combat orthopoxvirus, and mitindomide, a highly insoluble compound with antitumor activity *in vivo*.^7^ Furthermore, the rigidity of this scaffold has been exploited in the design of the dicationic organic structure-directing agent **SDA2**, which is used for the synthesis of mesoporous chiral zeolites.^8^

**Figure 1:**
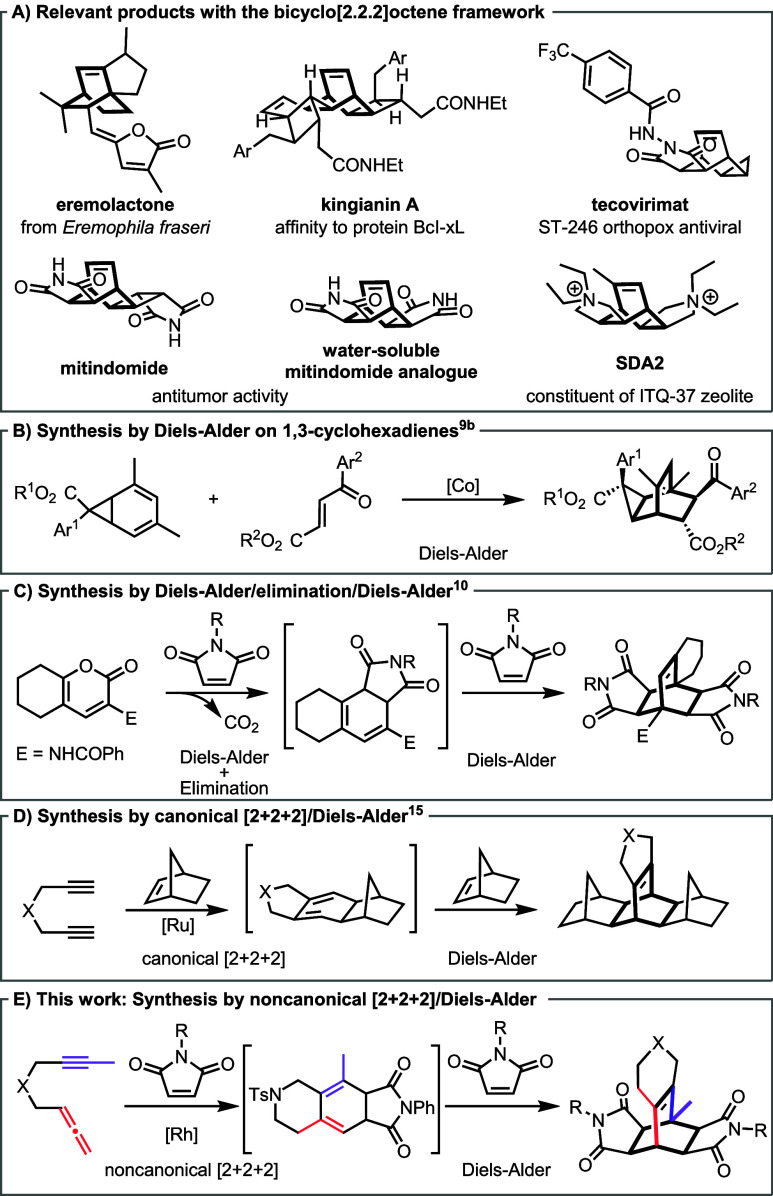
A) Relevant examples containing the bicyclo[2.2.2]­octene framework. B–D) Selected precedents. E) This work.

These diverse applications highlight the importance of developing efficient strategies for the synthesis of bicyclo[2.2.2]­octene scaffolds. The Diels–Alder reaction of 1,3-cyclohexadienes is a powerful tool for assembling nonsymmetrically substituted bicyclo[2.2.2]­octenes (Figure 1B).^9^ In contrast, access to symmetrically substituted analogues requires more elaborate sequences. One approach involves a double Diels–Alder reaction, where 1,3-dienes undergo an initial cycloaddition followed by 1,4-elimination (e.g., expelling CO_2_ for pyran-2-ones,^10^ (Figure 1C) or HBr,^11^ RCONH_2_ or RCOOH^12^ for other dienes), regenerating a 1,3-cyclohexadiene that engages in a second Diels–Alder reaction. An alternative strategy relies on transition metal-catalyzed [2 + 2 + 2] cycloaddition of a diyne and an alkene to generate a 1,3-cyclohexadiene, which then undergoes a subsequent Diels–Alder reaction with the same alkene. First described by Chalk^13^ under nickel catalysis and later exploited by Tsuda in copolymerizations,^14^ this methodology has also been demonstrated with Ru (Itoh) (Figure 1D),^15^ Co (Jeganmohan)^16^ and Rh (Tanaka)^17^ catalysts.

Building on the rich reactivity of allenes and their potential to access divergent products through chemoselectivity control,^18^ we report a cycloaddition/Diels–Alder cascade of an allenyne substrate. In this case, the allenyne cycloaddition, instead of yielding vinylallenes as previously reported,^19^ unexpectedly delivers 1,3-cyclohexadienes that readily engage in Diels–Alder reaction (Figure 1E). This strategy provides streamlined access to topologically different mitindomide analogues. Moreover, detailed mechanistic studies underscore the critical role of allenes in dictating selectivity in cycloaddition cascades.

We began our study by testing the reaction of *N*-tosyl-tethered allenyne **1a** and maleimide **2a** (Scheme 1). Using [Rh­(cod)­Cl]_2_/DPEphos in *o*-DCB at 150 °C, as in our previous study,^19b^ the reaction yielded two products in an 80:20 ratio. MS, NMR, and X-ray analysis showed that the major product **3a** features bicyclo[2.2.2]­octene core, whereas **4a** is a tricyclic diene. To favor **3a** and suppress **4a**, we optimized the reaction conditions (Table S1), screening ligands (Xantphos, BINAP, and DTBM-SEGphos), solvents (PhCl, toluene, DCE, acetonitrile, DCE:EtOH), temperatures, and catalyst loadings. Since **4a** appeared to isomerize to an endocyclic diene capable of a second Diels–Alder reaction, we introduced acidic additives to promote this step. Using catalytic TFA in DCE:EtOH (5:1) efficiently drove the formation of **3a**. The optimal conditions—1 equiv of **1a**, 5 equiv of **2a**, 5 mol % [Rh­(cod)­Cl]_2_, 15 mol % DPEphos and 5 mol % TFA in DCE:EtOH (5:1) at 80 °C for 16 h—afforded **3a** in 92% yield with complete diastereoselectivity (all four protons at the two ring junctions oriented opposite to the bridging ring (Scheme 1).

**Scheme 1:**
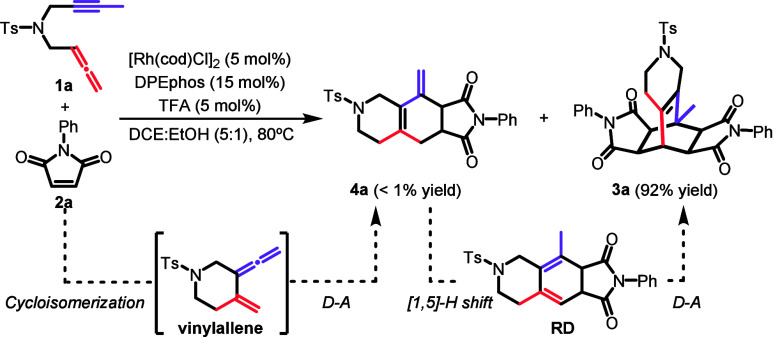
Optimized Reaction Conditions and Initially Postulated Mechanism

With the optimized reaction conditions in hand, we then evaluated the scope of the process (Scheme 2). A variety of alkyl- and aryl-substituted maleimides were tested. The treatment of compound **1a** with alkyl-substituted maleimides afforded the corresponding products with high yields of 92% (**3c**), 88% (**3d**), 79% (**3e**) and 90% (**3f**), respectively. Additionally, nonsubstituted maleimide and benzyl maleimide gave excellent yields of the corresponding pentacyclic adducts **3b** and **3g**. Aryl-substituted maleimides also participated efficiently in the reaction. Both electron-donating and electron-withdrawing groups in the phenyl ring [H (**3a**), *p*-OMe (**3h**), *p*-NO_2_ (**3i**), *p*-Br (**3j**), *p*-^t^Bu (**3k**), 3,5-CF_3_ (**3l**)] were well tolerated affording cycloadducts with yields ranging from 81% to 92%. Notably, the benchmark reaction between **1a** and phenylsubstituted maleimide **2a** to give **3a** could be scaled up to 1.0 mmol, affording the product in an excellent 87% yield. From these results, we can conclude that the electronics and sterics of the maleimide derivatives do not exert a significant influence. To further probe maleimide reactivity, mixed experiments were performed with allenyne **1a** and pairs of maleimides (N-H/N-Ph and N-Ph/N-Cy). In the N-H/N-Ph experiment, heteroadduct **3ab** was obtained in 37% yield, along with **3a** (18%) and **3b** (29%). In the N-Ph/N-Cy experiment, heteroadduct **3ae** was formed in 32% yield, together with **3a** (52%) and **3e** (10%). Comparison of the corresponding homocycloadduct yields indicates the following reactivity order: N-H > N-Ph > N-Cy. Although a wide range of maleimides are efficient in this process, unfortunately other electron-deficient alkenes failed to undergo the reaction.

**Scheme 2:**
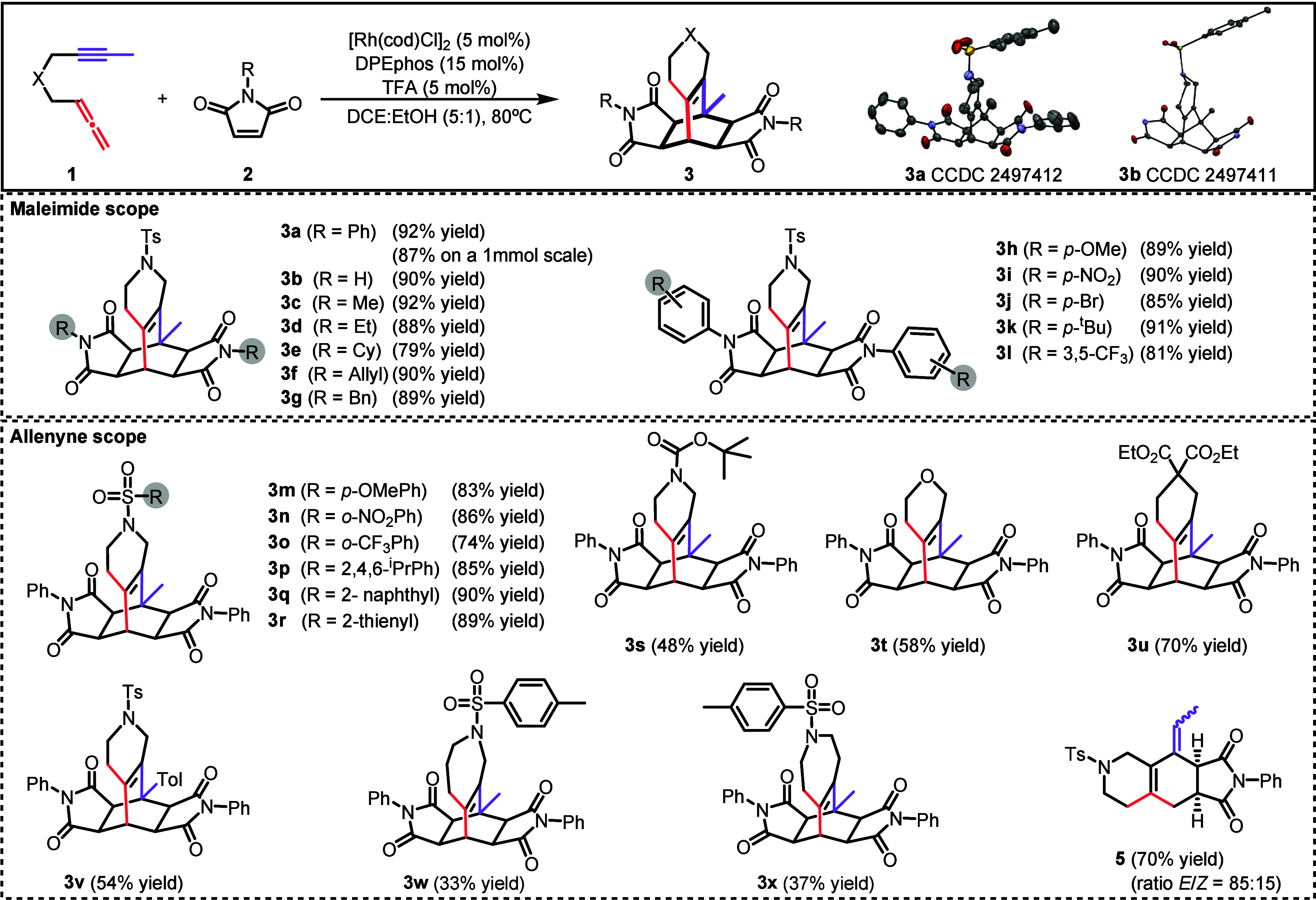
Scope of the Process

We then evaluated the scope of the allenyne. Using sulfonamide-tethered 1,5-allenynes, the corresponding pentacyclic scaffolds **3m**–**3r** were obtained in yields of up to 90%. The reaction tolerated a variety of substituents on the phenylsulfonamide moiety, including, electron-donating groups (**3m**), electron-withdrawing groups (**3n**, **3o**), alkyl groups (**3p**), as well as extended aromatic systems such as naphthalene (**3q**) and heteroaromatic thiophene (**3r**), affording the respective cycloadducts **3m**-**3r** in excellent yields. Beyond sulfonamide tethers, the methodology was also applicable to 1,5-allenynes bearing alternative tethering groups, including *tert*-butylcarbamate, an oxygen atom and diethyl malonate. These substrates reacted efficiently with Ph-maleimide, affording the corresponding products **3s**, **3t** and **3u** in good yields. To assess the impact of tether length, two 1,6-allenynes were evaluated, giving products **3w** and **3x** in 33% and 37% yield, respectively, along with unidentified byproducts. These results suggest that extending the tether changes the reaction profile, due to increased conformational flexibility and the greater geometric challenge of forming the larger ring. In contrast, when the alkyne of the allenyne had an ethyl group in the terminal position, the reaction stopped at derivative **5** and this was obtained in a 70% yield as a mixture of E/Z isomers in a ratio of 85:15. Remarkably, across all substrates studied, no detectable formation of tricyclic diene **4** was observed.

Surprisingly, the allenyne bearing a tolyl group at the alkyne terminus underwent the transformation to yield product **3v** with a 54% yield. This outcome was unexpected, as this substrate cannot form the vinylallene intermediate shown in Scheme 1. We therefore performed a series of mechanistic experiments (Scheme S4 in the SI). First, compound **4b** (N-R, R = H) was subjected to the optimized reaction conditions in the presence of Ph-maleimide. However, the starting material was recovered unchanged, and neither the isomerized product nor the bisadduct was detected. Repeating the reaction in the absence of the Rh complex yielded the same result, suggesting again a pathway markedly different from the one initially proposed (Scheme 1). Next, we run the reaction with allenyne **1m** containing two methyl groups in the terminal position of the allene and again the starting material was fully recovered. We also carried out deuterium-labeling experiments. In the first one, the two terminal protons of the allene moiety were replaced with deuterium. After the reaction, only 45% of the deuterium was retained at the original carbon, indicating significant deuterium loss. In the second experiment, the substrate was labeled at the methyl adjacent to the alkyne. Here, only 19% of deuterium remained in the product. Finally, when nondeuterated starting material was used but EtOD and TFA-d were added as external deuterium sources, extensive scrambling was observed, with 68%, 100%, and 80% deuterium incorporation at positions *A*, *B* and *C*, respectively (see compound **3c** in Scheme 3 for positions). The deuterium incorporation in all these final compounds was also checked with mass spectrometry (MS) (see Graphs 1–4 in the SI). Together, these results indicate the involvement of protic sources in multiple H-shift processes throughout the reaction.

**Scheme 3:**
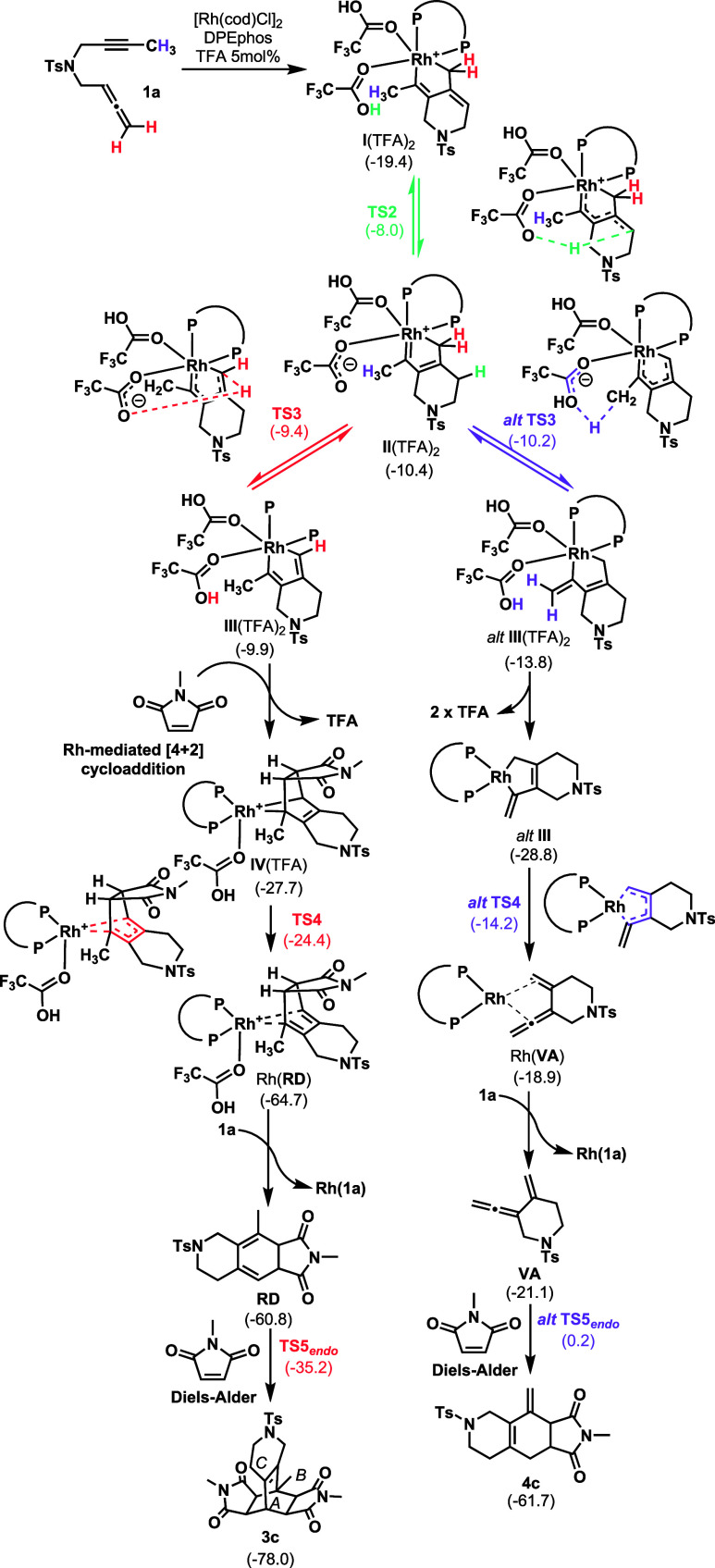
Mechanistic Proposal (Values Are Gibbs Energies with Respect to Rh­(**1a**) and Are Given in kcal·mol^–1^)

To get full insight into the chemoselectivity of the process, we simulated the Rh­(DPEphos)-catalyzed reaction between substrate **1a** and **2c** at the M06L-D3/cc-pVTZ-PP/SMD­(79% Dichloroethane, 21% Ethanol)//B3LYP-D3/cc-pVDZ-PP level of theory at 353 K (in Scheme 3 and Figures S12–S14 in the SI).

As reported previously by us,^19b^ the reaction starts with coordination of the Rh­(DPEphos) complex to allenyne **1a** forming Rh­(**1a**), which is taken as the reference point (Δ*G* = 0.0 kcal·mol^–1^) in Figure S12. Oxidative coupling through **TS1** (Δ*G* ^‡^ = 14.7 kcal·mol^–1^) leads to rhodacyclopentene intermediate **I**, releasing 19.4 kcal·mol^–1^. This intermediate can coordinate to two TFA molecules present in the reaction medium, thus delivering intermediate **I**(TFA)_2_, an octahedral 18-electron intermediate. Subsequently, one of the coordinated TFA ligands transfers its proton, yielding Rh­(III) intermediate **II**(TFA)_2_, which lies 9.0 kcal·mol^–1^ higher in the Gibbs energy surface (*ΔG* = −10.4 kcal·mol^–1^). Overall, coordination and proton transfer via **TS2** require an activation free energy of 11.4 kcal·mol^–1^. This same process was estimated to have a much higher Gibbs energy barrier of 28.4 kcal·mol^–1^ when H_2_O was considered as the proton source,^19b^ consistent with the greater acidity of TFA. From intermediate **II**(TFA)_2_, the resulting trifluoroacetate anion can be reprotonated to regenerate TFA through proton abstraction at two different positions, ultimately leading to the formation of products **3c** or **4c**. Abstraction from the methylene group vicinal to the Rh center (**TS3**) demands only 1.0 kcal·mol^–1^ and produces rhodacyclopentadiene intermediate **III**(TFA)_2_ in a slightly endergonic process. Importantly, the reversibility of the proton shifts accounts for the observed deuterium scrambling in the labeling experiments, as an equilibrium is established that allows hydrogen migration in both directions. In addition, because allenyne **1m** lacks a proton at the terminal position of the allene, this substrate failed to form the corresponding product **3m**. Proton abstraction is rapidly followed by exergonic loss of one TFA ligand and a barrierless Rh-mediated [4 + 2] cycloaddition with maleimide (see Figure S14 in the SI). The latter step takes place selectively with an *endo* approach, favored by steric congestion from the phosphine ligand. Overall, these three steps from **II**(TFA)_2_ to cycloadduct **IV**(TFA) release 17.3 kcal·mol^–1^. Finally, reductive elimination (**TS4**) coupled with TFA loss through a low Gibbs energy barrier of 3.5 kcal·mol^–1^ results in intermediate Rh­(**RD**) (*ΔG* = −64.7 kcal·mol^–1^). Displacement with a new molecule of **1a** and release of the cyclohexadiene intermediate **RD** closes the catalytic cycle. It is important to note that in the present case, the hydrogen-shift processes intricately integrated into the catalytic cycle itself, define a distinct variant of [2 + 2 + 2] cycloaddition chemistry delivering a cyclohexadiene instead of the expected cyclohexene product. An additional uncatalyzed Diels–Alder cycloaddition with methyl maleimide (**TS5**, Δ*G* ^‡^ = 21.3 kcal·mol^–1^), which again takes place selectively through *endo* approximation ultimately leads to product **3c**.

Alternatively, from **II**(TFA)_2_, a barrierless proton abstraction at the methyl group (*alt* **TS3**) can form intermediate *alt* **III**(TFA)_2_, which readily releases its two TFA ligands, delivering *alt* **III**. This intermediate is located slightly lower than **IV**(TFA) on the Gibbs energy surface (−28.8 kcal·mol^–1^). However, reductive elimination from *alt* **III** (*alt* **TS4**) has a much higher activation energy of 14.6 kcal·mol^–1^ and, moreover, it is endergonic by 9.9 kcal·mol^–1^. Such a difference accounts for the chemoselective formation of product **3c** under the reaction conditions employed. Finally, substitution of the vinylallene intermediate **VA** by a new molecule of **1a** closes the catalytic cycle. **VA** participates in a Diels–Alder cycloaddition with maleimide analogously to **RD** to finally form product **4c** in a process that demands 25.6 kcal·mol^**‑1**^ and that is exergonic by −9.2 kcal·mol^–1^.

To clarify why the ethyl-substituted allenyne diverts to product **5** instead of forming **3**, we identified the Rh-mediated [4 + 2] cycloaddition between **III**(TFA) and the maleimide as the key differentiating step. A frozen optimization at the BP86-D3/cc-pVDZ-PP level, fixing the maleimide–rhoda­cyclo­penta­diene distance at 2.02 Å, indicates that the ethyl group’s steric bulk disfavors their approach. Consequently, intermediate **II**(TFA)_2_ follows an alternative pathway, leading to **5**.

In summary, we have established a diastereoselective cascade strategy that converts 1,5- and 1,6-allenynes and maleimides into bicyclo[2.2.2]­octene frameworks related to the mitindomide class. The transformation proceeds through a tandem cycloaddition/Diels–Alder sequence in which a noncanonical [2 + 2 + 2] cycloaddition with one maleimide first builds a conjugated diene, which then undergoes a thermal Diels–Alder reaction with a second maleimide. Notably, as supported by DFT analysis and deuterium-labeling experiments, this [2 + 2 + 2] process deviates from the conventional pathway: rather than forming the expected cyclohexene, it proceeds through a sequence of intramolecular hydrogen-shift events that redirect the reaction course toward the observed cyclohexadiene intermediate. Such behavior contrasts with prior reports where similar rearrangements were proposed to occur off the metal-catalyzed manifold in a subsequent step. Here, however, the H-shift processes appear to be intricately integrated into the catalytic cycle itself, defining a new mechanistic variant of [2 + 2 + 2] cycloaddition chemistry. Beyond expanding the mechanistic understanding of these transformations, these findings open the door to the design of modified [2 + 2 + 2] manifolds capable of accessing previously unattainable molecular scaffolds through controlled cascade sequences.

## Supplementary Material



## Data Availability

The data underlying this study are available in the published article and its Supporting Information. xyz Cartesian coordinates, energies, and vibrational frequencies of all optimized complexes are openly available in the ioChem-BD repository at https://iochem.udg.edu/browse/handle/100/7462.^20^

## Notes

Experimental details, materials, methods, characterization data, NMR spectra for all unknown compounds, DFT calculations, and information on X-ray diffraction experiments (PDF)
