# Supplementary material for: Rh-Catalyzed Cycloaddition Cascade of Allenynes and Maleimides: A Powerful Strategy for Constructing Complex Pentacyclic Structures with a Bicyclo[2.2.2]octene Core
Source: Org Lett. 2025 Dec 22;28(1):141–6. doi: 10.1021/acs.orglett.5c04524 (PMC12797331; doi:10.1021/acs.orglett.5c04524)
Supplement: Supplementary file 1 [file ol5c04524_si_001.pdf]

SUPPLEMENTARY INFORMATION

Rh-Catalyzed Cycloaddition Cascade of Allenynes and Maleimides: A Powerful Strategy for Constructing Complex Pentacyclic Structures with a Bicyclo[2.2.2]Octene Core

Elias A. Romero-Cavagnaro,[a] Albert Artigas,[a] Anna Pla-Quintana,[a*] Anna Roglans[a*]

[a]Institut de Química Computacional i Catàlisi (IQCC) and Departament de Química, Universitat de Girona (UdG), Facultat de Ciències, C/ Maria Aurèlia Capmany, 69, 17003-Girona, Catalunya, Spain.

E-mail: anna.plaq@udg.edu; anna.roglans@udg.edu

Table of contents

General materials and methods 4

S1. General scheme for the synthesis of allenynes 1a-1o 5

S2. General procedure for the synthesis of *N*-alkyl/aryl maleimide derivatives 2a, 2e-2l 6

Table S1. Optimization of Rh(I)-catalyzed cycloaddition of allenyne 1a and maleimide 2a. 9

S3. General procedure for the Rh(I)-catalyzed cycloaddition of allenynes and maleimides. 10

S3.1 Maleimides' Scope 11

S3.2 Allenynes' scope 17

S3.3 Mixed Experiments 24

S3.4 Unsuccessful 1,6-allenynes and alkenes tested 26

S4. Mechanistic Studies 27

S4.1 Mechanistic experiments 27

S4.2 Synthesis of Monoadduct 4b 27

S4.3 Reaction between 4b and 2a 30

S4.4 Deuterium labelling experiments 31

^{1}H and ^{13}C NMR spectra 36

Compound 2a 36

Compound 2e 37

Compound 2f 38

Compound 2g 39

Compound 2h 40

Compound 2i 41

Compound 2j 42

Compound 2k 43

Compound 2l 44

Compound 3a 45

Compound 3b 50

Compound 3c 55

Compound 3d 59

Compound 3e 65

Compound 3f 70

Compound 3g 75

Compound 3h 79

Compound 3i 83

Compound 3j 87
Compound 3k 91
Compound 3l 96
Compound 3m 102
Compound 3n 107
Compound 3o 112
Compound 3p 117
Compound 3q 122
Compound 3r 127
Compound 3s 132
Compound 3t 137
Compound 3u 142
Compound 3v 147
Compound 3w 152
Compound 3x 157
Compound 5 162
Compound 3ab 167
Compound 3ae 172
S5. Crystal structure of compound 3a 177
Table S2. Sample and crystal data for NR_Ph_DDA. 178
Table S3. Data collection and structure refinement for NR_Ph_DDA. 178
Table S4. Atomic coordinates and equivalent isotropic atomic displacement parameters (Å2) for NR_Ph_DDA. 179
S6. Crystal structure of compound 3b 183
Table S5. Sample and crystal data for ER058_F5. 184
Table S6. Data collection and structure refinement for ER058_F5. 185
Table S7. Atomic coordinates and equivalent isotropic atomic displacement parameters (Å^2) for ER058_F5. 186
S7. Computational study 187
S7.1. Computational details 187
S7.2. Reaction mechanism 189
References: 194

General materials and methods

Unless otherwise noted, materials were obtained from commercial suppliers and used without further purification. CH_2Cl_2 and THF were dried under nitrogen by passing through solvent purification columns (MBraun, SPS-800). Reaction progress during the preparation of all compounds was monitored using thin layer chromatography on Macherey-Nagel Xtra SIL G/UV254 silica gel plates. Solvents were removed under reduced pressure with a rotary evaporator. Reaction mixtures were chromatographed on silica gel. All ^{1}H and ^{13}C NMR spectra were recorded on a Bruker ASCEND 400 spectrometer equipped with a 5 mm BBFO probe and a Brucker Ultrashield AVANCE III400 using $CDCl_3$ and DMSO as a deuterated solvent. Chemical shifts for ^{1}H and ^{13}C NMR are reported in ppm (δ) relative to residual solvent signals. Coupling constants are given in Hertz (Hz). ^{1}H and ^{13}C NMR signals were assigned based on 2D-NMR HSQC, HMBC, COSY, and NOESY experiments when necessary. Mass spectrometry analyses were recorded on a Bruker microTOF-Q II mass spectrometer (high resolution), equipped with electrospray ion source. The instrument was operated in the positive ESI (+) ion mode. IR spectra were recorded on an Agilent Cary 630 FT-IR spectrometer equipped with an ATR sampling accessory. The X-ray intensity data were measured on a three-circle diffractometer system equipped with a Ceramic X-ray tube (Mo Kα, λ = 0.71073 Å) and a doubly curved silicon crystal Bruker Triumph monochromator. Melting points were measured in a SMP10 apparatus from Stuart and were reported without any correction.

S1. General scheme for the synthesis of allenynes 1a-1o

Part A. Synthesis of diynes

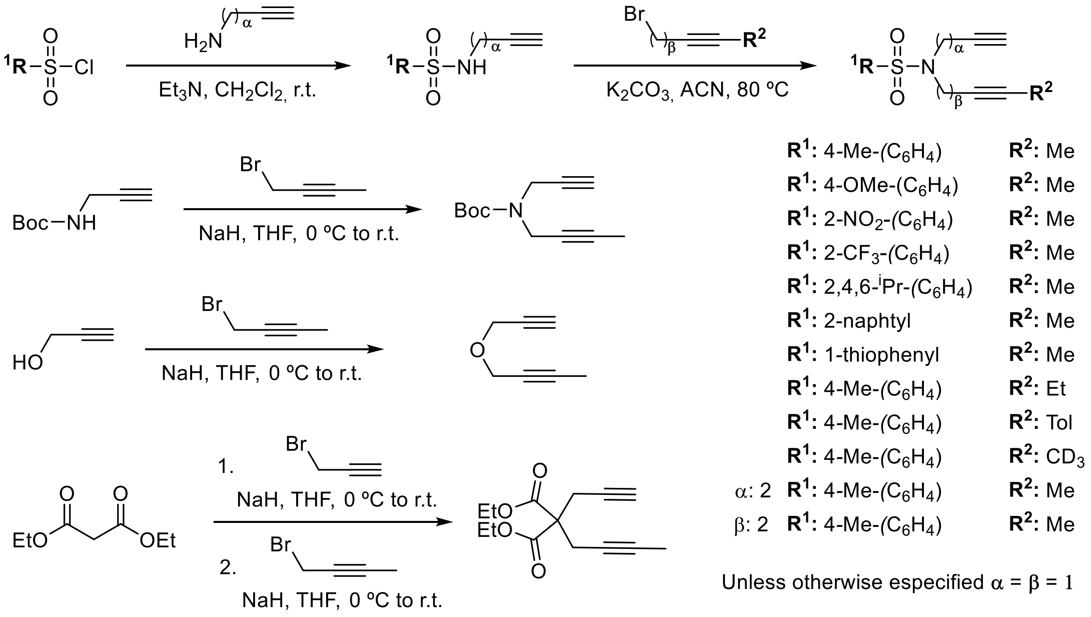

Part B. Allene formation (Crabbé reaction)

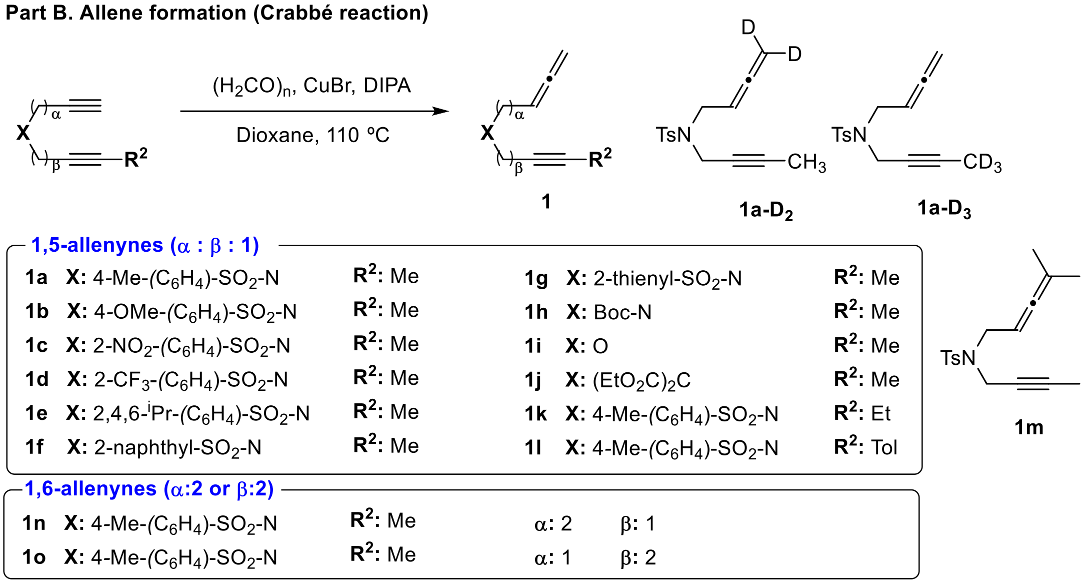

1,5-Allenynes **1a-1l** were prepared from the corresponding bisalkynes using Crabbé homologation reaction. Experimental procedures and full characterization have been described previously by us.[1a] Allenyne **1m** was prepared following the procedure reported by Zang et al.[1b] Allenyne **1n** and **1o** were prepared following the procedure reported by Chung et al.[1c]

S2. General procedure for the synthesis of *N*-alkyl/aryl maleimide derivatives 2a, 2e-2l

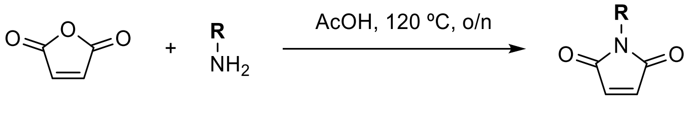

2a, 2e-2l

N-alkyl/aryl maleimide derivatives **2a, 2e-2l** were prepared according to the general method reported in the literature.[2] N-H (**2b**), N-Me (**2c**) and N-Et (**2d**) are commercially available.

In a 25 mL round-bottom flask containing a mixture of maleic anhydride (2.0 equiv.) in acetic acid, amine (1.0 equiv.) was added. The resulting mixture was stirred for 6 – 8 h at 120 °C (TLC monitoring) and was then cooled to room temperature. The crude was transferred to a 500 mL beaker and saturated aqueous $NaHCO_3$ solution was added until the solution is neutralized. The aqueous mixture was extracted with ethyl acetate (3 x 30 mL). The organic layer was further washed with 1M HCl (2 x 50 mL), with brine solution (30 mL), dried over anhydrous $MgSO_4$, filtered off and the solvent was removed under reduced pressure. The resulting crude was purified by column chromatography on silica gel (Hexanes/EtOAc 9:1) to afford the corresponding maleimide derivatives. [2]

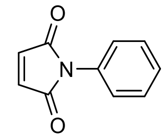

2a

Compound 2a was obtained from aniline (0.91 mL, 9.98 mmol, 1 equiv.) and maleic anhydride (1.97 g, 20.10 mmol, 2.0 equiv.) in acetic acid (15 mL) following the general procedure. Purification by column chromatography on silica gel provided **compound 2a** (1.30 g, 75% yield) as a yellow solid.[2a]

MW ($C_{10}H_7NO_2$)**:** 173.17 g/mol; **^{1}H-NMR (400 MHz, $CDCl_3$) δ (ppm)**: 7.52 – 7.43 (m, 2H), 7.40 – 7.31 (m, 3H), 6.85 (s, 2H); **HRMS (ESI) m/z:** $[M + Na]^+$ Calculated for $C_{10}H_7NO_2Na$ 196.0369, Found: 196.0371.

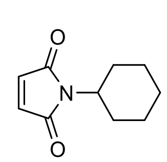

2e

Compound 2e was obtained from cyclohexylamine (0,58 mL, 5.06 mmol, 1 equiv.) and maleic anhydride (1.01 g, 10.30 mmol, 2.0 equiv.) in acetic acid (8 mL) following the general procedure. Purification by column chromatography on silica gel provided **compound 2e** (0.64 g, 71% yield) as a colourless solid.[2b]

MW ($C_{10}H_{13}NO_2$)**:** 179.22 g/mol; **^{1}H-NMR (400 MHz, $CDCl_3$) δ (ppm)**: 6.62 (s, 2H), 3.91 (tt, *J* = 12.4, 3.9 Hz, 1H), 2.05 (dq, *J* = 12.4, 3.9 Hz, 2H), 1.87 – 1.79 (m, 2H), 1.70 – 1.62 (m, 3H), 1.41 – 1.09 (m, 3H); **HRMS (ESI) m/z:** $[M + Na]^+$ Calculated for $C_{10}H_{13}NO_2Na$ 202.0838, Found: 202.0838.

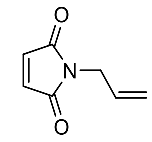

2f

Compound 2f was obtained from allylamine (0.40 mL, 5.34 mmol, 1 equiv.) and maleic anhydride (0.99 g, 10.01 mmol, 2.0 equiv.) in acetic acid (8 mL) following the general procedure. Purification by column chromatography on silica gel provided **compound 2f** (0.44 g, 60% yield) as a colourless solid.[2c]

MW ($C_7H_7NO_2$)**:** 137.14 g/mol; **^{1}H-NMR (400 MHz, CDCl$_3$) δ (ppm)**: 6.72 (s, 2H), 5.86 – 5.73 (m, 1H), 5.21 – 5.17 (m, 1H), 5.17 – 5.14 (m, 1H), 4.13 (dt, *J* = 5.4, 1.5 Hz, 2H); **HRMS (ESI) m/z:** $[2M + Na]^+$ Calculated for $C_{14}H_{14}N_2O_4Na$ 297.0846, Found: 297.0846.

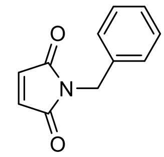

2g

Compound 2g was obtained from benzylamine (0.55 mL, 5.03 mmol, 1 equiv.) and maleic anhydride (0.98 g, 10.00 mmol, 2.0 equiv.) in acetic acid (8 mL) following the general procedure. Purification by column chromatography on silica gel provided **compound 2g** (0.60 g, 64% yield) as a colourless solid.[2b]

MW ($C_{11}H_9NO_2$)**:** 187.20 g/mol; **^{1}H-NMR (400 MHz, CDCl$_3$) δ (ppm)**: 7.37 – 7.27 (m, 5H), 6.71 (s, 2H), 4.68 (s, 2H); **HRMS (ESI) m/z:** $[M + Na]^+$ Calculated for $C_{11}H_9NO_2Na$ 210.0525, Found: 210.0520.

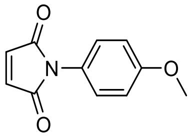

2h

Compound 2h was obtained from *p*-methoxyaniline (0.62 g, 5.04 mmol, 1 equiv.) and maleic anhydride (1.02 g, 10.40 mmol, 2.0 equiv.) in acetic acid (8 mL) following the general procedure. Purification by column chromatography on silica gel provided **compound 2h** (0,82 g, 80% yield) as a greenish solid.[2a]

MW ($C_{11}H_9NO_3$)**:** 203.20 g/mol; **^{1}H-NMR (400 MHz, CDCl$_3$) δ (ppm)**: 7.27 – 7.19 (m, 2H), 7.02 – 6.94 (m, 2H), 6.83 (s, 2H), 3.83 (s, 3H); **HRMS (ESI) m/z:** $[M + Na]^+$ Calculated for $C_{11}H_9NO_3Na$ 226.0475, Found: 226.0474.

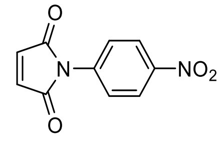

2i

Compound 2i was obtained from *p*-nitroaniline (0.48 mL, 5.11 mmol, 1 equiv.) and maleic anhydride (0.99 g, 10.01 mmol, 2.0 equiv.) in acetic acid (8 mL) following the general procedure. Purification by column chromatography on silica gel provided **compound 2i** (0.71 g, 64% yield) as a yellowish solid.[2b]

MW ($C_{10}H_6N_2O_4$)**:** 218.16 g/mol; **^{1}H-NMR (400 MHz, Acetone-d$_6$) δ (ppm)**: 8.42 – 8.34 (m, 2H), 7.82 – 7.74 (m, 2H), 7.13 (s, 2H); **HRMS (ESI) m/z:** $[M + MeOH + Na]^+$ Calculated for $C_{11}H_{10}N_2O_5Na$ 273.0482, Found: 273.0487.

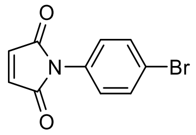

2j

Compound 2j was obtained from *p*-bromoaniline (0.87 g, 5.06 mmol, 1 equiv.) and maleic anhydride (1.00 g, 10.20 mmol, 2.0 equiv.) in acetic acid (8 mL) following the general procedure. Purification by column chromatography on silica gel provided **compound 2j** (1.06 g, 83% yield) as a greenish solid.[2b]

MW ($C_{10}H_6BrNO_2$)**:** 252.07 g/mol; **^{1}H-NMR (400 MHz, $CDCl_3$) δ (ppm)**: 7.63 – 7.55 (m, 2H), 7.30 – 7.22 (m, 2H), 6.86 (s, 2H); **HRMS (ESI) m/z:** $[M + MeOH + Na]^+$ Calculated for $C_{11}H_{10}BrNO_3Na$ 306.9736, Found: 305.9734.

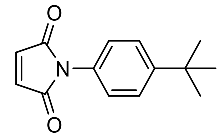

2k

Compound 2k was obtained from *p*-^{t}butylaniline (0.80 mL, 5.07 mmol, 1 equiv.) and maleic anhydride (0.97 g, 9.89 mmol, 2.0 equiv.) in acetic acid (8 mL) following the general procedure. Purification by column chromatography on silica gel provided **compound 2k** (0.99 g, 86% yield) as a yellow solid.[2c]

MW ($C_{14}H_{15}NO_2$)**:** 229.28 g/mol; **^{1}H-NMR (400 MHz, Acetone-d_6) δ (ppm)**: 7.56 – 7.48 (m, 2H), 7.33 – 7.25 (m, 2H), 7.01 (s, 2H), 1.34 (s, 9H); **HRMS (ESI) m/z:** $[M + Na]^+$ Calculated for $C_{14}H_{15}NO_2Na$ 252.0995, Found: 252.0994.

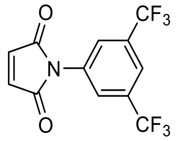

2l

Compound 2l was obtained from 3,5-bis(trifluoromethyl) aniline (0.78 mL, 4.99 mmol, 1 equiv.) and maleic anhydride (1.02 g, 10.40 mmol, 2.0 equiv.) in acetic acid (8 mL) following the general procedure. Purification by column chromatography on silica gel provided **compound 2l** (1.17 g, 76% yield) as a colourless solid.[2b]

MW ($C_{12}H_5F_6NO_2$)**:** 309.17 g/mol; **^{1}H-NMR (400 MHz, Acetone-d_6) δ (ppm)**: 8.17 (br s, 2H), 8.08 (br s, 1H), 7.17 (s, 2H); **HRMS (ESI) m/z:** Calculated for $[M + MeOH + H]^+$ Calculated for $C_{13}H_{10}F_6NO_3$ 342.0559, Found: 342.0556.

Table S1. Optimization of Rh(I)-catalyzed cycloaddition of allenyne 1a and maleimide 2a.

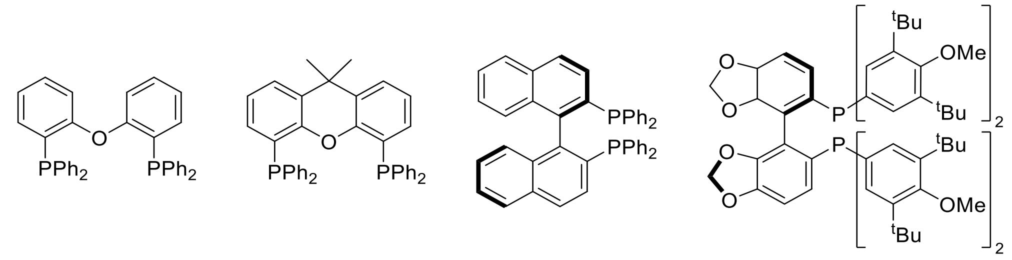

DPEphos **L1** | Xantphos **L2** | (*R*)-BINAP **L3** | (*R*)-DTBM-SEGphos **L4**

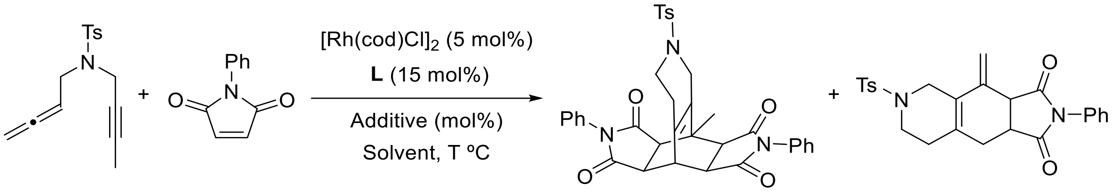

1a **2a** **3a** **4a**

Entry	Ligand	Solvent	Additive	T (ºC)	Ratio (3a:4a)[b]	Yield (3a)[c]
1	**L1**	*o*-DCB	-	150	80:20	70%
2	**L1**	PhCl	-	130	75:25	65%
3	**L1**	Tol	-	100	76:24	62%
4	**L1**	DCE	-	80	85:15	57%
5	**L1**	ACN	-	80	-	n.r.
6	**L1**	DCE	20 mol% TFA	80	99:>1	46%
7	**L1**	DCE:EtOH (5:1)	-	80	93:7	64%
8	**L1**	DCE:EtOH (5:1)	50 mol% TFA	80	99:>1	40%
9	**L1**	DCE:EtOH (5:1)	20 mol% TFA	80	99:>1	56%
10	**L1**	DCE:EtOH (5:1)	5 mol% TFA	80	99:>1	92%
11	**L2**	DCE:EtOH (5:1)	5 mol% TFA	80	1:2	12%
12[d]	**L3**	DCE:EtOH (5:1)	5 mol% TFA	80	>1:99	--
13[e]	**L4**	DCE:EtOH (5:1)	5 mol% TFA	80	-	--
14[f]	**L1**	DCE:EtOH (5:1)	5 mol% TFA	80	-	n.r
15	**L1**	DCE:EtOH (5:1)	5 mol% BA	80	-	n.r.
16	**L1**	DCE:EtOH (5:1)	5 mol% TFA	40	-	<50% conv
17[g]	**L1**	DCE:EtOH (5:1)	2 mol% TFA	80	-	56%

[a] Unless otherwise noted, reactions were carried out with 0.09 mmol of **1a**, 5 equiv. of **2a**, 5 mol% of $[Rh(cod)Cl]_2$ complex, 15 mol% of phosphine ligand in 5 mL of solvent at the indicated temperature overnight. [b] The yield was determined by NMR using Mesitylene as internal standard. [c] Isolated yield of **3a**. [d] Only **4a** was obtained in a 60% yield. [e] Only **4a** was obtained in a 26% yield. [f] No catalyst was used. [g] 2 mol% $[Rh(cod)Cl]_2$ and 6 mol% **L1**.

***o*-DCB**: *o*-dichlorobenzene; **Tol**: toluene; **DCE**: dichloroethane; **ACN**: acetonitrile; **TFA**: trifluoroacetic acid; **BA:** Benzoic acid.

S3. General procedure for the Rh(I)-catalyzed cycloaddition of allenynes and maleimides.

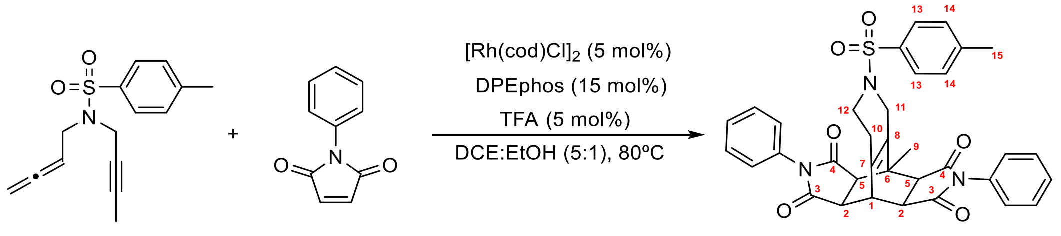

In a 10 mL capped vial, a mixture of $[Rh(cod)Cl]_2$ (2.2 mg, 0,0045 mmol, 0.05 equiv.) and DPEphos (7.3 mg, 0.0135 mmol, 0.15 equiv.) was purged with nitrogen, dissolved in an anhydrous mixture of DCE/EtOH/TFA (5:1:1.7x10^{-6}) (2 mL) and transferred via syringe into a solution of **allenyne 1a** (25.0 mg, 0.09 mmol, 1.0 equiv.) and **maleimide 2a** (78.0 mg, 0.45 mmol, 5 equiv.) in an anhydrous mixture of DCE/EtOH/TFA (5:1:1.7x10^{-6}) (3 mL) preheated at 80 ºC and under inert atmosphere. The resulting mixture was stirred for 16 h at the same temperature (TLC monitoring). The solvent was then removed under reduced pressure, and the resulting crude was purified by column chromatography on silica gel using mixtures of DCM:MeOH (100:0 to 99:1 v/v) to afford **compound 3a** (51.8 mg, 92% yield) as a colourless solid.

Large scale reaction:

In a 100 mL capped vial, a mixture of $[Rh(cod)Cl]_2$ (25.1 mg, 0,051 mmol, 0.05 equiv.) and DPEphos (79.8 mg, 0.148 mmol, 0.15 equiv.) was purged with nitrogen, dissolved in an anhydrous mixture of DCE/EtOH/TFA (5:1:1.7x10^{-6}) (10 mL) and transferred via syringe into a solution of **allenyne 1a** (276.1 mg, 1.00 mmol, 1.0 equiv.) and **maleimide 2a** (867.5 mg, 5.01 mmol, 5 equiv.) in an anhydrous mixture of DCE/EtOH/TFA (5:1:1.7x10^{-6}) (45 mL) preheated at 80 ºC and under inert atmosphere. The resulting mixture was stirred for 16 h at the same temperature (TLC monitoring). The solvent was then removed under reduced pressure, and the resulting crude was purified by column chromatography on silica gel using mixtures of DCM:MeOH (100:0 to 99:1 v/v) to afford **compound 3a** (542.6 mg, 87% yield) as a colourless solid.

MW ($C_{35}H_{31}N_3O_6S$)**:** 621.71 g/mol; **Rf:** 0.18 (DCM/MeOH 99:1); **MP (ºC)**: >190 (dec); **IR (ATR) ν (cm^{-1})**: 1703, 1372, 1152; **^{1}H-NMR (400 MHz, CDCl$_3$) δ (ppm)**: 7.61 (d, *J* = 8.2 Hz, 2H, H**13**), 7.45 – 7.36 (m, 6H, CH-**Ar**), 7.31 (d, *J* = 8.1 Hz, 2H, H**14**), 7.04 – 6.98 (m, 4H, CH-**Ar**), 3.73 (t, *J* = 3.1 Hz, 1H, H**1**), 3.5 (t, *J* = 2.7 Hz, 2H, H**11**), 3.21 (dd, *J* = 8.1, 3.1 Hz, 2H, H**2**), 2.98 (t, *J* = 5.6 Hz, 2H, H**12**), 2.87 (d, *J* = 8.2 Hz, 2H, H**5**), 2.45 (s, 3H, H**15**), 2.30-2.22 (m, , 2H, H**10**), 1.93 (s, 3H, H**9**); **^{13}C-NMR (101 MHz, CDCl$_3$) δ (ppm):** 175.3 (C**3**/C**4**), 174.6 (C**3**/C**4**), 144.0, 132.9, 132.7, 131.6, 131.4, 130.0 (C**14**), 129.5, 129.1, 127.8 (C**13**), 126.5, 49.1 (C**5**), 44.6

(C**11**), 43.8 (C**2**), 43.0 (C**12**), 42.7 (C**6**), 38.5 (C**1**), 29.4 (C**10**), 21.7 (C**15**), 16.3 (C**9**); **HRMS (ESI) m/z:** $[M + NH_4]^+$ Calculated for $C_{35}H_{35}N_4O_6S$ 639.2272, Found: 639.2251.

S3.1 Maleimides' Scope

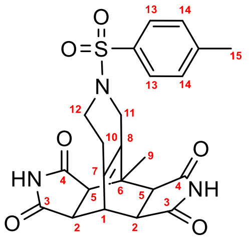

3b

Compound 3b was obtained from **allenyne 1a** (25.0 mg, 0.09 mmol, 1 equiv.) and **maleimide 2b** (43.7 mg, 0.45 mmol, 5 equiv.) using a mixture of $[Rh(cod)Cl]_2$ (2.0 mg, 0,0041 mmol, 0.05 equiv.) and DPEphos (7.3 mg, 0.0135 mmol, 0.15 equiv.) following the general procedure. Purification by column chromatography on silica gel using mixtures of DCM:MeOH (100:0 to 98:2 v/v) provided **compound 3b** (37.9 mg, 90% yield) as a colourless solid.

MW ($C_{23}H_{23}N_3O_6S$)**:** 469.51 g/mol; **Rf:** 0.24 (DCM/MeOH 98:2); **MP (ºC)**: >300 (dec); **IR (ATR) v (cm^{-1})**: 1703, 1323, 1158; **^{1}H-NMR (400 MHz, DMSO) δ (ppm)**: 11.18 (s, 2H, CONH), 7.63 (d, *J* = 8.2 Hz, 2H, H**13**), 7.45 (d, *J* = 8.2 Hz, 2H, H**14**), 3.20 (br s, 2H, H**11**), 3.12 – 3.05 (m, 3H, H**1**, H**2**), 2.84 (t, *J* = 5.6 Hz, 2H, H**12**), 2.78 (d, *J* = 7.8 Hz, 2H, H**5**), 2.40 (s, 3H, H**15**), 2.03 – 1.97 (m, 2H, H**10**), 1.63 (s, 3H, H9); **^{13}C-NMR (101 MHz, DMSO) δ (ppm):** 178.4 (C**3**/C**4**), 178.0 (C**3**/C**4**), 143.9 , 132.0, 131.4, 130.3, 130.0 (C**14**), 127.4 (C**13**), 49.4 (C**5**), 44.4 (C**11**), 44.3 (C**2**), 42.6 (C**12**), 41.1 (C**6**), 37.7 (C**1**), 28.8 (C**10**), 21.0 (C**15**), 15.5 (C**9**); **HRMS (ESI) m/z:** $[M + H]^+$ Calculated for $C_{23}H_{24}N_3O_6S$ 470.1380, Found: 470.1377.

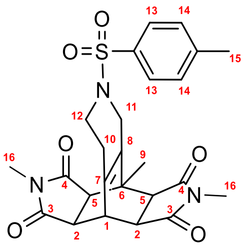

3c

Compound 3c was obtained from **allenyne 1a** (25.0 mg, 0.09 mmol, 1 equiv.) and **N-methylmaleimide 2c** (50 mg, 0.45 mmol, 5 equiv.) using a mixture of $[Rh(cod)Cl]_2$ (2.3 mg, 0,0046 mmol, 0.05 equiv.) and DPEphos (7.0 mg, 0.0130 mmol, 0.15 equiv.) following the general procedure. Purification by column chromatography on silica gel using mixtures of DCM:MeOH (100:0 to 99:1 v/v) provided **compound 3c** (41.2 mg, 92% yield) as a colourless solid.

MW ($C_{25}H_{27}N_3O_6S$)**:** 497.57 g/mol; **Rf:** 0.18 (DCM/MeOH 99:1); **MP (ºC)**: >300 (dec); **IR (ATR) v (cm^{-1})**: 1690, 1430, 1378; **^{1}H-NMR (400 MHz, $CDCl_3$) δ (ppm)**: 7.59 (d, *J* = 8.2 Hz, 2H, H**13**), 7.31 (d, *J* = 8.2 Hz, 2H, H**14**), 3.55 (t, *J* = 3.0 Hz, 1H, H**1**), 3.35 (t, *J* = 2.9 Hz, 2H, H**11**), 2.99 (dd, *J* = 8.0, 3.0 Hz, 2H, H**2**), 2.94 (t, *J* = 5.7 Hz, 2H, H**12**), 2.81 (s, 6H, H**16**), 2.66 (d, *J* = 8.0 Hz, 2H, H**5**), 2.42 (s, 3H, H**15**), 2.16 – 2.11 (m, 2H, H**10**), 1.84 (s, 3H, H**9**); **^{13}C-NMR (101 MHz,**

$CDCl_3$) δ (ppm): 176.3 (C**3**/C**4**), 175.5 (C**3**/C**4**), 143.9 , 134.0, 132.4, 131.6, 129.9 (C**14**), 127.6 (C**13**), 49.1 (C**5**), 44.1 (C**11**), 43.8 (C**2**), 42.7 (C**12**), 42.1 (C**6**), 38.2 (C**1**), 29.1 (C**10**), 25.0 (C**16**), 21.7 (C**15**), 16.3 (C**9**) ; **HRMS (ESI) m/z:** [M + H]$^+$ Calculated for $C_{25}H_{28}N_3O_6S$ 498.1693, Found: 498.1674.

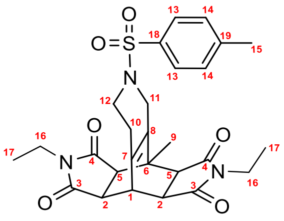

Compound 3d was obtained from **allenyne 1a** (24.9 mg, 0.09 mmol, 1 equiv.) and **N-ethylmaleimide 2d** (56.3 mg, 0.45 mmol, 5 equiv.) using a mixture of $[Rh(cod)Cl]_2$ (2.1 mg, 0,0043 mmol, 0.05 equiv.) and DPEphos (7.4 mg, 0.0137 mmol, 0.15 equiv.) following the general procedure. Purification by column chromatography on silica gel using mixtures of DCM:MeOH (100:0 to 99:1 v/v) provided **compound 3d** (41.6 mg, 88% yield) as a colourless solid.

MW ($C_{27}H_{31}N_3O_6S$)**:** 525.62 g/mol; **Rf:** 0.21 (DCM/MeOH 99:1); **MP (ºC)**: >280 (dec); **IR (ATR) v (cm^{-1})**: 1689, 1344, 1126; **^{1}H-NMR (400 MHz, $CDCl_3$) δ (ppm)**: 7.57 (d, *J* = 8.2 Hz, 2H, H**13**), 7.30 (d, *J* = 8.2 Hz, 2H, H**14**), 3.55 (t, *J* = 3.1 Hz, 1H, H**1**), 3.37 (q, *J* = 7.2 Hz, 4H, H**16**), 3.30 (t, *J* = 2.8 Hz, 2H, H**11**), 2.95 (dd, *J* = 8.0, 3.1 Hz, 2H, H**2**), 2.88 (t, *J* = 5.7 Hz, 2H, H**12**), 2.62 (d, *J* = 8.0 Hz, 2H, H**5**), 2.41 (s, 3H, H**15**), 2.19 – 2.13 (m, 2H, H**10**), 1.84 (s, 3H, H**9**) 0.89 (t, *J* = 7.2 Hz, 6H, H**17**); **^{13}C-NMR (101 MHz, $CDCl_3$) δ (ppm):** 176.1 (C**3**), 175.3 (C**4**), 144.0 (C**19**), 132.7 (C**18**), 132.0 (C**7**), 131.0 (C**8**), 129.9 (C**14**), 127.7 (C**13**), 49.0 (C**5**), 44.2 (C**11**), 43.6 (C**2**), 42.5 (C**12**), 42.2 (C**6**), 38.0 (C**1**), 33.9 (C**16**), 29.1 (C**10**), 21.6 (C**15**), 16.3 (C**9**), 13.2 (C**17**); **HRMS (ESI) m/z:** [M + H]$^+$ Calculated for $C_{27}H_{32}N_3O_6S$ 526.2006, Found: 526.1981.

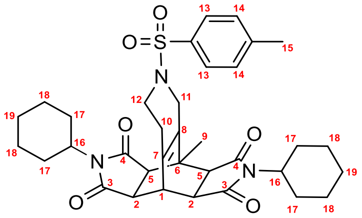

Compound 3e was obtained from **allenyne 1a** (25.0 mg, 0.09 mmol, 1 equiv.) and **maleimide 2e** (80.6 mg, 0.45 mmol, 5 equiv.) using a mixture of $[Rh(cod)Cl]_2$ (1.9 mg, 0,0039 mmol, 0.05 equiv.) and DPEphos (7.2 mg, 0.0134 mmol, 0.15 equiv.) following the general procedure. The resulting mixture was stirred for 24 h (TLC monitoring). Purification by column chromatography on silica gel using mixtures of DCM:MeOH (100:0 to 99:1 v/v) provided **compound 3e** (44.9 mg, 79% yield) as a colourless solid.

MW ($C_{35}H_{43}N_3O_6S$)**:** 633.80 g/mol; **Rf:** 0.20 (DCM/MeOH 99:1); **MP (ºC)**: >300 (dec); **IR (ATR) v (cm^{-1})**: 2928, 1688, 1339; **^{1}H-NMR (400 MHz, $CDCl_3$) δ (ppm)**: 7.58 (d, *J* = 8.0 Hz, 2H, H**13**), 7.31 (d, *J* = 8.0 Hz, 2H, H**14**), 3.76 (tt, *J* = 12.4, 3.9 Hz, 2H, H**16**), 3.50 (t, *J* = 3.2 Hz, 1H, H**1**), 3.28 (t, *J* = 2.7 Hz, 2H, H**11**), 2.91 – 2.84 (m, 4H, H**2**; H**12**), 2.54 (d, *J* = 8.1 Hz, 2H, H**5**), 2.41

(s, 3H, H**15**), 2.18 – 2.12 (m, 2H, H**10**), 1.99-1.85 (m, 4H, H**17**), 1.80 (s, 3H, H**9**) 1.76 – 1.66 (m, 4H, H**18**), 1.60 (m, 2H, H**19**), 1.32 – 1.04 (m, 10H, H**17**, H**18,** H**19**); **^{13}C-NMR (101 MHz, $CDCl_3$) δ (ppm):** 176.4 (C**3**/C**4**), 175.5 (C**3**/C**4**), 143.9 , 132.3, 131.9, 130.6, 129.8 (C**14**), 127.8 (C**13**), 51.9 (C**16**), 48.7 (C**5**), 44.3 (C**11**), 43.3 (C**2**), 42.7 (C**12**), 42.3 (C**6**), 38.2 (C**1**), 28.9 (C**10**), 28.6 (C**17**), 25.9 (C**18**), 25.0 (C**19**), 21.7 (C**15**), 16.2 (C**9**); **HRMS (ESI) m/z:** $[M + H]^+$ Calculated for $C_{35}H_{44}N_3O_6S$ 634.2945, Found: 634.2941.

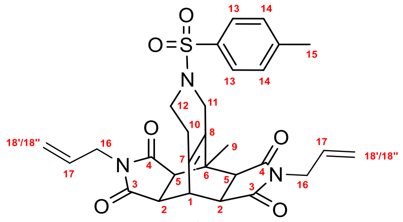

3f

Compound 3f was obtained from **allenyne 1a** (25.1 mg, 0.09 mmol, 1 equiv.) and **maleimide 2f** (62.0 mg, 0.45 mmol, 5 equiv.) using a mixture of $[Rh(cod)Cl]_2$ (2.5 mg, 0,005 mmol, 0.05 equiv.) and DPEphos (7.3 mg, 0.0135 mmol, 0.15 equiv.) following the general procedure. Purification by column chromatography on silica gel using mixtures of DCM:MeOH (100:0 to 99:1 v/v) provided **compound 3f** (45.9 mg, 90% yield) as a colourless solid.

MW ($C_{29}H_{31}N_3O_6S$)**:** 549.64 g/mol; **Rf:** 0.24 (DCM/MeOH 99:1); **MP (ºC)**: 210 – 215; **IR (ATR) ν (cm^{-1})**: 1691, 1391, 1166; **^{1}H-NMR (400 MHz, $CDCl_3$) δ (ppm**): 7.59 (d, *J* = 8.0 Hz, 2H, H**13**), 7.33 (d, *J* = 8.0 Hz, 2H, H**14**), 5.47 (ddt, *J* = 17.0, 10.1, 6.3 Hz, 2H, H**17**), 5.09 (dd, *J* = 17.0, 1.2 Hz, 2H, H**18'/18"**), 4.93 (dd, *J* = 10.1, 1.2 Hz, 2H, H**18'/18"**), 3.91 (d, *J* = 6.3 Hz, 4H, H**16**), 3.55 (t, *J* = 3.1 Hz, 1H, H**1**), 3.29 (t, *J* = 2.8 Hz, 2H, H**11**), 2.98 (dd, *J* = 8.1, 3.1 Hz, 2H, H**2**), 2.86 (t, *J* = 5.7 Hz, 2H, H**12**), 2.65 (d, *J* = 8.1 Hz, 2H, H**5**), 2.43 (s, 3H, H**15**), 2.15 – 2.08 (m, 2H, H**10**), 1.84 (s, 3H, H**9**); **^{13}C-NMR (101 MHz, $CDCl_3$) δ (ppm):** 175.7 (C**3**/C**4**), 174.9 (C**3**/C**4**), 143.9 , 132.9, 132.1, 130.9, 130.4 (C**17**), 129.9 (C**14**), 127.8 (C**13**), 119.8 (C**18**), 49.0 (C**5**), 44.1 (C**11**), 43.7 (C**2**), 42.3 (C**12**), 42.2 (C**6**), 41.2 (C**16**), 38.1 (C**1**), 28.8 (C**10**), 21.6 (C**15**), 16.3 (C**9**); **HRMS (ESI) m/z:** $[M + H]^+$ Calculated for $C_{29}H_{32}N_3O_6S$ 550.2006, Found: 550.2014.

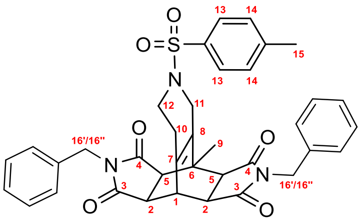

3g

Compound 3g was obtained from **allenyne 1a** (24.9 mg, 0.09 mmol, 1 equiv.) and **maleimide 2g** (84.1 mg, 0.45 mmol, 5 equiv.) using a mixture of $[Rh(cod)Cl]_2$ (2.0 mg, 0,0041 mmol, 0.05 equiv.) and DPEphos (7.3 mg, 0.0135 mmol, 0.15 equiv.) following the general procedure. Purification by column chromatography on silica gel using mixtures of DCM:MeOH (100:0 to 99:1 v/v) provided **compound 3g** (52.2 mg, 89% yield) as a colourless solid.

MW ($C_{37}H_{35}N_3O_6S$): 649.76 g/mol; **Rf:** 0.23 (DCM/MeOH 99:1); **MP (ºC)**: 183 – 187; **IR (ATR) ν (cm^{-1})**: 1695, 1393, 1337; **^{1}H-NMR (400 MHz, $CDCl_3$) δ (ppm):** 7.56 (d, *J* = 8.3 Hz, 2H, H**13**), 7.39 (d, *J* = 8.3 Hz, 2H, H**14**), 7.24 – 7.19 (m, 4H, **Ar**-H), 7.14 – 7.05 (m, 6H, **Ar**-H), 4.51 (d, *J* =

14.0 Hz, 2H, H**16'/16''**), 4.36 (d, *J* = 14.0 Hz, 2H, H**16'/16''**), 3.45 (t, *J* = 3.0 Hz, 1H, H**1**), 3.20 (t, *J* = 2.8 Hz, 2H, H**11**), 2.93 (dd, *J* = 8.1, 3.0 Hz, 2H, H**2**), 2.65 (d, *J* = 8.1 Hz, 2H, H**5**), 2.49 (s, 3H, H**15**),), 2.11 (t, *J* = 5.8 Hz, 2H, H**12**), 1.86 (s, 3H, H**9**), 1.81 – 1.75 (m, 2H, H**10**) (residual DCM solvent peak is observed); **^{13}C NMR (101 MHz, $CDCl_3$) δ (ppm)**: 175.8 (C**3**/C**4**), 175.1 (C**3**/C**4**), 143.7 , 135.6, 134.3, 132.1, 131.4, 129.9 (C**14**), 128.9 (C**18**), 128.7 (C**17**), 128.2 (C**19**), 127.8 (C**13**), 49.1 (C**5**), 43.9 (C**11**), 43.8 (C**2**), 42.7 (C**16**), 42.0 (C**6**), 41.5 (C**12**), 37.9 (C**1**), 28.9 (C**10**), 21.7 (C**15**), 16.4 (C**9**); **HRMS (ESI) m/z:** $[M + H]^+$ Calculated for $C_{37}H_{36}N_3O_6S$ 650,2319, Found: 650.2317**.**

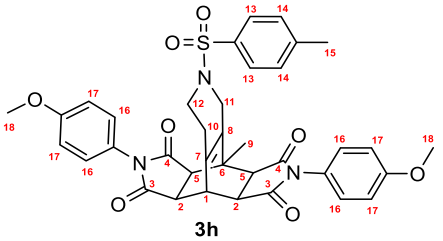

3h

Compound 3h was obtained from **allenyne 1a** (25.1 mg, 0.09 mmol, 1 equiv.) and **maleimide 2h** (91.4 mg, 0.45 mmol, 5 equiv.) using a mixture of $[Rh(cod)Cl]_2$ (2.2 mg, 0,0045 mmol, 0.05 equiv.) and DPEphos (7.3 mg, 0.0135 mmol, 0.15 equiv.) following the general procedure. Purification by column chromatography on silica gel using mixtures of DCM:MeOH (100:0 to 99:1 v/v) provided **compound 3h** (55.2 mg, 89% yield) as a colourless solid.

MW ($C_{37}H_{35}N_3O_8S$): 681.76 g/mol; **Rf:** 0.13 (DCM/MeOH 99:1); **MP (ºC)**: 172 – 175; **IR (ATR) ν (cm^{-1})**: 1703, 1509, 1162; **^{1}H-NMR (400 MHz, $CDCl_3$) δ (ppm):** 7.61 (d, *J* = 8.3 Hz, 2H, H**13**), 7.33 (d, *J* = 8.3 Hz, 2H, H**14**), 6.95 – 6.88 (m, 8H, H**16**, H**17**), 3.81 (s, 6H, H**18**), 3.72 (t, *J* = 3.1 Hz, 1H, H**1**), 3.48 (t, *J* = 2.8 Hz, 2H, H**11**), 3.19 (dd, *J* = 8.1, 3.0 Hz, 2H, H**2**), 2.97 (t, *J* = 5.8 Hz, 2H, H**12**), 2.84 (d, *J* = 8.1 Hz, 2H, H**5**), 2.46 (s, 3H, H**15**), 2.29 – 2.23 (m, 2H, H**10**), 1.92 (s, 3H, H**9**); **^{13}C NMR (101 MHz, $CDCl_3$) δ (ppm)**: 175.6 (C**3**/C**4**), 174.8 (C**3**/C**4**), 159.9, 144.0, 132.9, 132.8, 131.5, 130.0 (C**14**), 127.8 (C**13**), 127.7 (C**16**/C**17**), 123.9 , 114.8 (C**16/17**), 55.6 (C**18**), 49.1 (C**5**), 44.6 (C**11**), 43.7 (C**2**), 43.1 (C**12**), 42.7 (C**6**), 38.5 (C**1**), 29.4 (C**10**), 21.7 (C**15**), 16.3 (C**9**); **HRMS (ESI) m/z:** $[M + H]^+$ Calculated for $C_{37}H_{36}N_3O_8S$ 682.2218, Found: 682.2212**.**

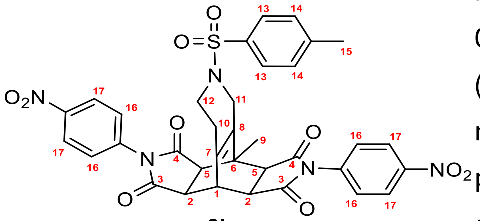

3i

Compound 3i was obtained from **allenyne 1a** (25.0 mg, 0.09 mmol, 1 equiv.) and **maleimide 2i** (98.2 mg, 0.45 mmol, 5 equiv) using a mixture of $[Rh(cod)Cl]_2$ (2.1 mg, 0,0043 mmol, 0.05 equiv.) and DPEphos (7.5 mg, 0.0139 mmol, 0.15 equiv.) following the general procedure. Purification by column chromatography on silica gel using mixtures of DCM:MeOH (100:0 to 99:1 v/v) provided **compound 3i** (57.2 mg, 90% yield) as a colourless solid.

MW ($C_{35}H_{29}N_5O_{10}S$): 711.70 g/mol; **Rf:** 0.15 (DCM/MeOH 99:1); **MP (ºC)**: 275 – 280; **IR (ATR) ν (cm^{-1})**: 2920, 1701, 1521, 1338; **^{1}H-NMR (400 MHz, CDCl$_3$) δ (ppm):** 8.25 (d, *J* = 9.0 Hz, 4H, H**17**), 7.60 (d, *J* = 8.2 Hz, 2H, H**13**), 7.35 (d, *J* = 8.2 Hz, 2H, H**14**), 7.30 (d, *J* = 9.0 Hz, 4H, H**16**), 3.77 (t, *J* = 2.8 Hz, 1H, H**1**), 3.51 (t, *J* = 2.9 Hz, 2H, H**11**), 3.32 (dd, *J* = 7.9, 2.8 Hz, 2H, H**2**), 2.97 (d, *J* = 7.9 Hz, 2H, H**5**), 2.93 (t, *J* = 5.6 Hz, 2H, H**12**), 2.48 (s, 3H, H**15**), 2.29 – 2.21 (m, 2H, H**10**), 1.96 (s, 3H, H**9**); **^{13}C NMR (101 MHz, CDCl$_3$) δ (ppm)**: 174.4 (C**3**/C**4**), 173.7 (C**3**/C**4**), 147.5, 144.5, 136.6, 133.3, 132.8, 132.1, 130.2 (C**14**), 127.7 (C**13**), 127.2 (C**16**), 124.7 (C**17**), 49.1 (C**5**), 44.6 (C**11**), 43.8 (C**2**), 43.0 (C**6**), 42.8 (C**12**), 38.5 (C**1**), 29.4 (C**10**), 21.7 (C**15**), 16.3 (C**9**); **HRMS (ESI) m/z:** [M + H]$^+$ Calculated for $C_{35}H_{30}N_5O_{10}S$ 712.1708, Found: 712.1692.

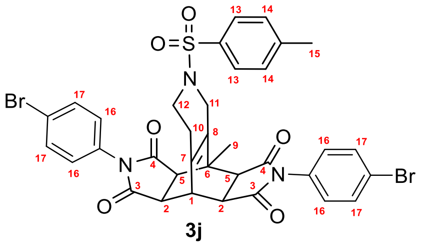

3j

Compound 3j was obtained from **allenyne 1a** (24.9mg, 0.09 mmol, 1 equiv.) and **maleimide 2j** (125.2 mg, 0.45 mmol, 5 equiv.) using a mixture of [Rh(cod)Cl]$_2$ (2.1 mg, 0,0043 mmol, 0.05 equiv.) and DPEphos (7.3 mg, 0.0135 mmol, 0.15 equiv.) following the general procedure. Purification by column chromatography on silica gel using mixtures of DCM:MeOH (100:0 to 99:1 v/v) provided **compound 3j** (59.8 mg, 85% yield) as a colourless solid.

MW ($C_{35}H_{29}Br_2N_3O_6S$): 779.50 g/mol; **Rf:** 0.16 (DCM/MeOH 99:1); **MP (ºC)**: >300 (dec); **IR (ATR) ν (cm^{-1})**: 2920, 1707, 1487; **^{1}H-NMR (400 MHz, CDCl$_3$) δ (ppm):**, 7.60 (d, *J* = 8.2 Hz, 2H, H**13**), 7.56 – 7.48 (m, 4H, H**17**), 7.33 (d, *J* = 8.2 Hz, 2H, H**14**), 6.96 – 6.88 (m, 4H, H**16**), 3.74 (t, *J* = 3.1 Hz, 1H, H**1**), 3.47 (t, *J* = 2.8 Hz, 2H, H**11**), 3.23 (dd, *J* = 8.2, 3.1 Hz, 2H, H**2**), 2.94 (t, *J* = 5.6 Hz, 2H, H**12**), 2.88 (d, *J* = 8.2 Hz, 2H, H**5**), 2.48 (s, 3H, H**15**), 2.28 – 2.21 (br, 2H, H**10**), 1.93 (s, 3H, H**9**); **^{13}C NMR (101 MHz, CDCl$_3$) δ (ppm)**: 174.9 (C**3**/C**4**), 174.1 (C**3**/C**4**), 144.2, 133.0, 132.8, 132.7 (C**17**), 131.8, 130.2, 130.1 (C**14**),128.0 (C**16**), 127.8 (C**13**),123.1, 49.1 (C**5**), 44.6 (C**11**), 43.7 (C**2**), 43.0 (C**12**), 42.7 (C**6**), 38.5 (C**1**), 29.4 (C**10**), 21.8 (C**15**), 16.3 (C**9**); **HRMS (ESI) m/z:** [M + H]$^+$ Calculated for $C_{35}H_{30}Br_2N_3O_6S$ 778.0217 (^{79}Br/^{79}Br), 780.0196 (^{79}Br/^{81}Br) and 782.0176 (^{81}Br/^{81}Br), Found: 778.0218 (^{79}Br/^{79}Br), 780.0212 (^{79}Br/^{81}Br) and 782.0200 (^{81}Br/^{81}Br).

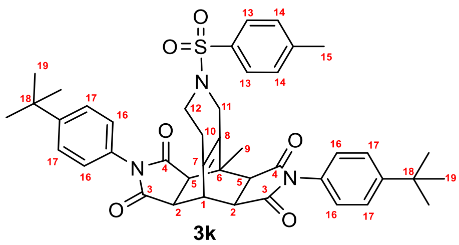

Compound 3k was obtained from **allenyne 1a** (25.0 mg, 0.09 mmol, 1 equiv.) and **maleimide 2k** (114.9 mg, 0.45 mmol, 5 equiv.) using a mixture of $[Rh(cod)Cl]_2$ (2.4 mg, 0,0049 mmol, 0.05 equiv.) and DPEphos (7.1 mg, 0.0132 mmol, 0.15 equiv.) following the general procedure. Purification by column chromatography on silica gel using mixtures of DCM:MeOH (100:0 to 99:1 v/v) provided **compound 3k** (60.0 mg, 91% yield) as a colourless solid.

MW ($C_{43}H_{47}N_3O_6S$): 733.92 g/mol; **Rf:** 0.21 (DCM/MeOH 99:1); **MP (ºC)**: 182 – 186; **IR (ATR) ν (cm^{-1})**: 2958, 1707, 1377; **^{1}H-NMR (400 MHz, $CDCl_3$) δ (ppm):**, 7.63 (d, J = 8.3 Hz, 2H, H**13**), 7.43 – 7.38 (m, 4H, H**17**), 7.33 (d, J = 8.3 Hz, 2H, H**14**), 6.95 – 6.87 (m, 4H, H**16**), 3.75 (t, J = 3.1 Hz, 1H, H**1**), 3.51 (t, J = 2.8 Hz, 2H, H**11**), 3.22 (dd, J = 8.2, 3.1 Hz, 2H, H**2**), 2.98 (t, J = 5.6 Hz, 2H, H**12**), 2.87 (d, J = 8.1 Hz, 2H, H**5**), 2.47 (s, 3H, H**15**), 2.28 – 2.20 (m, 2H, H**10**), 1.94 (s, 3H, H**9**) 1.32 (s, 18H, H**18**); **^{13}C NMR (101 MHz, $CDCl_3$) δ (ppm)**: 175.5 (C**3**/C**4**), 174.7 (C**3**/C**4**), 152.2, 143.9, 132.9, 132.8, 131.5, 130.0 (C**14**), 128.6, 127.9 (C**13**), 126.5 (C**16**), 125.9 (C**17**), 49.1 (C**5**), 44.6 (C**11**), 43.8 (C**2**), 43.0 (C**12**), 42.7 (C**6**), 38.5 (C**1**), 34.9 (C**18**), 31.4 (C**19**), 29.3 (C**10**), 21.8 (C**15**), 16.3 (C**9**); **HRMS (ESI) m/z:** [M + H]$^+$ Calculated for $C_{43}H_{48}N_3O_6S$ 734.3258, Found: 734.3253.

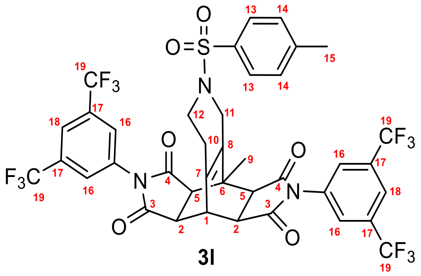

Compound 3l was obtained from **allenyne 1a** (24.9 mg, 0.09 mmol, 1 equiv.) and **maleimide 2l** (139.1 mg, 0.45 mmol, 5 equiv.) using a mixture of $[Rh(cod)Cl]_2$ (2.5 mg, 0,0051 mmol, 0.05 equiv.) and DPEphos (7.0 mg, 0.0130 mmol, 0.15 equiv.) following the general procedure. Purification by column chromatography on silica gel using mixtures of DCM:MeOH (100:0 to 99:1 v/v) provided **compound 3l** (64.9 mg, 81% yield) as a colourless solid.

MW ($C_{39}H_{27}F_{12}N_3O_6S$): 893.70 g/mol; **Rf:** 0.11 (DCM/MeOH 99:1); **MP (ºC)**: >300 (dec); **IR (ATR) ν (cm^{-1})**: 1717, 1388, 1126; **^{1}H-NMR (400 MHz, DMSO) δ (ppm):** 8.24 (s, 2H, H**17**), 7.75 (s, 4H, H**16**), 7.62 (d, J = 8.3 Hz, 2H, H**13**), 7.38 (d, J = 8.3 Hz, 2H, H**14**), 3.60 (dd, J = 8.1, 3.0 Hz, 2H, H**2**), 3.37 – 3.35 (m, 3H, H**1;**H**11**), 3.28 (d, J = 8.1 Hz, 2H, H**5**), 2.87 (t, J = 5.7 Hz, 2H, H**12**), 2.36 (s, 3H, H**15**), 2.14 – 2.04 (br s, 2H, H**10**), 1.78 (s, 3H, H**9**); **^{13}C NMR (101 MHz, DMSO) δ (ppm)**: 175.8 (C**3**/C**4**), 174.9 (C**3**/C**4**), 143.7, 133.6, 132.5, 132.4, 131.2, 131.1 (q, $^2J_{C-F}$ = 33 Hz, 4C, C**17**), 129.8 (C**14**), 127.5 (C**16**), 127.3 (C**13**), 122.7 (q, $^1J_{C-F}$ = 275 Hz, 4C, C**19**), 122.6 (C**18**), 48.4 (C**5**), 44.3 (C**11**), 43.4 (C**2**), 42.8 (C**12**), 41.9 (C**6**), 38.3 (C**1**), 29.2

(C**10**), 20.8 (C**15**), 15.4 (C**9**); **^{19}F NMR (377 MHz, DMSO) δ (ppm)**: -66.5; **HRMS (ESI) m/z:** $[M + H]^+$ Calculated for $C_{39}H_{28}F_{12}N_3O_6S$ 894.1502, Found: 894.1500.

S3.2 Allenynes' scope

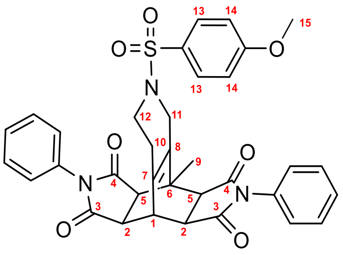

3m

Compound 3m was obtained from **allenyne 1b** (26.2 mg, 0.09 mmol, 1 equiv.) and **maleimide 2a** (78.0 mg, 0.45 mmol, 5 equiv.) using a mixture of $[Rh(cod)Cl]_2$ (2.2 mg, 0,0045 mmol, 0.05 equiv.) and DPEphos (7.6 mg, 0.0141 mmol, 0.15 equiv.) following the general procedure. Purification by column chromatography on silica gel using mixtures of DCM:MeOH (100:0 to 99:1 v/v) provided **compound 3m** (47.6 mg, 83% yield) as a colourless solid.

MW ($C_{35}H_{31}N_3O_7S$): 637.70 g/mol; **Rf:** 0.23 (DCM/MeOH 99:1); **MP (ºC)**: 163 – 167; **IR (ATR) ν (cm^{-1})**: 1704, 1374, 1148; **^{1}H-NMR (400 MHz, $CDCl_3$) δ (ppm)**: 7.70 – 7.63 (m, 2H, H**13**), 7.46 – 7.34 (m, 6H, **Ar**-H), 7.04 – 6.95 (m, 6H, **Ar**-H/H**14**), 3.89 (s, 3H, H**15**), 3.76 (t, *J* = 3.1 Hz, 1H, H**1**), 3.50 (t, *J* = 2.6 Hz, 2H, H**11**), 3.24 (dd, *J* = 8.2, 3.1 Hz, 2H, H**2**), 2.96 (t, *J* = 5.6 Hz, 2H, H**12**), 2.89 (d, *J* = 8.2 Hz, 2H, H**5**), 2.30 – 2.24 (m, 2H, H**10**), 1.95 (s, 3H, H**9**); **^{13}C-NMR (101 MHz, $CDCl_3$) δ (ppm):** 175.3 (C**3**/C**4**), 174.5 (C**3**/C**4**), 163.3, 132.9, 131.6, 131.3, 130.0 (C**13**), 129.5, 129.2, 127.2, 126.5, 114.6 (C**14**), 55.8 (C**15**), 49.2 (C**5**), 44.6 (C**11**), 43.8 (C**2**), 43.1 (C**12**), 42.7 (C**6**), 38.5 (C**1**), 29.3 (C**10**), 16.4 (C**9**); **HRMS (ESI) m/z:** $[M + H]^+$ Calculated for $C_{35}H_{32}N_3O_7S$ 638.1955, Found: 638.1943.

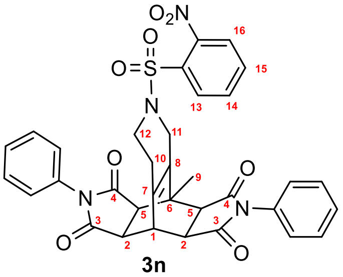

3n

Compound 3n was obtained from **allenyne 1c** (27.6 mg, 0.09 mmol, 1 equiv.) and **maleimide 2a** (78.1 mg, 0.45 mmol, 5 equiv.) using a mixture of $[Rh(cod)Cl]_2$ (2.4 mg, 0,0049 mmol, 0.05 equiv.) and DPEphos (7.4 mg, 0.0137 mmol, 0.15 equiv.) following the general procedure. Purification by column chromatography on silica gel using mixtures of DCM:MeOH (100:0 to 99:1 v/v) provided **compound 3n** (51.2 mg, 86% yield) as a colourless solid.

MW ($C_{34}H_{28}N_4O_8S$): 652.59 g/mol; **Rf:** 0.18 (DCM/MeOH 99:1); **MP (ºC)**: 182 – 187; **IR (ATR) ν (cm^{-1})**: 2923, 1706, 1376; **^{1}H-NMR (400 MHz, $CDCl_3$) δ (ppm)**: 7.94 (dd, *J* = 7.5, 1.8 Hz, 1H, H**16**), 7.69 – 7.59 (m, 2H, H**14**,H**15**), 7.56 (dd, *J* = 7.5, 1.8 Hz, 1H, H**13**), 7.48 – 7.33 (m, 6H, **Ar**-H), 7.15 – 7.09 (m, 4H, **Ar**-H), 3.80 (t, *J* = 3.1 Hz, 1H, H**1**), 3.77 (t, *J* = 2.7 Hz, 2H, H**11**), 3.33

(t, J = 5.6 Hz, 2H, H**12**), 3.25 (dd, J = 8.2, 3.1 Hz, 2H, H**2**), 2.92 (d, J = 8.2 Hz, 2H, H**2**), 2.35 – 2.31 (m, 2H, H**10**), 1.98 (s, 3H, H**9**); **^{13}C-NMR (101 MHz, $CDCl_3$) δ (ppm):** 175.3 (C**3**/C**4**), 174.5 (C**3**/C**4**), 148.2, 133.9, 133.1, 132.0, 131.9, 131.8, 131.3 (C**16**), 131.2, 129.5, 129.1, 126.5, 124.4 (C**13**), 49.2 (C**5**), 44.3 (C**11**), 44.0 (C**2**), 42.9 (C**12**), 42.6 (C**6**), 38.7 (C**1**), 29.5 (C**10**), 16.4 (C**9**); **HRMS (ESI) m/z:** [M + H]$^+$ Calculated for $C_{34}H_{29}N_4O_8S$ 653.1701, Found: 653.1699.

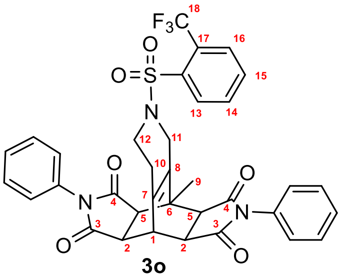

Compound 3o was obtained from **allenyne 1d** (29.6 mg, 0.09 mmol, 1 equiv.) and **maleimide 2a** (77.9 mg, 0.45 mmol, 5 equiv.) using a mixture of $[Rh(cod)Cl]_2$ (2.1 mg, 0,0043 mmol, 0.05 equiv.) and DPEphos (7.0 mg, 0.0130 mmol, 0.15 equiv.) following the general procedure. Purification by column chromatography on silica gel using mixtures of DCM:MeOH (100:0 to 99:1 v/v) provided **compound 3o** (45.2 mg, 74% yield) as a colourless solid.

MW ($C_{35}H_{28}F_3N_3O_6S$): 675.68 g/mol; **Rf:** 0.22 (DCM/MeOH 99:1); **MP (ºC)**: 158 – 163; **IR (ATR) ν (cm^{-1})**: 1705, 1305, 1161; **^{1}H-NMR (400 MHz, $CDCl_3$) δ (ppm)**: 8.05 (dd, J = 7.5, 1.9 Hz, 1H, H**16**), 7.85 (dd, J = 7.5, 1.9, 1H, H**13**), 7.70 – 7.58 (m, 2H, H**14**/H**15**), 7.49 – 7.34 (m, 6H, **Ar**-H), 7.16 – 7.09 (m, 4H, **Ar**-H), 3.79 (t, J = 3.1Hz, 1H, H**1**), 3.73 (t, J = 2.6 Hz, 2H, H**11**), 3.28 – 3.21 (m, 4H, H**2**/H**12**), 2.91 (d, J = 8.2 Hz, 2H, H**5**), 2.35 – 2.22 (m, 2H, H**10**), 1.95 (s, 3H, H**9**) (residual acetone solvent peak is observed); **^{13}C-NMR (101 MHz, $CDCl_3$) δ (ppm):** 175.3 (C**3**/C**4**), 174.5 (C**3**/C**4**), 137.6, 133.0, 132.5, 132.1 (C**16**), 132.0, 131.3, 129.4, 129.1 (C**13**), 128.8, 128.7, 128.0 (q, $^2J_{C-F}$ = 30 Hz, C**17**), 126.5, 122.6 (q, $^1J_{C-F}$ = 273 Hz, C**18**), 49.2 (C**5**), 44.0 (C**2**) (C**11**), 42.6 (C**12**), 42.5 (C**6**), 38.7 (C**1**), 29.2 (C**10**), 16.3 (C**9**); **^{19}F NMR (377 MHz, $CDCl_3$) δ (ppm)**: -58.8; **HRMS (ESI) m/z:** [M + H]$^+$ Calculated for $C_{35}H_{29}F_3N_3O_6S$ 676.1725, Found: 676.1724.

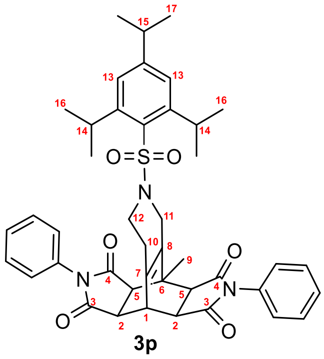

Compound 3p was obtained from **allenyne 1e** (34.9 mg, 0.09 mmol, 1 equiv.) and **maleimide 2a** (78.2 mg, 0.45 mmol, 5 equiv.) using a mixture of $[Rh(cod)Cl]_2$ (2.3 mg, 0,0046 mmol, 0.05 equiv.) and DPEphos (7.4 mg, 0.0137 mmol, 0.15 equiv.) following the general procedure. Purification by column chromatography on silica gel using mixtures of DCM:MeOH (100:0 to 99:1 v/v) provided **compound 3p** (56.1 mg, 85% yield) as a colourless solid.

MW ($C_{43}H_{47}N_3O_6S$): 733.92 g/mol; **Rf:** 0.20 (DCM/MeOH 99:1); **MP (ºC)**: 163 – 167; **IR (ATR) ν (cm^{-1})**: 2955, 1708, 1370; **^{1}H-NMR (400 MHz, $CDCl_3$) δ (ppm)**: 7.47 – 7.32 (m, 6H, **Ar**-H), 7.21 – 7.16 (m, 4H, **Ar**-H), 7.12 (s, 2H, H**13**), 3.97 (hept, *J* = 6.8 Hz, 2H, H**14**), 3.79 (t, *J* = 3.1 Hz, 1H, H**1**), 3.67 (t, *J* = 2.7 Hz, 2H, H**11**), 3.25 (dd, *J* = 8.3, 3.1 Hz, 2H, H**2**), 3.18 (t, *J* = 5.5 Hz, 2H, H**12**), 2.90 (d, *J* = 8.3 Hz, 2H, H**5**), 2.89 (hept, *J* = 7.0 Hz, 1H, H**15** overlaped), 2.32 – 2.25 (m, 2H, H**10**), 1.95 (s, 3H, H**9**), 1.24 (d, *J* = 7.0 Hz, 6H, H**17**), 1.12 (d, *J* = 6.8 Hz, 12H, H**16**); **^{13}C-NMR (101 MHz, $CDCl_3$) δ (ppm):** 175.3 (C**3**/C**4**), 174.5 (C**3**/C**4**), 153.4 (C**15**), 151.6 (C**14**), 133.2, 132.1, 131.4, 130.3, 129.3, 128.9, 126.5, 124.2 (C**13**), 49.4 (C**5**), 44.2 (C**2**), 43.0 (C**11**), 42.6 (C**6**), 41.6 (C**12**), 38.7 (C**1**), 34.3 (C**15**), 29.6 (C**14**), 28.8 (C**10**), 24.9 (C**16**), 23.7 (C**17**), 16.4 (C**9**); **HRMS (ESI) m/z:** $[M + H]^+$ Calculated for $C_{43}H_{48}N_3O_6S$ 734.3258, Found: 734.3257.

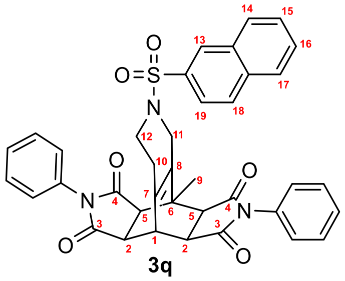

3q

Compound 3q was obtained from **allenyne 1f** (28.1 mg, 0.09 mmol, 1 equiv.) and **maleimide 2a** (77.9 mg, 0.45 mmol, 5 equiv.) using a mixture of $[Rh(cod)Cl]_2$ (2.5 mg, 0,0051 mmol, 0.05 equiv.) and DPEphos (7.1 mg, 0.0132 mmol, 0.15 equiv.) following the general procedure. Purification by column chromatography on silica gel using mixtures of DCM:MeOH (100:0 to 99:1 v/v) provided **compound 3q** (53.3 mg, 90% yield) as a colourless solid.

MW ($C_{38}H_{31}N_3O_6S$): 657.74 g/mol; **Rf:** 0.22 (DCM/MeOH 99:1); **MP (ºC)**: 185 – 192; **IR (ATR) ν (cm^{-1})**: 1705, 1373, 1162; **^{1}H-NMR (400 MHz, $CDCl_3$) δ (ppm)**: 8.32 (br. s, 1H, H**13**), 8.01 – 7.92 (m, 3H, **Ar**-H), 7.75 – 7.66 (m, 2H, **Ar**-H), 7.66 – 7.62 (m, 1H, **Ar**-H), 7.34 – 7.26 (m, 2H, **Ar**-H), 7.28 – 7.19 (m, 4H, **Ar**-H), 6.98 – 6.91 (m, 4H, **Ar**-H), 3.75 (t, *J* = 3.1 Hz, 1H, H**1**), 3.62 (t, *J* = 2.7 Hz, 2H, H**11**), 3.23 (dd, *J* = 8.2, 3.1 Hz, 2H, H**2**), 3.05 (t, *J* = 5.6 Hz, 2H, H**12**), 2.88 (d, *J* = 8.2 Hz, 2H, H**5**), 2.31 – 2.27 (m, 2H, H**10**), 1.96 (s, 3H, H**9**) (residual DCM solvent peak is observed); **^{13}C-NMR (101 MHz, $CDCl_3$) δ (ppm):** 175.3 (C**3**/C**4**), 174.5 (C**3**/C**4**), 135.1, 133.0, 132.9, 132.4, 131.6, 131.2, 129.6, 129.5, 129.4, 129.2, 129.1, 129.0, 128.1, 127.9, 126.4, 122.8, 49.1 (C**5**), 44.7 (C**11**), 43.8 (C**2**), 43.2 (C**12**), 42.8 (C**6**), 38.6 (C**1**), 29.4 (C**10**), 16.4 (C**9**); **HRMS (ESI) m/z:** $[M + H]^+$ Calculated for $C_{38}H_{32}N_3O_6S$ 658.2006, Found: 658.2005.

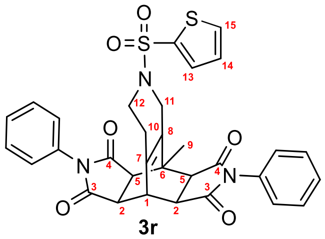

3r

Compound 3r was obtained from **allenyne 1g** (26.8 mg, 0.09 mmol, 1 equiv.) and **maleimide 2a** (78.1 mg, 0.45 mmol, 5 equiv.) using a mixture of $[Rh(cod)Cl]_2$ (2.1 mg, 0.0043 mmol, 0.05 equiv.) and DPEphos (7.4 mg, 0.0137 mmol, 0.15 equiv.) following the general procedure. Purification by column chromatography on silica gel using mixtures of DCM:MeOH (100:0 to 99:1 v/v) provided **compound 3r** (48.9 mg, 89% yield) as a colourless solid.

MW ($C_{32}H_{27}N_3O_6S_2$): 613.70 g/mol; **Rf:** 0.28 (DCM/MeOH 99:1); **MP (ºC)**: 161 – 168; **IR (ATR) ν (cm^{-1})**: 2922, 1705, 1375; **^{1}H-NMR (400 MHz, $CDCl_3$) δ (ppm)**: 7.63 (dd, *J* = 5.0, 1.3 Hz, 1H, H**13**), 7.53 (dd, *J* = 3.7, 1.3 Hz, 1H, H**15**), 7.49 – 7.34 (m, 6H, **Ar**-H), 7.16 (dd, *J* = 5.0, 3.7 Hz, 1H, H**14**), 7.05 – 6.98 (m, 4H, **Ar**-H), 3.76 (t, *J* = 3.1 Hz, 1H, H**1**), 3.58 (t, *J* = 2.8 Hz, 2H, H**11**), 3.23 (dd, *J* = 8.1, 3.1 Hz, 2H, H**2**), 3.02 (t, *J* = 5.6 Hz, 2H, H**12**), 2.89 (d, *J* = 8.1 Hz, 2H, H**5**), 2.35 – 2.26 (m, 2H, H**10**), 1.96 (s, 3H, H**9**); **^{13}C-NMR (101 MHz, $CDCl_3$) δ (ppm):** 175.3 (C**3**/C**4**), 174.5 (C**3**/C**4**), 135.8, 133.0, 132.9 (C**15**), 132.5 (C**13**), 131.4, 131.3, 129.5, 129.2, 127.9 (C**14**), 126.5, 49.1 (C**5**), 44.7 (C**11**), 43.8 (C**2**), 43.2 (C**12**), 42.7 (C**6**), 38.5 (C**1**), 29.3 (C**10**), 16.3 (C**9**); **HRMS (ESI) m/z:** $[M + H]^+$ Calculated for $C_{32}H_{28}N_3O_6S_2$ 614.1411, Found: 614.1411.

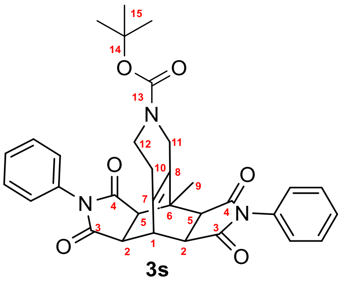

3s

Compound 3s was obtained from **allenyne 1h** (19.8 mg, 0.09 mmol, 1 equiv.) and **maleimide 2a** (78.0 mg, 0.45 mmol, 5 equiv.) using a mixture of $[Rh(cod)Cl]_2$ (2.3 mg, 0,0047 mmol, 0.05 equiv.) and DPEphos (7.4 mg, 0.0137 mmol, 0.15 equiv.) following the general procedure. Purification by column chromatography on silica gel using mixtures of DCM:MeOH (100:0 to 99:1 v/v) provided **compound 3s** (24.5 mg, 48% yield) as a colourless solid.

MW ($C_{33}H_{33}N_3O_6$): 567.64 g/mol; **Rf:** 0.4 (DCM/MeOH 99:1); **MP (ºC)**: 165 – 168; **IR (ATR) ν (cm^{-1})**: 1709, 1496, 1374; **^{1}H-NMR (400 MHz, $CDCl_3$, 40 ºC) δ (ppm)**: 7.48 – 7.33 (m, 6H, **Ar**-H), 7.14 – 7.06 (m, 4H, **Ar**-H), 3.90 (br s, 2H, H**11**), 3.76 (t, *J* = 3.1 Hz, 1H, H**1**), 3.38 (t, *J* = 5.4 Hz, 2H, H**12**), 3.25 (dd, *J* = 8.2, 3.1 Hz, 2H, H**2**), 2.91 (d, *J* = 8.2 Hz, 2H, H**5**), 2.22 – 2.11 (m, 2H, H**10**), 1.99 (s, 3H, H**9**), 1.40 (s, 9H, H**15**) (broad peaks were observed at 25 ºC) (residual DCM solvent peak is observed); **^{13}C-NMR (101 MHz, $CDCl_3$) δ (ppm)**: 175.5 (C**3**/C**4**), 174.6 (C**3**/C**4**), 154.5 (C**13**), 132.6, 131.5, 129.4, 129.1, 126.4, 80.3 (C**14**), 49.2 (C**5**), 43.9 (C**2**),

42.5 (C**11**), 38.7 (C**1**), 29.0 (C**10**), 28.5 (C**15**), 16.3 (C**9**); **HRMS (ESI) m/z:** $[M + Na]^+$ Calculated for $C_{33}H_{33}N_3O_6Na$ 590.2262, Found: 590.2250.

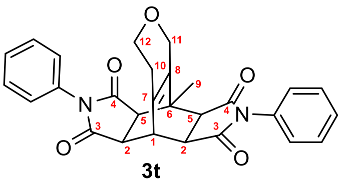

3t

Compound 3t was obtained from **allenyne 1i** (11.1 mg, 0.09 mmol, 1 equiv.) and **maleimide 2a** (77.8 mg, 0.45 mmol, 5 equiv.) using a mixture of $[Rh(cod)Cl]_2$ (2.1 mg, 0,0043 mmol, 0.05 equiv.) and DPEphos (7.0 mg, 0.0130 mmol, 0.15 equiv.) following the general procedure. Purification by column chromatography on silica gel using mixtures of DCM:MeOH (100:0 to 99:1 v/v) provided **compound 3t** (24.5 mg, 58% yield) as a colourless solid.

MW ($C_{28}H_{24}N_2O_5$): 468.51 g/mol; **Rf:** 0.32 (DCM/MeOH 99:1); **MP (ºC)**: >270 (dec); **IR (ATR) ν (cm^{-1})**: 2920, 1702, 1374; **^{1}H-NMR (400 MHz, $CDCl_3$) δ (ppm)**: 7.50 – 7.38 (m, 6H, **Ar**-H), 7.17 – 7.13 (m, 4H, **Ar**-H), 4.07 (t, *J* = 2.9 Hz, 2H, H**11**), 3.76 (t, *J* = 3.1 Hz, 1H, H**1**), 3.67 (t, *J* = 5.5 Hz, 2H, H**12**), 3.26 (dd, *J* = 8.1, 3.1 Hz, 2H, H**2**), 2.91 (d, *J* = 8.1 Hz, 2H, H**5**), 2.20 – 2.14 (m, 2H, H**10**), 1.90 (s, 3H, H**9**); **^{13}C-NMR (101 MHz, $CDCl_3$) δ (ppm):** 175.6 (C**3**/C**4**), 174.8 (C**3**/C**4**), 135.1, 131.7, 131.6, 129.6, 129.2, 126.6, 64.8 (C**11**), 64.5 (C**12**), 49.3 (C**5**), 44.0 (C**2**), 42.1 (C**6**), 38.6 (C**1**), 28.5 (C**10**), 15.9 (C**9**); **HRMS (ESI) m/z:** $[M + Na]^+$ Calculated for $C_{28}H_{24}N_2O_5Na$ 491.1577, Found: 491.1578.

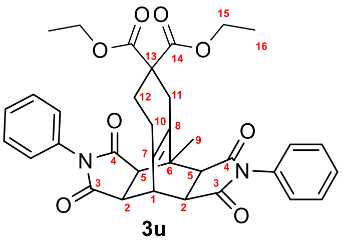

3u

Compound 3u was obtained from **allenyne 1j** (23.8 mg, 0.09 mmol, 1 equiv.) and **maleimide 2a** (77.8 mg, 0.45 mmol, 5 equiv.) using a mixture of $[Rh(cod)Cl]_2$ (2.1 mg, 0,0043 mmol, 0.05 equiv.) and DPEphos (7.5 mg, 0.0139 mmol, 0.15 equiv.) following the general procedure in an anhydrous mixture of PhCl/EtOH/TFA (5:1:1.7x10^{-6}) (5 mL). The resulting mixture was heated at 130 ºC and stirred for 16 h (TLC monitoring). Purification by column chromatography on silica gel using mixtures of DCM:MeOH (100:0 to 99:1 v/v) provided **compound 3u** (38.4 mg, 70% yield) as a colourless solid.

MW ($C_{35}H_{34}N_2O_8$): 610.66 g/mol; **Rf:** 0.41 (DCM/MeOH 99:1); **MP (ºC)**: 125 – 128; **IR (ATR) ν (cm^{-1})**: 1702, 1376, 1179; **^{1}H-NMR (400 MHz, $CDCl_3$) δ (ppm)**: 7.49 – 7.41 (m, 4H, **Ar**-H), 7.40 – 7.34 (m, 2H, **Ar**-H), 7.30 – 7.24 (m, 4H, **Ar**-H), 4.05 – 3.89 (m, 4H, H**14**), 3.70 (t, *J* = 3.0 Hz, 1H, H**1**), 3.17 (dd, *J* = 8.4, 3.0 Hz, 2H, H**2**), 2.88 (d, *J* = 8.4 Hz, 2H, H**5**), 2.53 (t, *J* = 2.5 Hz, 2H, H**11**), 2.16 – 2.10 (m, 2H, H**10**), 2.02 (s, 3H , H**9**), 1.96 (t, *J* = 6.2 Hz, 2H, H**12**), 1.10 (t, *J* = 7.1 Hz, 6H, H**15**) (residual DCM solvent peak is observed); **^{13}C-NMR (101 MHz, $CDCl_3$) δ (ppm):**

175.6 (C**3**/C**4**), 174.7 (C**3**/C**4**), 170.6 (C**14**), 133.0, 132.2, 131.7, 129.0, 128.7, 126.5, 61.6 (C**15**), 52.9 (C**13**), 49.5 (C**5**), 44.3 (C**2**), 43.2 (C**6**), 38.6 (C**1**), 30.7 (C**11**), 27.2 (C**12**), 26.1 (C**10**), 17.4 (C**9**), 14.0 (C**16**); **HRMS (ESI) m/z:** $[M + Na]^+$ Calculated for $C_{35}H_{34}N_2O_8Na$ 633.2207, Found: 633.220.

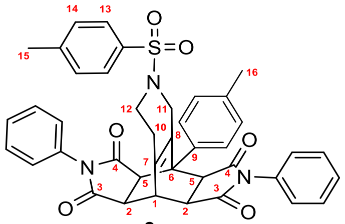

3v

Compound 3v was obtained from **allenyne 1l** (31.7 mg, 0.09 mmol, 1 equiv.) and **maleimide 2a** (78.3 mg, 0.45 mmol, 5 equiv.) using a mixture of $[Rh(cod)Cl]_2$ (2.1 mg, 0,0043 mmol, 0.05 equiv.) and DPEphos (7.3 mg, 0.0135 mmol, 0.15 equiv.) following the general procedure and stirred for 24 h (TLC monitoring). Purification by column chromatography on silica gel using mixtures of Hexanes:EtOAc (1:1 v/v) provided **compound 3v** (33.4 mg, 54% yield) as a colourless solid.

MW ($C_{41}H_{35}N_3O_6S$): 697.81 g/mol; **Rf:** 0.2 (Hexanes:EtOAc 1:1); **MP (ºC)**: 140 – 145; **IR (ATR) ν (cm^{-1})**: 1708, 1370, 1186; **^{1}H-NMR (400 MHz, $CDCl_3$) δ (ppm)**: 7.53 (d, *J* = 8.2, 2H, H**13**), 7.43 – 7.31 (m, 7H, **Ar**-H), 7.29 (d, *J* = 8.2 Hz, 2H, H**14**), 7.22 – 7.18 (m, 3H, **Ar**-H), 7.16 – 7.10 (m, 4H, **Ar**-H), 3.76 (t, *J* = 2.9 Hz, 1H, H**1**), 3.57 (t, *J* = 2.8 Hz, 2H, H**11**), 3.53 (d, *J* = 8.2 Hz, 2H, H**5**), 3.27 (dd, *J* = 8.2, 2.9 Hz, 2H, H**2**), 3.05 (t, *J* = 5.8 Hz, 2H, H**12**), 2.43 (s, 3H, H**15**), 2.45 – 2.40 (br s, 2H, H**10**), 2.38 (s, 3H, H**16**); **^{13}C NMR (101 MHz, $CDCl_3$) δ** 175.1 (C**3**/C**4**), 173.5 (C**3**/C**4**), 144.1, 138.1, 136.0, 132.3, 131.4, 130.9, 130.7, 130.0 (C**14**), 129.7, 129.3, 129.0, 128.3, 127.9 (C**13**), 126.6, 51.9 (C**9**), 48.6 (C**5**), 48.2 (C**11**), 44.1 (C**2**), 43.4 (C**12**), 38.6 (C**1**), 30.6 (C**10**), 21.7 (C**15**), 21.2 (C**16**); **HRMS (ESI) m/z:** $[M + Na]^+$ Calculated for $C_{41}H_{35}N_3O_6SNa$ 720.2139, Found: 720.2137.

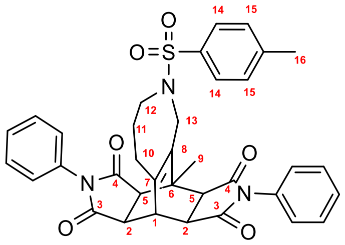

3w

Compound 3w was obtained from **allenyne 1n** (26.1 mg, 0.09 mmol, 1 equiv.) and **maleimide 2a** (78.3 mg, 0.45 mmol, 5 equiv.) using a mixture of $[Rh(cod)Cl]_2$ (2.2 mg, 0.0051 mmol, 0.05 equiv.) and DPEphos (7.5 mg, 0.0139 mmol, 0.15 equiv.) following the general procedure and stirred for 24h (TLC monitoring). Purification by column chromatography on silica gel using mixtures of DCM/MeOH (100:0 to 99:1 v/v) provided **compound 3w** (18.9 mg, 33% yield) as a colourless solid.

MW ($C_{36}H_{33}N_3O_6S$): 635.74 g/mol; **Rf:** 0.24 (DCM/MeOH 99:1); **MP (ºC)**: 167 – 172; **IR (ATR) ν (cm^{-1})**: 2943, 1705, 1346; **^{1}H-NMR (400 MHz, $CDCl_3$) δ (ppm)**: 7.59 (d, *J* = 8.3 Hz, 2H, H**14**), 7.48 – 7.41 (m, 4H, **Ar**-H), 7.40 – 7.35 (m, 2H, **Ar**-H), 7.29 – 7.25 (m, 6H, **Ar**-H/H**15**), 3.79 (t, *J* = 3.1 Hz, 1H, H**1**), 3.74 (s, 2H, H**13**), 3.20 (dd, *J* = 8.4, 3.1 Hz, 2H, H**2**), 3.15 (t, *J* = 6.4 Hz, 2H,

H**12**), 2.86 (d, *J* = 8.4 Hz, 2H, H**5**), 2.46 – 2.41 (m, 2H, H**10**), 2.40 (s, 3H, H**16**), 2.07 (s, 3H, H**9**), 1.73 – 1.62 (m, 2H, H**11**) (residual DCM solvent peak is observed); **^{13}C-NMR (101 MHz, $CDCl_3$) δ (ppm):** 175.7 (C**3**/C**4**), 174.7 (C**3**/C**4**), 143.6, 139.4, 135.0, 134.8, 131.5, 130.0, 129.3, 128.9, 127.4, 126.6, 49.3 (C**12**), 49.2 (C**5**), 46.9 (C**13**), 44.1 (C**6**), 43.8 (C**2**), 41.8 (C**1**), 32.1 (C**10**), 26.6 (C**11**), 21.6 (C**16**), 18.2 (C**9**); **HRMS (ESI) m/z:** [M + Na]$^+$ Calculated for $C_{36}H_{33}N_3O_6SNa$ 658.1982, Found: 658.1978.

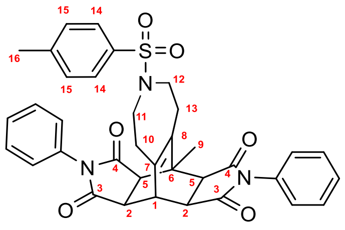

3x

Compound 3x was obtained from **allenyne 1o** (26.4 mg, 0.09 mmol, 1 equiv.) and **maleimide 2a** (79.2 mg, 0.45 mmol, 5 equiv.) using a mixture of $[Rh(cod)Cl]_2$ (2.1 mg, 0.0051 mmol, 0.05 equiv.) and DPEphos (7.4 mg, 0.0139 mmol, 0.15 equiv.) following the general procedure and stirred for 24h (TLC monitoring). Purification by column chromatography on silica gel using mixtures of DCM/MeOH (100:0 to 99:1 v/v) provided **compound 3x** (21.4 mg, 37% yield) as a colourless solid.

MW ($C_{36}H_{33}N_3O_6S$): 635.74 g/mol; **Rf:** 0.20 (DCM/MeOH 99:1); **MP (°C)**: 182- 184; **IR (ATR) ν (cm^{-1})**: 2944, 1703, 1358; **^{1}H-NMR (400 MHz, $CDCl_3$) δ (ppm)**: 7.54 (d, *J* = 8.2 Hz, 2H, H**14**), 7.46 – 7.38 (m, 6H, **Ar**-H), 7.19 (d, *J* = 8.2 Hz, 2H, H**15**), 7.10 – 7.05 (m, 4H, **Ar**-H), 3.70 (t, *J* = 3.0 Hz, 1H, H**1**), 3.17 (m, 6H, H**2**; H**11**; H**12**), 2.84 (d, *J* = 8.4 Hz, 2H, H**5**), 2.41 (s, 3H, H**16**), 2.44 – 2.39 (m, 2H, H**10**/H**13**), 2.38 – 2.33 (m, 2H, H**10**/H**13**), 1.96 (s, 3H, H**9**); **^{13}C-NMR (101 MHz, $CDCl_3$) δ (ppm):** 175.5 (C**3**/C**4**), 174.8 (C**3**/C**4**), 143.6, 138.7, 136.3, 135.3, 131.4, 129.9 (C**15**), 129.5, 129.0, 127.2 (C**14**), 126.1, 48.9 (C**5**), 47.4 (C**12**/C**11**), 47.3 (C**12**/C**11**), 44.5 (C**6**), 44.0 (C**2**), 41.9 (C**1**), 35.0 (C**10**/C**13**), 29.7 (C**10**/C**13**), 21.7 (C**16**), 19.1 (C**9**); **HRMS (ESI) m/z:** [M + Na]$^+$ Calculated for $C_{36}H_{33}N_3O_6SNa$ 658.1982, Found: 658.1993

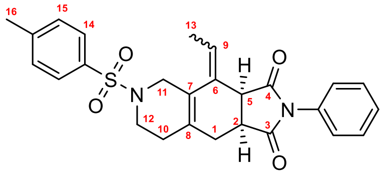

5

Compound 5 was obtained from **allenyne 1k** (26.1 mg, 0.09 mmol, 1 equiv.) and **maleimide 2a** (77.8 mg, 0.45 mmol, 5 equiv.) using a mixture of $[Rh(cod)Cl]_2$ (2.2 mg, 0,0051 mmol, 0.05 equiv.) and DPEphos (7.5 mg, 0.0139 mmol, 0.15 equiv.) following the general procedure in an anhydrous mixture of PhCl/EtOH/TFA (5:1:1.7x10^{-6}) (5 mL). The resulting mixture was heated at 130 °C and stirred for 16 h (TLC monitoring). Purification by column chromatography on silica gel using mixtures of Hexanes/EtOAc (100:0 to 70:30 v/v) provided a mixture of *E/Z* isomers of **5** (29.1 mg, 70% yield, *E/Z* = 85:15) as a colourless solid.

MW ($C_{26}H_{26}N_2O_4S$): 462.56 g/mol; **Rf:** 0.17 (Hexanes/EtOAc 7:3); **MP (ºC)**: 85 – 89; **IR (ATR) ν (cm⁻¹)**: 2933, 1689, 1344; With the presence of traces of Z isomer **^{1}H-NMR (400 MHz, $CDCl_3$) δ (ppm)**: 7.64 (d, *J* = 8.1 Hz, 2H, H**14**), 7.46 – 7.32 (m, 3H, **Ar**-H), 7.29 (d, *J* = 8.1 Hz, 2H, H**15**), 7.13 – 7.05 (m, 2H, **Ar**-H), 5.72 (q, *J* = 7.2 Hz, 1H, H**9**), 4.00 (dq, *J* = 15.9, 2.7 Hz, 1H, H**11**), 3.67 (d, *J* = 8.7 Hz, 1H, H**5**), 3.46 (dd, *J* = 15.9, 2.7 Hz, 1H, H**11**), 3.33 (ddd, *J* = 8.7, 6.5, 2.0 Hz, 1H, H**2**), 3.22 – 3.07 (m, 2H, H**12**), 2.55 (dd, *J* = 14.9, 2.0, 1H, H**1**), 2.42 (s, 3H, H**16**), 2.39 – 2.21 (m, 3H, H**1**; H**10**), 1.76 (d, *J* = 7.2 Hz, 3H, H**13**) (residual EtOAc solvent peaks are observed); **^{13}C-NMR (101 MHz, $CDCl_3$) δ (ppm):** 178.4 (C**3**/C**4**), 176.7 (C**3**/C**4**), 143.8, 133.4, 133.2, 132.1, 129.9 (C**15**), 129.3, 128.9, 128.8, 127.8 (C**14**), 127.6, 127.5 (C**9**), 126.5, 50.4 (C**5**), 46.7 (C**11**), 43.1 (C**12**), 40.6 (C**2**), 30.8 (C**10**), 29.5 (C**1**), 21.7 (C**16**), 15.7 (C**13**); **HRMS (ESI) m/z:** $[M + H]^+$ Calculated for $C_{26}H_{27}N_2O_4S$ 463.1686, Found: 463.1694.

S3.3 Mixed Experiments

In order to evaluate the reactivity of different maleimides, we set up the reaction using a mixture of two maleimides.

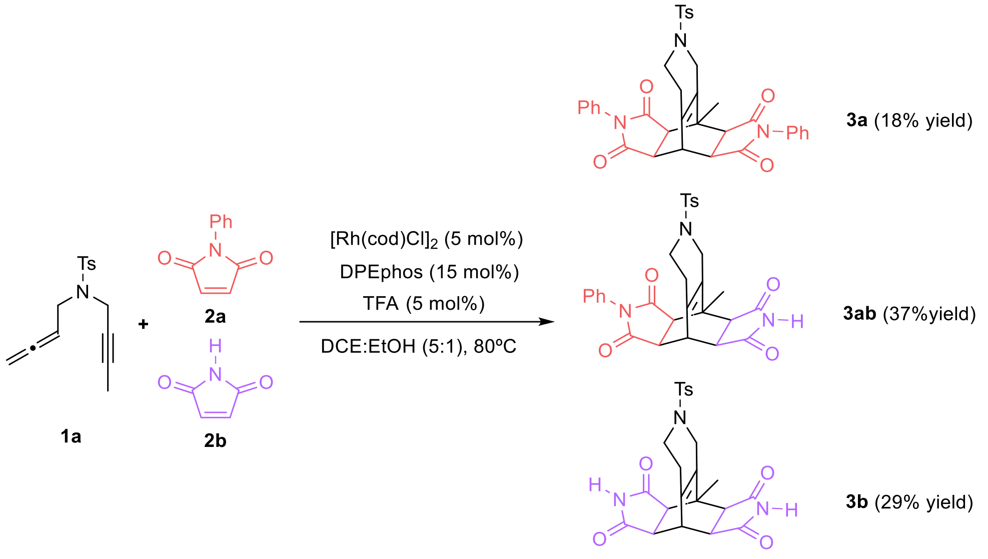

Compound 3ab was obtained from **allenyne 1a** (25.3 mg, 0.09 mmol, 1 equiv.), **maleimide 2a** (39.4 mg, 0.23 mmol, 2.5 equiv.) and **maleimide 2b** (22.0 mg, 0.24 mmol, 2.5 equiv.) using a mixture of $[Rh(cod)Cl]_2$ (2.2 mg, 0,0045 mmol, 0.05 equiv.) and DPEphos (7.4 mg, 0.0137 mmol, 0.15 equiv.) following the general procedure and stirred for 16 h (TLC monitoring). Purification by column chromatography on silica gel using mixtures of DCM:MeOH (98:2 v/v) provided **compound 3a** (10.3 mg, 18% yield), **compound 3b** (12.6mg, 29% yield) and **compound 3ab** (18.7 mg, 37% yield) as a colourless solid.

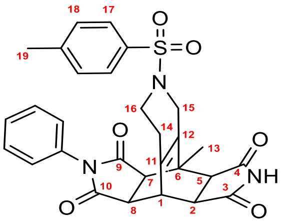

MW ($C_{29}H_{27}N_3O_6S$): 545.61 g/mol; **Rf:** 0.24 (DCM:MeOH 98:2); **MP (ºC)**: >280 (dec); **IR (ATR) ν (cm^{-1})**: 1710, 1374, 1189; **^{1}H-NMR (400 MHz, DMSO) δ (ppm):** 11.22 (s, 1H, **NH**), 7.66 (d, *J* = 8.2 Hz, 2H, H**17**), 7.48 (d, *J* = 8.2 Hz, 2H, H**18**), 7.44 – 7.31 (m, 3H, **Ar-**H), 6.90 – 6.83 (m, 2H, **Ar-**H), 3.36 (dd, *J* = 7.9, 2.7 Hz, 1H, H**2**/H**8**), 3.25 (m, 2H, H**15**), 3.24 – 3.17 (m, 2H, H**1; H2**/H**8**), 2.99 (d, *J* = 7.9 Hz, 1H, H**5**/H**7**), 2.95 – 2.87 (m, 2H, H**16**; H**5**/H**7**), 2.74 (m, 1H, H**16**), 2.43 (s, 3H, H**19**), 2.01 (m, 2H, H**14**), 1.69 (s, 3H, H**13**) (residual DCM solvent peak is observed); **^{13}C NMR (101 MHz, DMSO) δ:** 178.4 (C**3**/C**4**/C**9**/C**10**), 178.0 (C**3**/C**4**/C**9**/C**10**), 176.3 (C**3**/C**4**/C**9**/C**10**), 175.5 (C**3**/C**4**/C**9**/C**10**), 143.9, 131.9, 131.7, 131.6, 130.2, 130.0, 128.9, 128.5, 127.5, 126.7, 54.9, 49.4 (C**5**/C**7**), 48.2 (C**5**/C**7**), 44.3 (C**15**), 43.9 (C**2**/C**8**), 43.6 (C**2**/C**8**), 42.7 (C**16**), 41.4 (C**6**), 38.1 (C**1**), 28.8 (C**14**), 21.1 (C**19**), 15.5 (C**13**); **HRMS (ESI) m/z:** [M + Na]$^+$ Calculated for $C_{29}H_{27}N_3O_6SNa$ 568.1513, Found: 568.1518

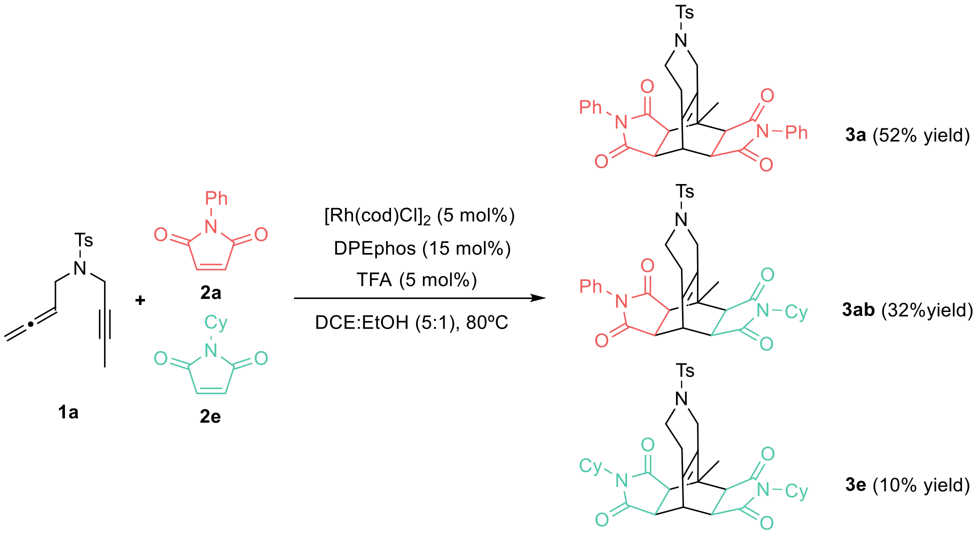

Compound 3ae was obtained from **allenyne 1a** (25.0 mg, 0.09 mmol, 1 equiv.), **maleimide 2a** (39.1 mg, 0.23 mmol, 2.5 equiv.) and **maleimide 2e** (40.5 mg, 0.23 mmol, 2.5 equiv) using a mixture of $[Rh(cod)Cl]_2$ (2.5 mg, 0,0051 mmol, 0.05 equiv.) and DPEphos (7.3 mg, 0.0135 mmol, 0.15 equiv.) following the general procedure and stirred for 16 h (TLC monitoring). Purification by column chromatography on silica gel using mixtures of DCM:MeOH (98:2 v/v) provided **compound 3a** (30.2 mg, 52% yield), **compound 3e** (5.6 mg, 10% yield) and **compound 3ae** (18.3 mg, 32% yield) as a colourless solid.

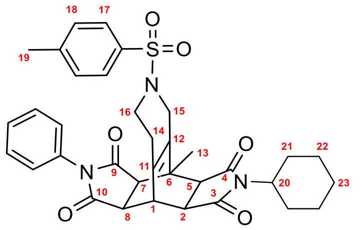

3ae

MW ($C_{35}H_{37}N_3O_6S$): 627.76 g/mol; **Rf:** 0.22 (Hexanes:EtOAc 1:1); **MP (ºC)**: 160 – 165; **IR (ATR) ν (cm^{-1})**: 1707, 1373, 1180; **^{1}H-NMR (400 MHz, CDCl$_3$) δ (ppm)**: 7.60 (d, *J* = 8.2 Hz, 2H, H**17**), 7.43 – 7.34 (m, 3H, **Ar**-H), 7.31 (d, *J* = 8.2 Hz, 2H, H**18**), 7.01 – 6.94 (m, 2H, **Ar**-H), 3.80 (tt, *J* = 12.4, 3.9 Hz, 1H, H**20**), 3.63 (t, *J* = 3.1 Hz, 1H, H**1**), 3.48 – 3.38 (m, 2H, H**15**), 3.15 (dd, *J* = 8.1, 3.1 Hz, 1H, H**2**/H**8**), 2.97 (dd, *J* = 8.1, 3.2 Hz, 1H, H**2**/H**8**), 2.92 (t, *J* = 5.8 Hz, 2H, H**16**), 2.80 (d, *J* = 8.1 Hz, 1H, H**5**/H**7**), 2.63 (d, *J* = 8.1 Hz, 1H, H**5**/H**7**), 2.44 (s, 3H, H**19**), 2.25 – 2.17 (br s, 2H, H**14**), 2.08 – 1.89 (m, 2H, H**21**), 1.87 (s, 3H, H**13**), 1.80 – 1.69 (m, 2H, H**22**), 1.67 – 1.68 (m, 2H, H**23**), 1.37 – 1.26 (m, 2H, H**21**), 1.26 – 1.06 (m, 2H, H**22**); **^{13}C-NMR (101 MHz, CDCl$_3$) δ (ppm):** 176.3 (C**3**/C**4**/C**9**/C**10**), 175.4 (C**3**/C**4**/C**9**/C**10**), 174.6 (C**3**/C**4**/C**9**/C**10**), 143.9, 132.5, 132.4, 131.4, 131.1, 129.9 (C**18**), 129.4, 129.1, 127.8 (C**17**), 126.5, 52.0 (C**20**), 49.2 (C**5**/C**7**), 48.6 (C**5**/C**7**), 44.5 (C**15**), 43.8 (C**2**/C**8**), 43.3 (C**2**/C**8**), 42.8 (C**16**), 42.5 (C**6**), 38.4 (C**1**), 29.2 (C**14**), 28.7 (C**21**), 25.9 (C**22**), 25.0 (C**23**), 21.7 (C**19**), 16.3 (C**13**); **HRMS (ESI) m/z:** [M + Na]$^+$ Calculated for $C_{35}H_{37}N_3O_6SNa$ 650.2295, Found: 650.2295.

S3.4 Unsuccessful 1,6-allenynes and alkenes tested

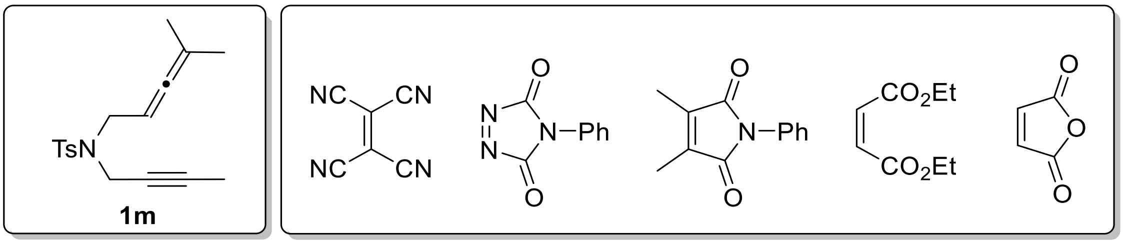

S4. Mechanistic Studies

S4.1 Mechanistic experiments

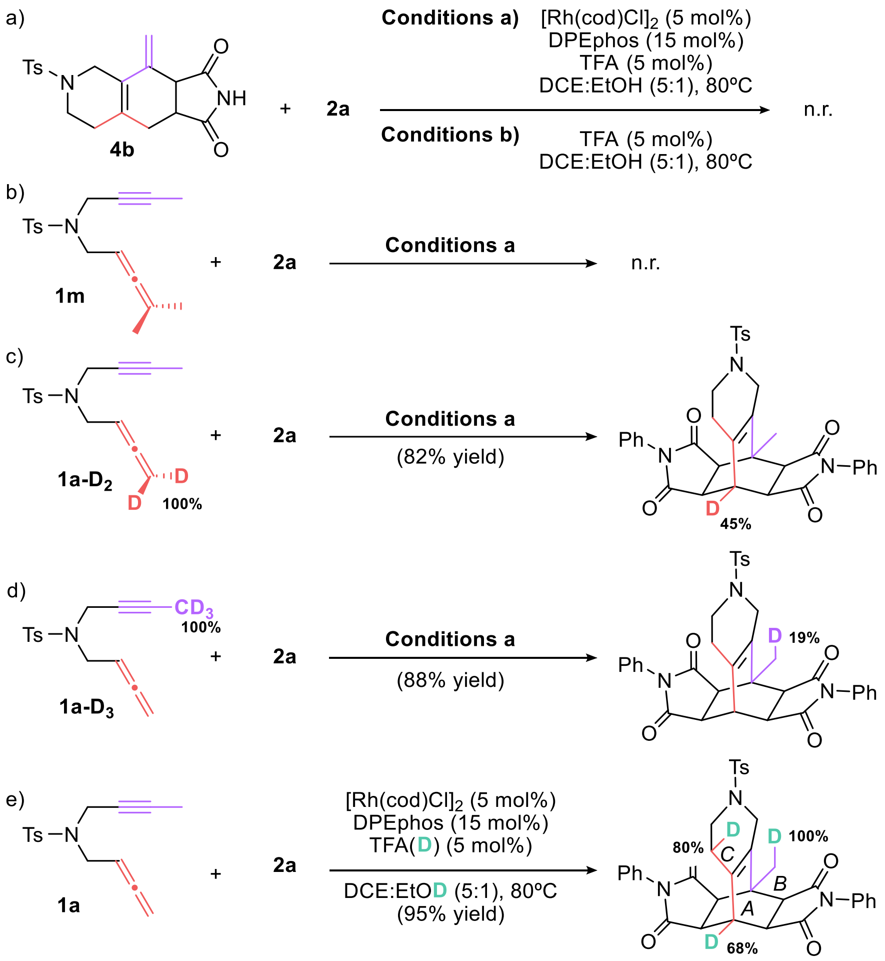

S4.2 Synthesis of Monoadduct 4b

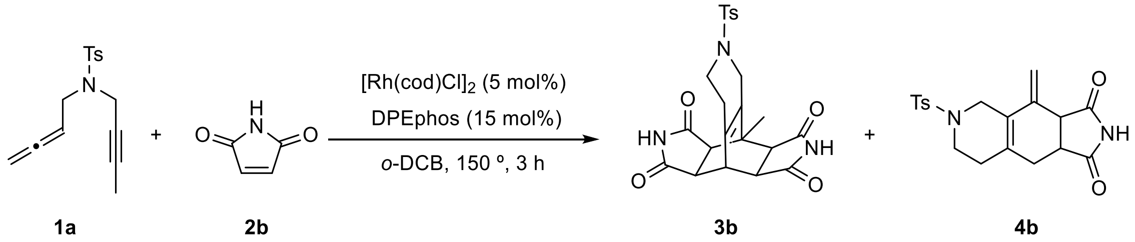

In a 25 mL capped vial, a mixture of $[Rh(cod)Cl]_2$ (4.3 mg, 0,009 mmol, 0.05 equiv.) and DPEphos (14.8 mg, 0.018 mmol, 0.10 equiv.) was purged with nitrogen, dissolved in *o*-DCB (4 mL) and transferred via syringe into a solution of **allenyne 1a** (50.2 mg, 0.18 mmol, 1.0 equiv.) and **maleimide 2b** (44 mg, 0.45 mmol, 2.5 equiv.) in *o*-DCB (6 mL) preheated at 150ºC and under inert atmosphere. The resulting mixture was stirred for 3 h at the same temperature (TLC monitoring). The solvent was then removed under reduced pressure, and the resulting crude was purified by column chromatography on silica gel using mixtures of

DCM:MeOH (100:0 to 98:2 v/v) to afford **compound 3b** (44.3 mg, 52% yield) as a colourless solid and **compound 4b** (12.2 mg, 18% yield) as a colourless solid.

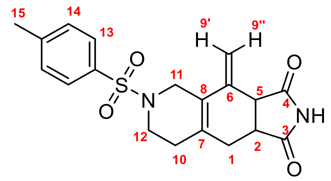

4b

MW ($C_{19}H_{20}N_2O_4S$)**:** 372.44 g/mol; **Rf:** 0.4 (DCM/MeOH 98:2); **MP (ºC)**: >300 (dec); **IR (ATR) ν (cm^{-1})**: 1705, 1323, 1155; **^{1}H-NMR (400 MHz, $CDCl_3$) δ (ppm)**: 8.21 (br s, 1H, N**H**), 7.66 (d, *J* = 8.1 Hz, 2H, H**13**), 7.32 (d, *J* = 8.1 Hz, 2H, H**14**), 5.32 (s, 1H, H**9''**), 5.14 (s, 1H, H**9'**), 3.85 (dd, *J* = 15.3, 2.8 Hz, 1H, H**11**), 3.69 (dt, *J* = 8.9, 1.3 Hz, 1H, H**5**), 3.48 (dt, *J* = 15.3, 2.2 Hz, 1H, H**11**), 3.26 (dt, *J* = 11.7, 5.8 Hz, 1H, H**12**), 3.19 (ddd, *J* = 8.8, 7.6, 3.1 Hz, 1H, H**2**), 3.06 (ddd, *J* = 11.7, 5.8 Hz, 1H, H**12**), 2.43 (s, 3H, H**15**), 2.54 – 2.12 (m, 4H, H**10**; H**1**); **^{13}C-NMR (101 MHz, $CDCl_3$) δ (ppm):** 178.8 (C**3**/C**4**), 176.9 (C**3**/C**4**), 144.0, 133.3, 133.2, 131.3, 129.9 (C**14**), 127.8 (C**13**), 125.2, 113.1 (C**9**), 46.5 (C**5**), 44.9 (C**11**), 42.5 (C**12**), 39.3 (C**2**), 30.7 (C**1**), 27.4 (C**10**), 21.7 (C**15**); **HRMS (ESI) m/z:** [M + Na]$^+$ Calculated for $C_{19}H_{20}N_2O_4SNa$ 395.1036, Found: 395.1046.

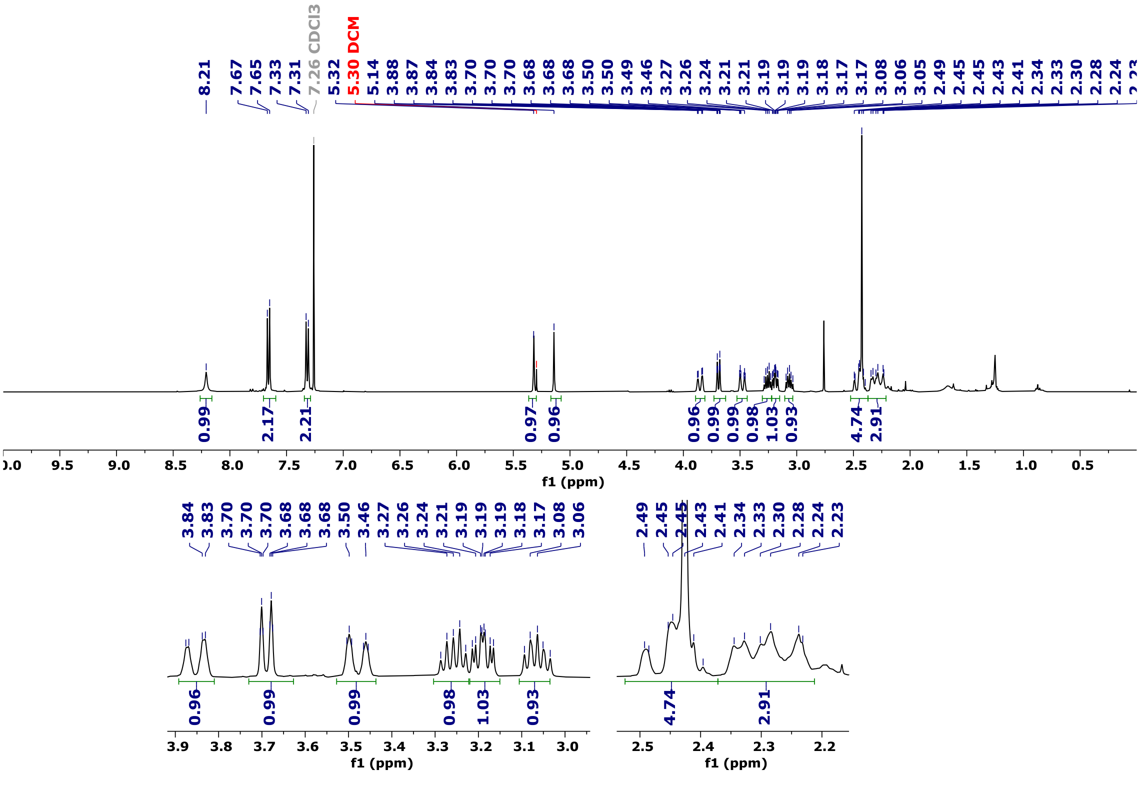

Figure S1. *^{1}H NMR (400 MHz, $CDCl_3$) of the reaction product* ***4b***.

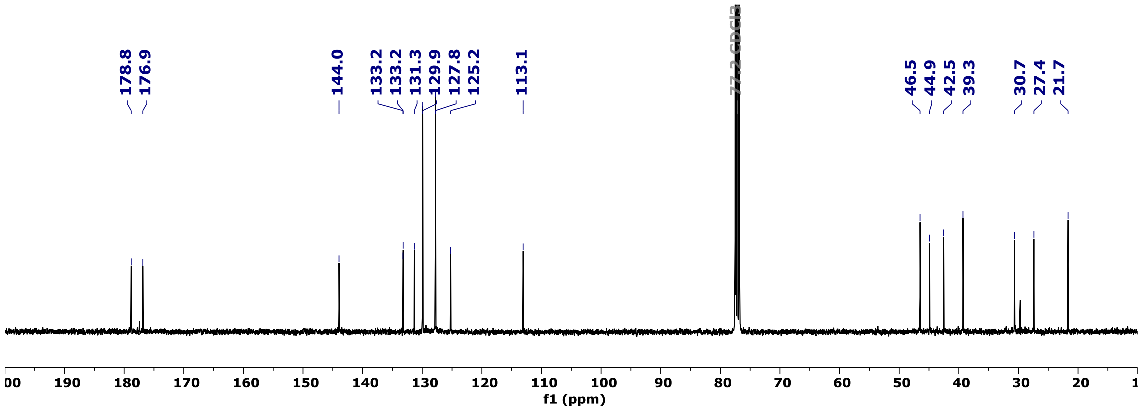

Figure S2. [13]*C NMR (400 MHz, CDCl*$_3$*) of the reaction product **4b***.

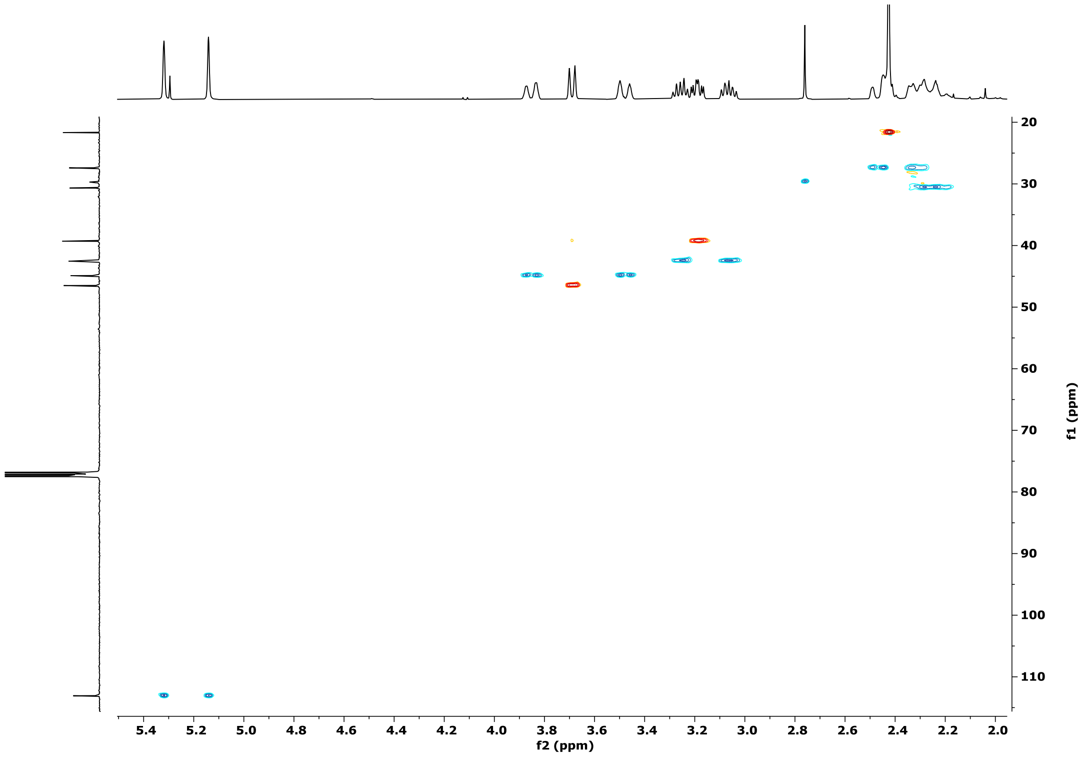

Figure S3. *HSQC experiment of reaction product **4b***.

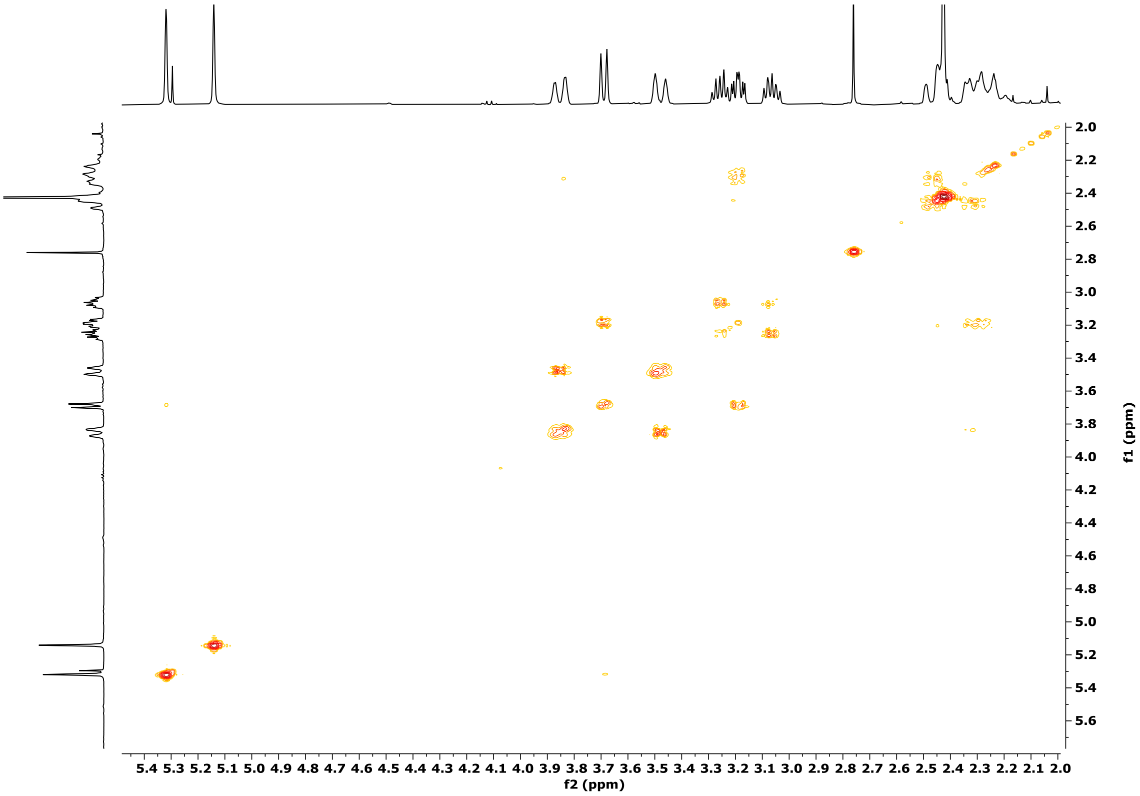

Figure S4. *COSY experiment of reaction product **4b**.*

S4.3 Reaction between 4b and 2a

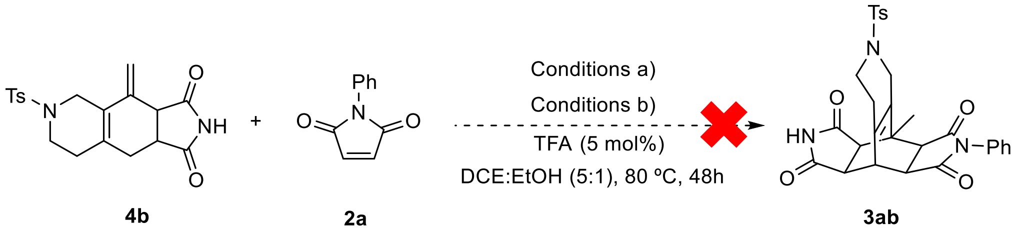

Conditions a)

In a 10 mL capped vial, a mixture of $[Rh(cod)Cl]_2$ (0.8 mg, 0,0016 mmol, 0.06 equiv.) and DPEphos (2.4 mg, 0.0045 mmol, 0.16 equiv.) was purged with nitrogen, dissolved in an anhydrous mixture of DCE/EtOH/TFA (5:1:1.7x10^{-6}) (1 mL) and transferred via syringe into a solution of **compound 4b** (10.2 mg, 0.027 mmol, 1.0 equiv.) and **maleimide 2a** (23.7 mg, 0.140 mmol, 5 equiv.) in an anhydrous mixture of DCE/EtOH/TFA (5:1:1.7x10^{-6}) (2 mL) preheated at 80 ºC and under inert atmosphere. The resulting mixture was stirred for 48 h at the same temperature. The solvent was removed under reduced pressure and ^{1}H-NMR

analysis of the crude shows that the reaction does not work and only starting materials were recovered.

Conditions b)

In a 10 mL capped vial, **compound 4b** (11.3 mg, 0.030 mmol, 1.0 equiv.) and **maleimide 2a** (25.0 mg, 0.144 mmol, 5 equiv.) was solved in an anhydrous mixture of DCE/EtOH/TFA (5:1:1.7x10^{-6}) (2 mL) preheated at 80 ºC and under inert atmosphere. The resulting mixture was stirred for 48 h at the same temperature. The solvent was removed under reduced pressure and ^{1}H-NMR analysis of the crude shows that the reaction does not work and only starting materials were recovered.

S4.4 Deuterium labelling experiments

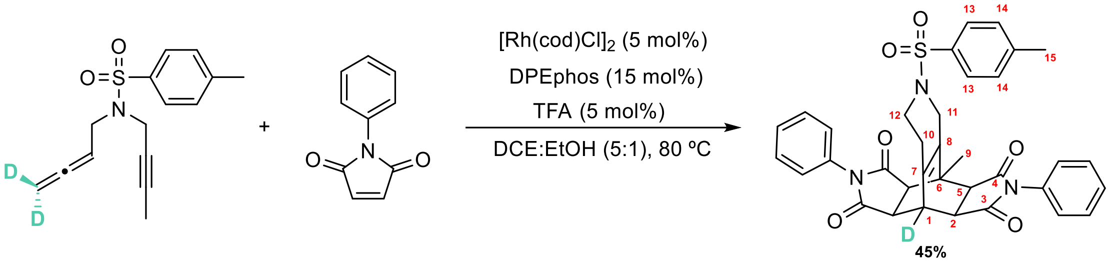

1a-D$_2$ (>99 D) **2a** **3a(D1)**

Compound 3a(D1) was obtained from **allenyne 1a-D$_2$** (20.0 mg, 0.07 mmol, 1.0 equiv.) and **maleimide 2a** (60.6 mg, 0.35 mmol, 5 equiv.) using a mixture of [Rh(cod)Cl]$_2$ (1.8 mg, 0,0036 mmol, 0.05 equiv.) and DPEphos (5.7 mg, 0.0107 mmol, 0.15 equiv.) following the general procedure. The resulting mixture was heated at 80 ºC and stirred for 16 h (TLC monitoring). The resulting crude was purified by column chromatography on silica gel using mixtures of DCM:MeOH (100:0 to 99:1 v/v) to afford **compound 3a(D1)** (35.9 mg, 82% yield) as a colourless solid.

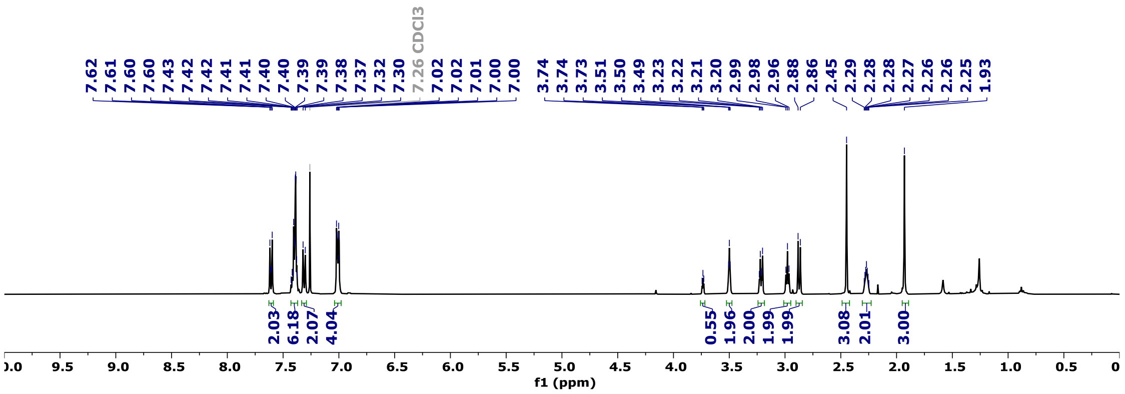

Figure S5. *^{1}H NMR (400 MHz, CDCl$_3$) of the reaction product* ***3a(D1).***

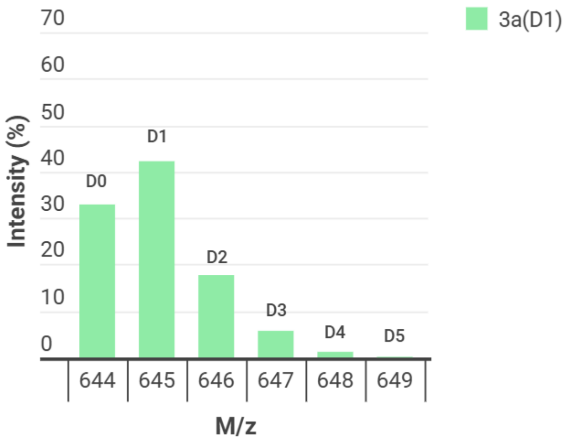

***Graph 1.** Experimental HRMS_ESI of deuterated adduct **3a(D1)**.*

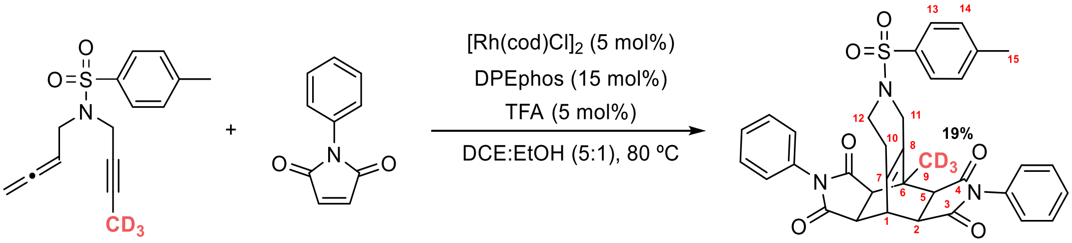

1a-D$_3$ (>99% D) **2a** **3a(D2)**

Compound 3a(D2) was obtained from **allenyne 1a-D$_3$** (19.5 mg, 0.07 mmol, 1.0 equiv.) and **maleimide 2a** (60.4 mg, 0.35 mmol, 5 equiv.) using a mixture of [Rh(cod)Cl]$_2$ (1.7 mg, 0,0035 mmol, 0.05 equiv.) and DPEphos (5.7 mg, 0.0106 mmol, 0.15 equiv.) following the general procedure. The resulting mixture was heated at 80 ºC and stirred for 16 h (TLC monitoring). The resulting crude was purified by column chromatography on silica gel using mixtures of DCM:MeOH (100:0 to 99:1 v/v) to afford **compound 3a(D2)** (38.5 mg, 88% yield) as a colourless solid.

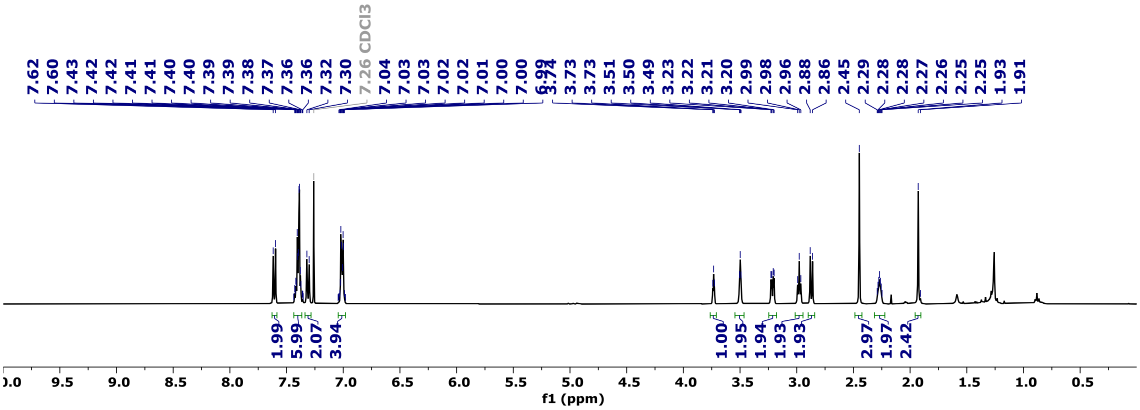

***Figure S6.** ^{1}H NMR (400 MHz, CDCl$_3$) of the reaction product **3a(D2)**.*

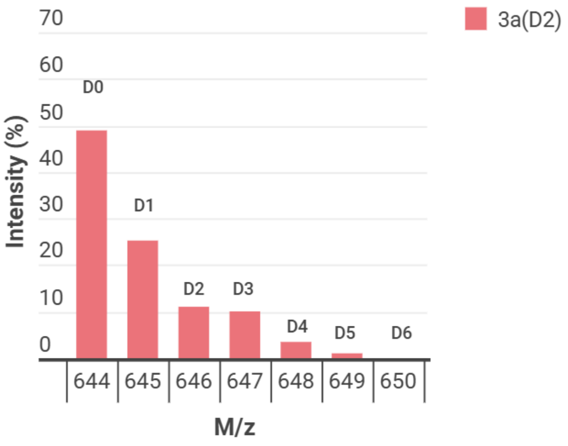

Graph 2. *Experimental HRMS_ESI of deuterated adduct* ***3a(D2)***.

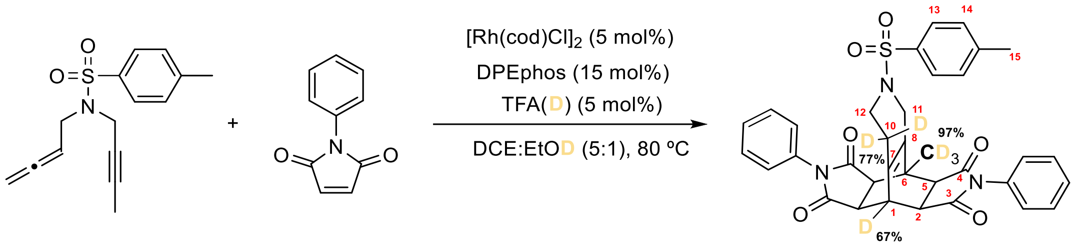

1a **2a** **3a(D3)**

Compound 3a(D3) was obtained from **allenyne 1a** (25.0 mg, 0.09 mmol, 1.0 equiv.) and **maleimide 2a** (78.2 mg, 0.45 mmol, 5 equiv.) using a mixture of $[Rh(cod)Cl]_2$ (2.1 mg, 0,0043 mmol, 0.05 equiv.) and DPEphos (7.0 mg, 0.0130 mmol, 0.15 equiv.) following the general procedure but using deuterated TFA and EtOD. The resulting mixture was heated at 80 ºC and stirred for 16 h (TLC monitoring). The resulting crude was purified by column chromatography on silica gel using mixtures of DCM:MeOH (100:0 to 99:1 v/v) to afford **compound 3a(D3)** (53.2 mg, 95% yield) as a colourless solid.

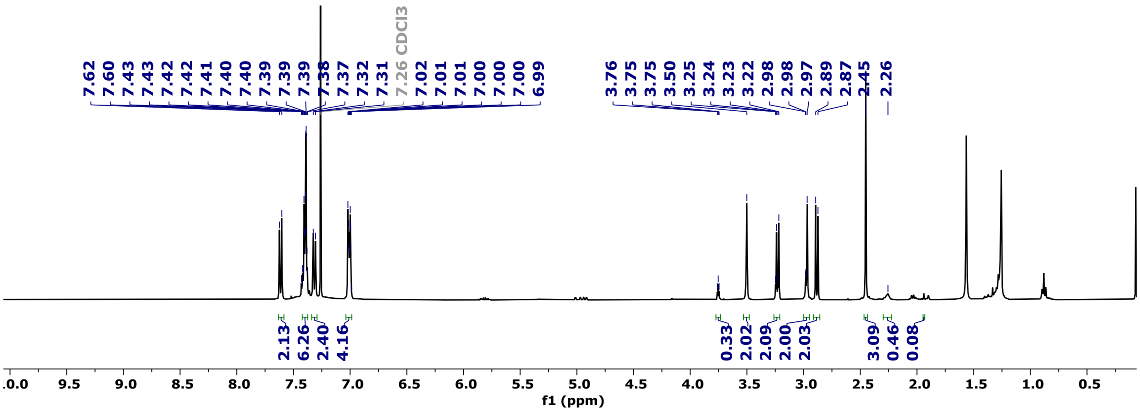

Figure S7. *^{1}H NMR (400 MHz, $CDCl_3$) of the reaction product* ***3a(D3)***.

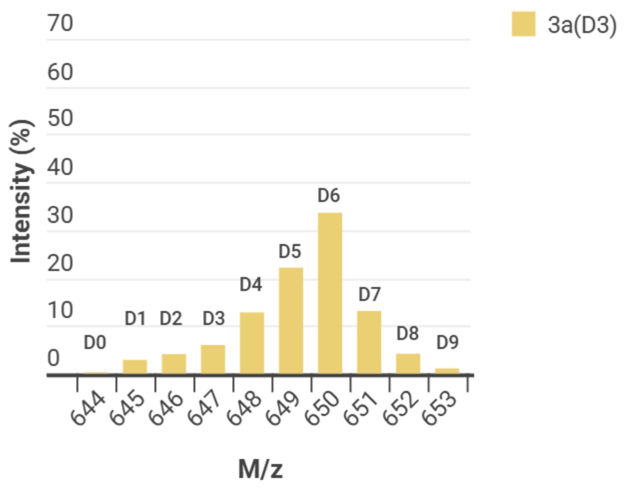

Graph 3. *Experimental HRMS_ESI of deuterated adduct **3a(D3)**.*

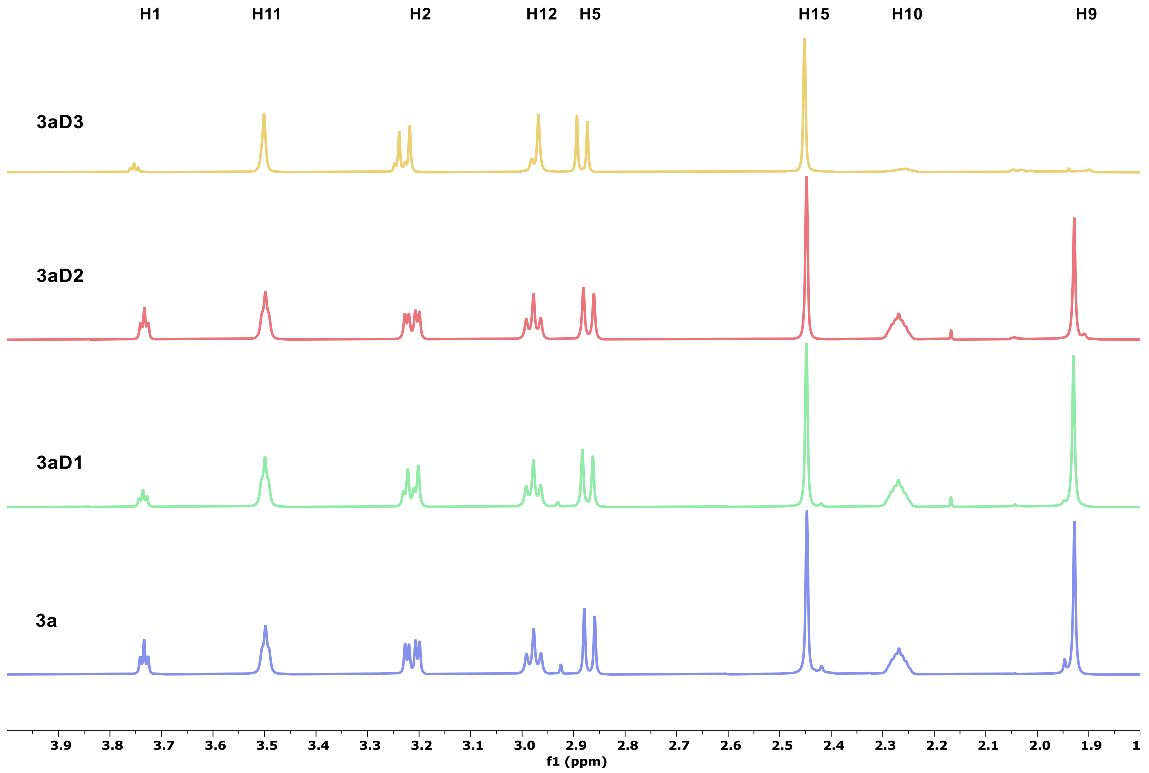

Figure S8. *^{1}H NMR (400 MHz, $CDCl_3$) of the reaction product **3a** and the deuterated experiments.*

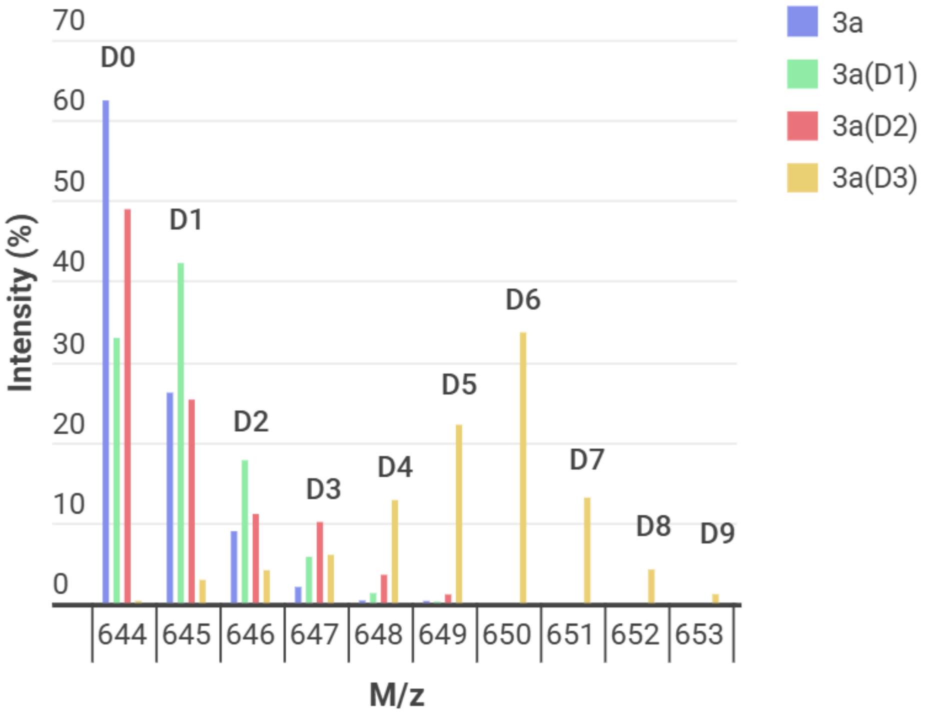

Graph 4. *Experimental HRMS_ESI of all deuterated adduct*

^{1}H and ^{13}C NMR spectra

Compound 2a

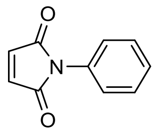

^{1}H NMR (400 MHz, $CDCl_3$)

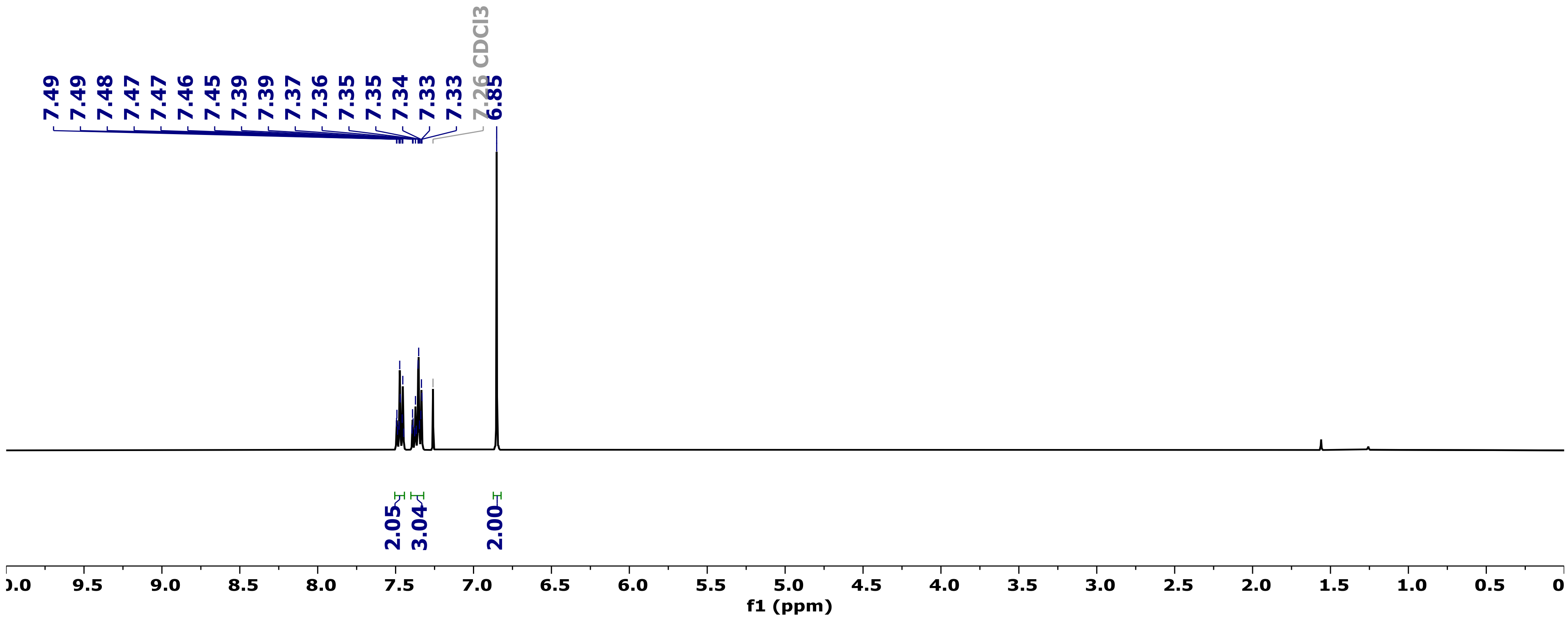

Compound 2e

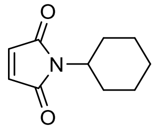

^{1}H NMR (400 MHz, $CDCl_3$)

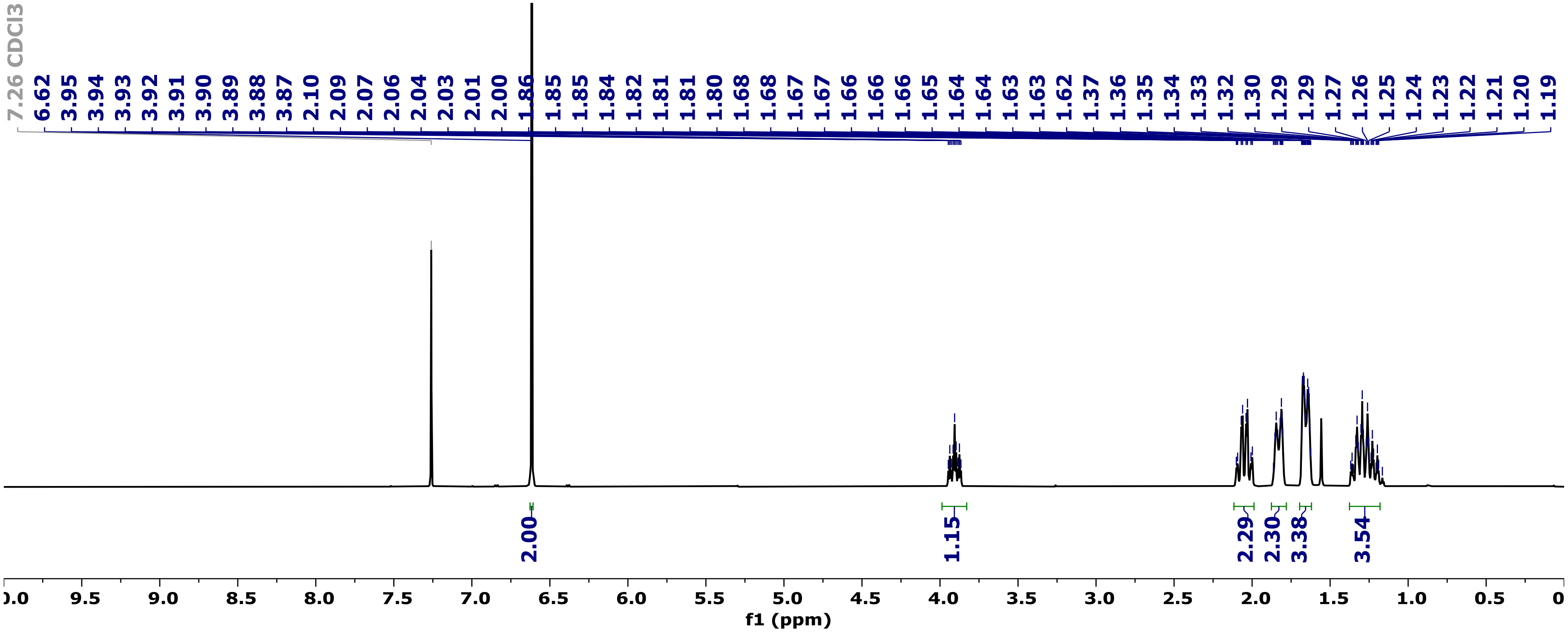

Compound 2f

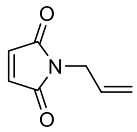

^{1}H NMR (400 MHz, $CDCl_3$)

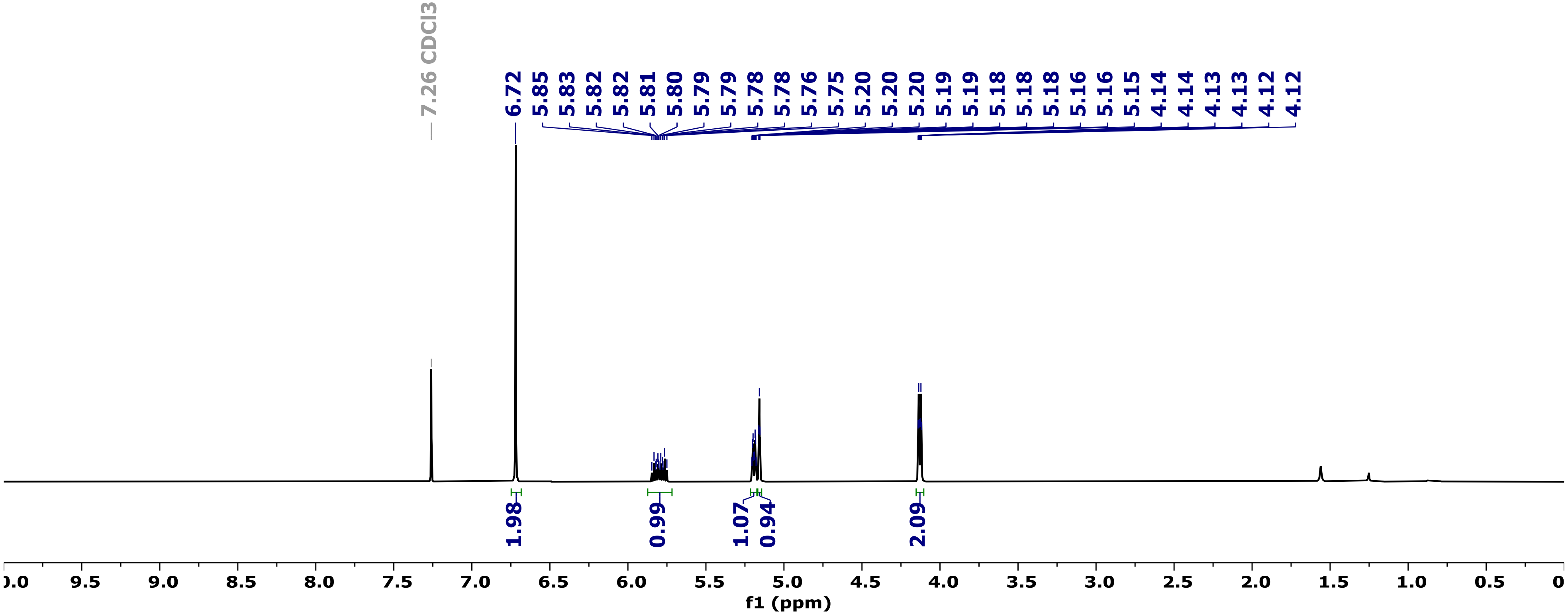

Compound 2g

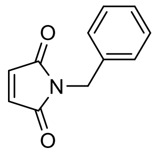

^{1}H NMR (400 MHz, $CDCl_3$)

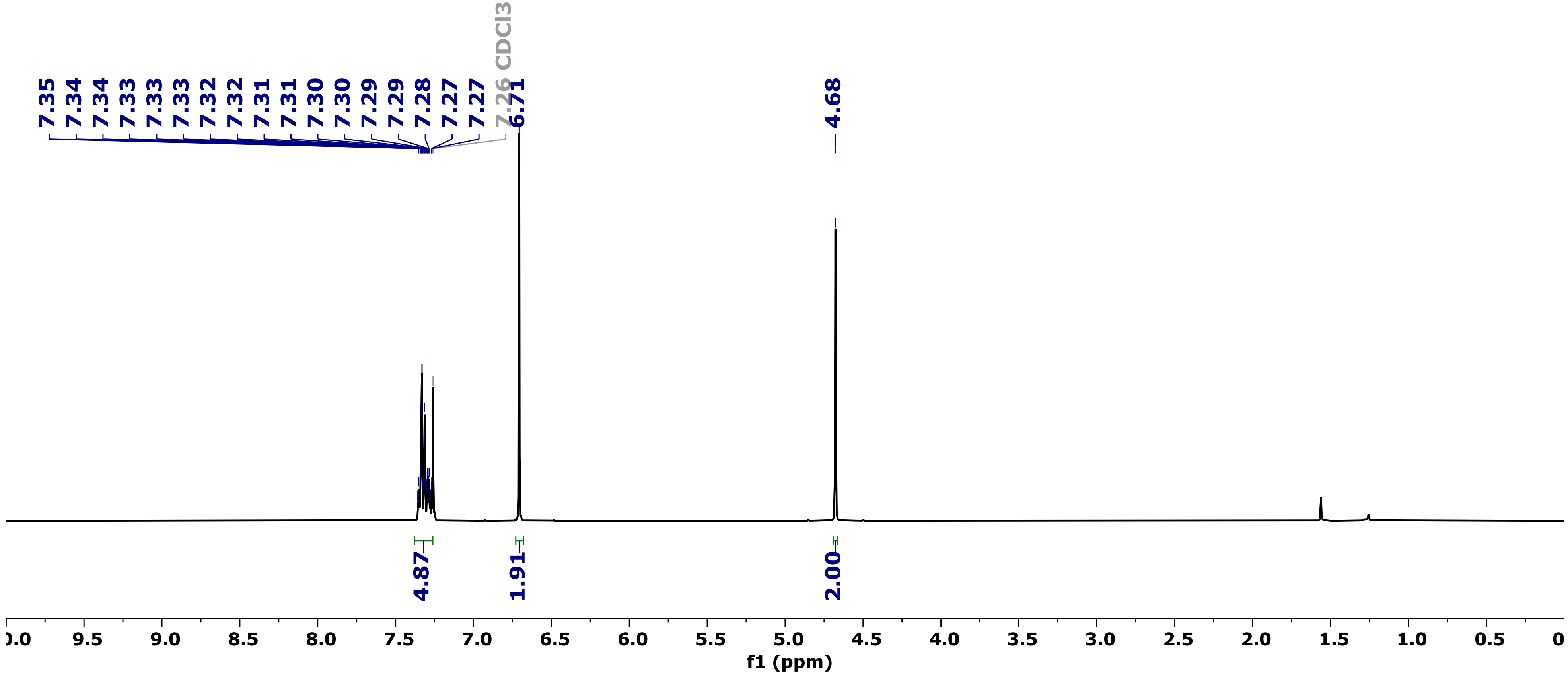

Compound 2h

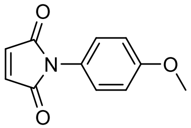

^{1}H NMR (400 MHz, $CDCl_3$)

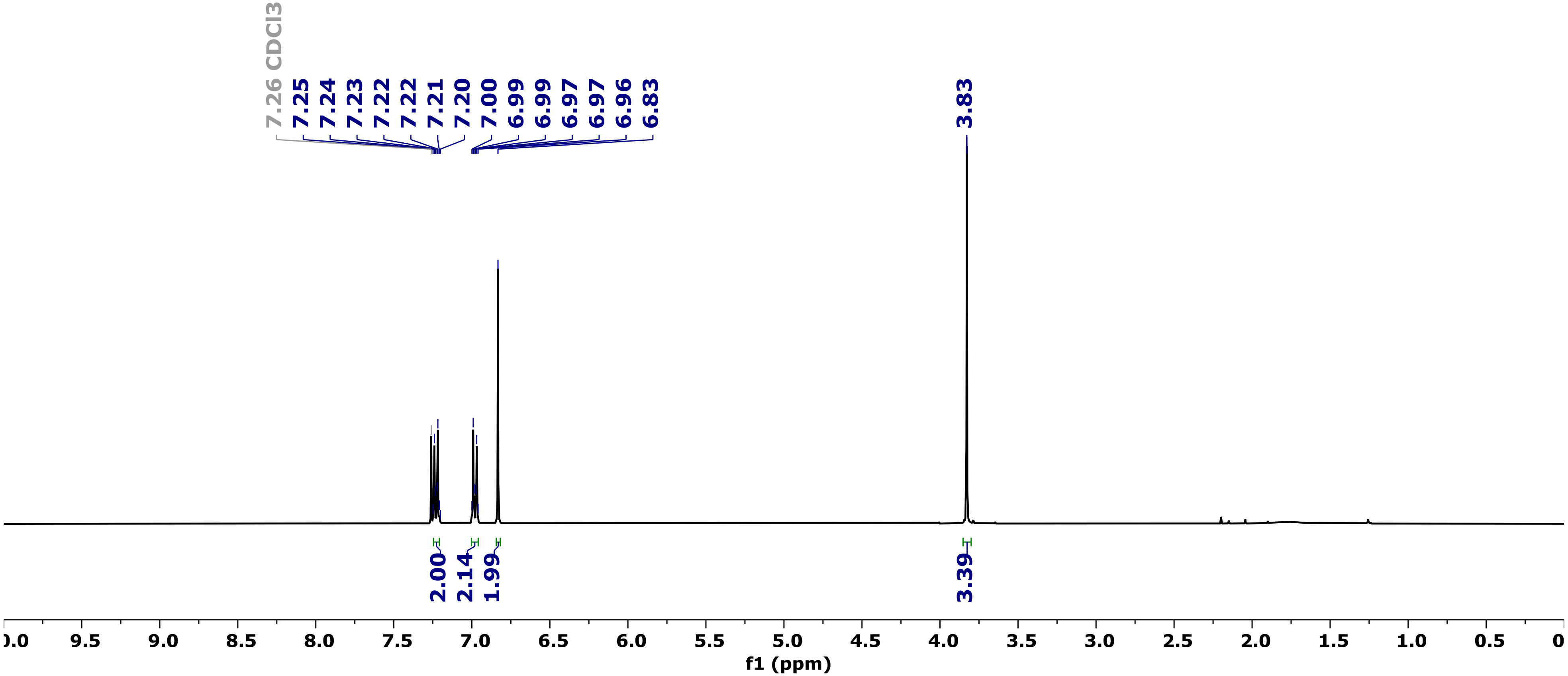

Compound 2i

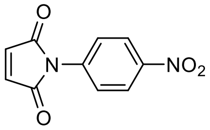

^{1}H NMR (400 MHz, Acetone-d$_6$)

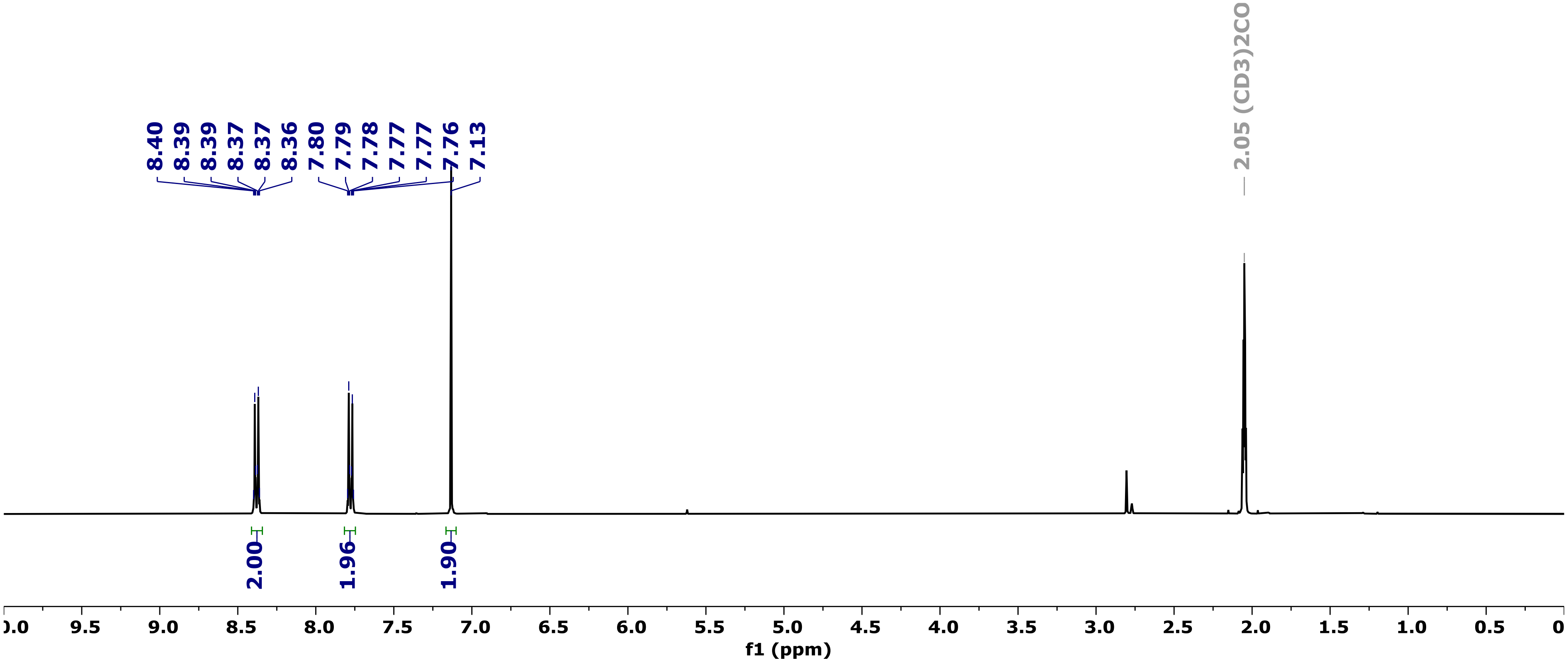

Compound 2j

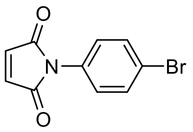

^{1}H NMR (400 MHz, $CDCl_3$)

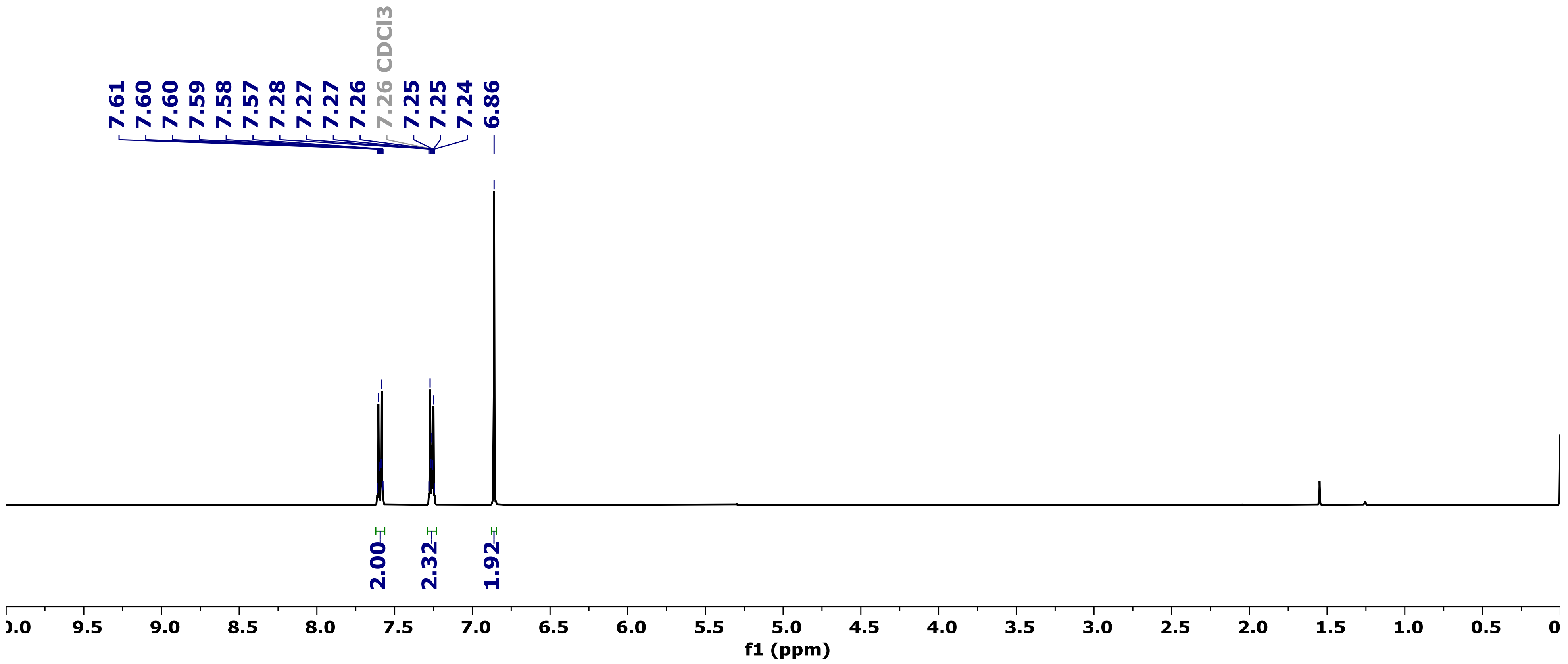

Compound 2k

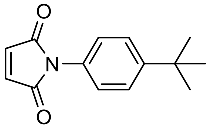

^{1}H NMR (400 MHz, Acetone-d_6)

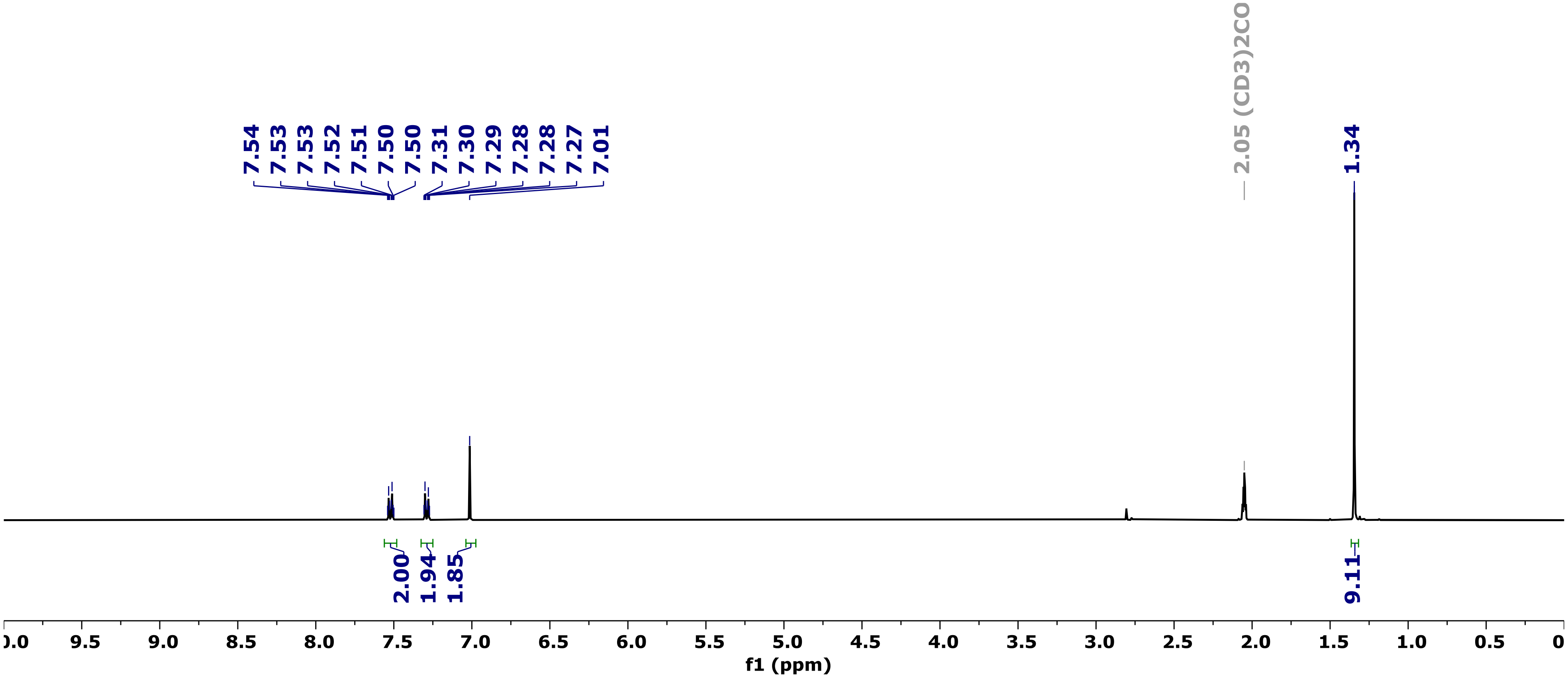

Compound 2l

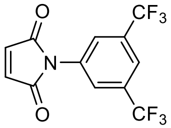

^{1}H NMR (400 MHz, Acetone-d$_6$)

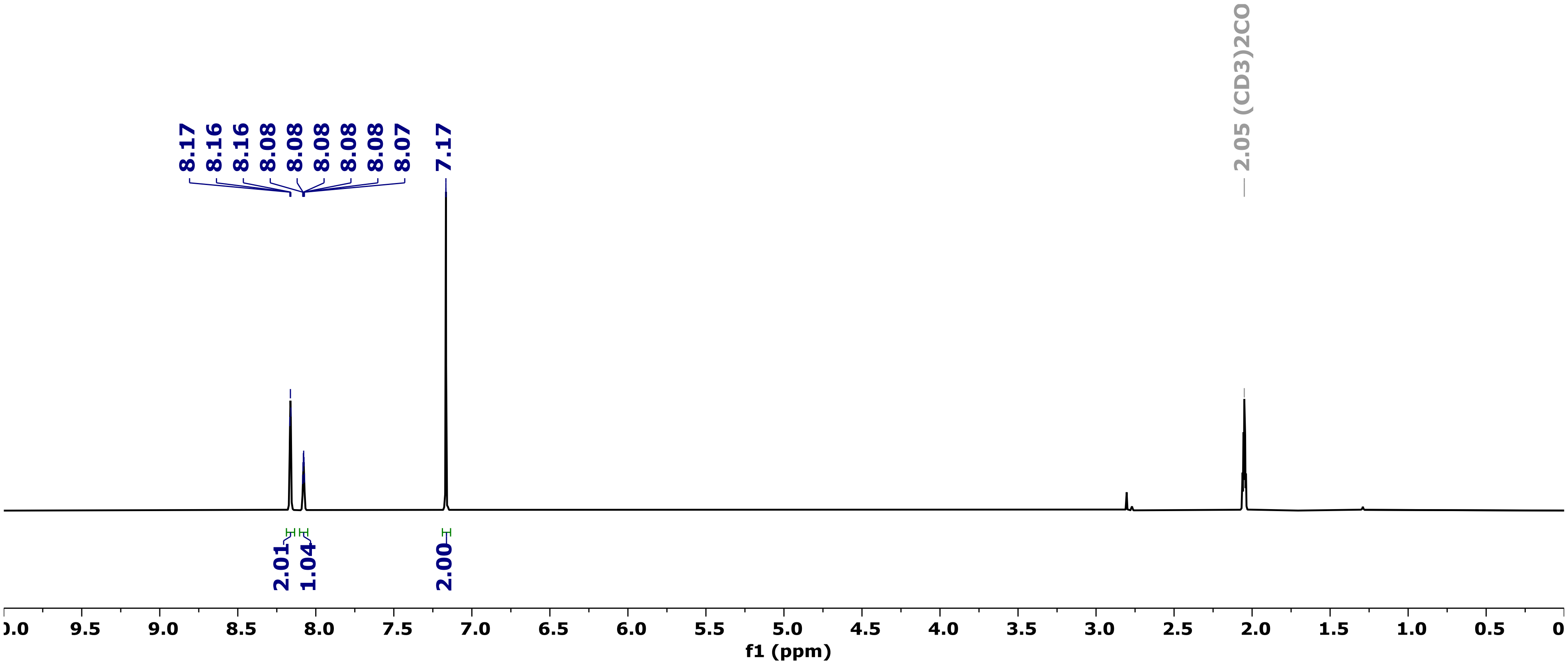

Compound 3a

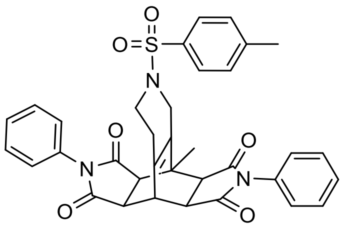

^{1}H NMR (400 MHz, $CDCl_3$)

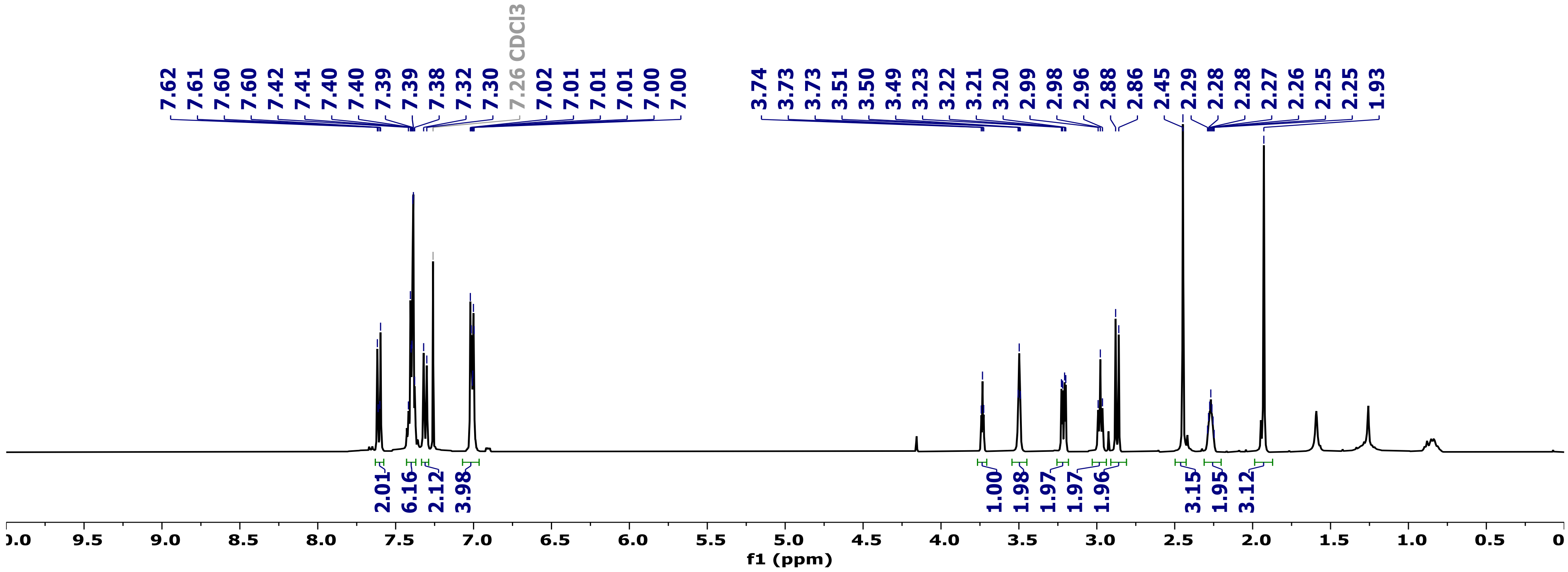

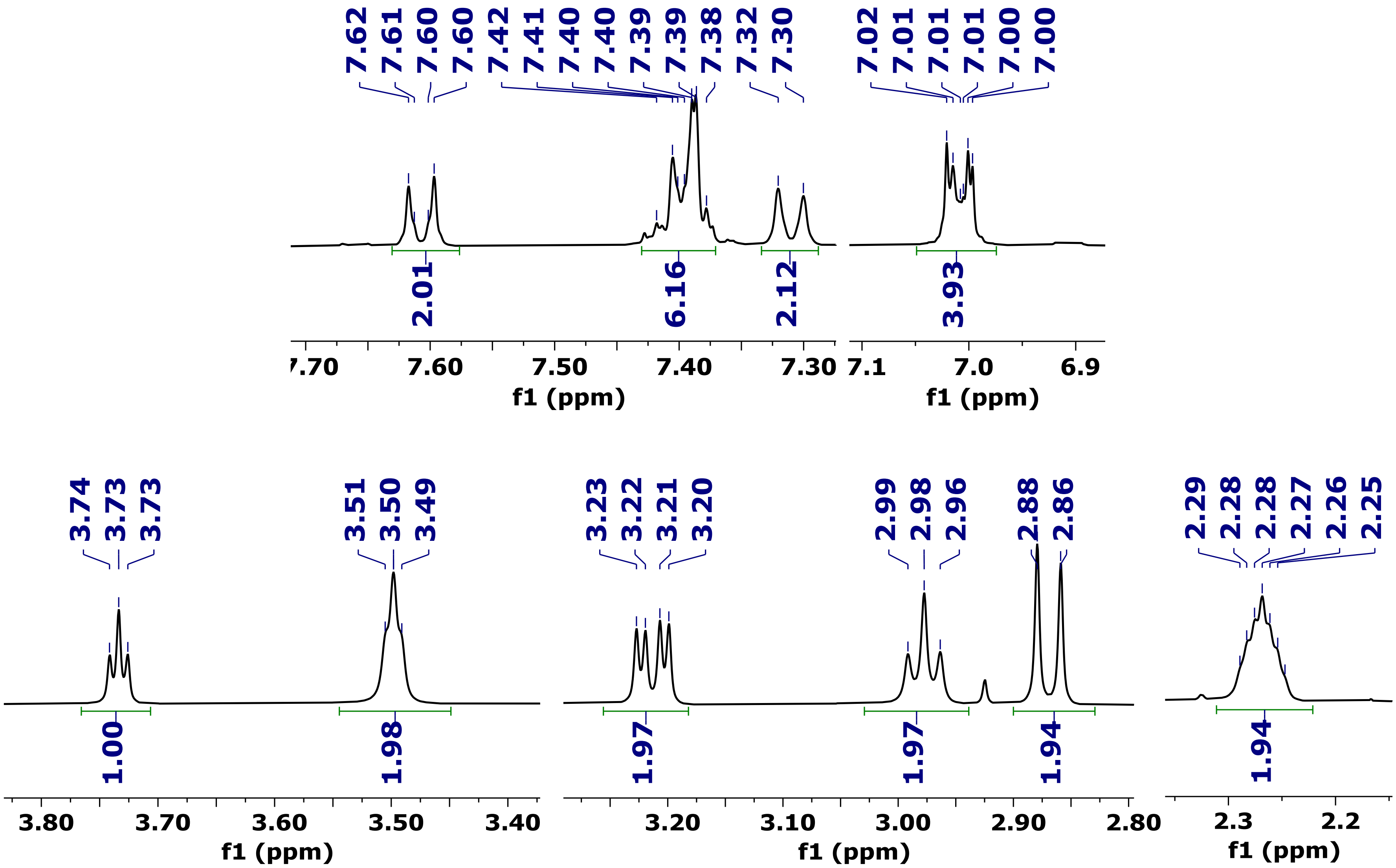
7.62 7.61 7.60 7.60 7.42 7.41 7.40 7.40 7.39 7.39 7.38 7.32 7.30
7.02 7.01 7.01 7.01 7.00 7.00
2.01
6.16
2.12
3.93
7.60 7.50 7.40 7.30
f1 (ppm)
7.1 7.0 6.9
f1 (ppm)
3.74 3.73 3.73 3.51 3.50 3.49
3.23 3.22 3.21 3.20 2.99 2.98 2.96 2.88 2.86
2.29 2.28 2.28 2.27 2.26 2.25
1.00
1.98
1.97
1.97
1.94
1.94
3.80 3.70 3.60 3.50 3.40
f1 (ppm)
3.20 3.10 3.00 2.90 2.80
f1 (ppm)
2.3 2.2
f1 (ppm)

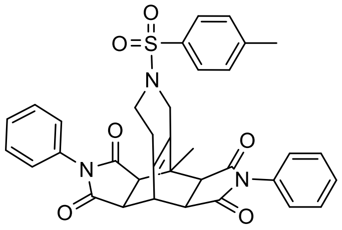

^{13}C NMR (101 MHz, CDCl$_3$)

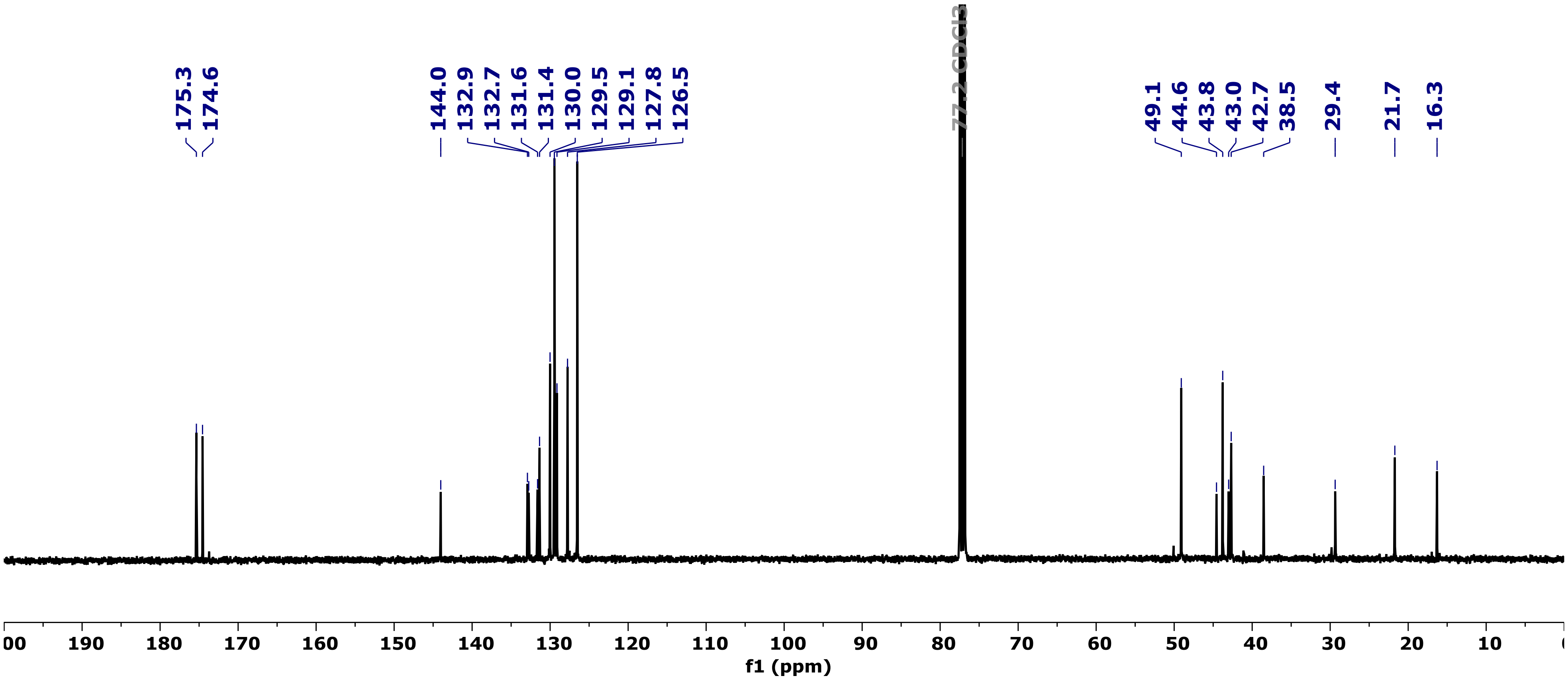

2D NMR HSQC

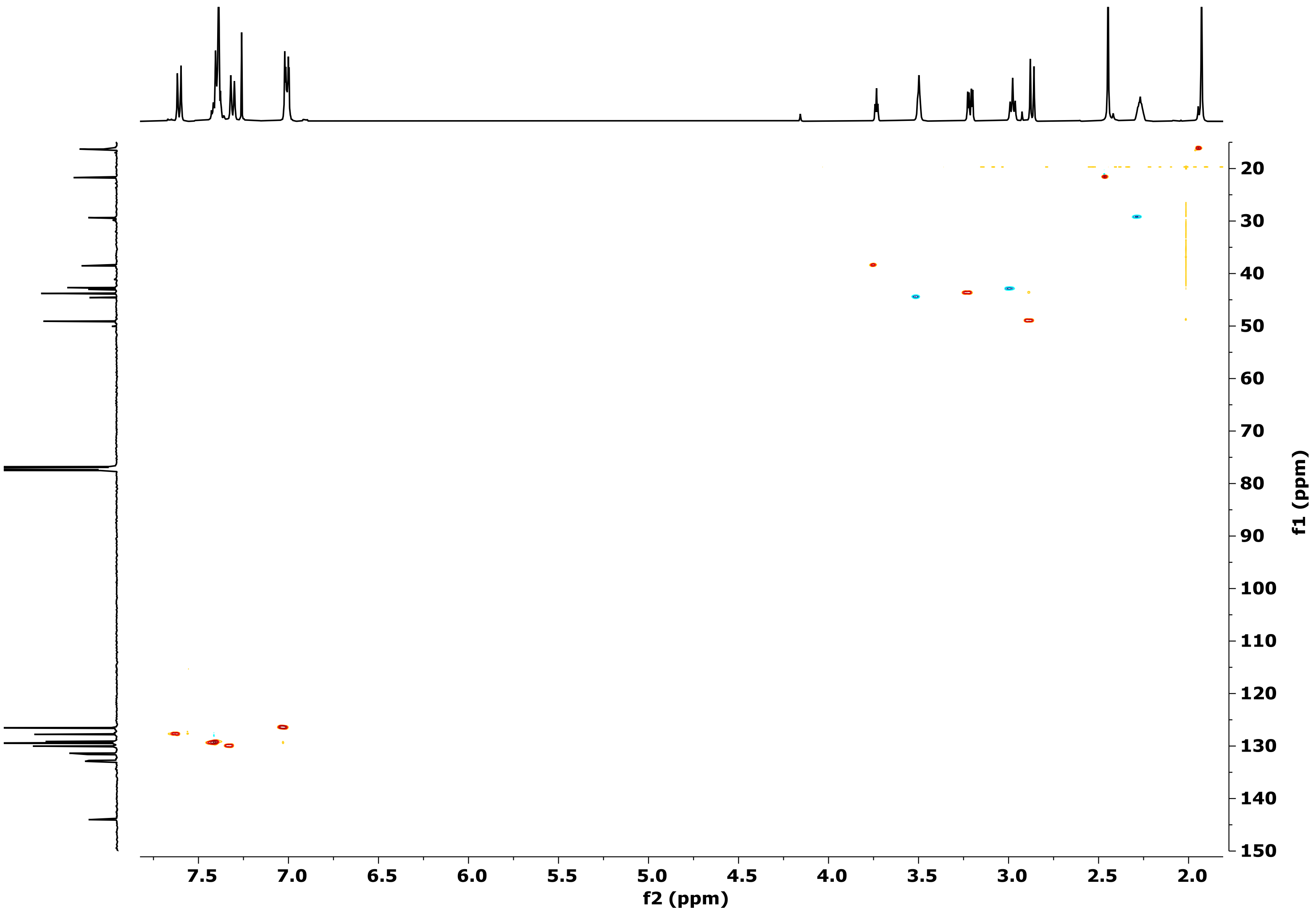

20
30
40
50
60
70
80
90
100
110
120
130
140
150
f1 (ppm)
7.5
7.0
6.5
6.0
5.5
5.0
4.5
4.0
3.5
3.0
2.5
2.0
f2 (ppm)

2D NMR COSY

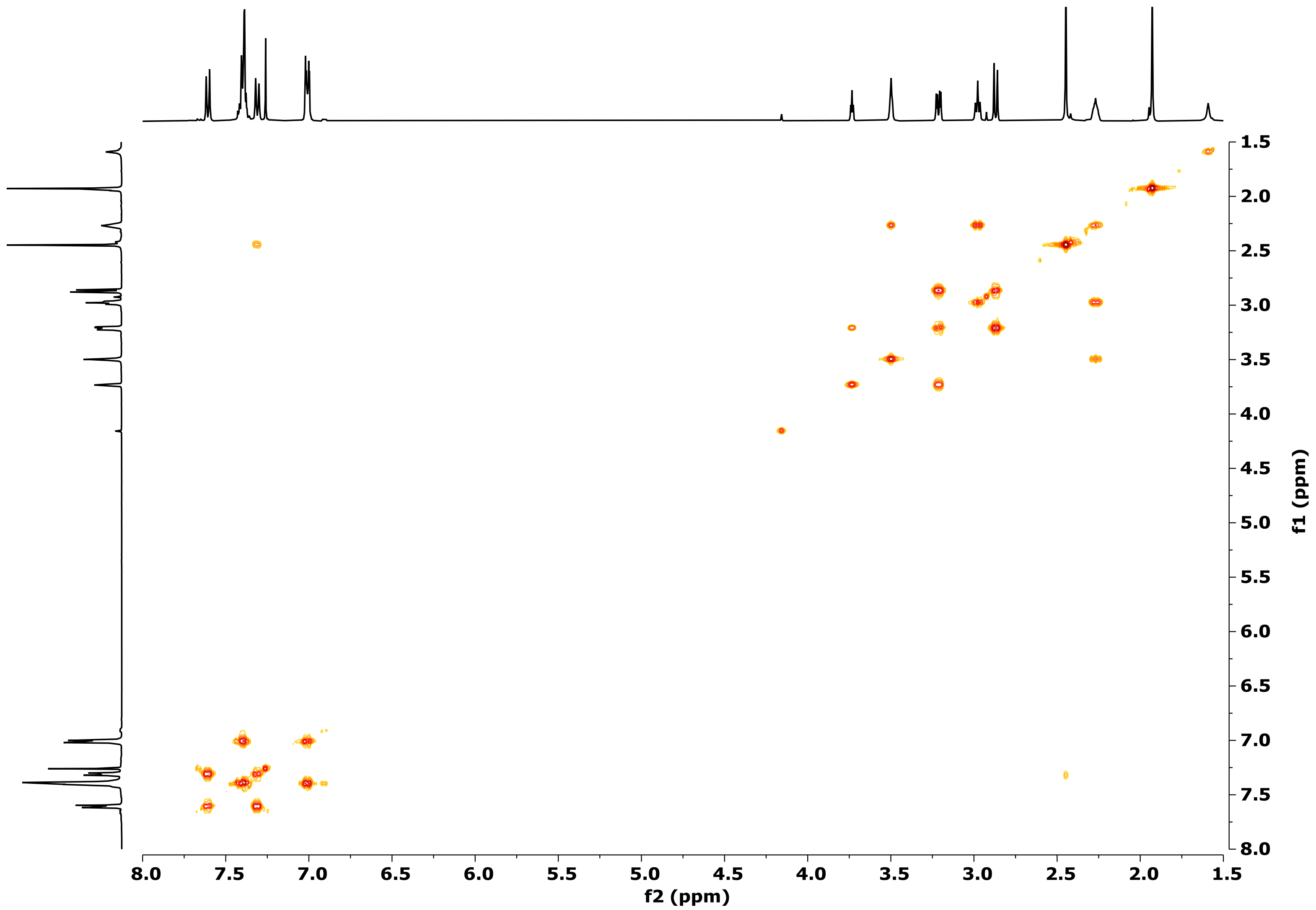

Compound 3b

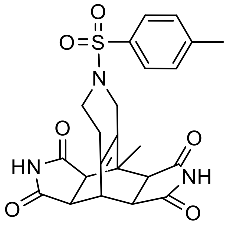

^{1}H NMR (400 MHz, DMSO)

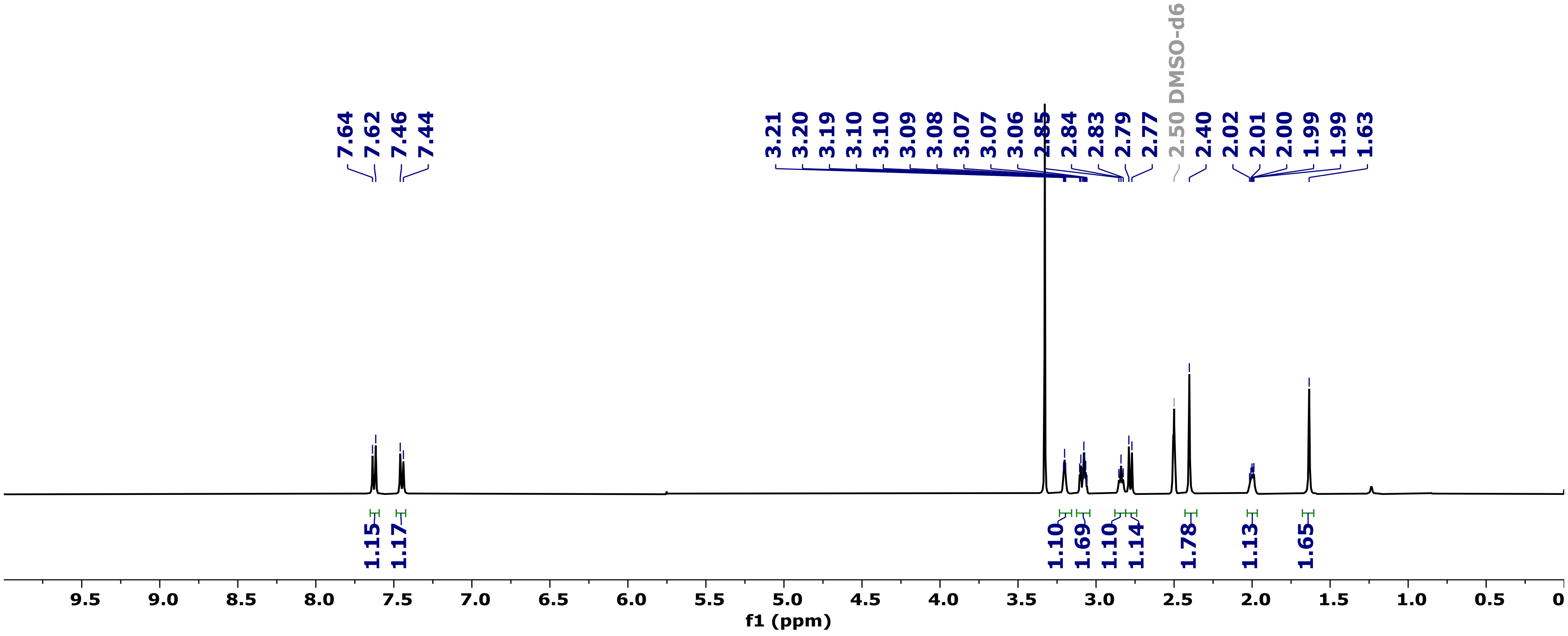

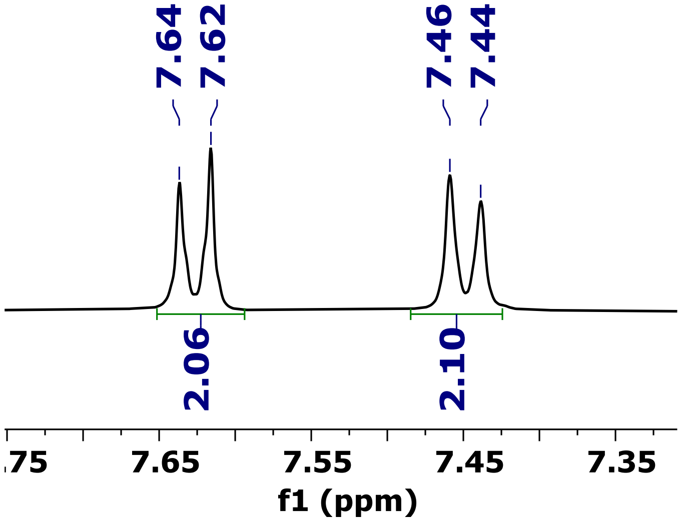
7.64
7.62
7.46
7.44
2.06
2.10
.75
7.65
7.55
7.45
7.35
f1 (ppm)

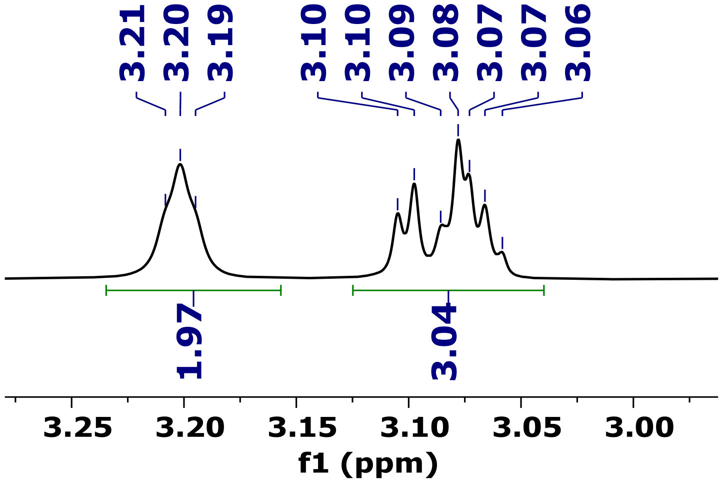
3.21
3.20
3.19
3.10
3.10
3.09
3.08
3.07
3.07
3.06
1.97
3.04
3.25
3.20
3.15
3.10
3.05
3.00
f1 (ppm)

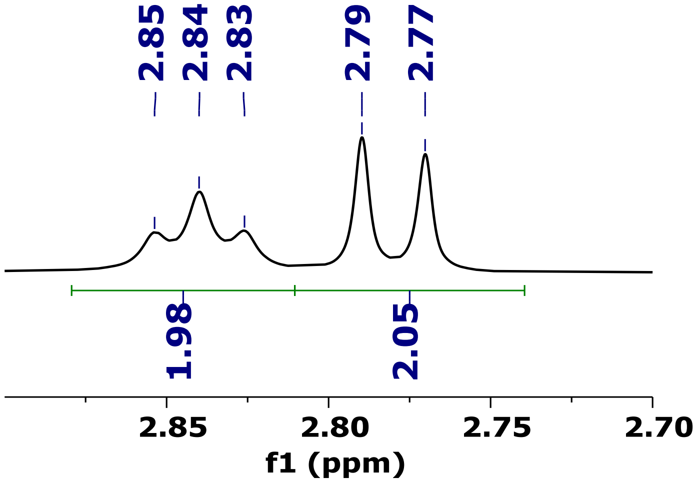
2.85
2.84
2.83
2.79
2.77
1.98
2.05
2.85
2.80
2.75
2.70
f1 (ppm)

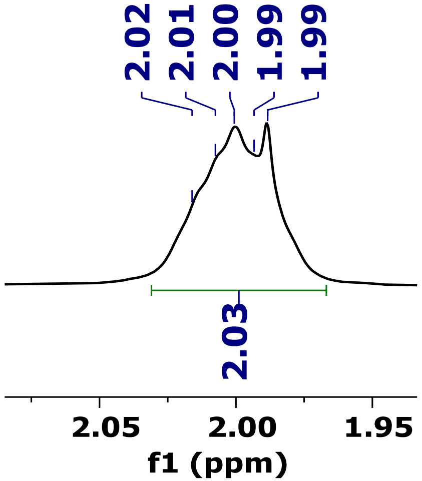
2.02
2.01
2.00
1.99
1.99
2.03
2.05
2.00
1.95
f1 (ppm)

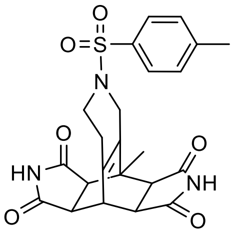

^{13}C NMR (101 MHz, DMSO)

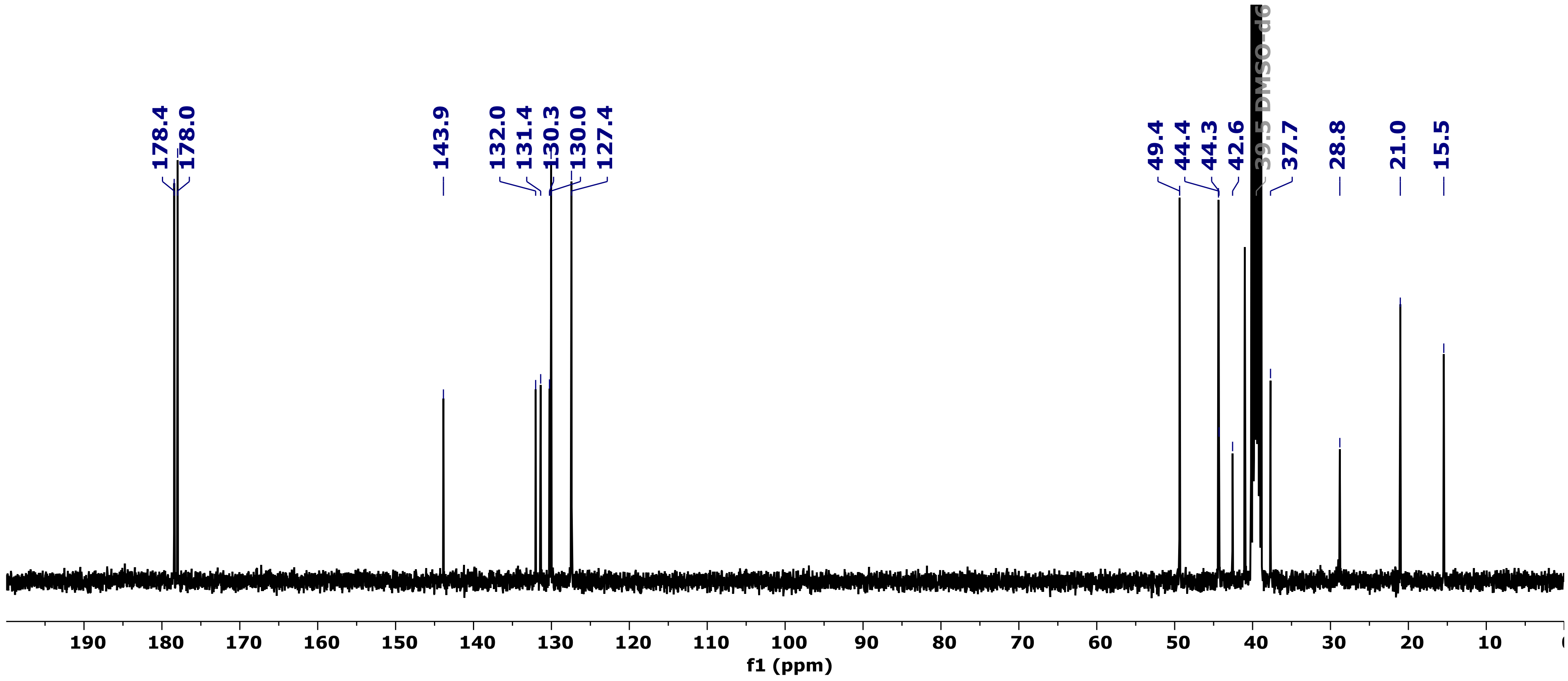

2D NMR HSQC

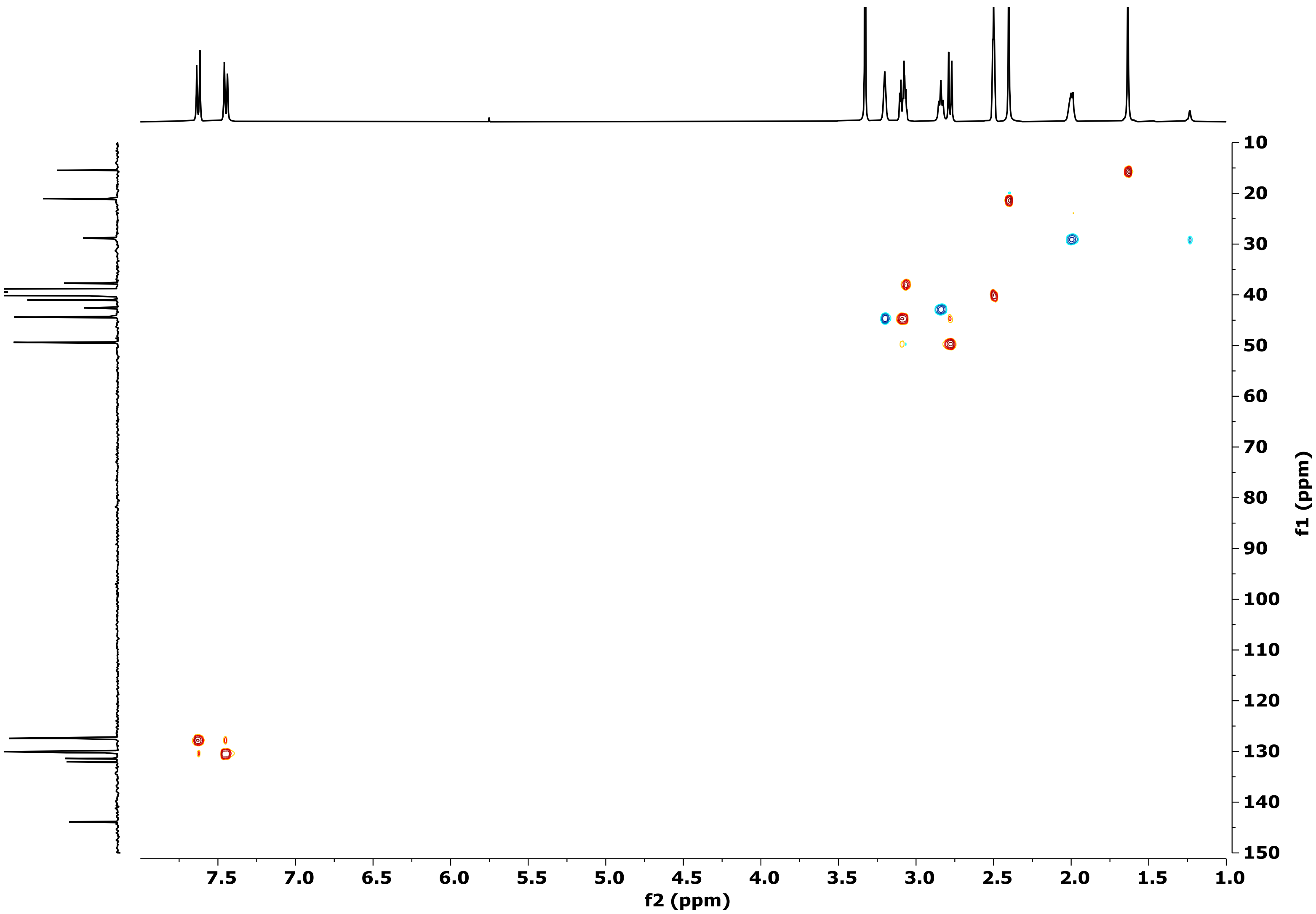

7.5
7.0
6.5
6.0
5.5
5.0
4.5
4.0
3.5
3.0
2.5
2.0
1.5
1.0
f2 (ppm)
10
20
30
40
50
60
70
80
90
100
110
120
130
140
150
f1 (ppm)

2D NMR COSY

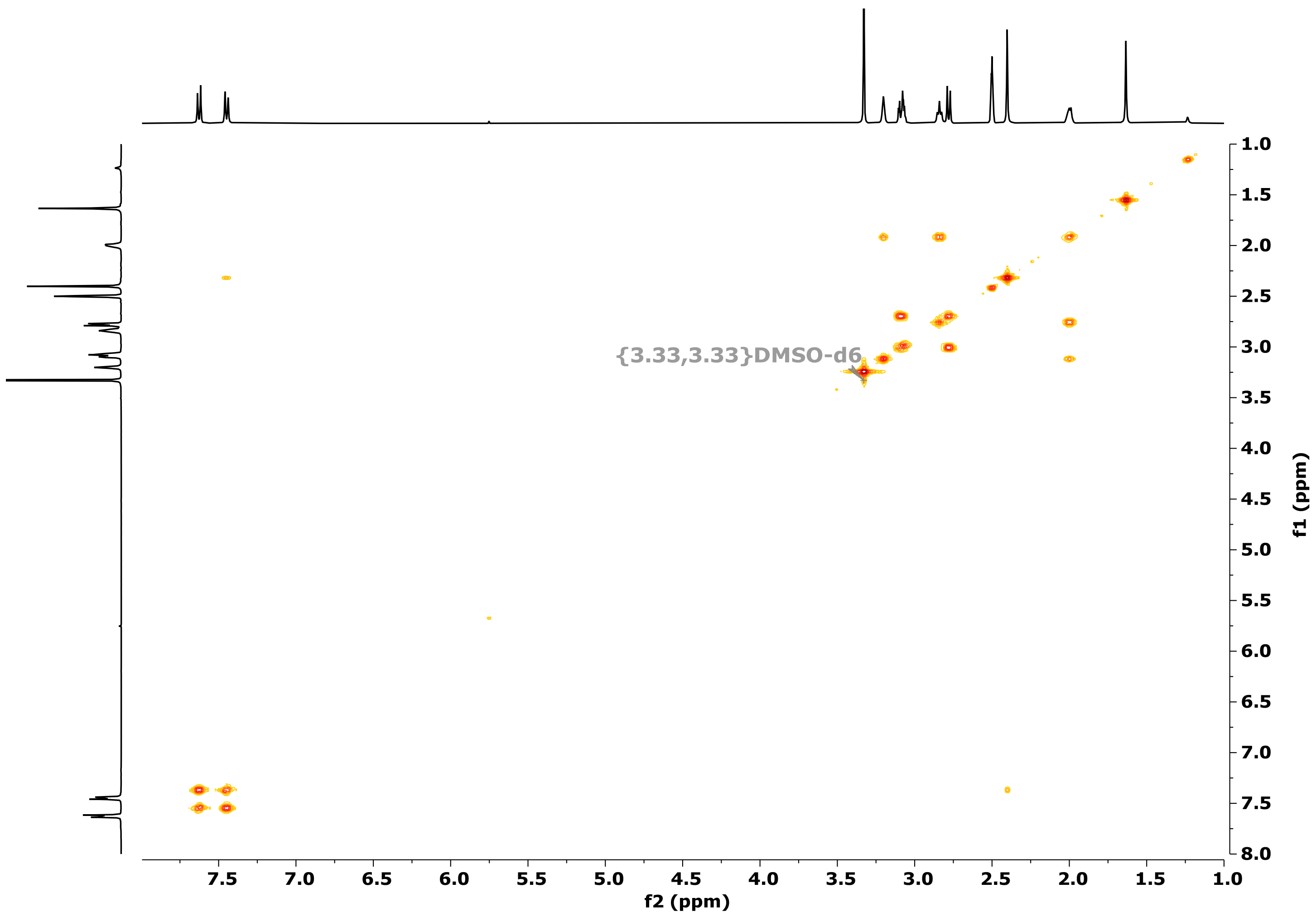
{3.33,3.33}DMSO-d6
f1 (ppm)
f2 (ppm)
1.0
1.5
2.0
2.5
3.0
3.5
4.0
4.5
5.0
5.5
6.0
6.5
7.0
7.5
8.0
7.5
7.0
6.5
6.0
5.5
5.0
4.5
4.0
3.5
3.0
2.5
2.0
1.5
1.0

Compound 3c

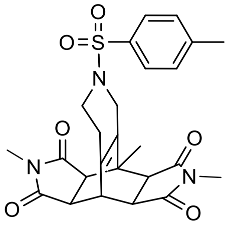

^{1}H NMR (400 MHz, CDCl$_3$)

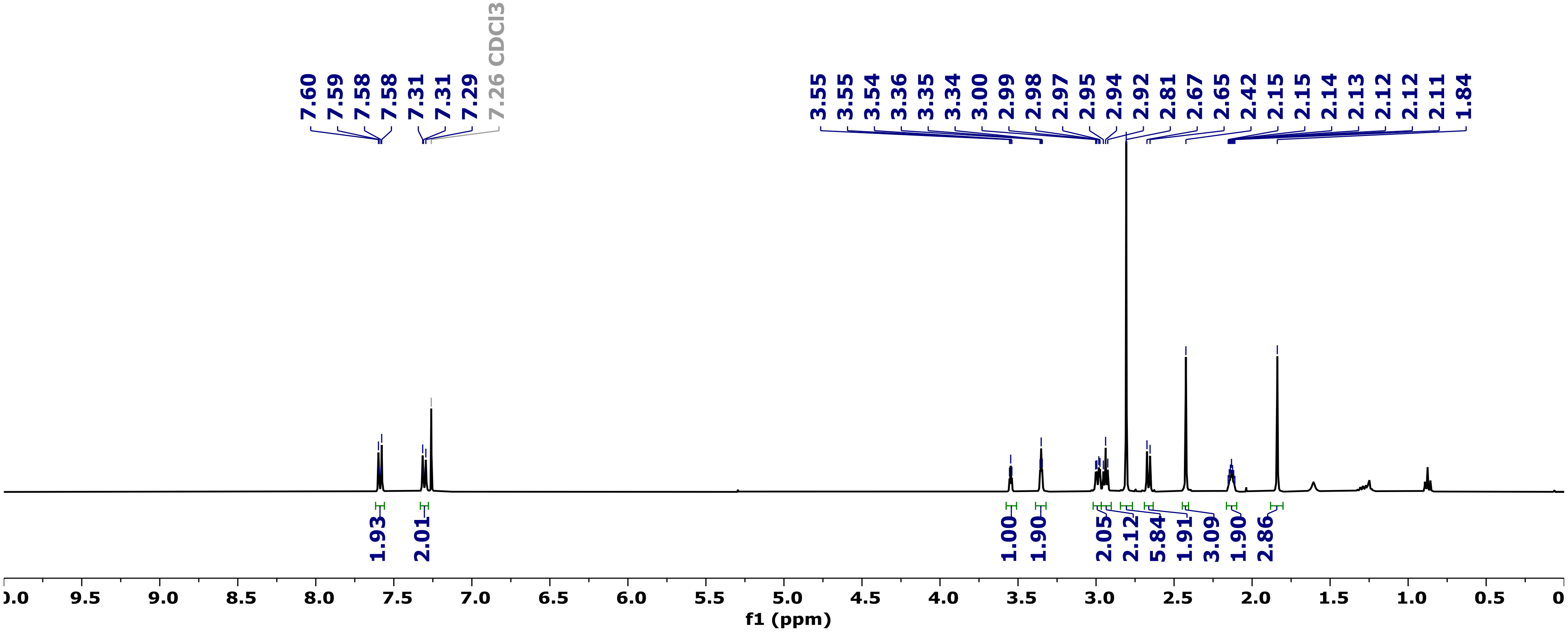

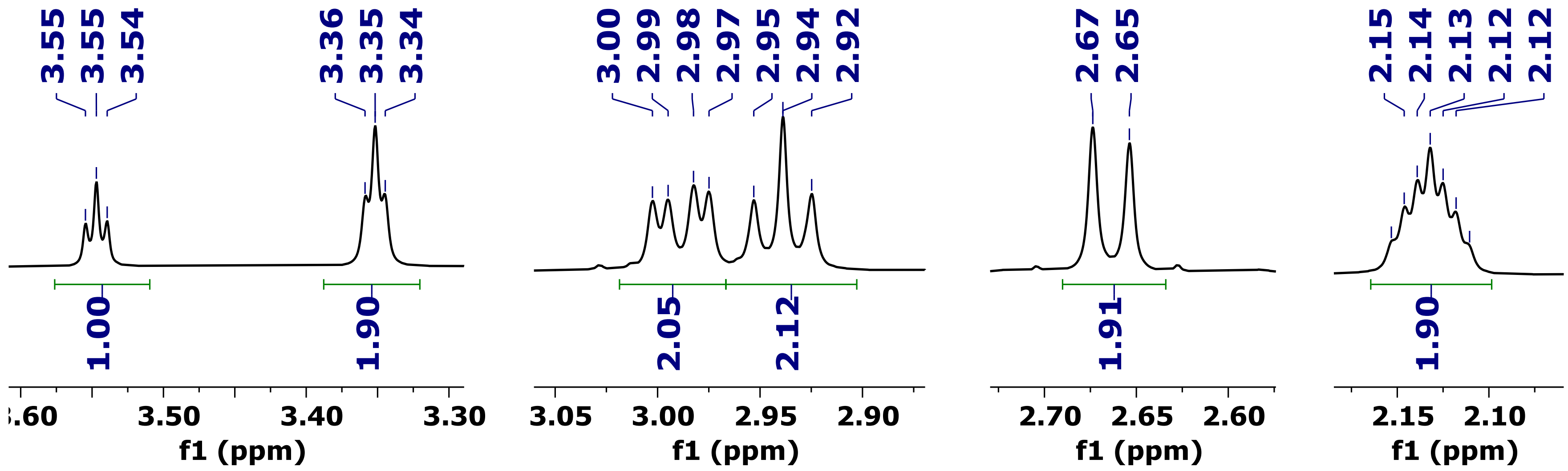
3.55
3.55
3.54
3.36
3.35
3.34
1.00
1.90
3.50
3.40
3.30
f1 (ppm)
3.00
2.99
2.98
2.97
2.95
2.94
2.92
2.05
2.12
3.05
3.00
2.95
2.90
f1 (ppm)
2.67
2.65
1.91
2.70
2.65
2.60
f1 (ppm)
2.15
2.14
2.13
2.12
2.12
1.90
2.15
2.10
f1 (ppm)

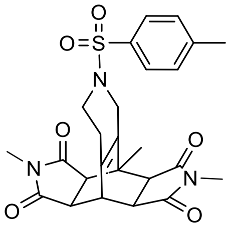

^{13}C NMR (101 MHz, $CDCl_3$)

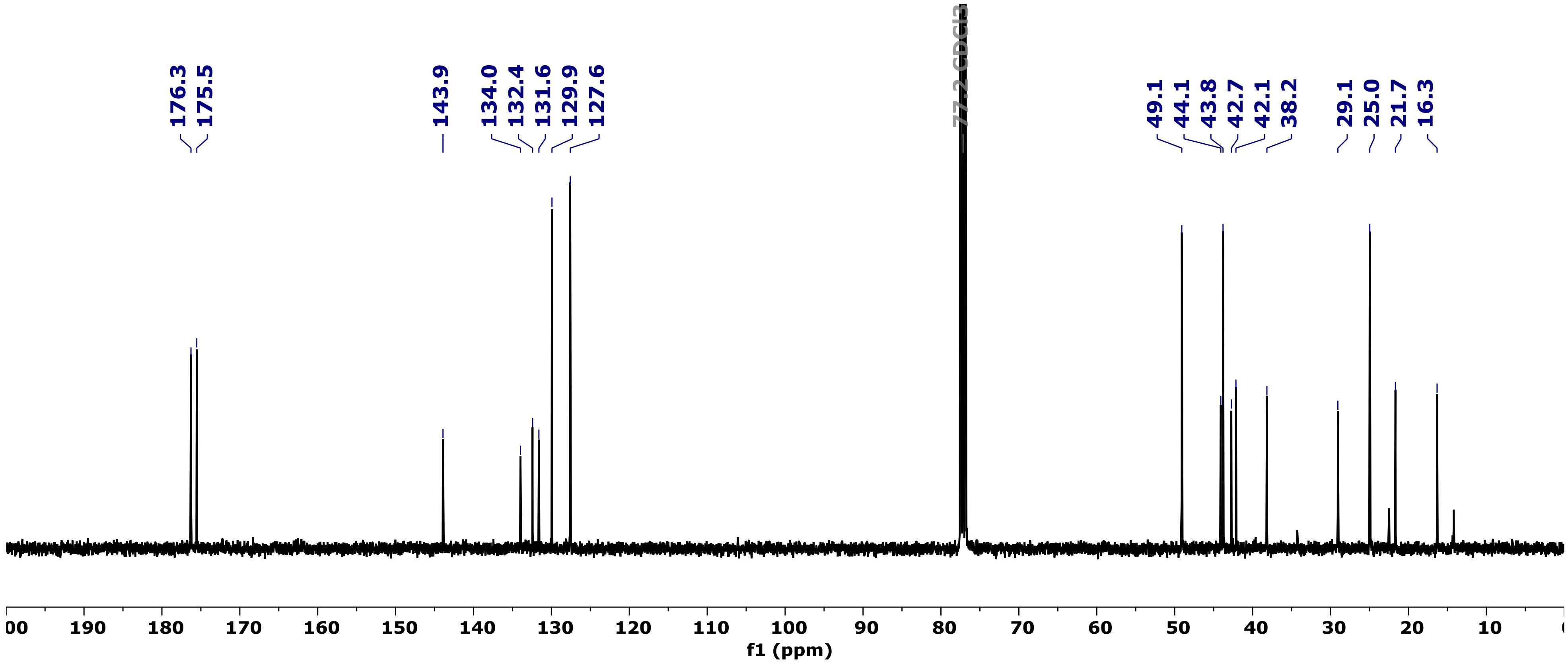

2D NMR HSQC

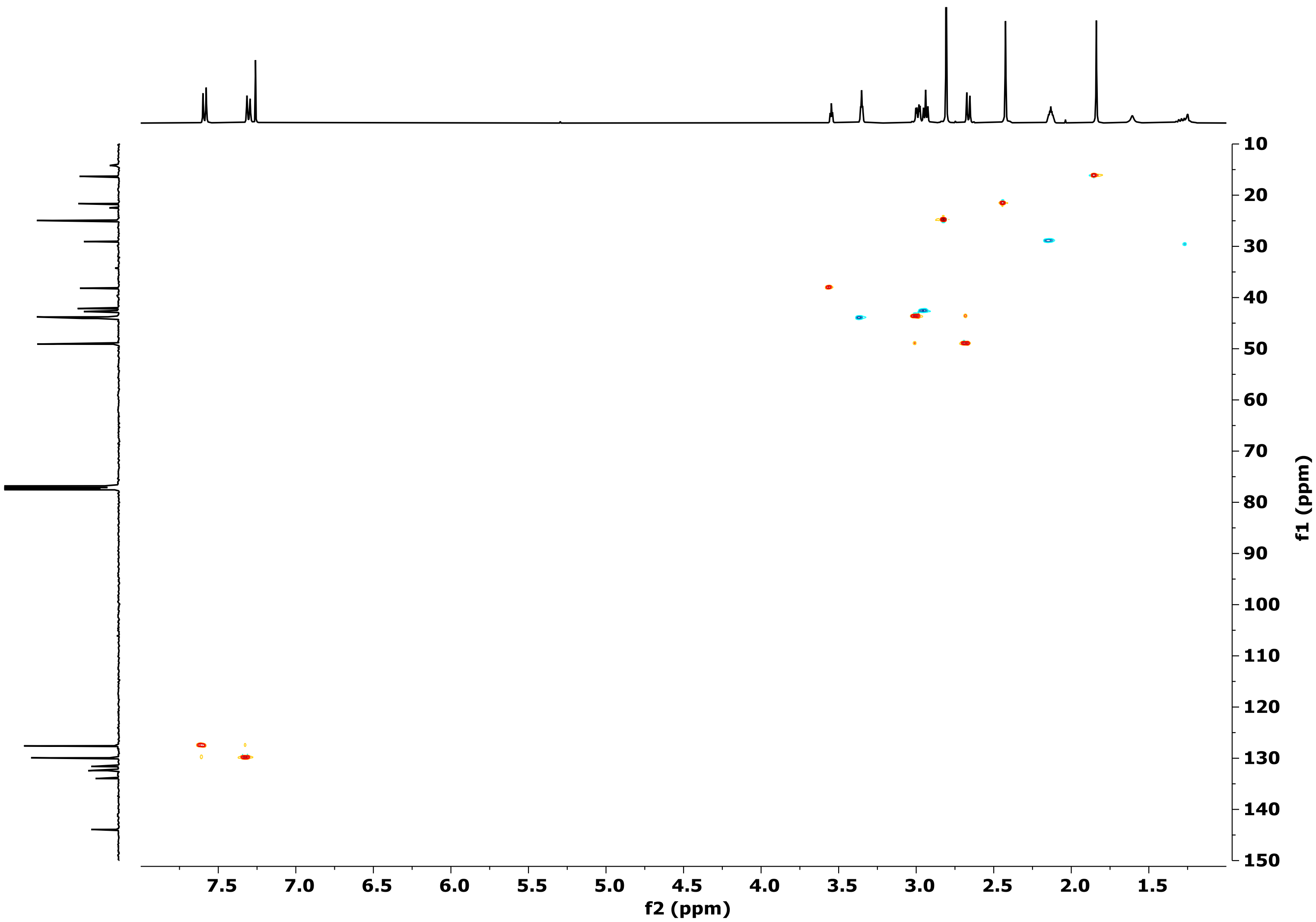

Compound 3d

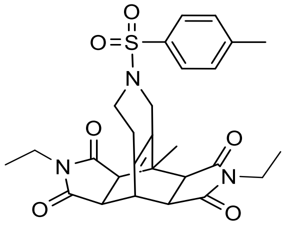

^{1}H NMR (400 MHz, $CDCl_3$)

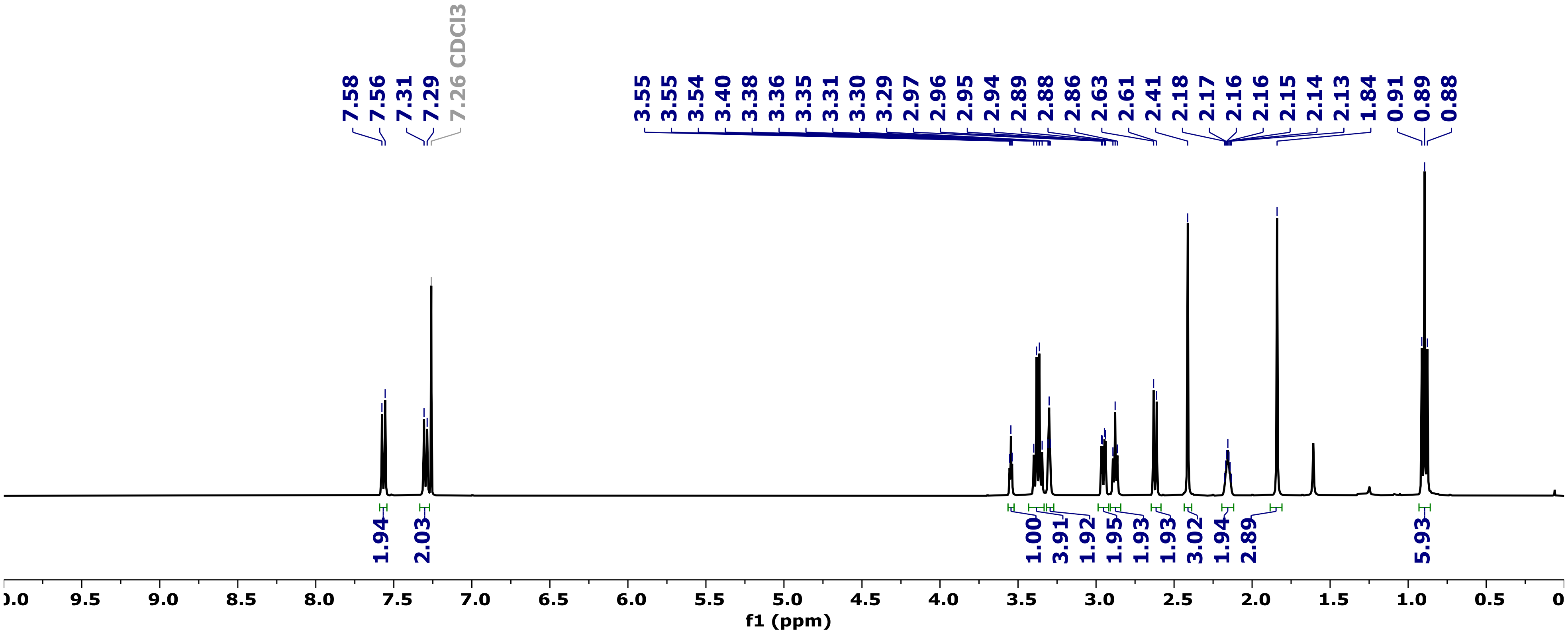

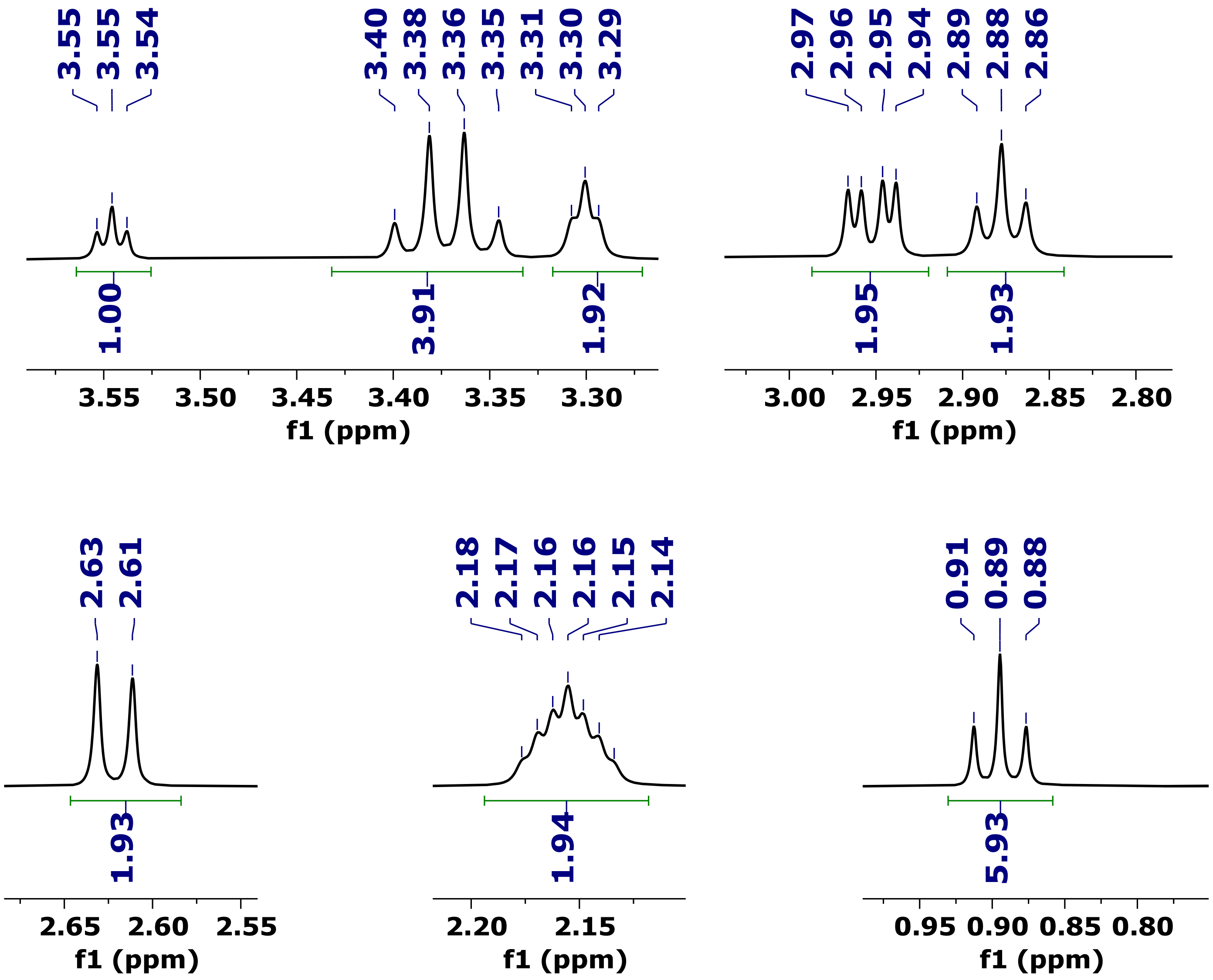
3.55 3.55 3.54 3.40 3.38 3.36 3.35 3.31 3.30 3.29
1.00 3.91 1.92
3.55 3.50 3.45 3.40 3.35 3.30
f1 (ppm)
2.97 2.96 2.95 2.94 2.89 2.88 2.86
1.95 1.93
3.00 2.95 2.90 2.85 2.80
f1 (ppm)
2.63 2.61
1.93
2.65 2.60 2.55
f1 (ppm)
2.18 2.17 2.16 2.16 2.15 2.14
1.94
2.20 2.15
f1 (ppm)
0.91 0.89 0.88
5.93
0.95 0.90 0.85 0.80
f1 (ppm)

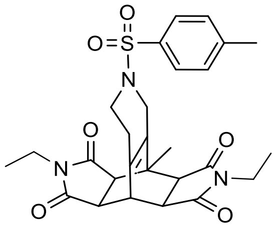

^{13}C NMR (101 MHz, CDCl₃)

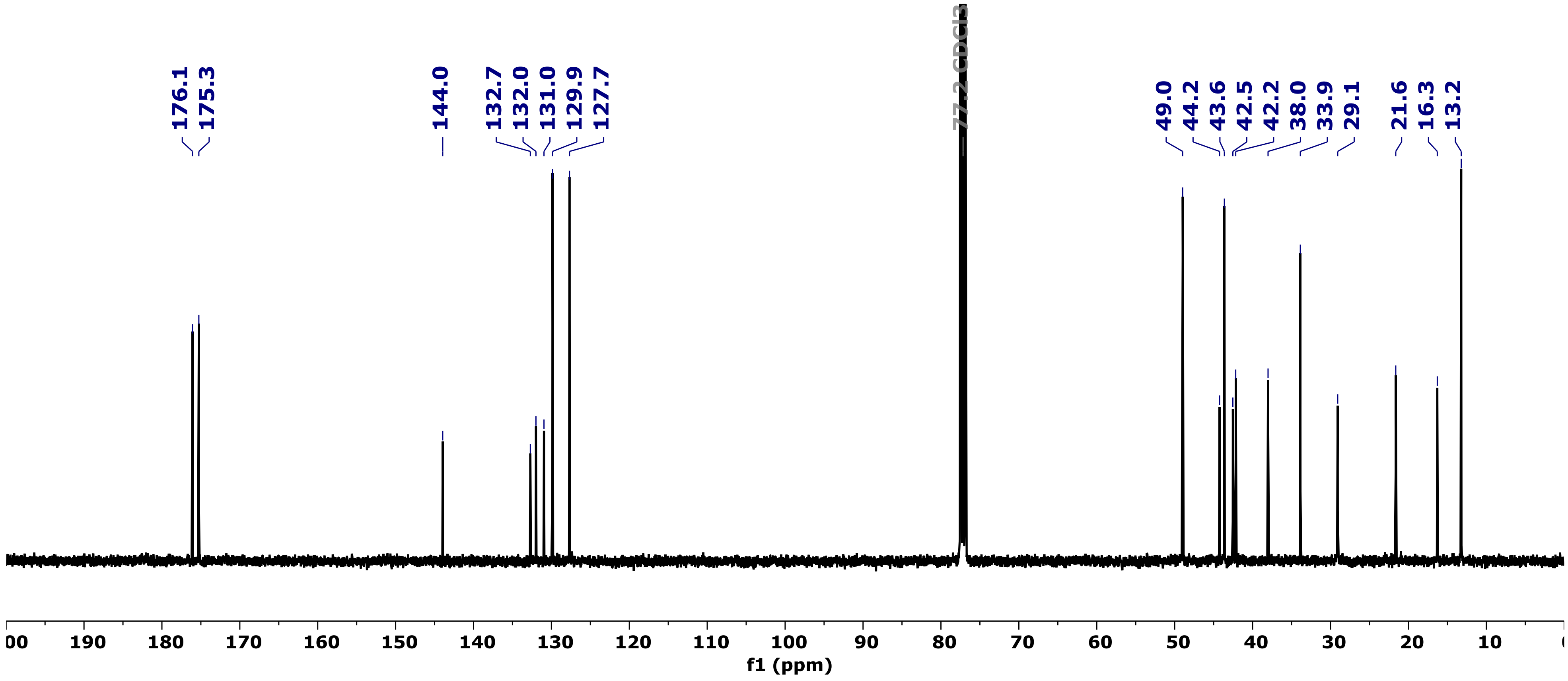

2D NMR HSQC

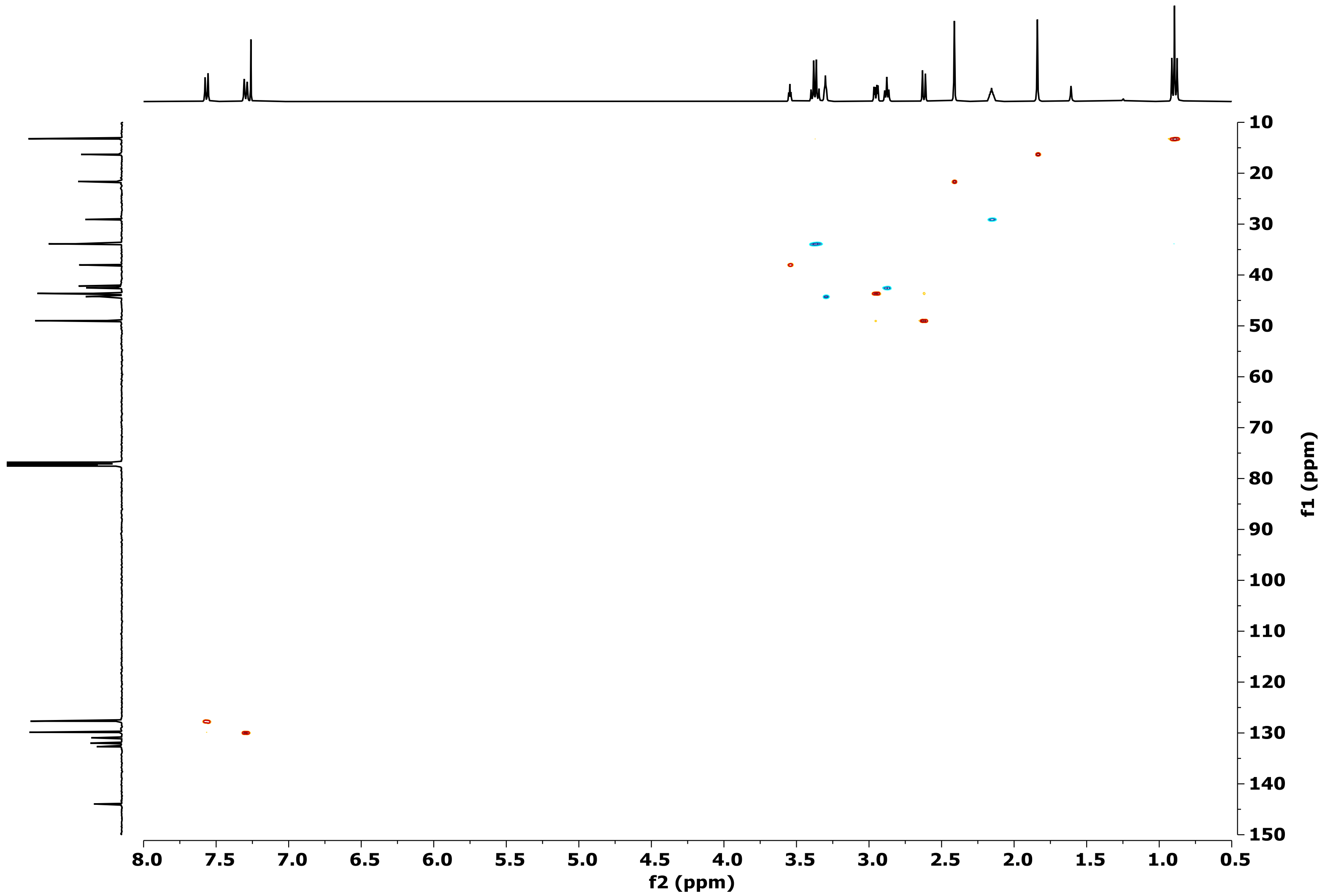

2D NMR COSY

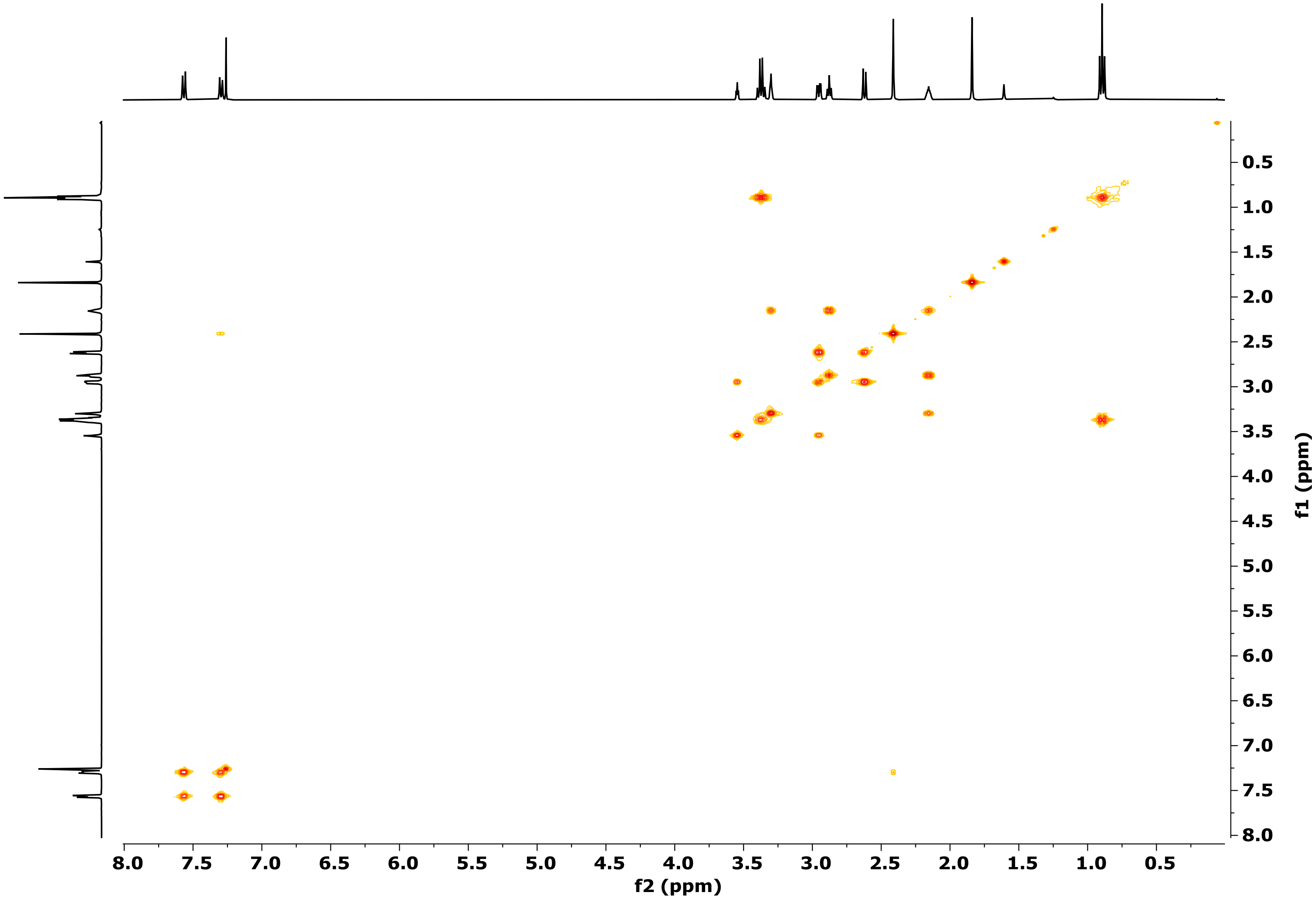

2D NMR HMBC

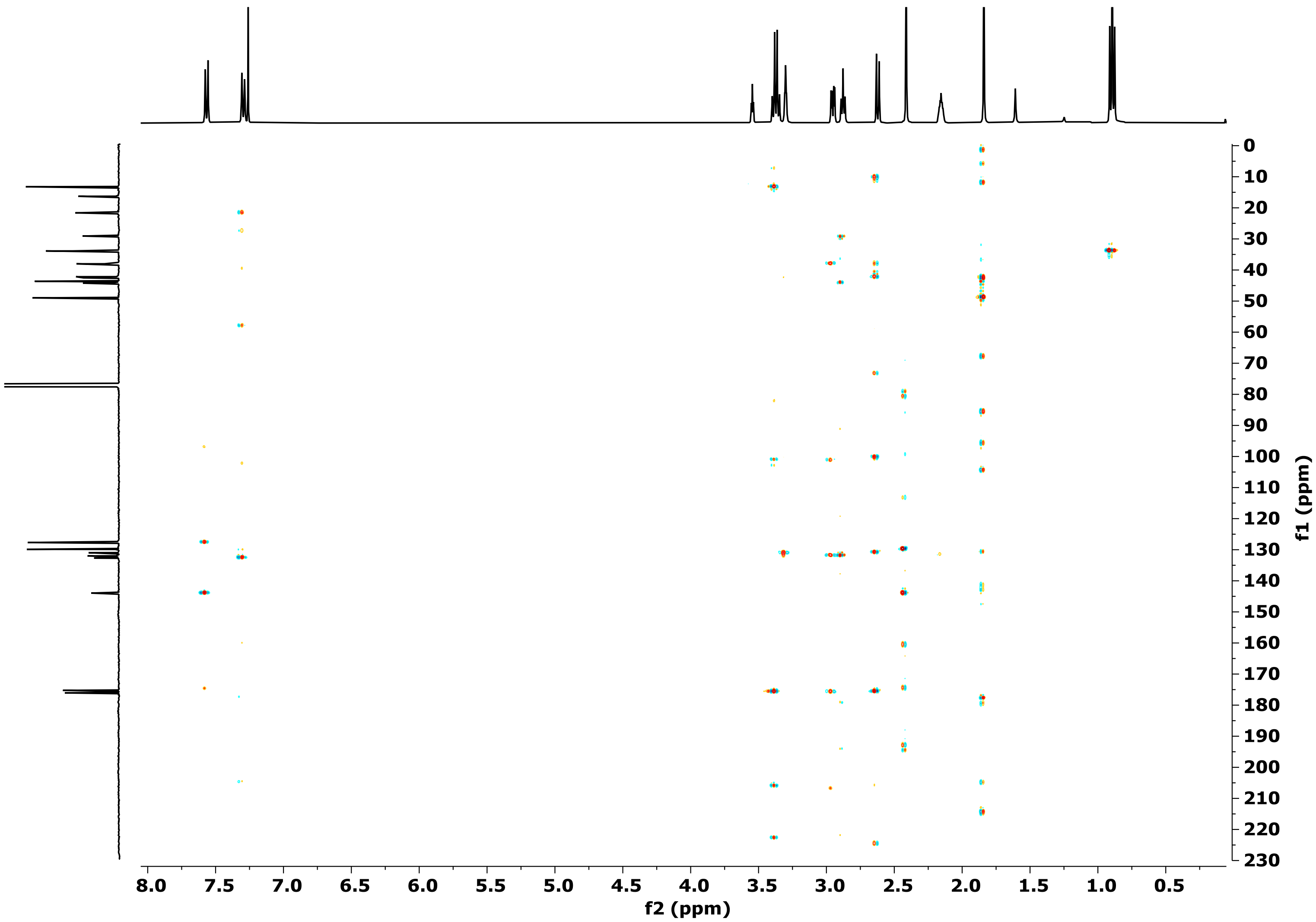

0
10
20
30
40
50
60
70
80
90
100
110
120
130
140
150
160
170
180
190
200
210
220
230
f1 (ppm)
8.0
7.5
7.0
6.5
6.0
5.5
5.0
4.5
4.0
3.5
3.0
2.5
2.0
1.5
1.0
0.5
f2 (ppm)

Compound 3e

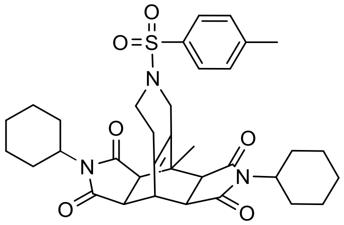

^{1}H NMR (400 MHz, CDCl$_3$

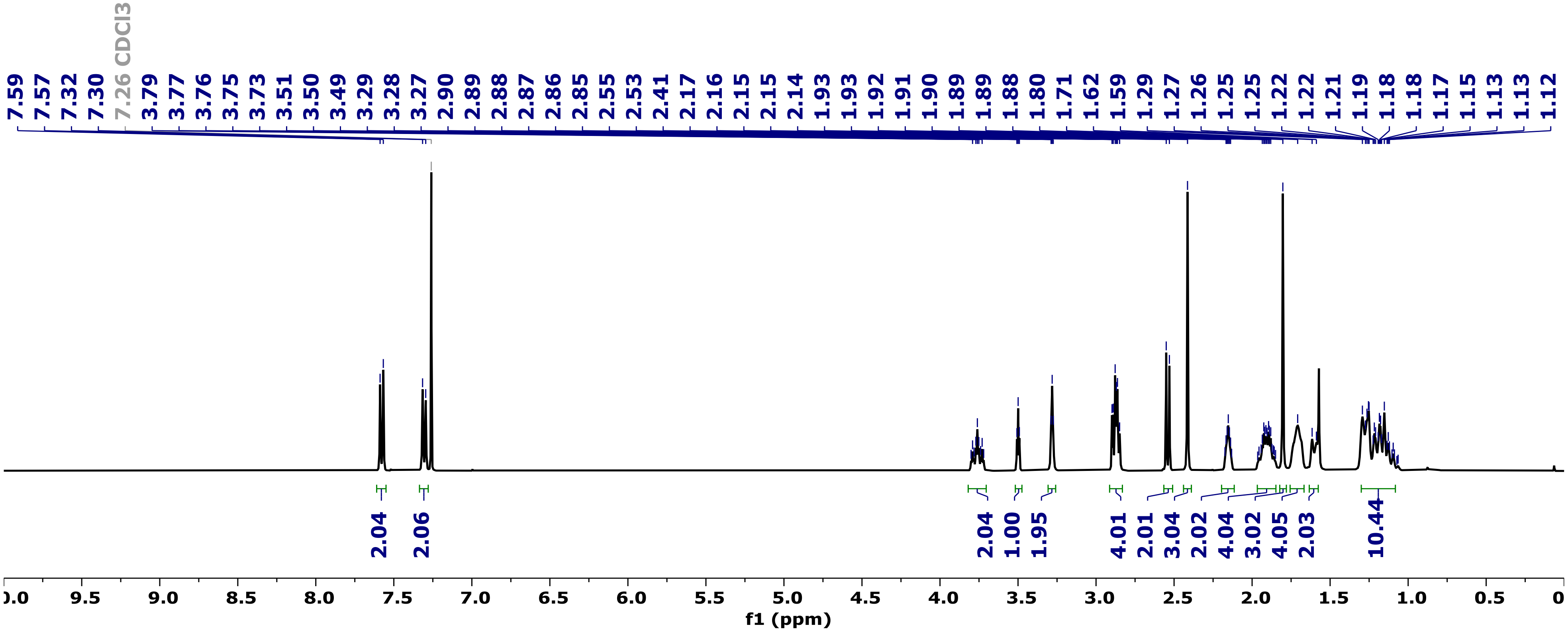

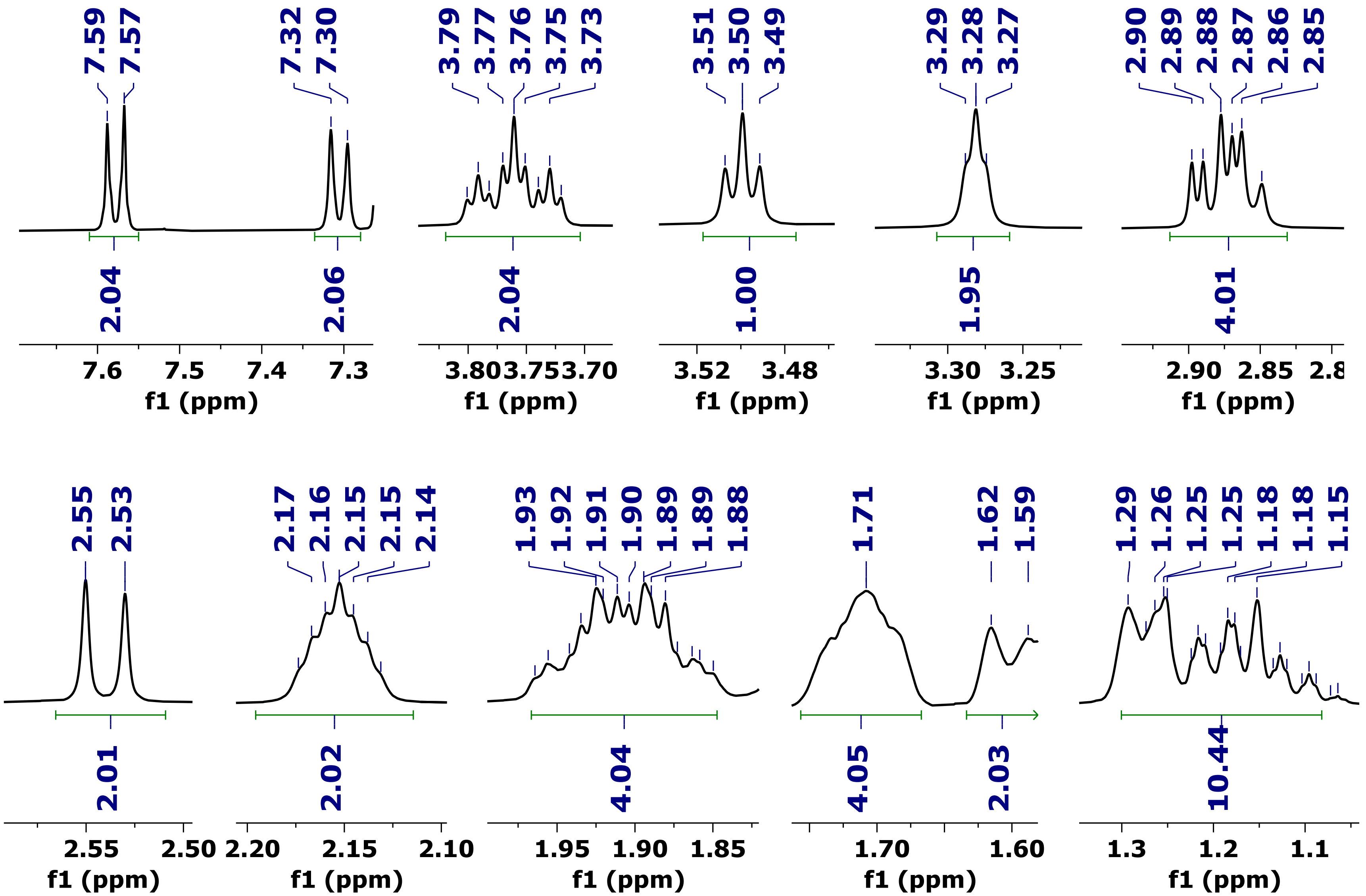
7.59 7.57 7.32 7.30
2.04 2.06
7.6 7.5 7.4 7.3
f1 (ppm)
3.79 3.77 3.76 3.75 3.73
2.04
3.80 3.75 3.70
f1 (ppm)
3.51 3.50 3.49
1.00
3.52 3.48
f1 (ppm)
3.29 3.28 3.27
1.95
3.30 3.25
f1 (ppm)
2.90 2.89 2.88 2.87 2.86 2.85
4.01
2.90 2.85 2.8
f1 (ppm)
2.55 2.53
2.01
2.55 2.50
f1 (ppm)
2.17 2.16 2.15 2.15 2.14
2.02
2.20 2.15 2.10
f1 (ppm)
1.93 1.92 1.91 1.90 1.89 1.89 1.88
4.04
1.95 1.90 1.85
f1 (ppm)
1.71 1.62 1.59
4.05 2.03
1.70 1.60
f1 (ppm)
1.29 1.26 1.25 1.25 1.18 1.18 1.15
10.44
1.3 1.2 1.1
f1 (ppm)

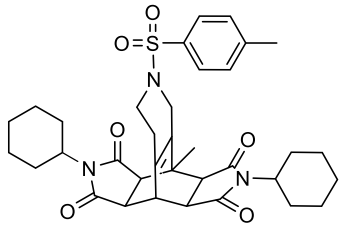

^{13}C NMR (101 MHz, CDCl$_3$)

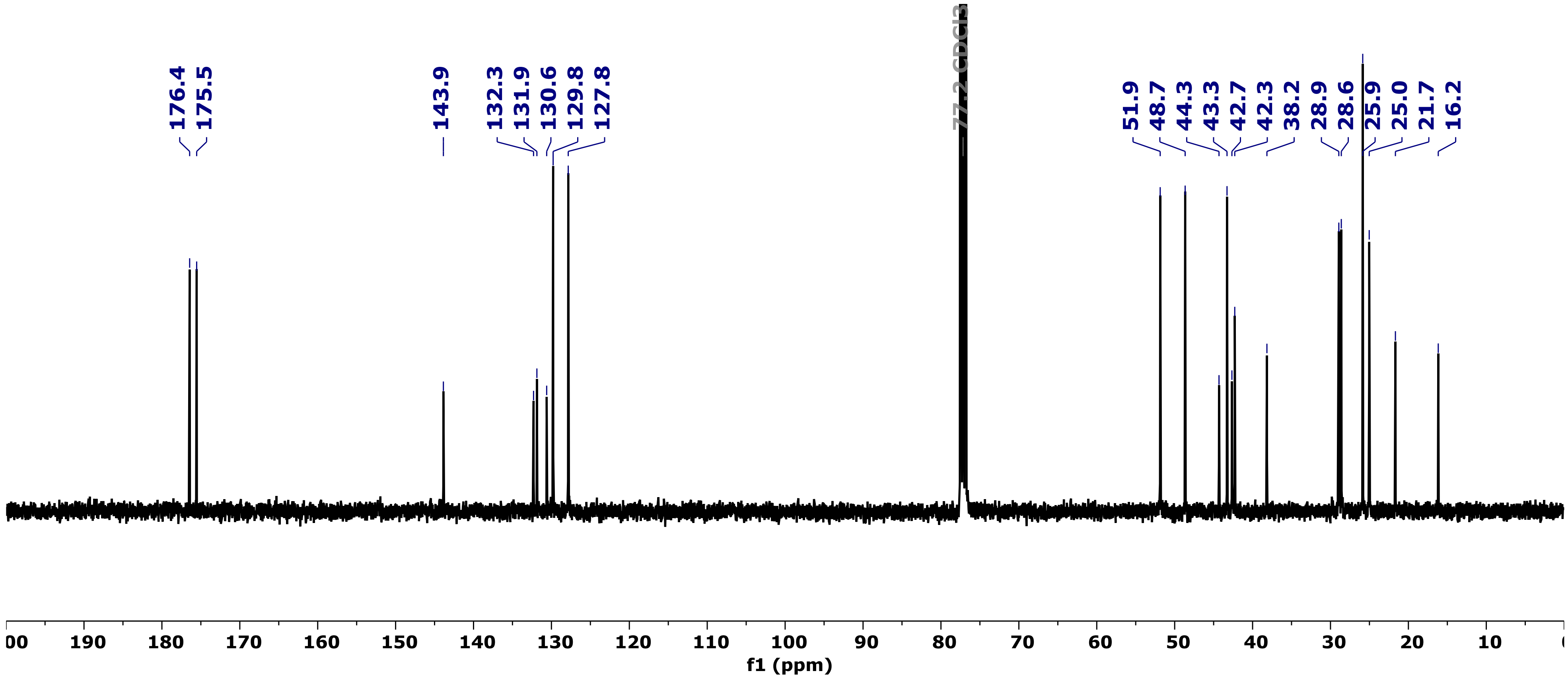

2D NMR HSQC

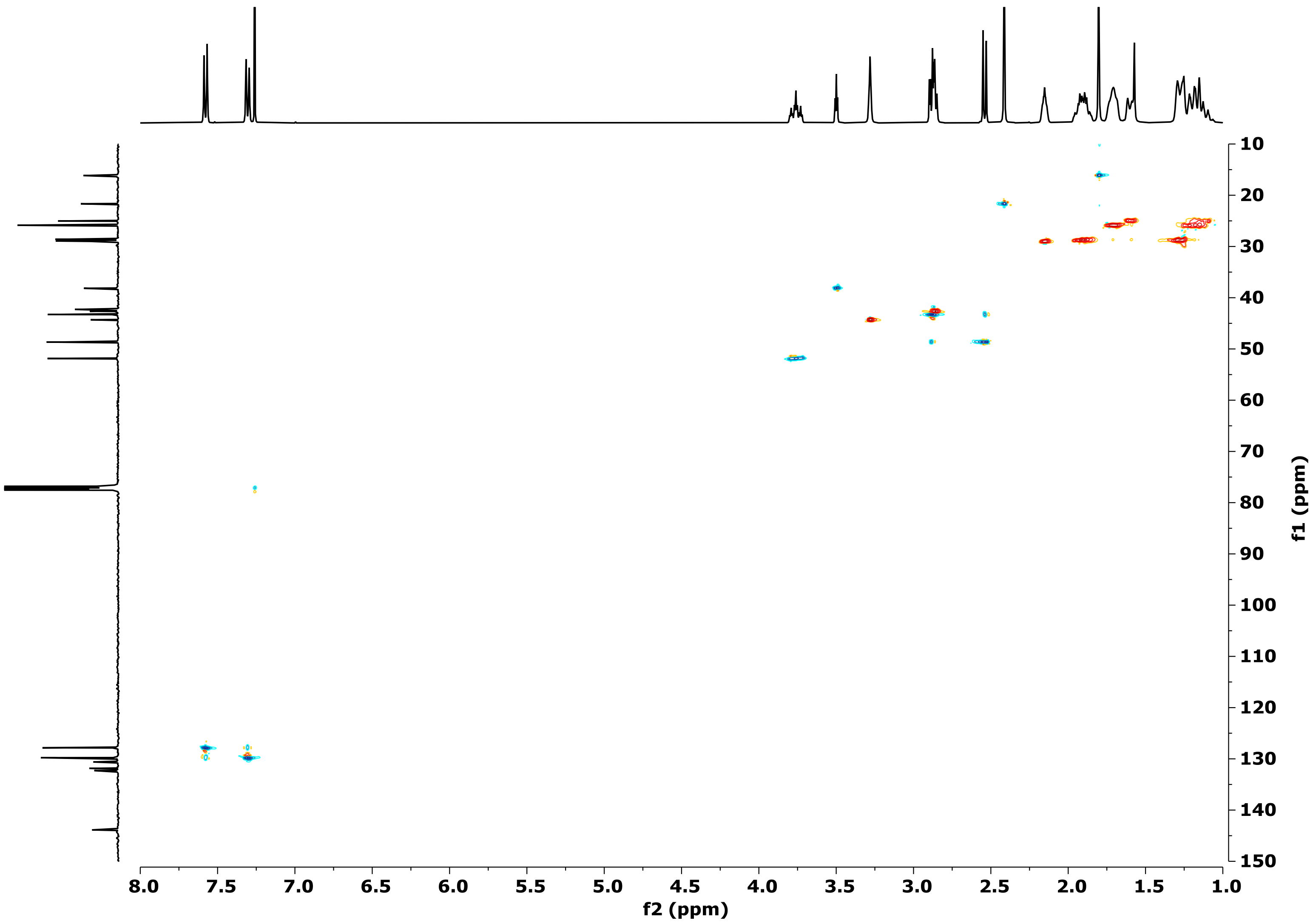

10
20
30
40
50
60
70
80
90
100
110
120
130
140
150
f1 (ppm)
8.0
7.5
7.0
6.5
6.0
5.5
5.0
4.5
4.0
3.5
3.0
2.5
2.0
1.5
1.0
f2 (ppm)

2D NMR COSY

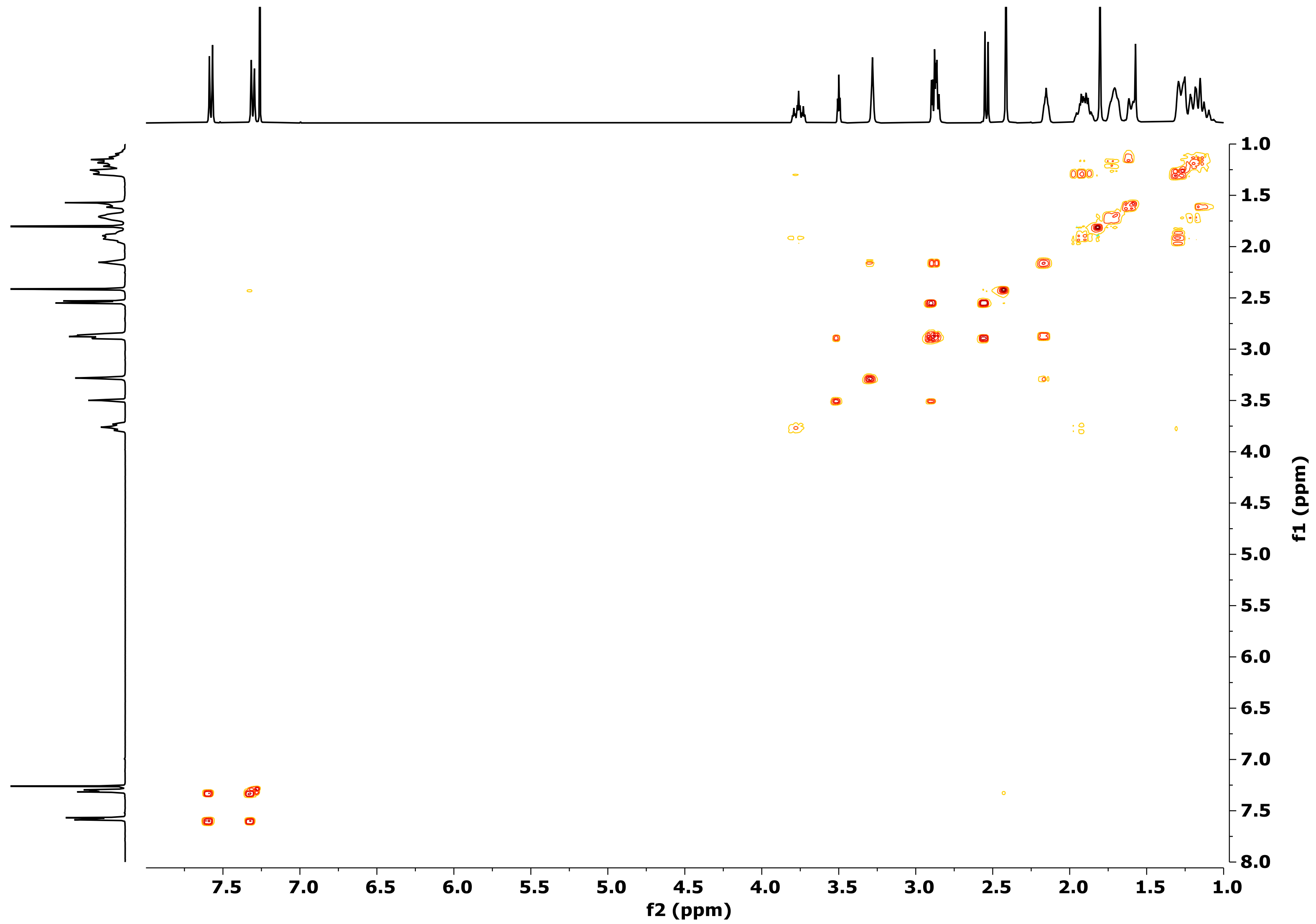

1.0
1.5
2.0
2.5
3.0
3.5
4.0
4.5
5.0
5.5
6.0
6.5
7.0
7.5
8.0
f1 (ppm)
7.5
7.0
6.5
6.0
5.5
5.0
4.5
4.0
3.5
3.0
2.5
2.0
1.5
1.0
f2 (ppm)

Compound 3f

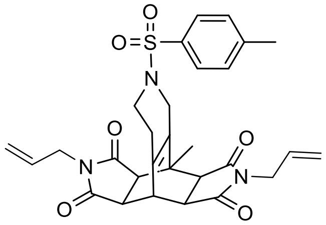

^{1}H NMR (400 MHz, $CDCl_3$)

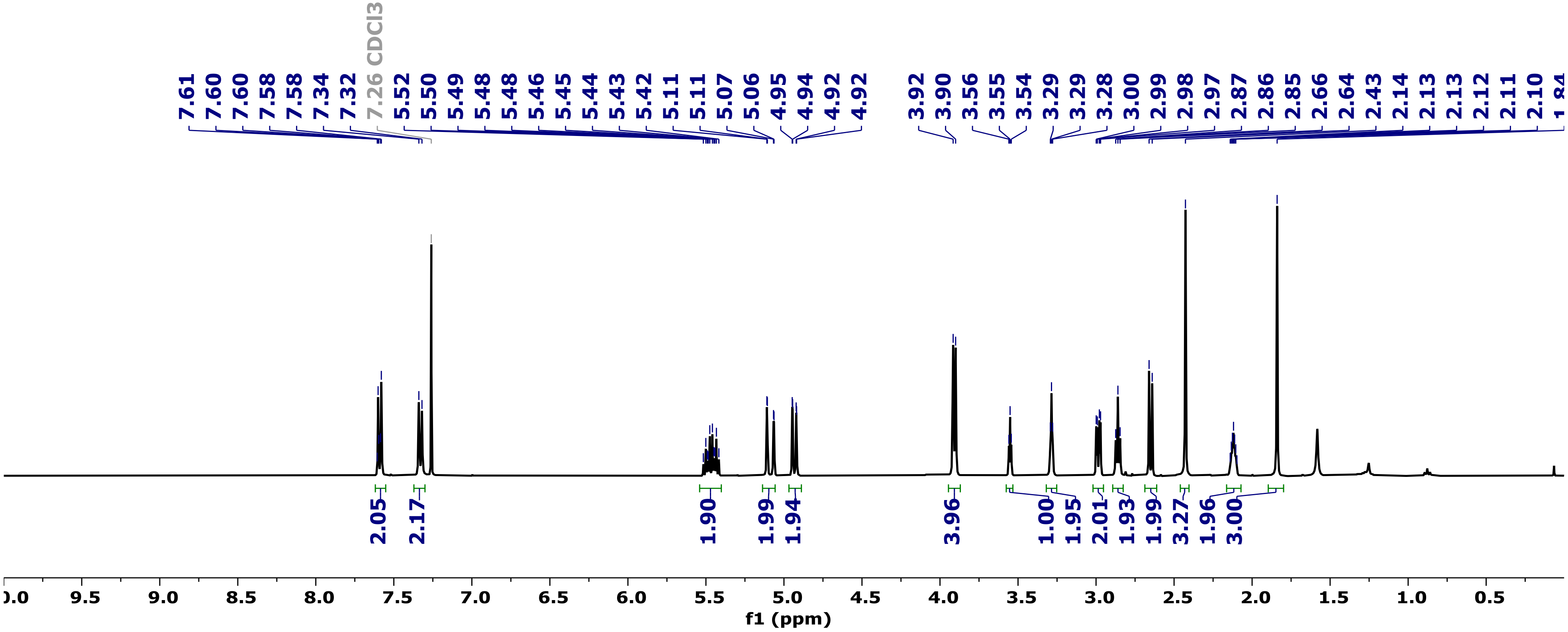

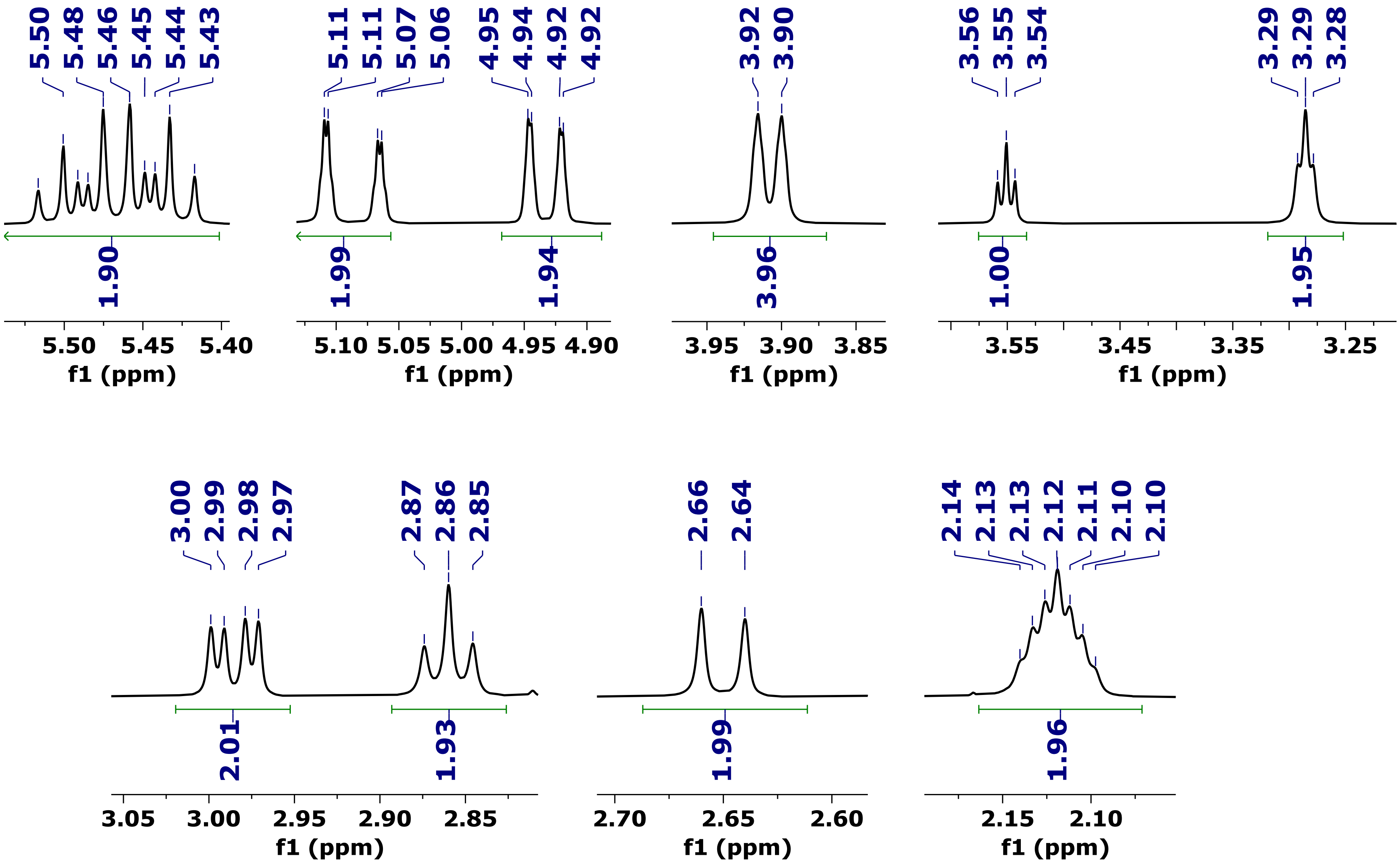
5.50 5.48 5.46 5.45 5.44 5.43
1.90
5.50 5.45 5.40
f1 (ppm)
5.11 5.11 5.07 5.06 4.95 4.94 4.92 4.92
1.99
1.94
5.10 5.05 5.00 4.95 4.90
f1 (ppm)
3.92 3.90
3.96
3.95 3.90 3.85
f1 (ppm)
3.56 3.55 3.54 3.29 3.29 3.28
1.00
1.95
3.55 3.45 3.35 3.25
f1 (ppm)
3.00 2.99 2.98 2.97 2.87 2.86 2.85
2.01
1.93
3.05 3.00 2.95 2.90 2.85
f1 (ppm)
2.66 2.64
1.99
2.70 2.65 2.60
f1 (ppm)
2.14 2.13 2.13 2.12 2.11 2.10 2.10
1.96
2.15 2.10
f1 (ppm)

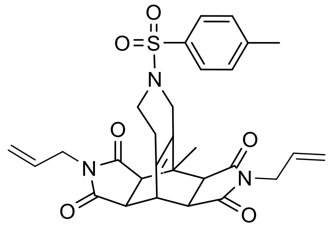

^{13}C NMR (101 MHz, $CDCl_3$)

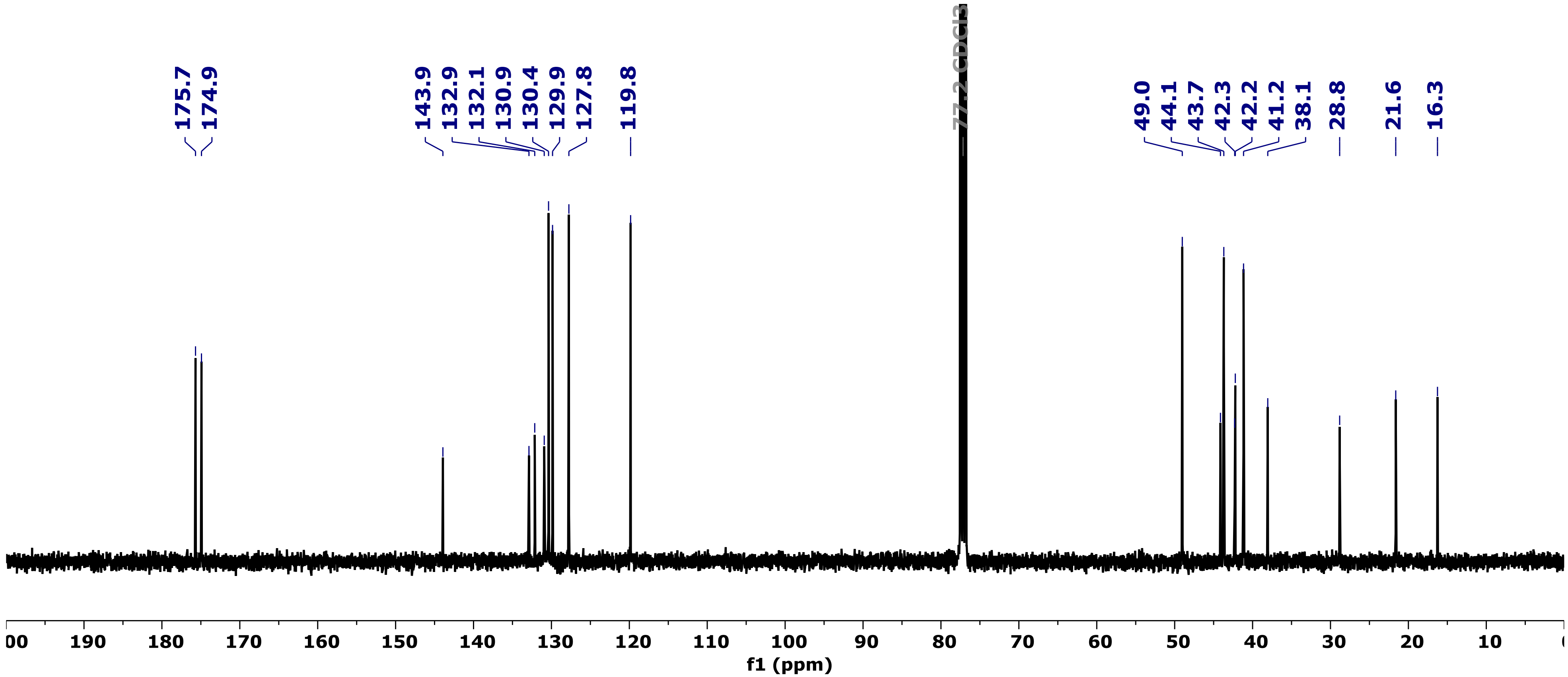

2D NMR HSQC

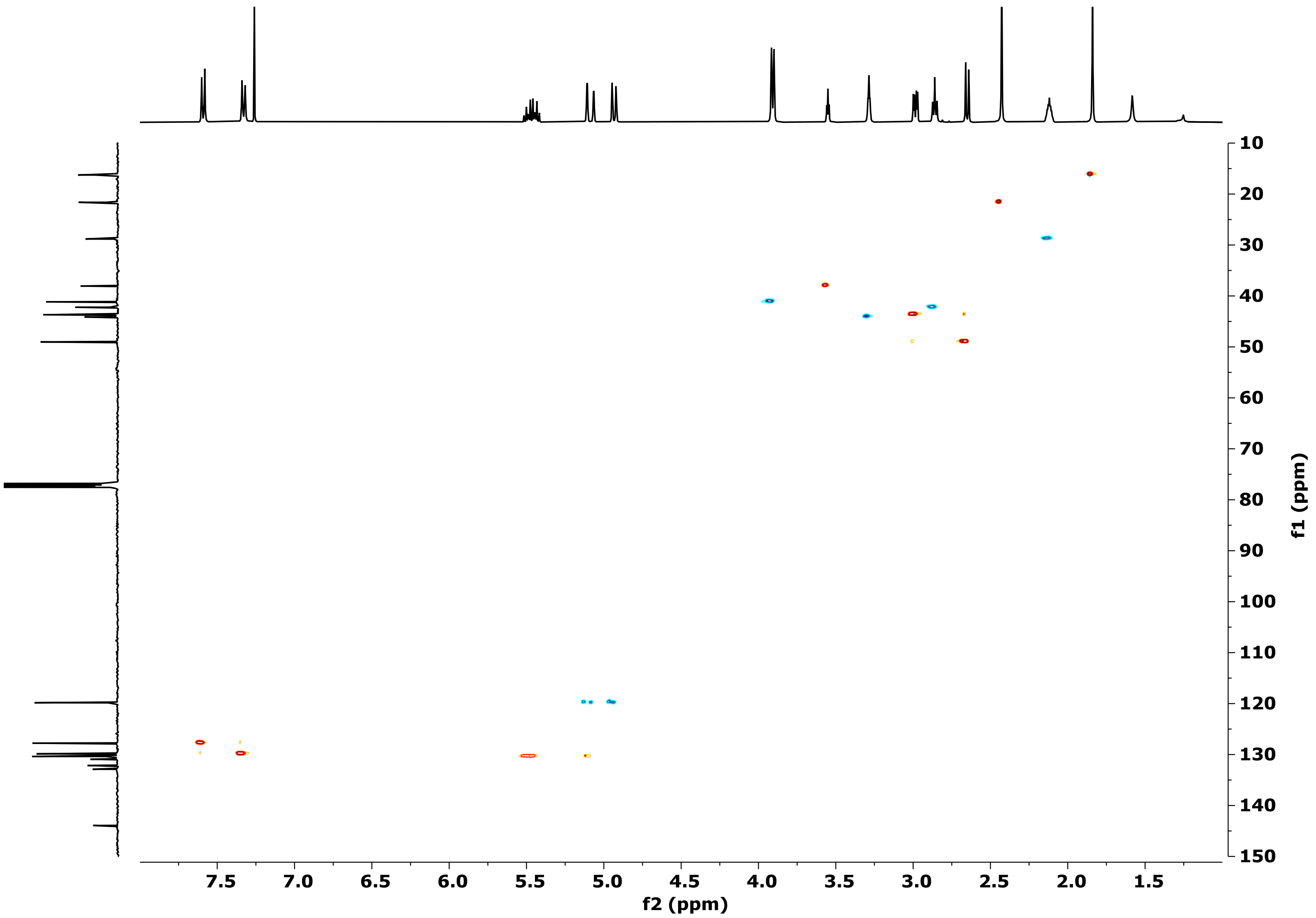

10
20
30
40
50
60
70
80
90
100
110
120
130
140
150
f1 (ppm)
7.5
7.0
6.5
6.0
5.5
5.0
4.5
4.0
3.5
3.0
2.5
2.0
1.5
f2 (ppm)

2D NMR COSY

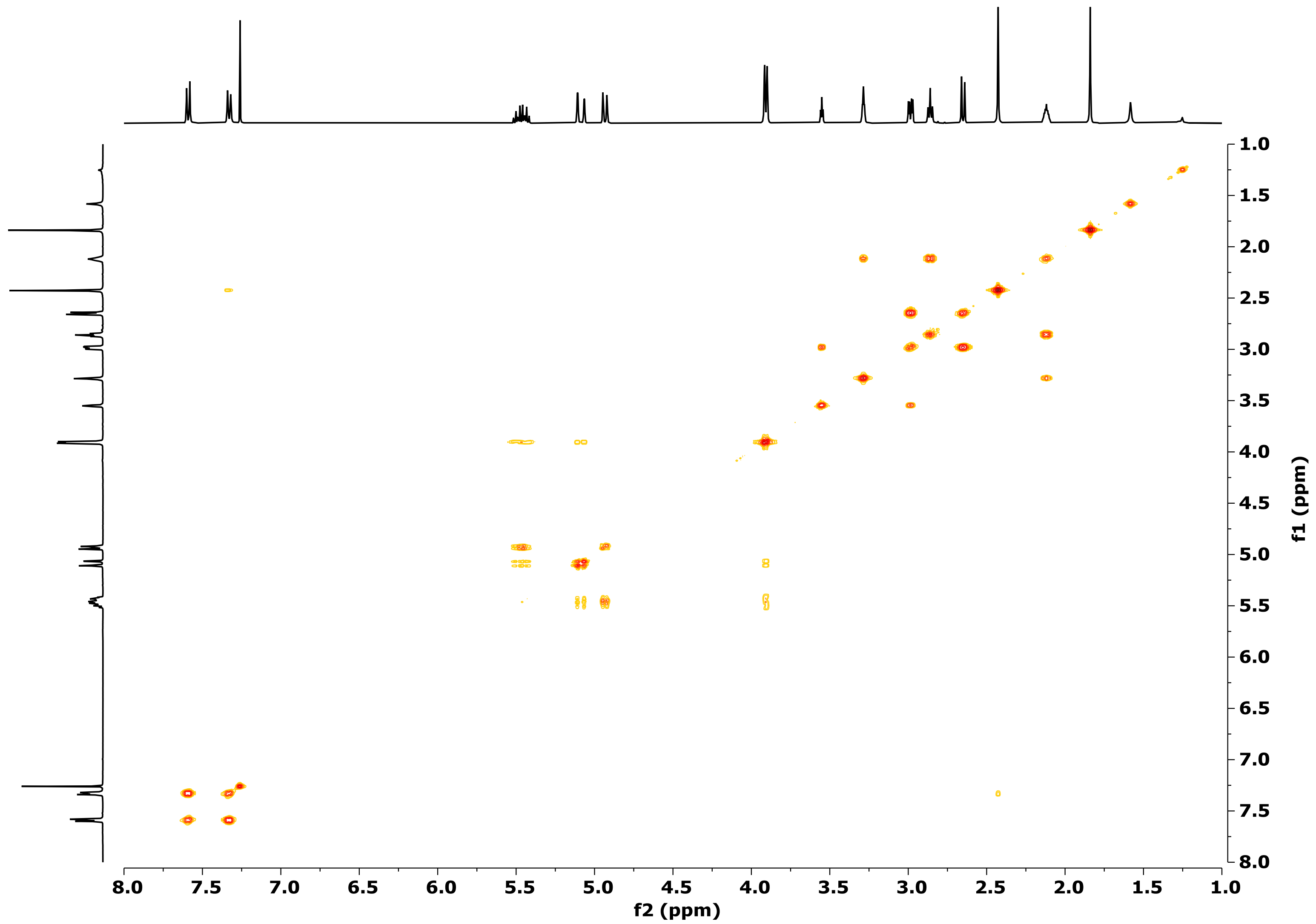

Compound 3g

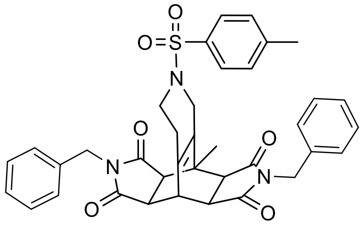

^{1}H NMR (400 MHz, $CDCl_3$)

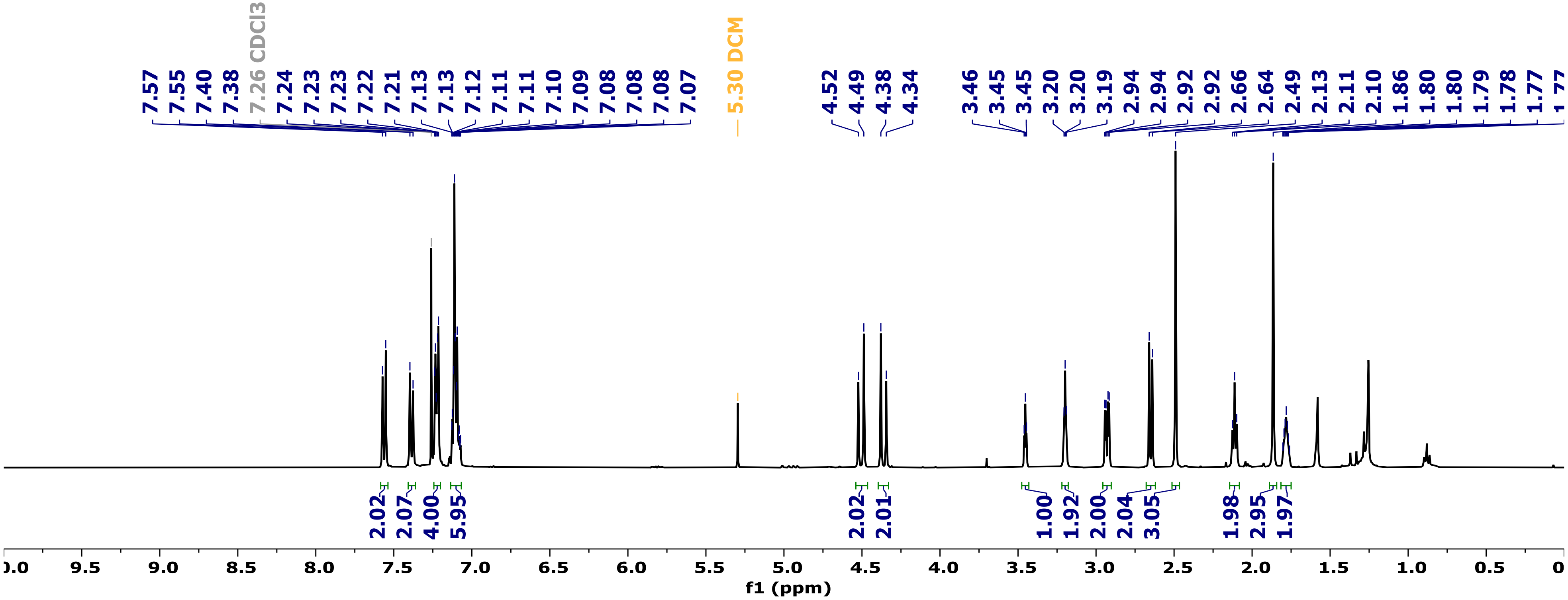

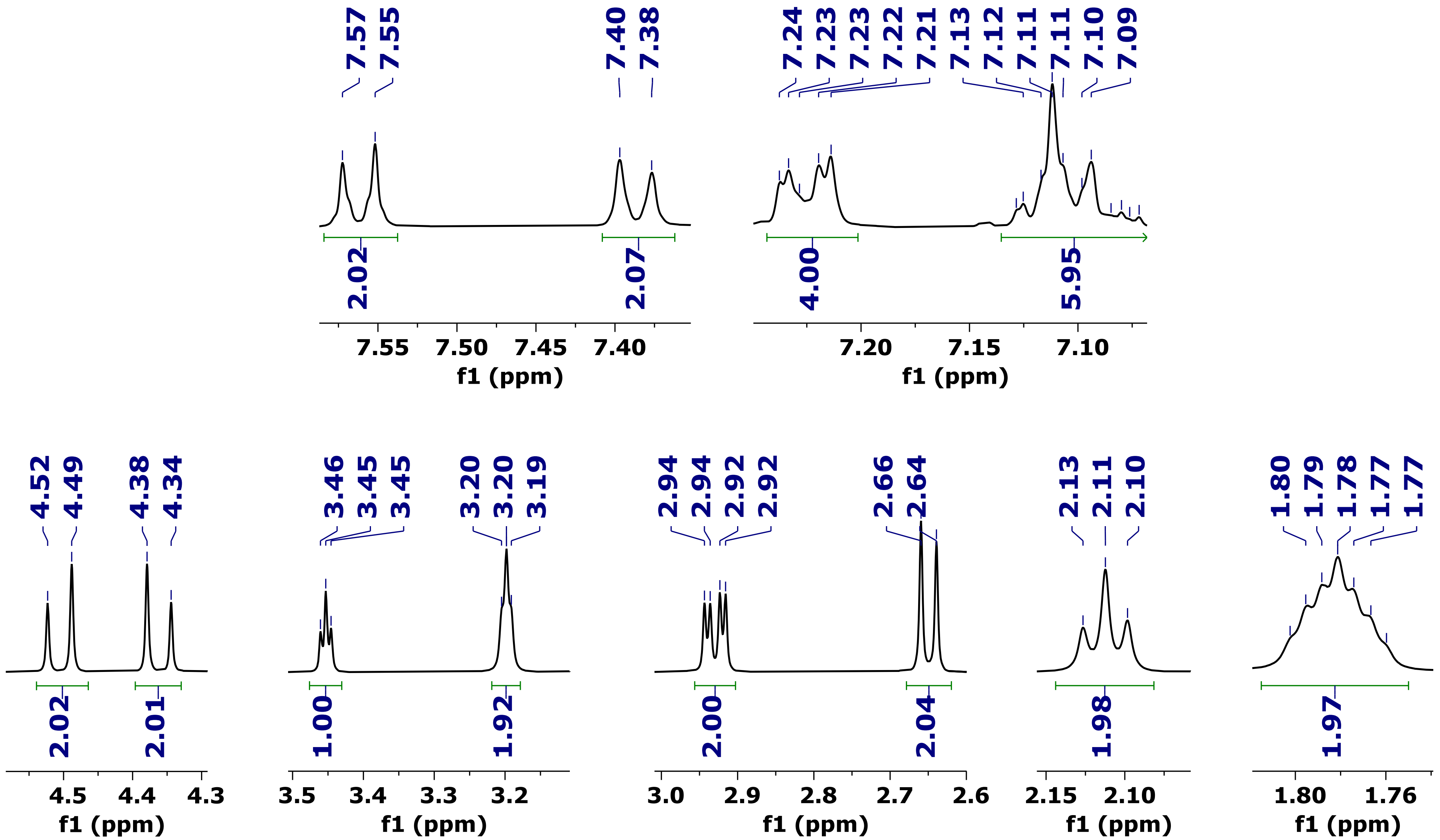

7.57
7.55
7.40
7.38
2.02
2.07
7.55 7.50 7.45 7.40
f1 (ppm)
7.24
7.23
7.23
7.22
7.21
7.13
7.12
7.11
7.11
7.10
7.09
4.00
5.95
7.20 7.15 7.10
f1 (ppm)
4.52
4.49
4.38
4.34
2.02
2.01
4.5 4.4 4.3
f1 (ppm)
3.46
3.45
3.45
3.20
3.20
3.19
1.00
1.92
3.5 3.4 3.3 3.2
f1 (ppm)
2.94
2.94
2.92
2.92
2.66
2.64
2.00
2.04
3.0 2.9 2.8 2.7 2.6
f1 (ppm)
2.13
2.11
2.10
1.98
2.15 2.10
f1 (ppm)
1.80
1.79
1.78
1.77
1.77
1.97
1.80 1.76
f1 (ppm)

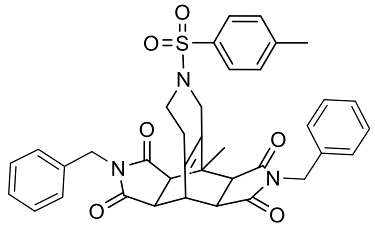

^{13}C NMR (101 MHz, $CDCl_3$)

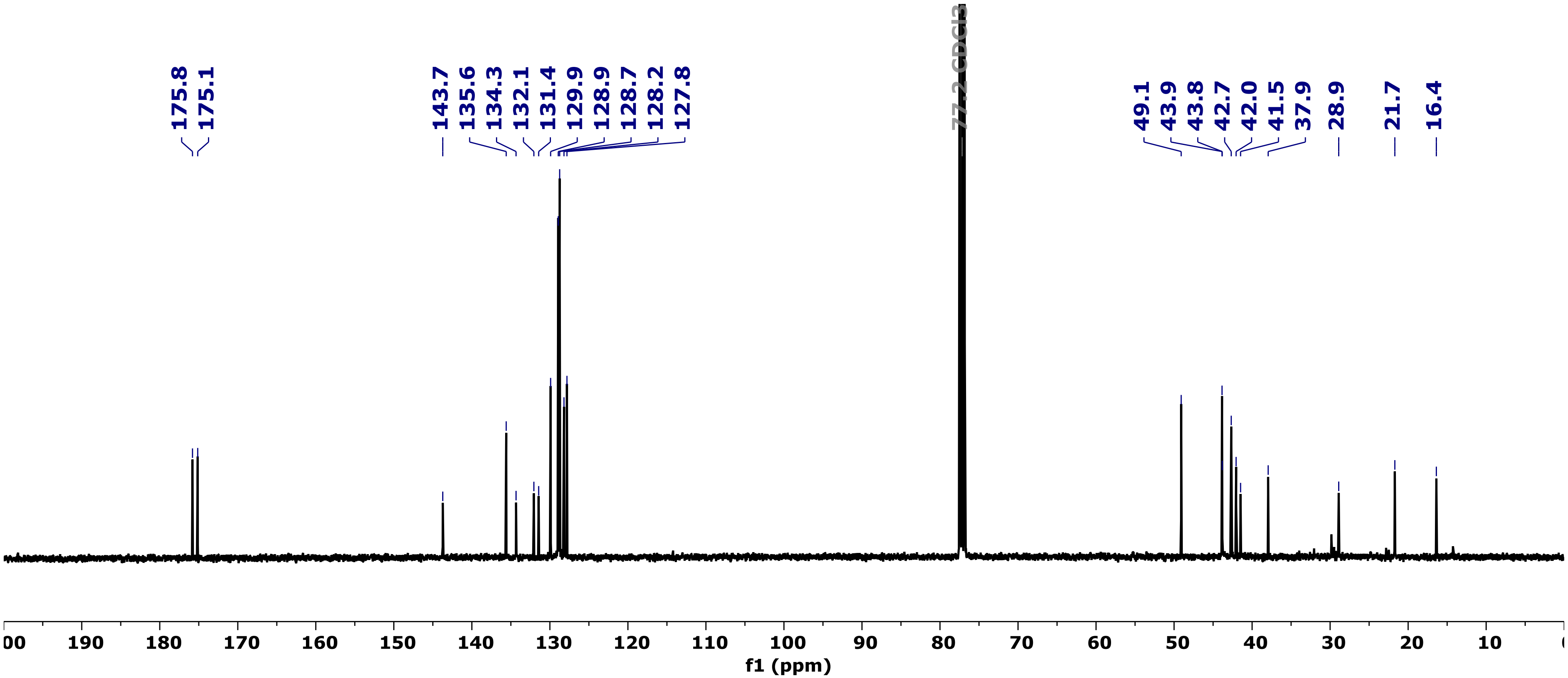

2D NMR HSQC

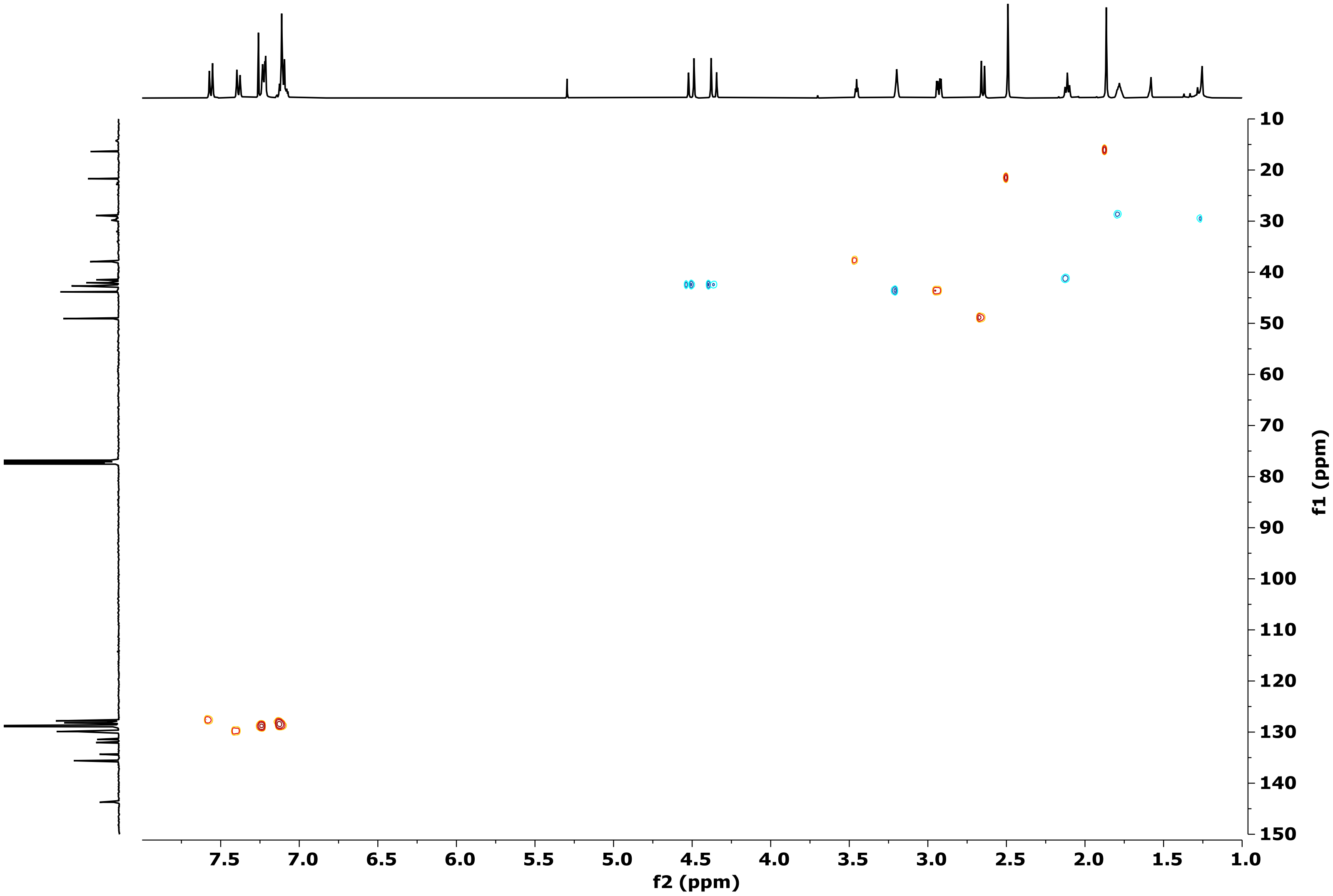

7.5
7.0
6.5
6.0
5.5
5.0
4.5
4.0
3.5
3.0
2.5
2.0
1.5
1.0
f2 (ppm)
10
20
30
40
50
60
70
80
90
100
110
120
130
140
150
f1 (ppm)

Compound 3h

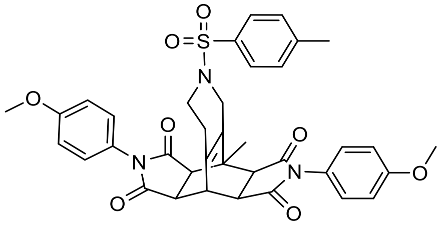

^{1}H NMR (400 MHz, $CDCl_3$)

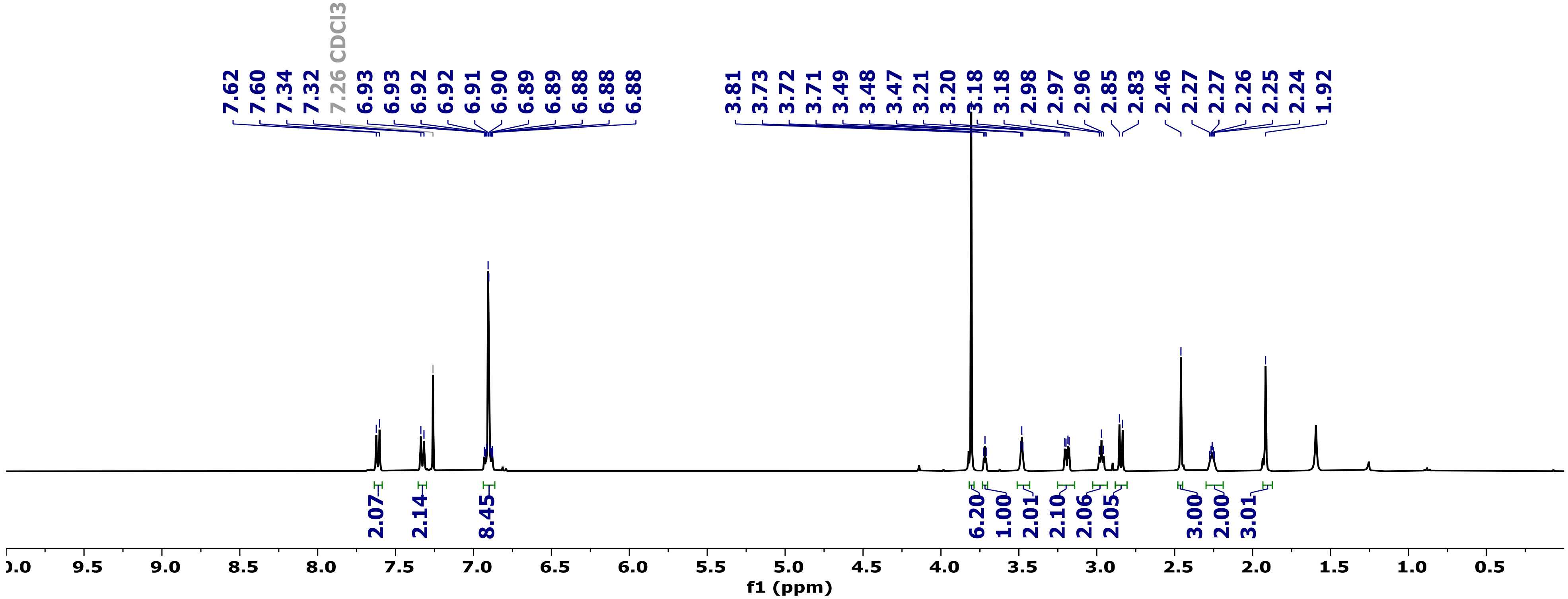

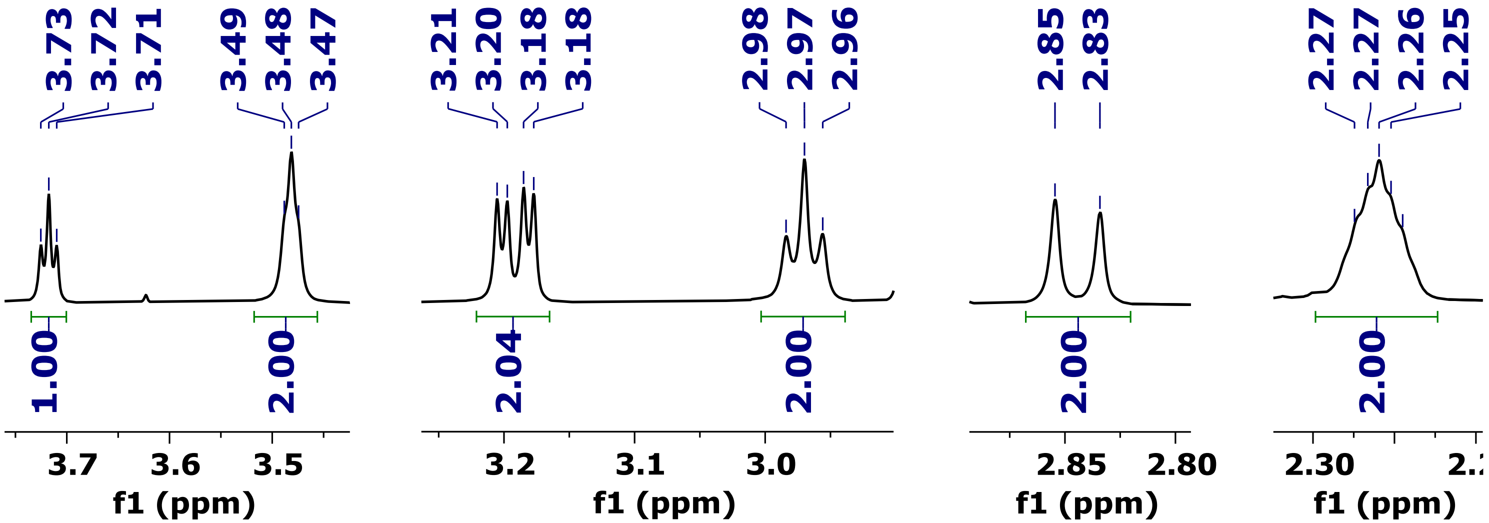
3.73
3.72
3.71
3.49
3.48
3.47
3.21
3.20
3.18
3.18
2.98
2.97
2.96
2.85
2.83
2.27
2.27
2.26
2.25
1.00
2.00
2.04
2.00
2.00
2.00
3.7
3.6
3.5
3.2
3.1
3.0
2.85
2.80
2.30
f1 (ppm)
f1 (ppm)
f1 (ppm)
f1 (ppm)

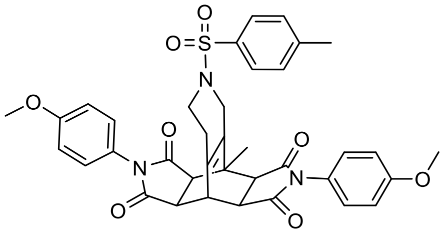

^{13}C NMR (101 MHz, $CDCl_3$)

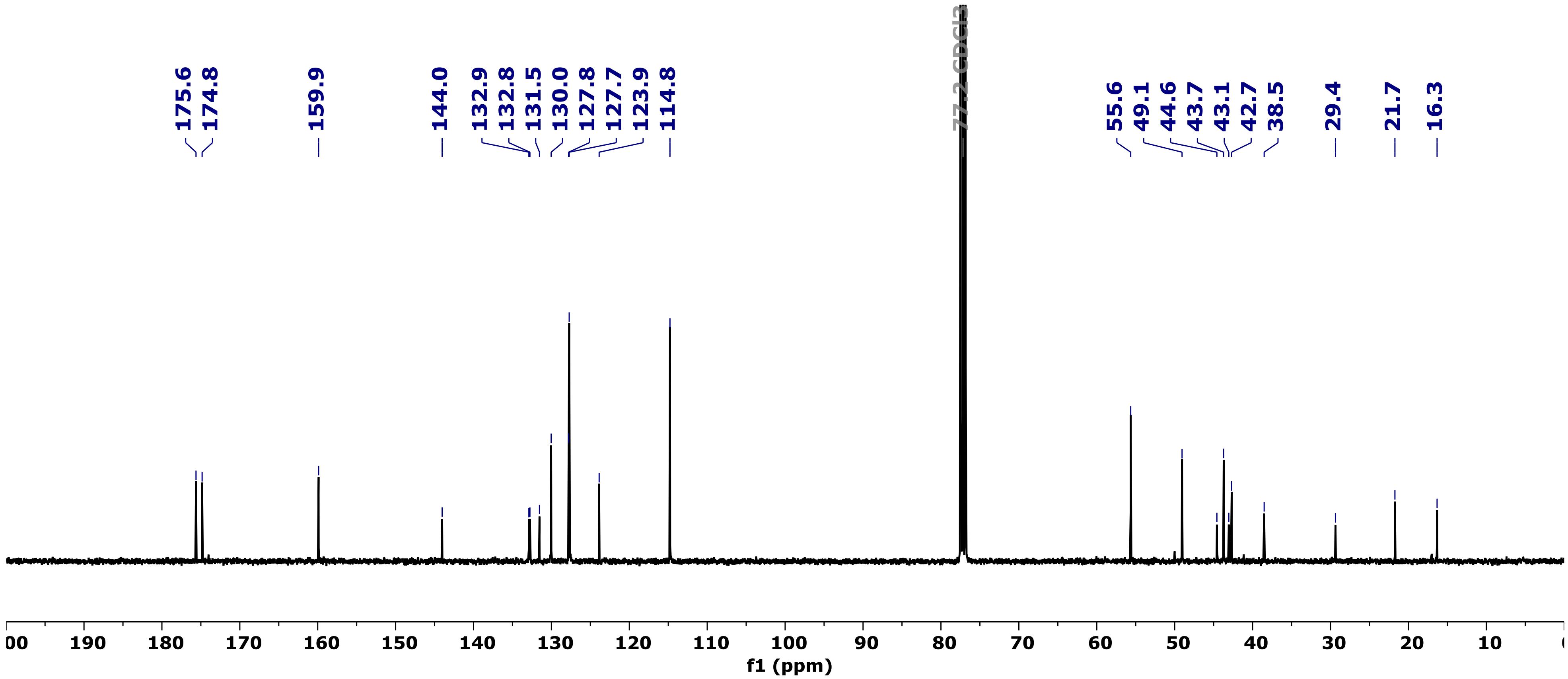

2D NMR HSQC

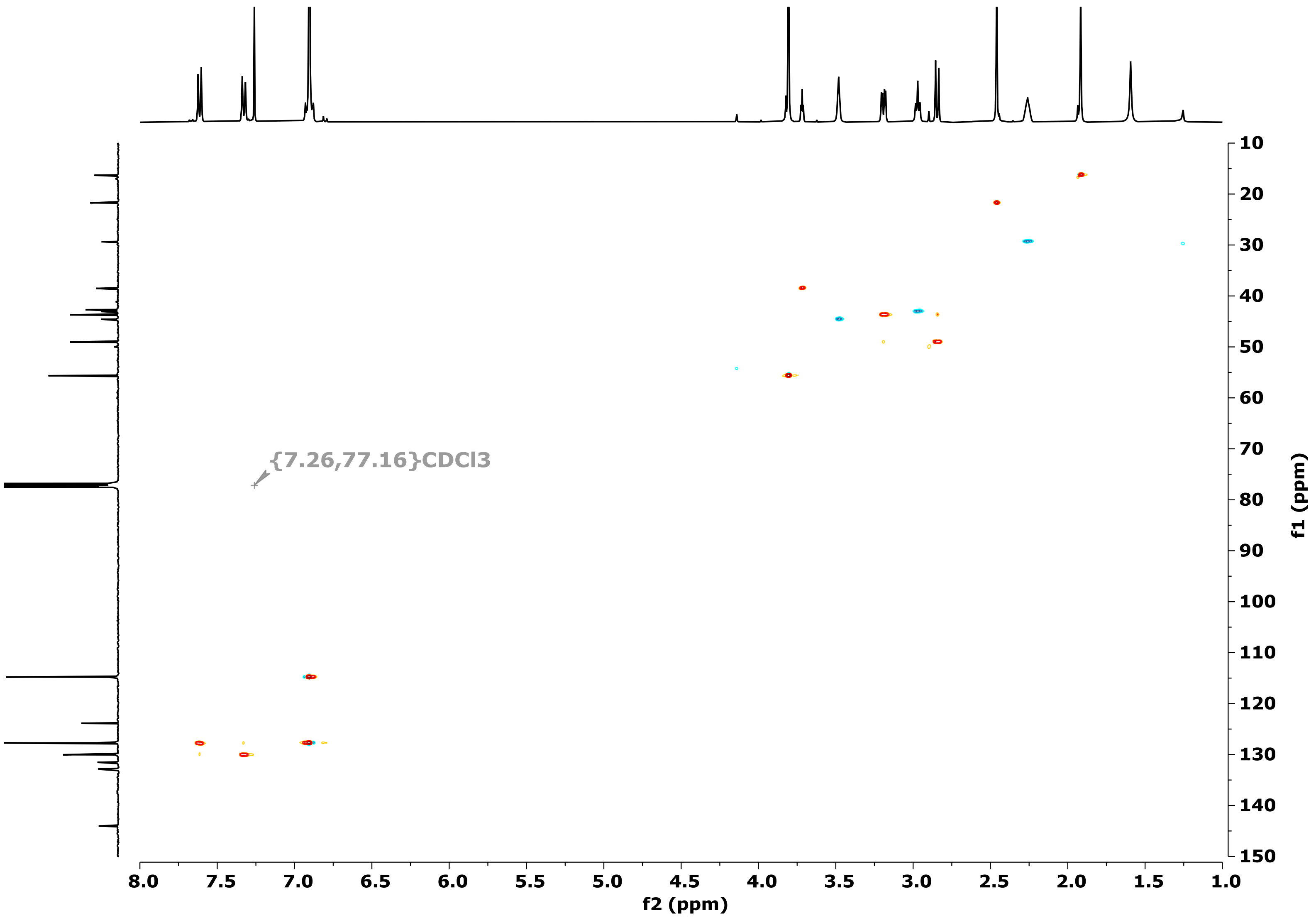

{7.26,77.16}CDCl3
f2 (ppm)
f1 (ppm)
8.0
7.5
7.0
6.5
6.0
5.5
5.0
4.5
4.0
3.5
3.0
2.5
2.0
1.5
1.0
10
20
30
40
50
60
70
80
90
100
110
120
130
140
150

Compound 3i

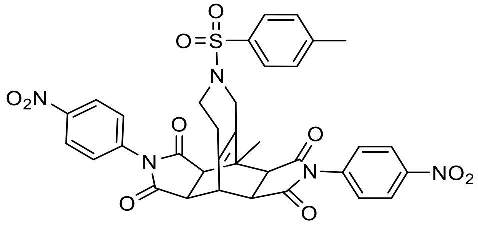

^{1}H NMR (400 MHz, $CDCl_3$)

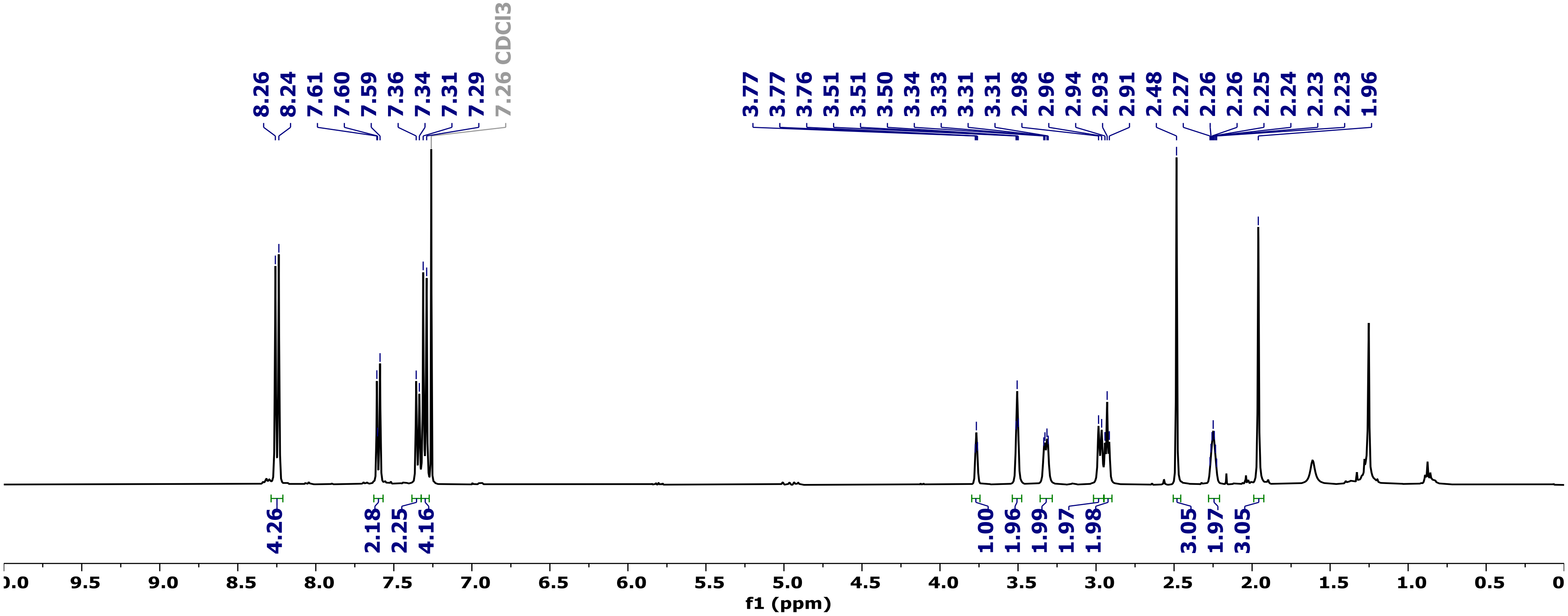

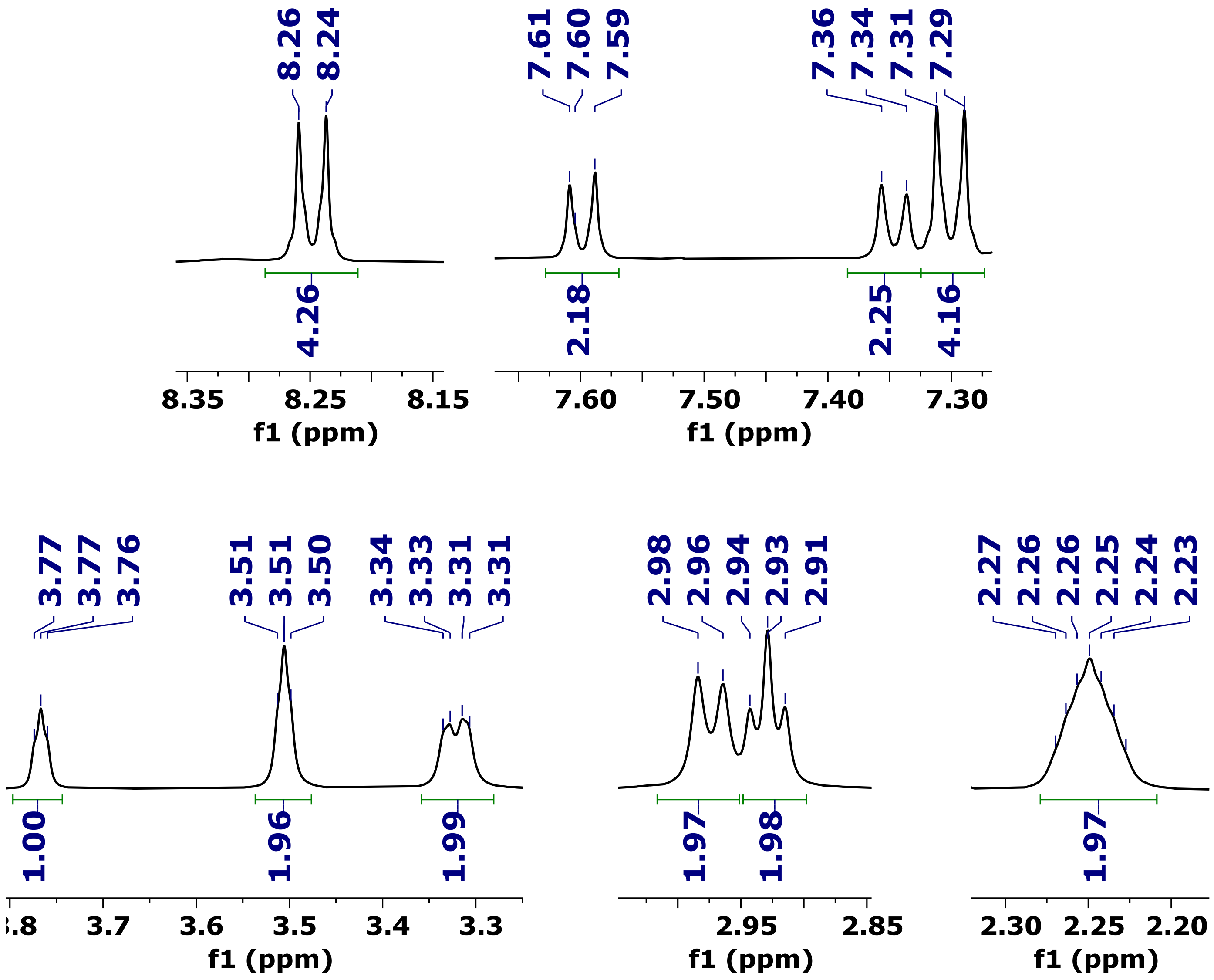
8.26
8.24
4.26
8.35
8.25
8.15
f1 (ppm)
7.61
7.60
7.59
7.36
7.34
7.31
7.29
2.18
2.25
4.16
7.60
7.50
7.40
7.30
f1 (ppm)
3.77
3.77
3.76
3.51
3.51
3.50
3.34
3.33
3.31
3.31
1.00
1.96
1.99
3.7
3.6
3.5
3.4
3.3
f1 (ppm)
2.98
2.96
2.94
2.93
2.91
1.97
1.98
2.95
2.85
f1 (ppm)
2.27
2.26
2.26
2.25
2.24
2.23
1.97
2.30
2.25
2.20
f1 (ppm)

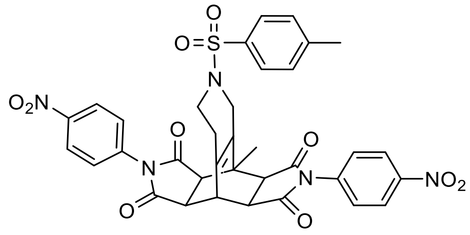

^{13}C NMR (101 MHz, $CDCl_3$)

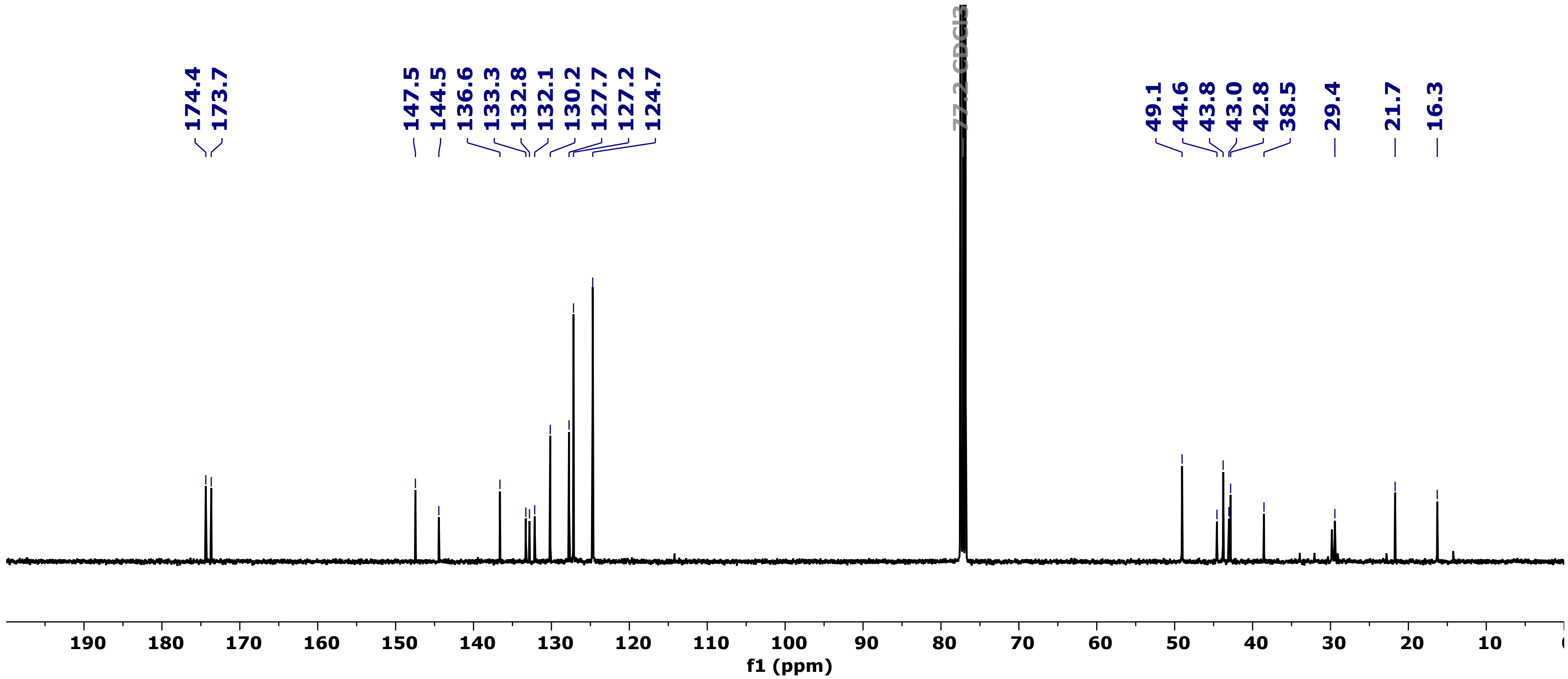

2D NMR HSQC

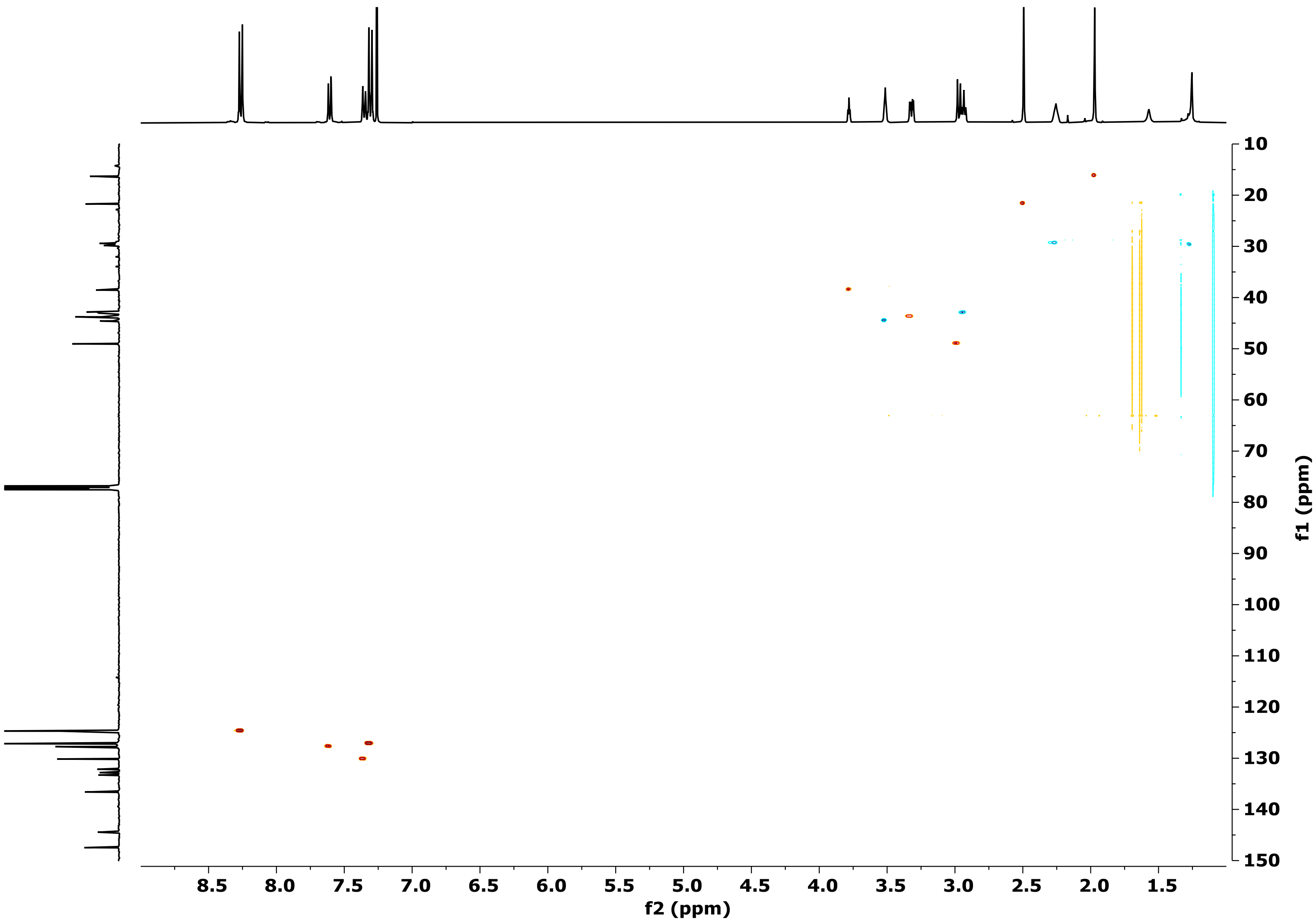

f1 (ppm)
10
20
30
40
50
60
70
80
90
100
110
120
130
140
150
8.5
8.0
7.5
7.0
6.5
6.0
5.5
5.0
4.5
4.0
3.5
3.0
2.5
2.0
1.5
f2 (ppm)

Compound 3j

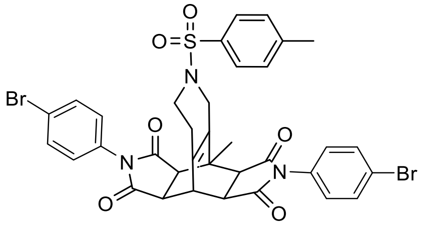

^{1}H NMR (400 MHz, $CDCl_3$)

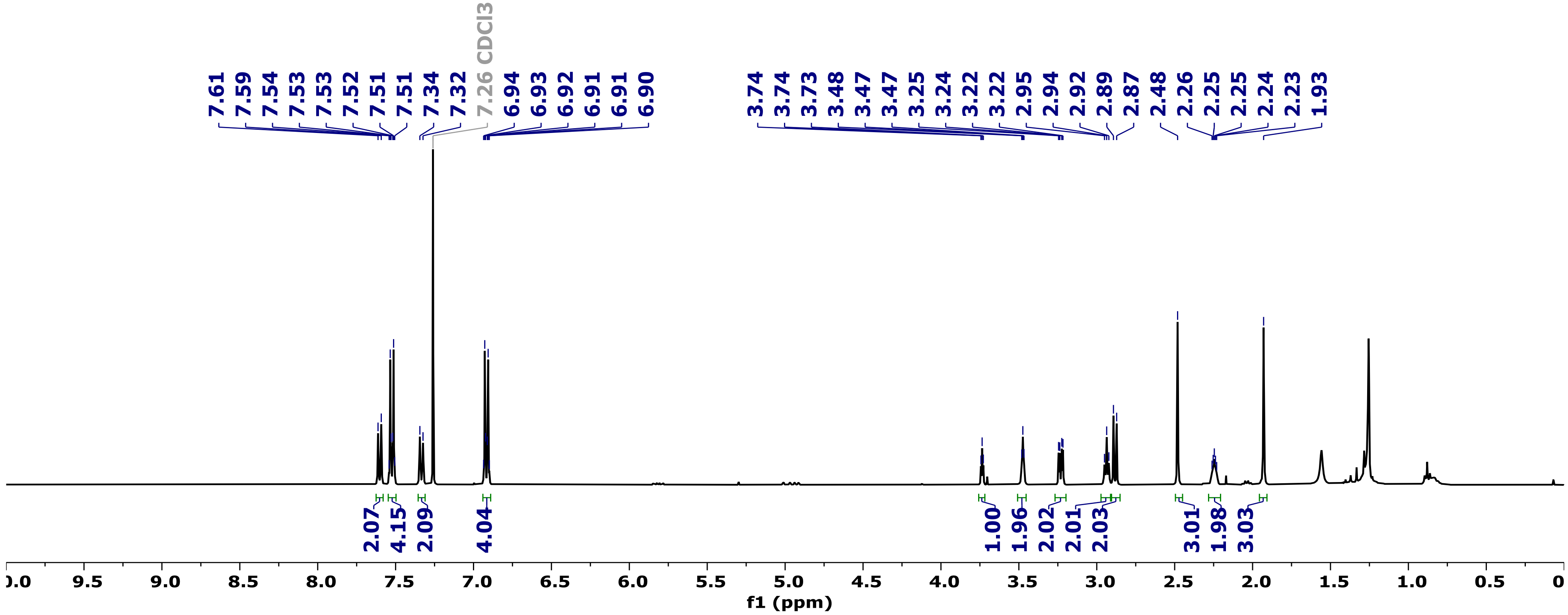

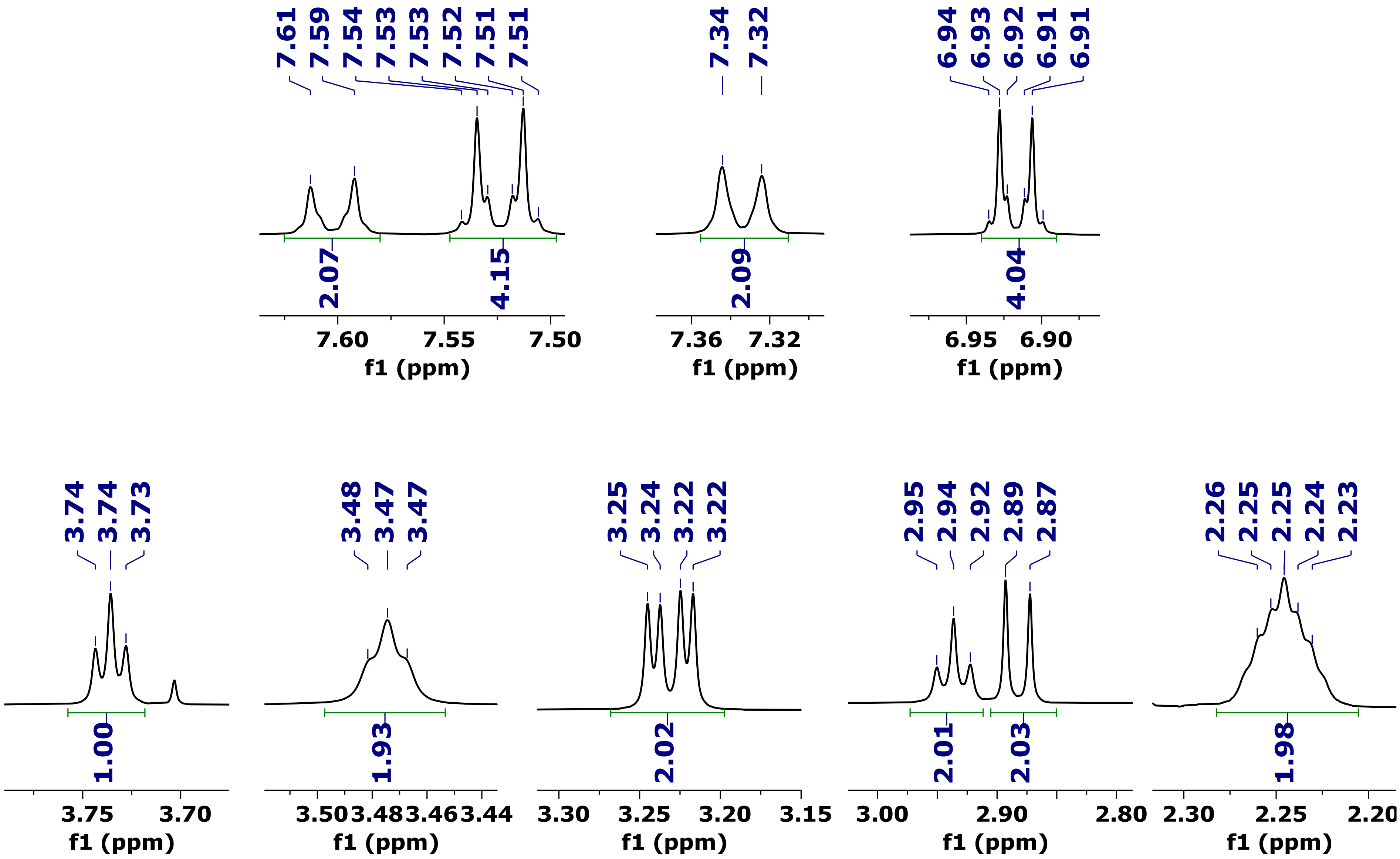
7.61
7.59
7.54
7.53
7.53
7.52
7.51
7.51
2.07
4.15
7.60
7.55
7.50
f1 (ppm)
7.34
7.32
2.09
7.36
7.32
f1 (ppm)
6.94
6.93
6.92
6.91
6.91
4.04
6.95
6.90
f1 (ppm)
3.74
3.74
3.73
1.00
3.75
3.70
f1 (ppm)
3.48
3.47
3.47
1.93
3.50
3.48
3.46
3.44
f1 (ppm)
3.25
3.24
3.22
3.22
2.02
3.30
3.25
3.20
3.15
f1 (ppm)
2.95
2.94
2.92
2.89
2.87
2.01
2.03
3.00
2.90
2.80
f1 (ppm)
2.26
2.25
2.25
2.24
2.23
1.98
2.30
2.25
2.20
f1 (ppm)

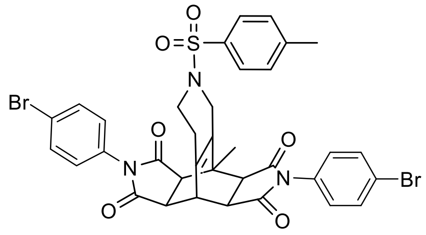

^{13}C NMR (101 MHz, $CDCl_3$)

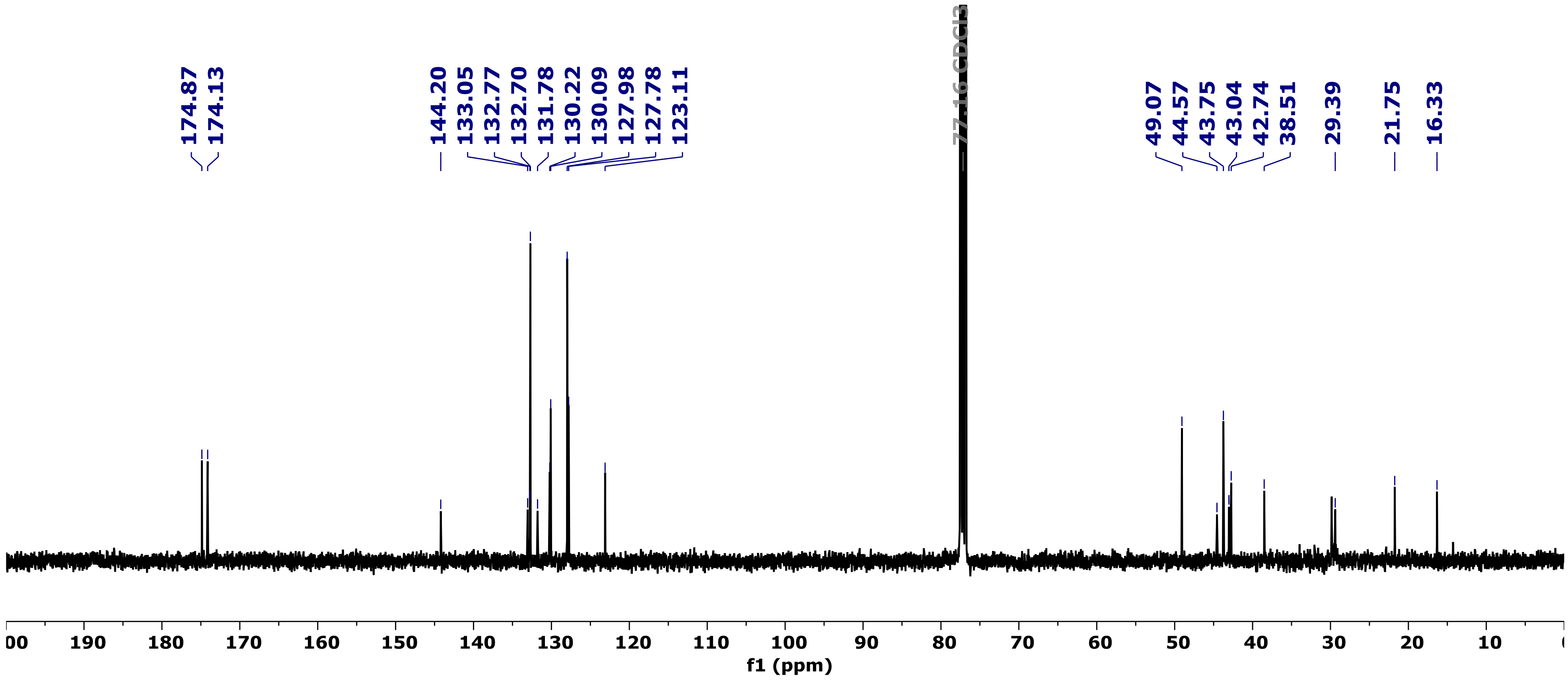

2D NMR HSQC

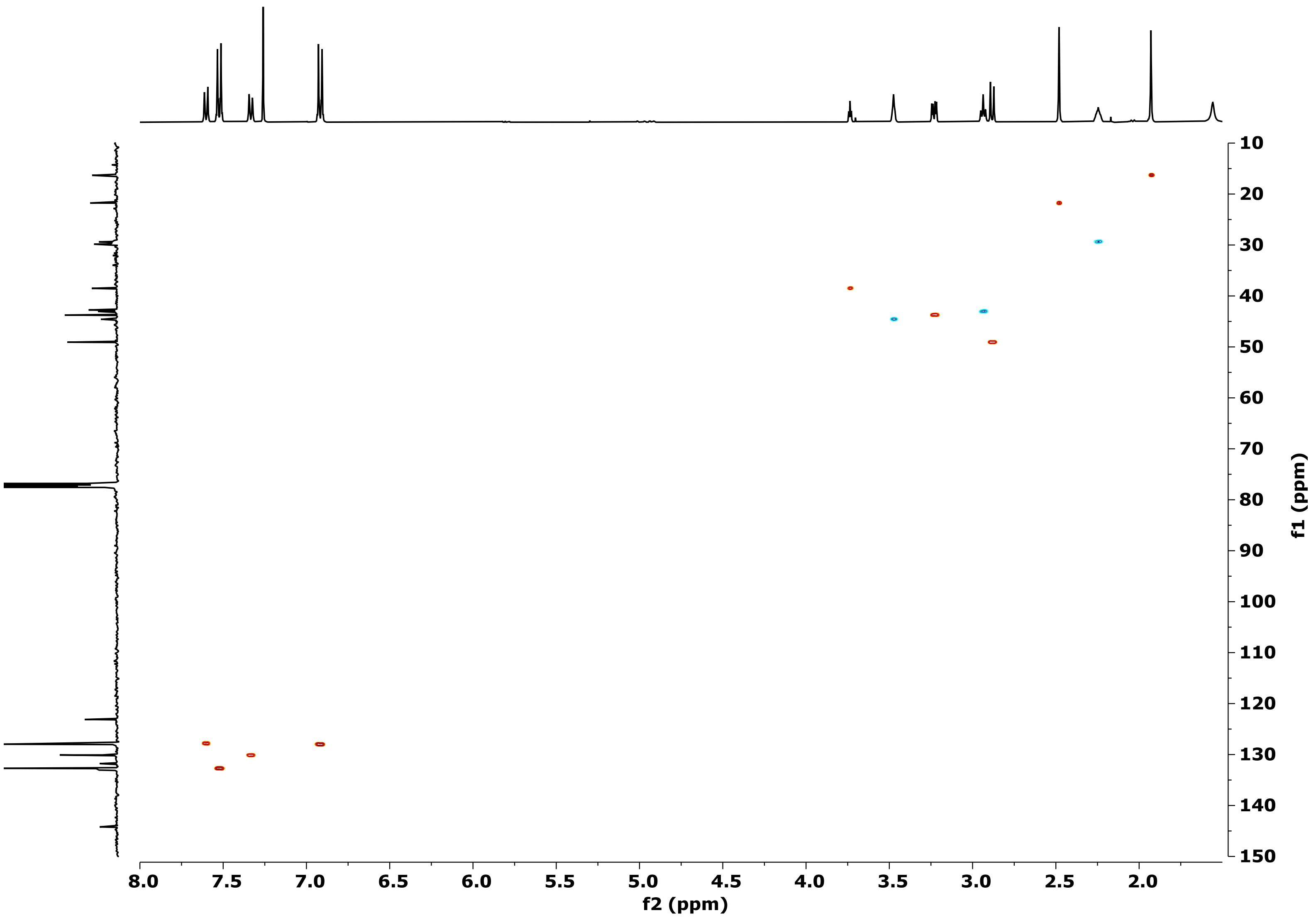

10
20
30
40
50
60
70
80
90
100
110
120
130
140
150
f1 (ppm)
8.0
7.5
7.0
6.5
6.0
5.5
5.0
4.5
4.0
3.5
3.0
2.5
2.0
f2 (ppm)

Compound 3k

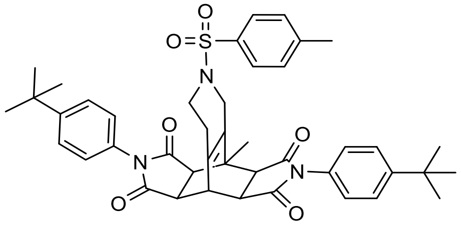

^{1}H NMR (400 MHz, CDCl$_3$)

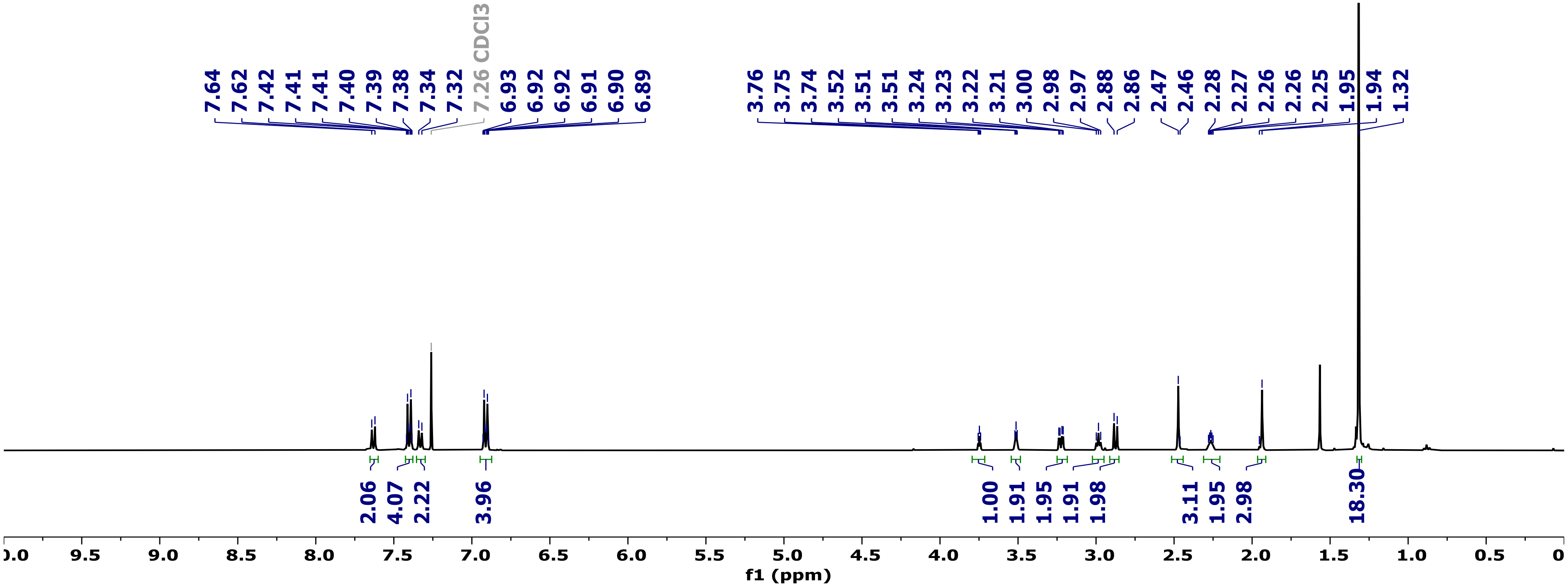

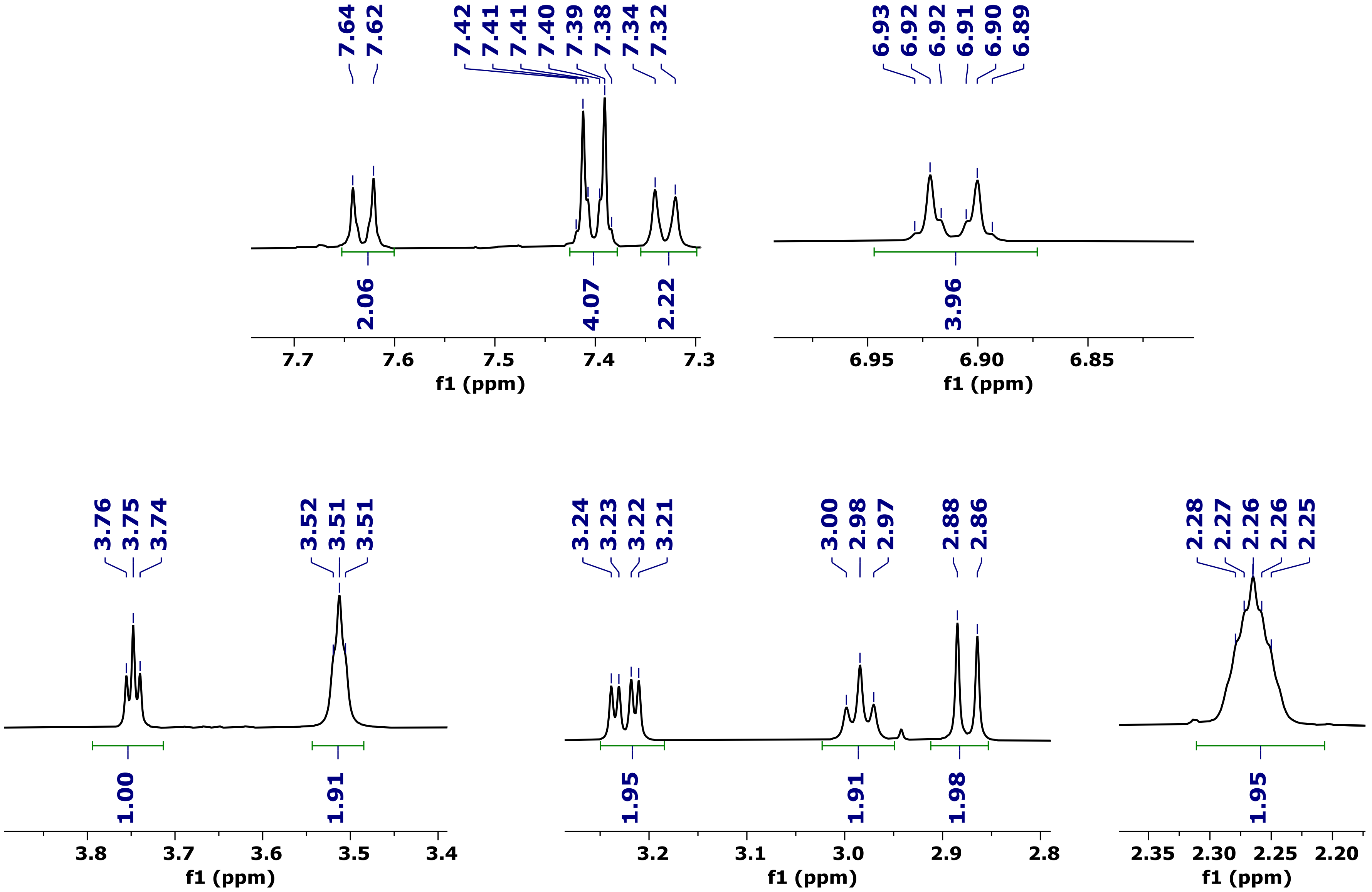
7.64
7.62
7.42
7.41
7.41
7.40
7.39
7.38
7.34
7.32
2.06
4.07
2.22
7.7
7.6
7.5
7.4
7.3
f1 (ppm)
6.93
6.92
6.92
6.91
6.90
6.89
3.96
6.95
6.90
6.85
f1 (ppm)
3.76
3.75
3.74
3.52
3.51
3.51
1.00
1.91
3.8
3.7
3.6
3.5
3.4
f1 (ppm)
3.24
3.23
3.22
3.21
3.00
2.98
2.97
2.88
2.86
1.95
1.91
1.98
3.2
3.1
3.0
2.9
2.8
f1 (ppm)
2.28
2.27
2.26
2.26
2.25
1.95
2.35
2.30
2.25
2.20
f1 (ppm)

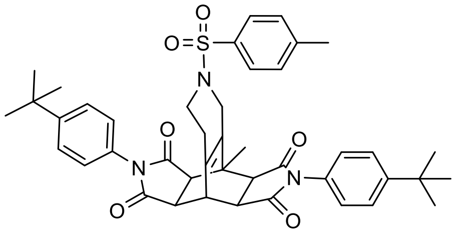

^{13}C NMR (101 MHz, CDCl$_3$)

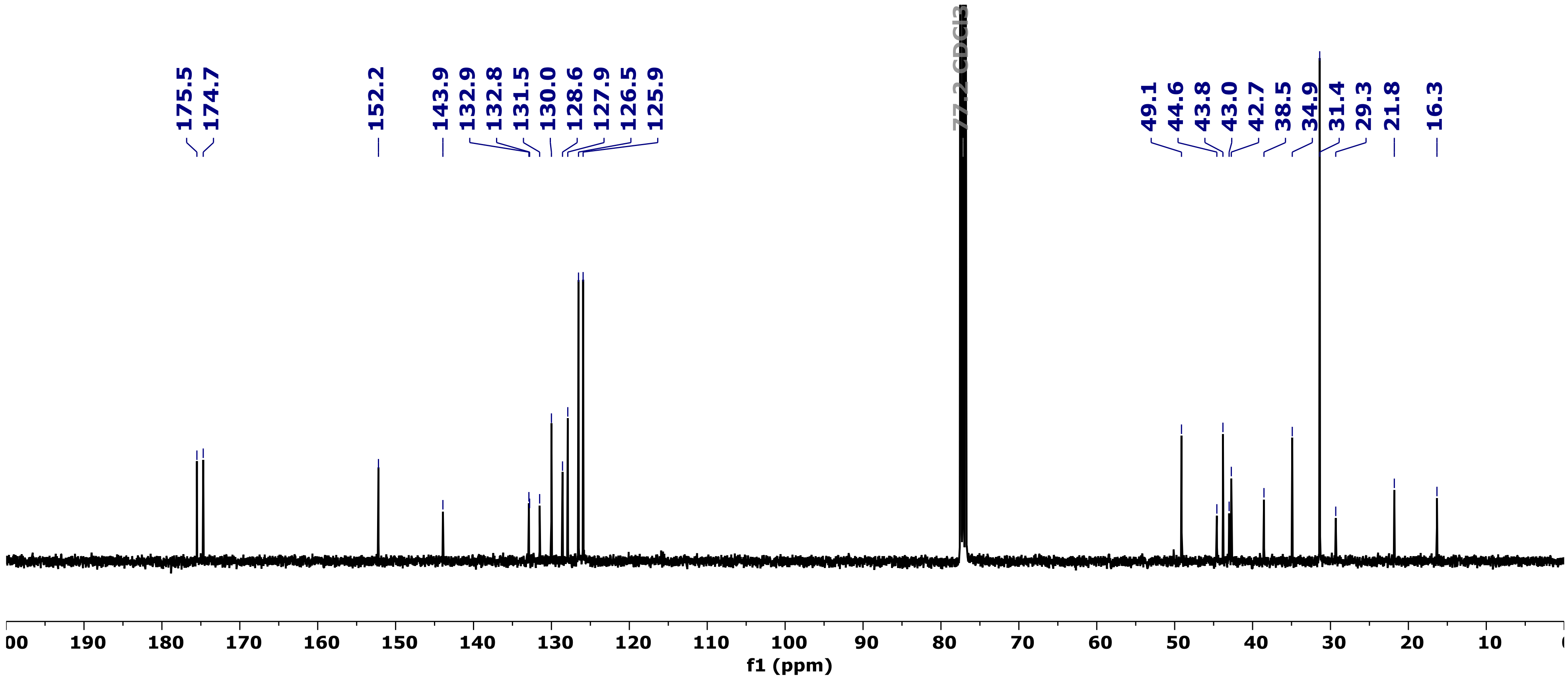

2D NMR HSQC

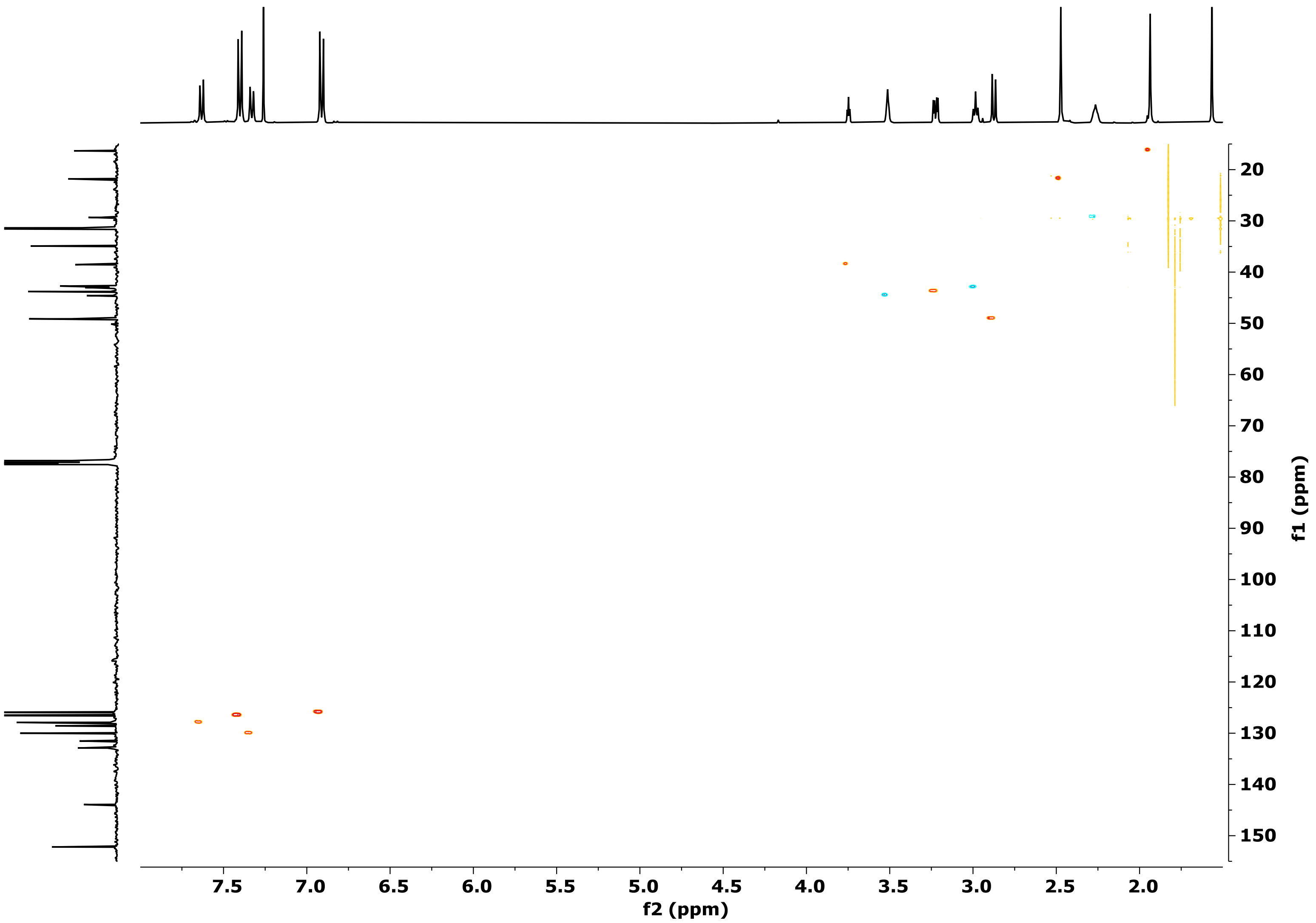

2D NMR COSY

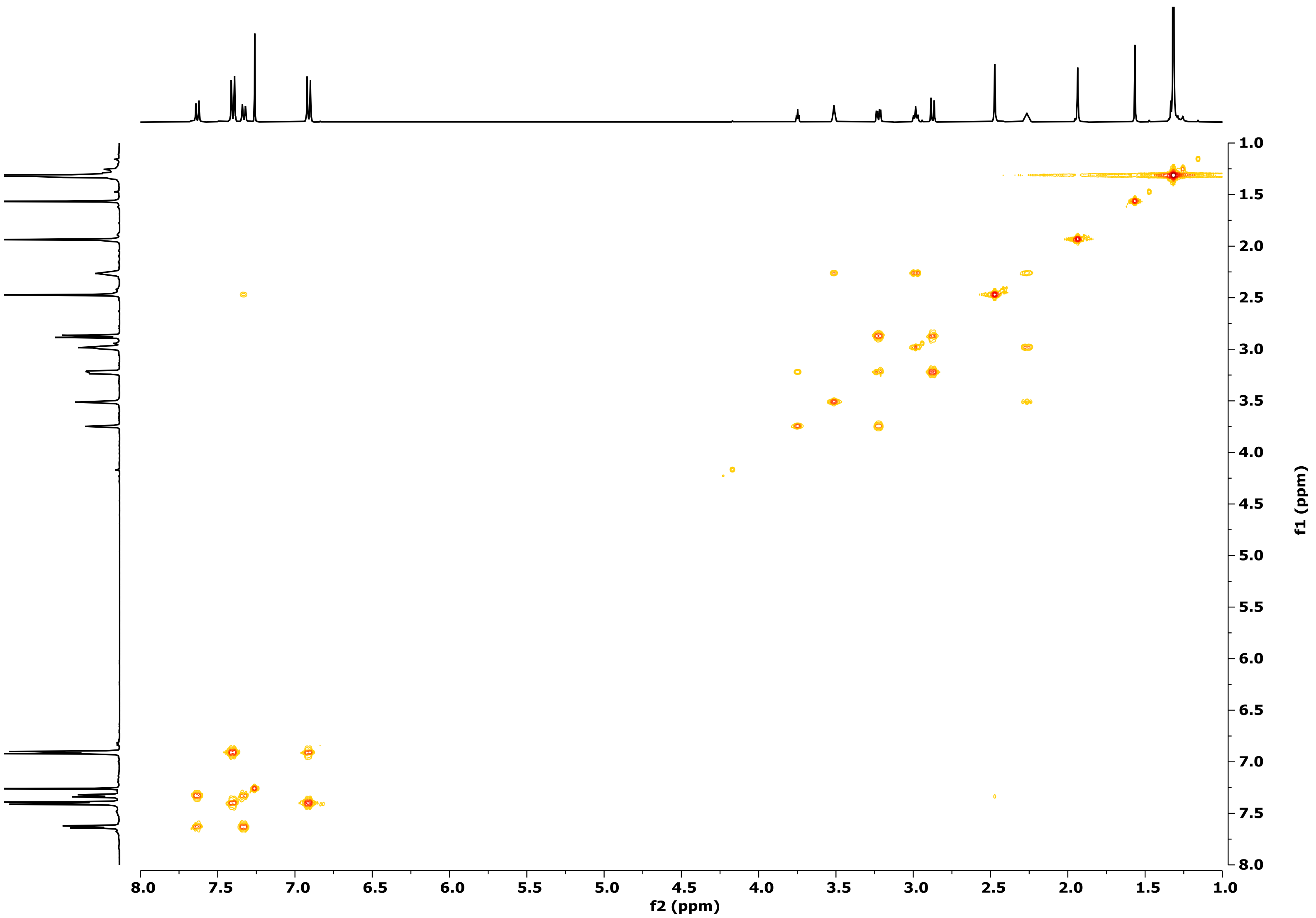

Compound 3l

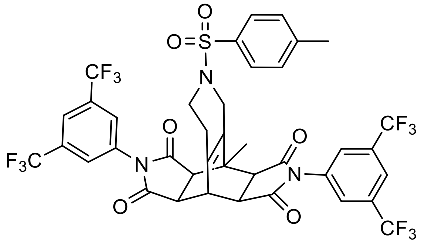

^{1}H NMR (400 MHz, DMSO)

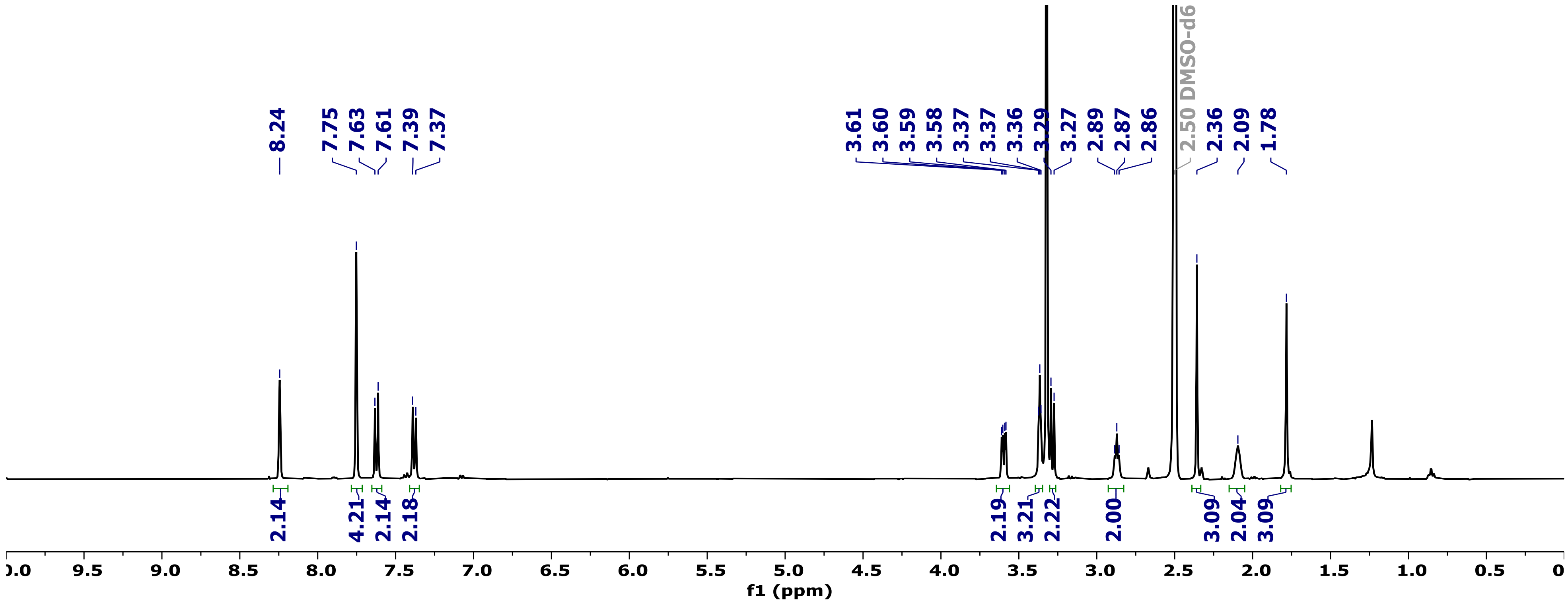

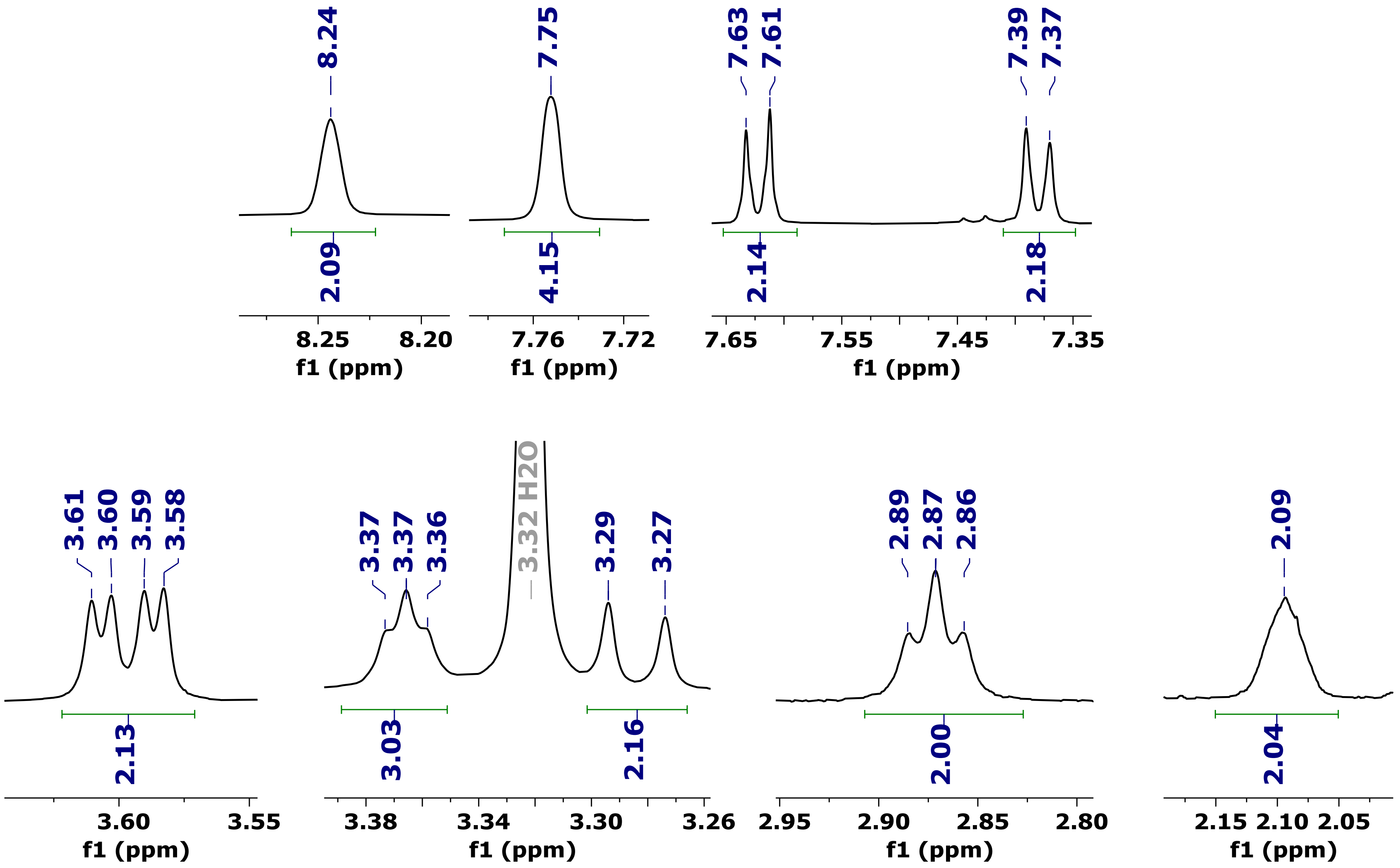
8.24
2.09
8.25
8.20
f1 (ppm)
7.75
4.15
7.76
7.72
f1 (ppm)
7.63
7.61
7.39
7.37
2.14
2.18
7.65
7.55
7.45
7.35
f1 (ppm)
3.61
3.60
3.59
3.58
2.13
3.60
3.55
f1 (ppm)
3.37
3.37
3.36
3.32 H2O
3.29
3.27
3.03
2.16
3.38
3.34
3.30
3.26
f1 (ppm)
2.89
2.87
2.86
2.00
2.95
2.90
2.85
2.80
f1 (ppm)
2.09
2.04
2.15
2.10
2.05
f1 (ppm)

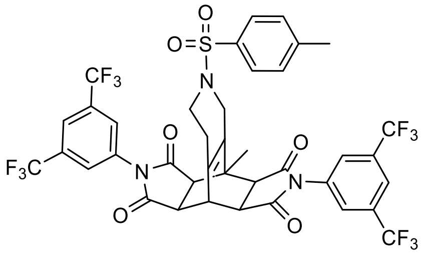

^{13}C NMR (101 MHz, DMSO)

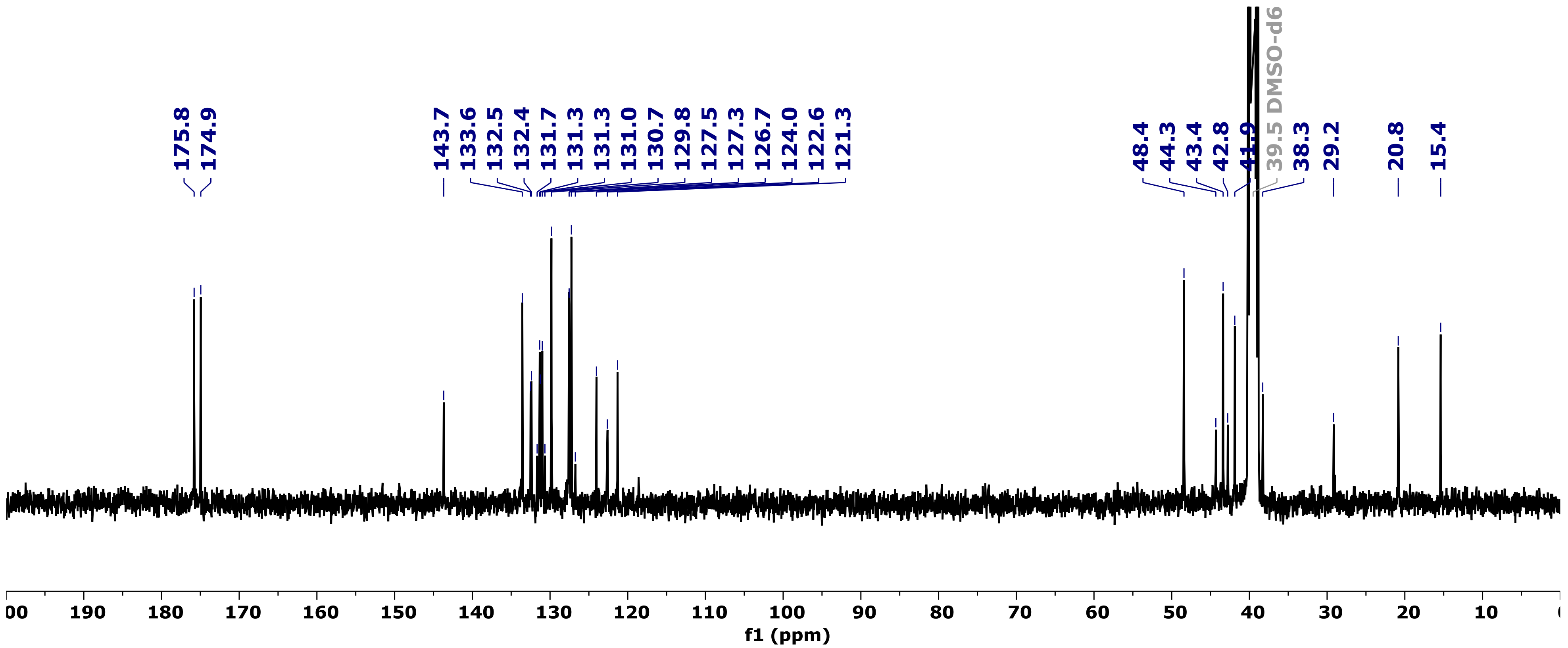

^{19}F NMR (377 MHz, DMSO)

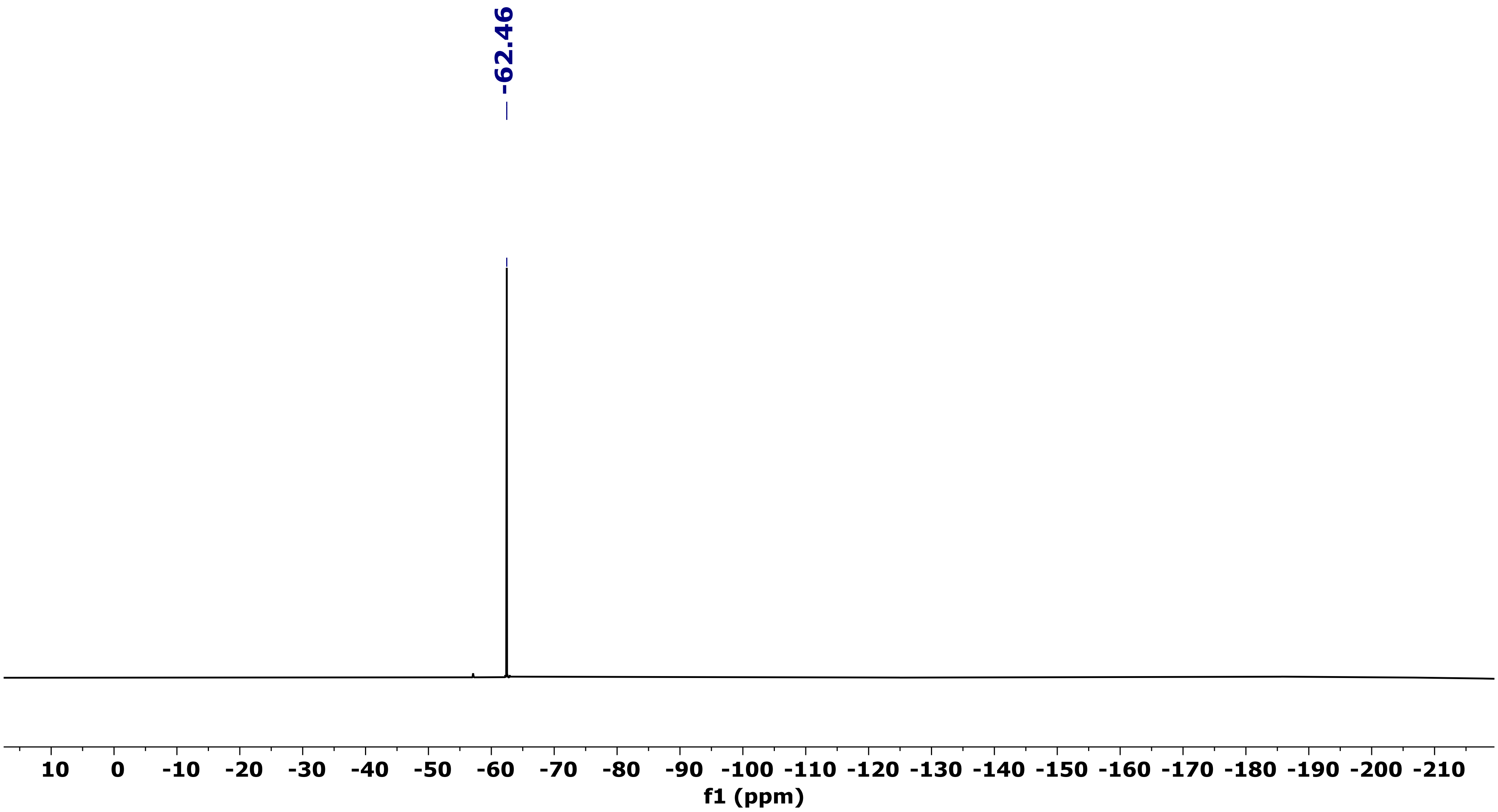

2D NMR HSQC

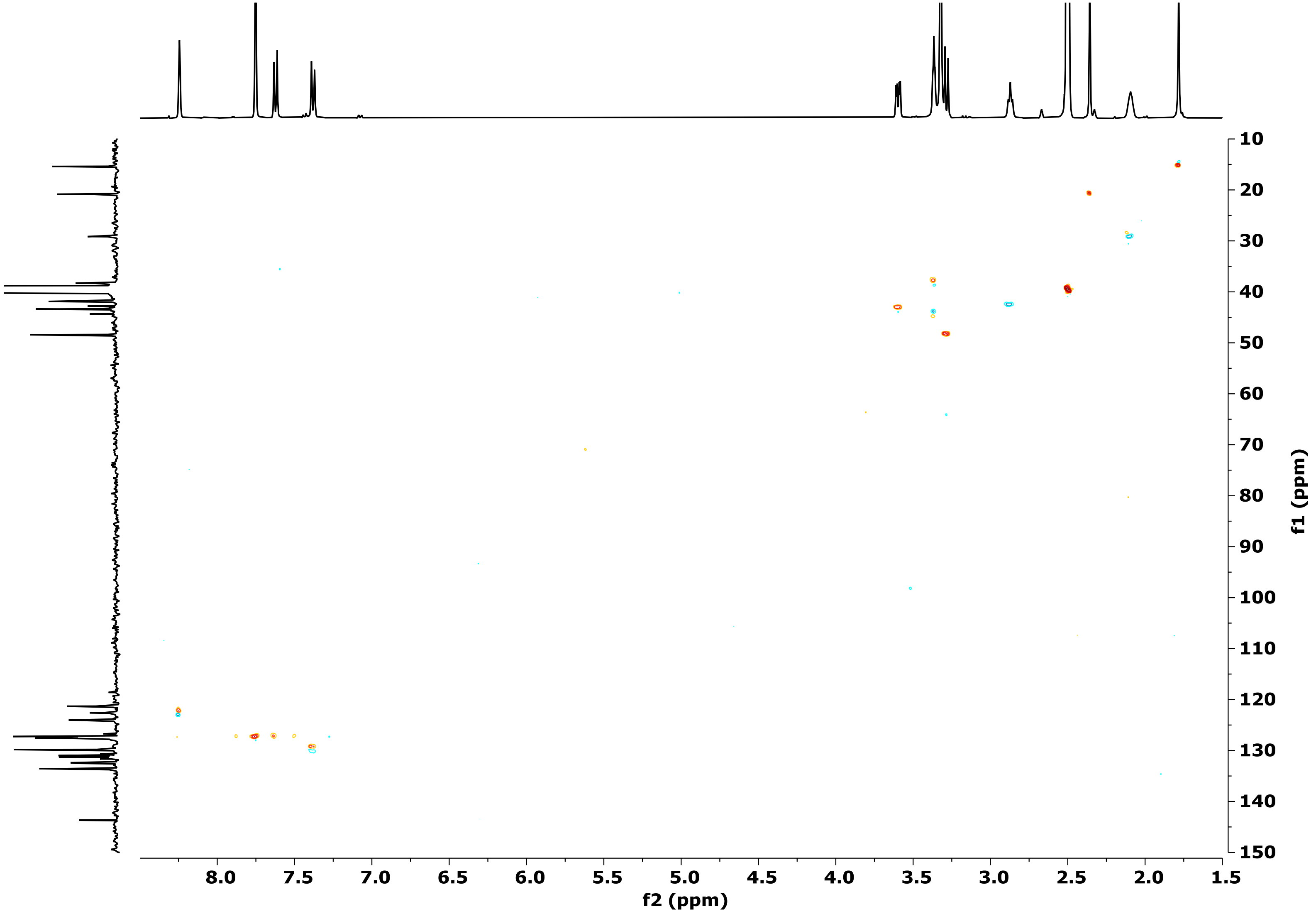

10
20
30
40
50
60
70
80
90
100
110
120
130
140
150
f1 (ppm)
8.0
7.5
7.0
6.5
6.0
5.5
5.0
4.5
4.0
3.5
3.0
2.5
2.0
1.5
f2 (ppm)

2D NMR COSY

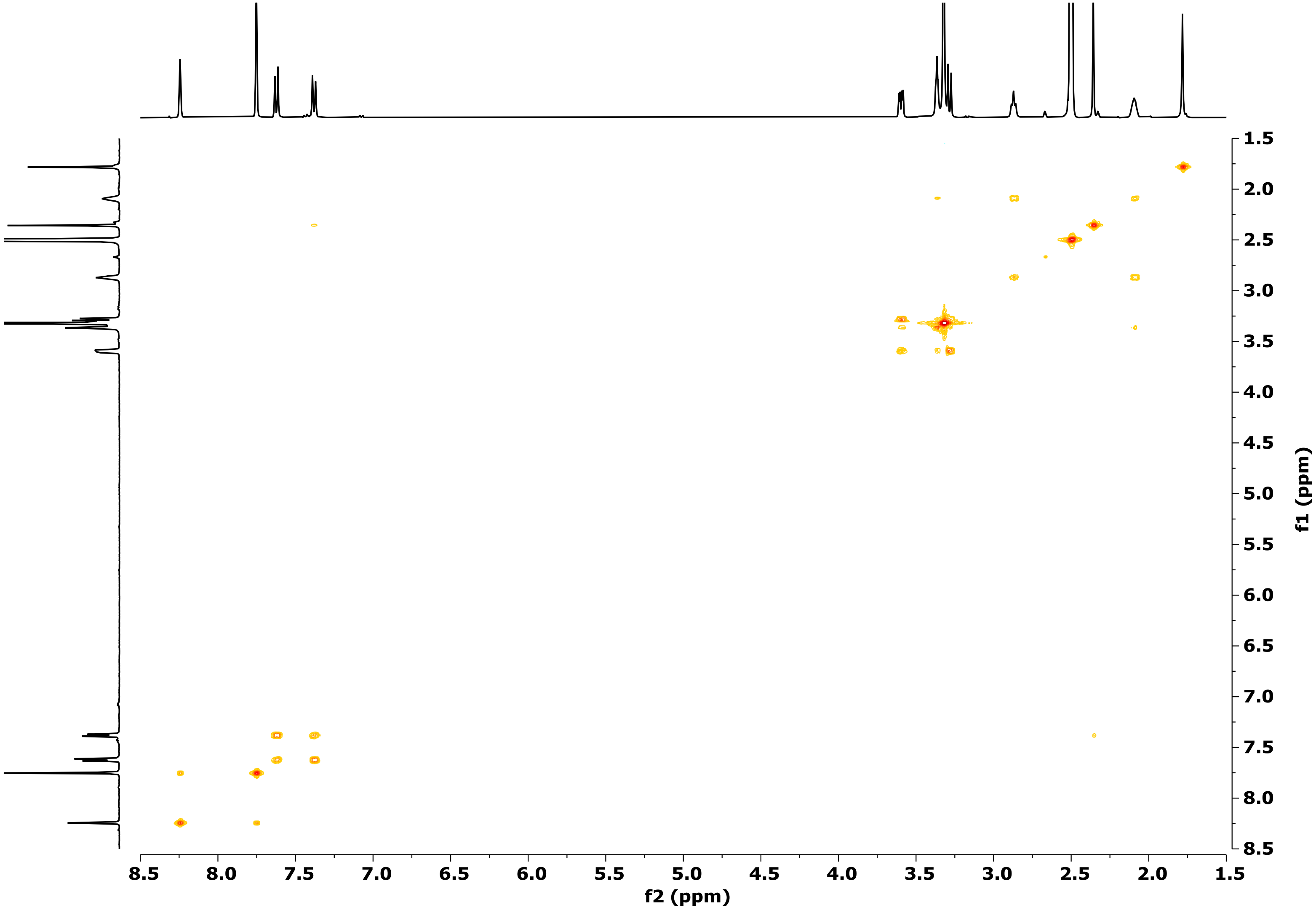

1.5
2.0
2.5
3.0
3.5
4.0
4.5
5.0
5.5
6.0
6.5
7.0
7.5
8.0
8.5
f1 (ppm)
8.5 8.0 7.5 7.0 6.5 6.0 5.5 5.0 4.5 4.0 3.5 3.0 2.5 2.0 1.5
f2 (ppm)

Compound 3m

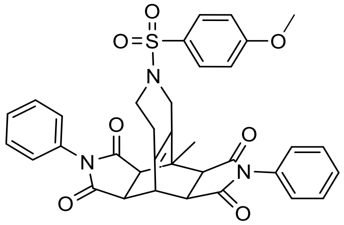

^{1}H NMR (400 MHz, $CDCl_3$)

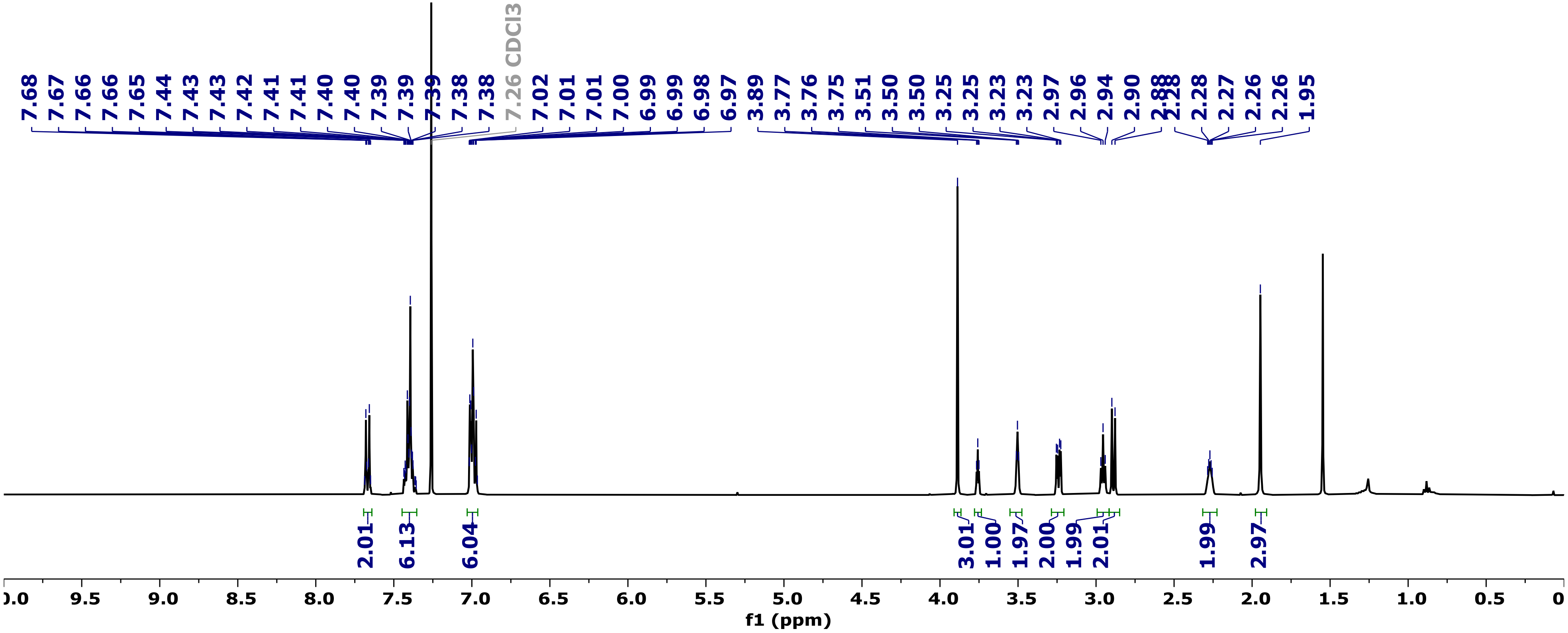

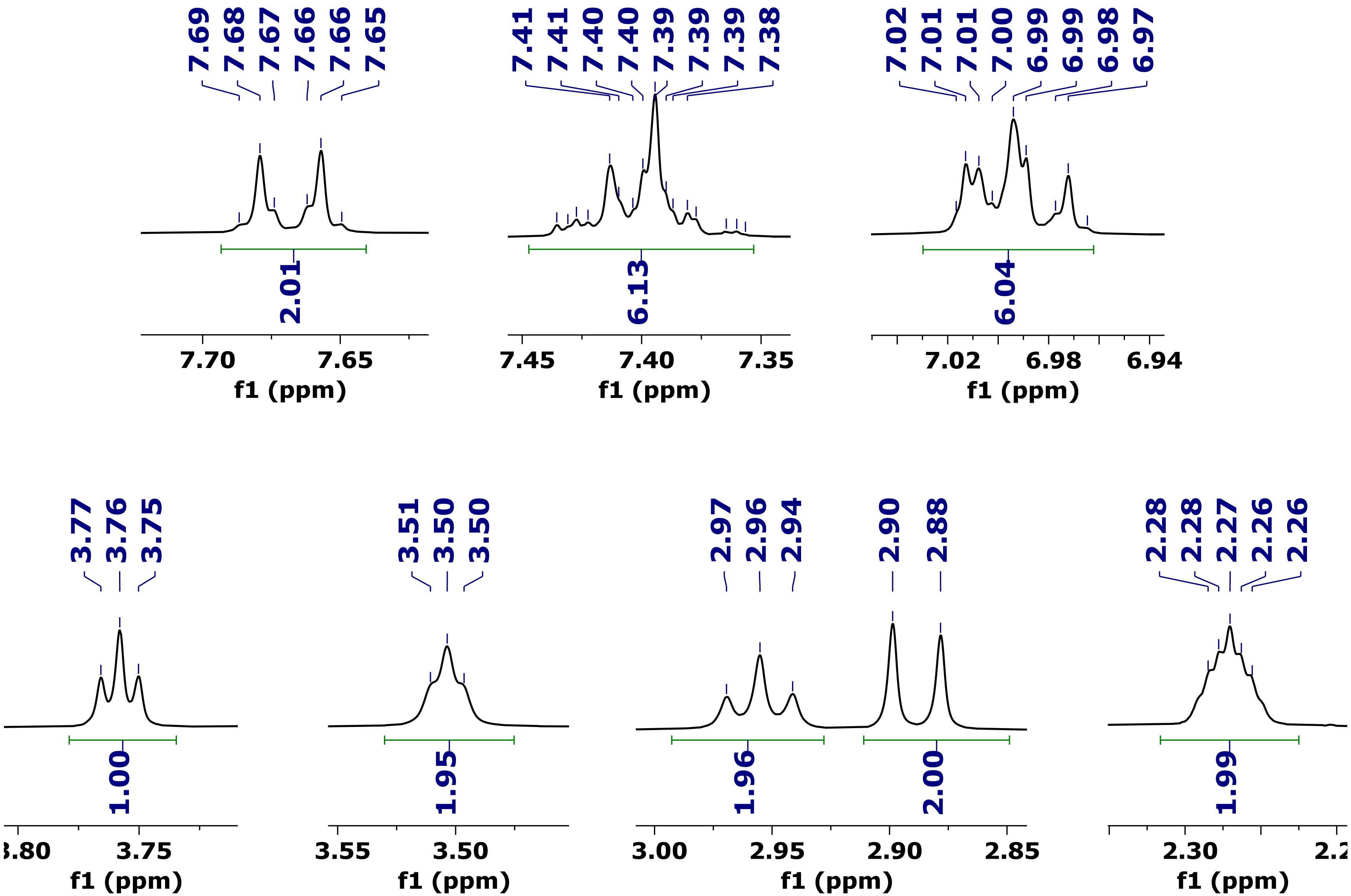
7.69 7.68 7.67 7.66 7.66 7.65
2.01
7.70 7.65
f1 (ppm)
7.41 7.41 7.40 7.40 7.39 7.39 7.39 7.38
6.13
7.45 7.40 7.35
f1 (ppm)
7.02 7.01 7.01 7.00 6.99 6.99 6.98 6.97
6.04
7.02 6.98 6.94
f1 (ppm)
3.77 3.76 3.75
1.00
3.80 3.75
f1 (ppm)
3.51 3.50 3.50
1.95
3.55 3.50
f1 (ppm)
2.97 2.96 2.94 2.90 2.88
1.96 2.00
3.00 2.95 2.90 2.85
f1 (ppm)
2.28 2.28 2.27 2.26 2.26
1.99
2.30
f1 (ppm)

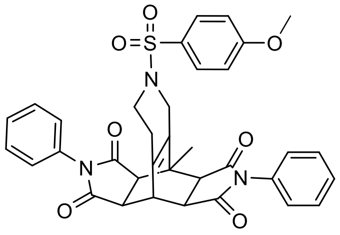

^{13}C NMR (101 MHz, CDCl$_3$)

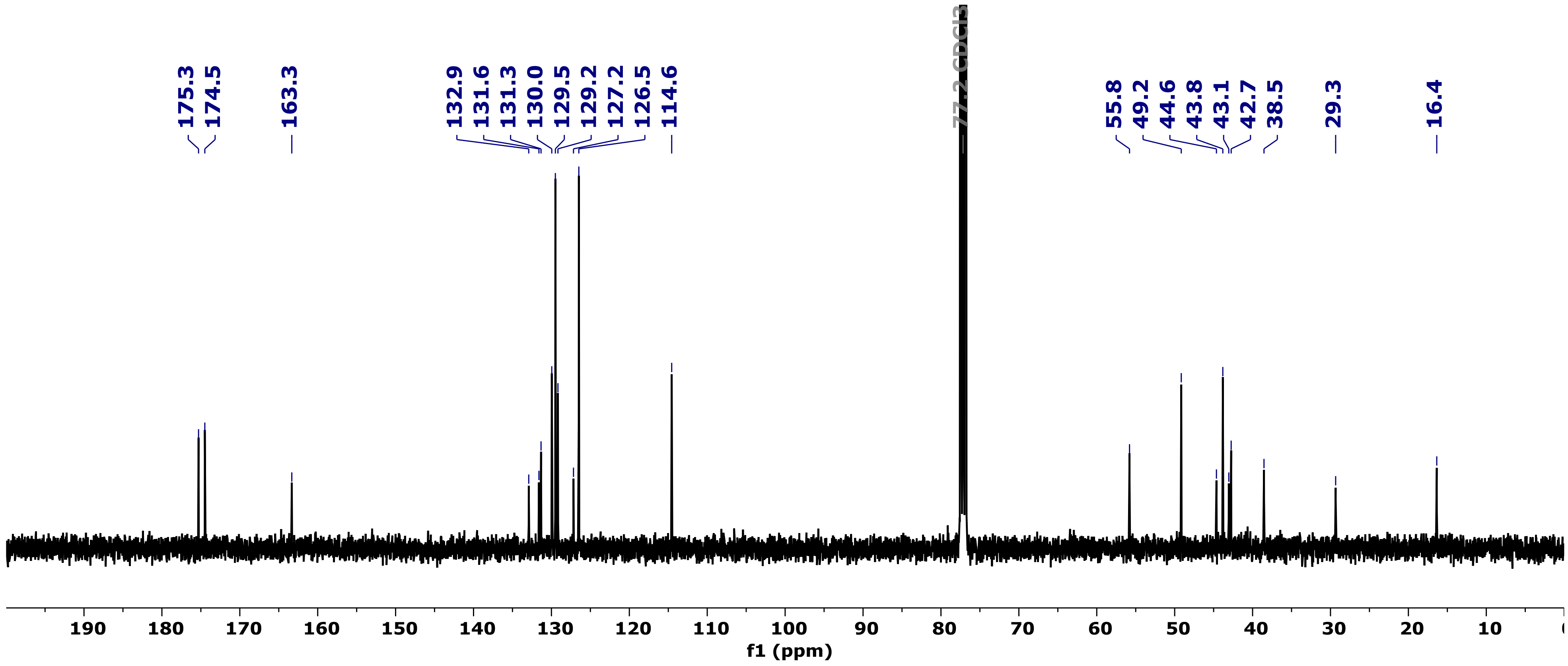

2D NMR HSQC

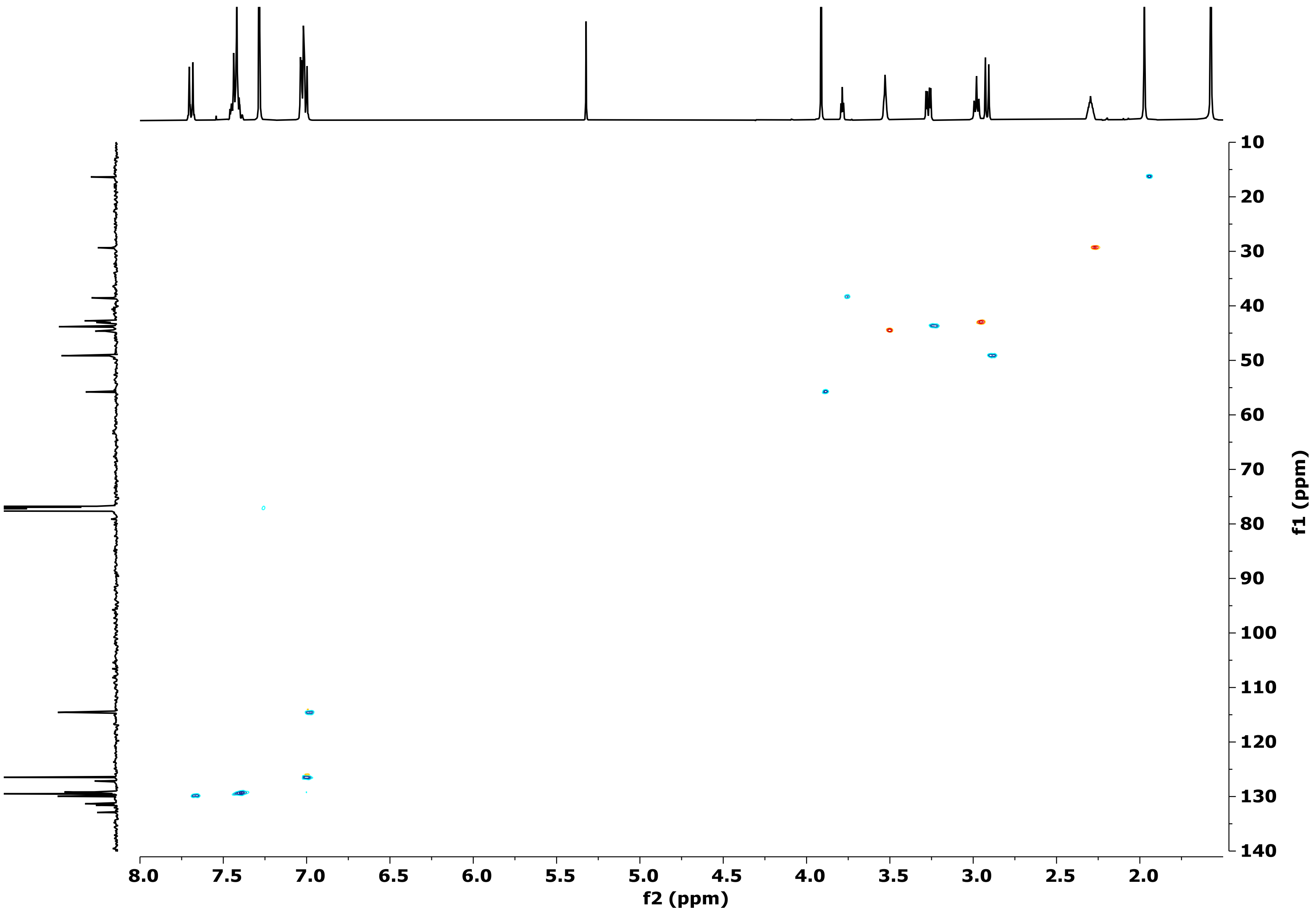

10
20
30
40
50
60
70
80
90
100
110
120
130
140
f1 (ppm)
8.0
7.5
7.0
6.5
6.0
5.5
5.0
4.5
4.0
3.5
3.0
2.5
2.0
f2 (ppm)

2D NMR COSY

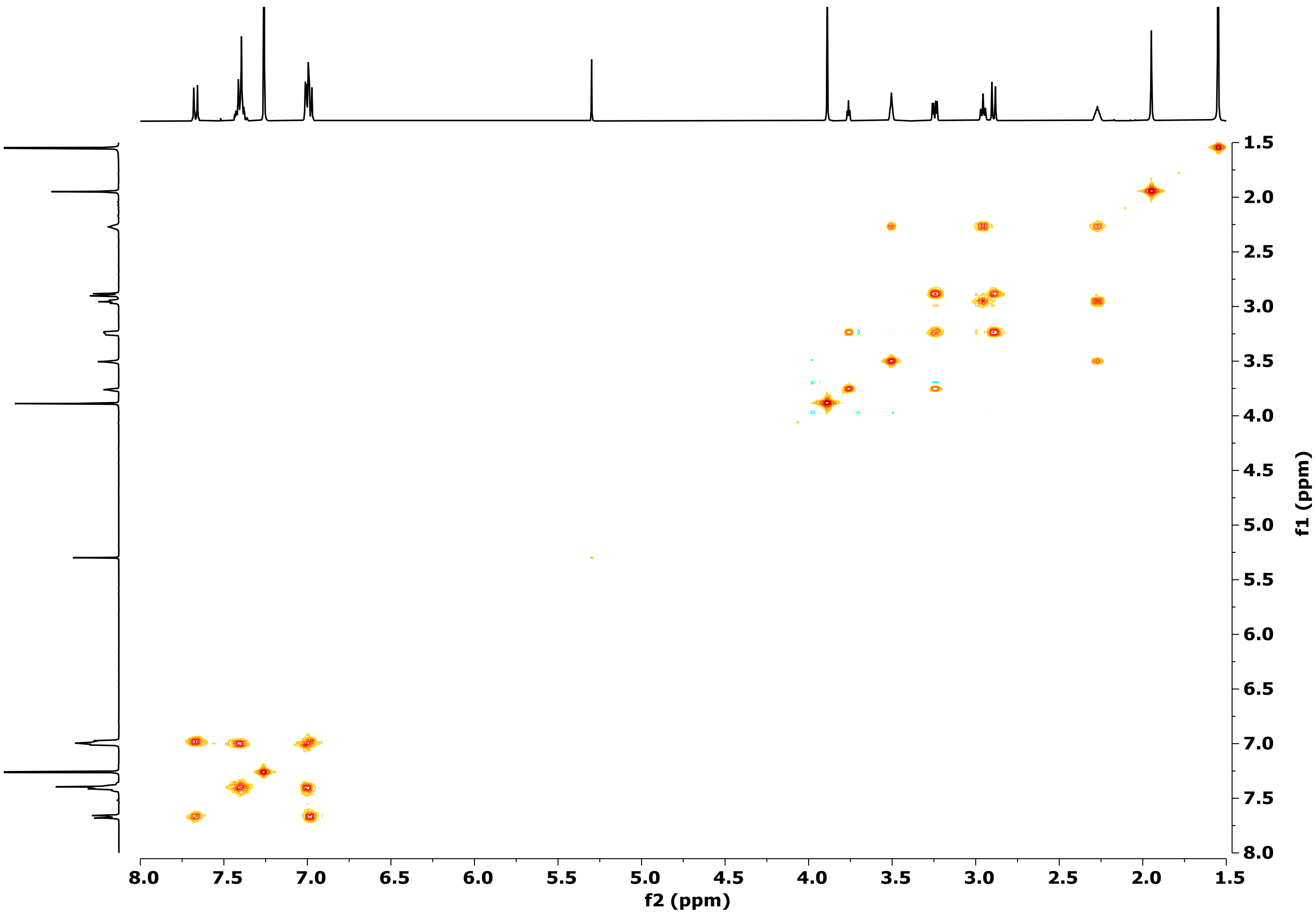

Compound 3n

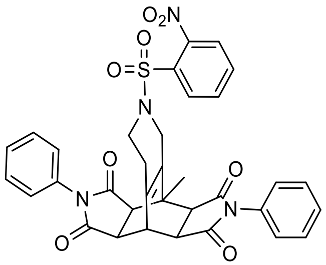

^{1}H NMR (400 MHz, $CDCl_3$)

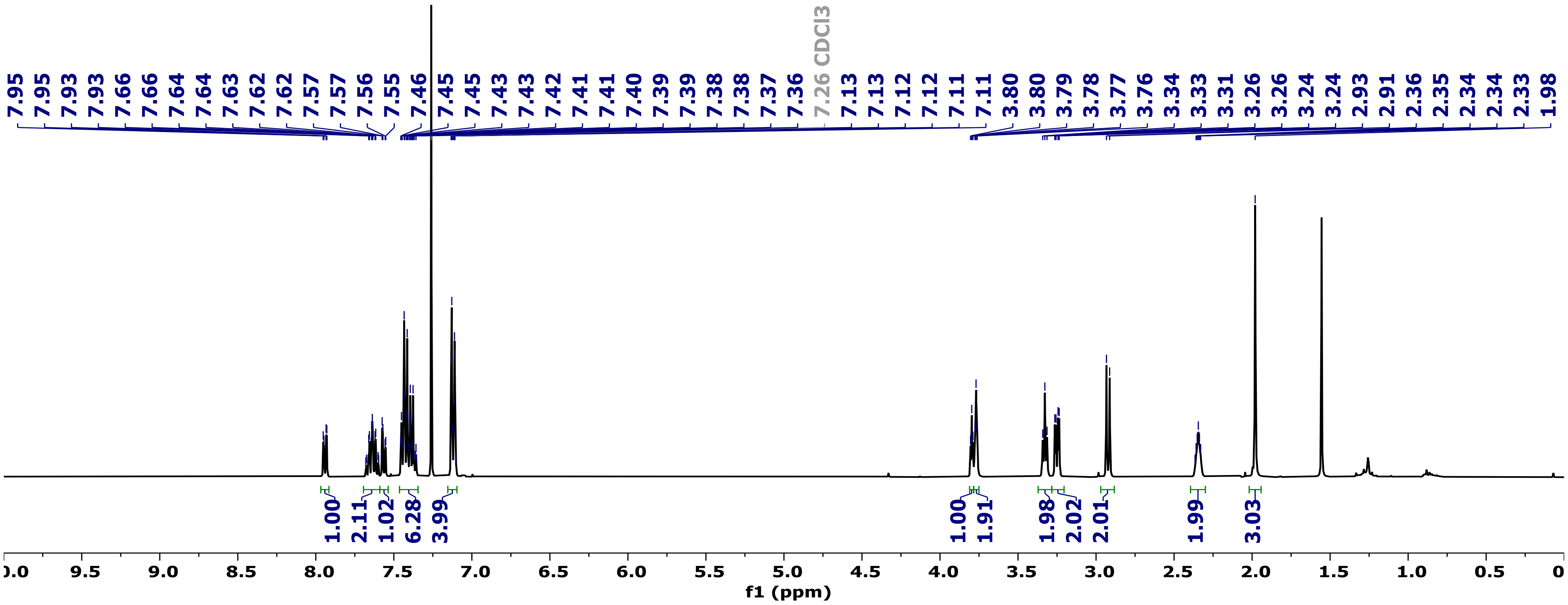

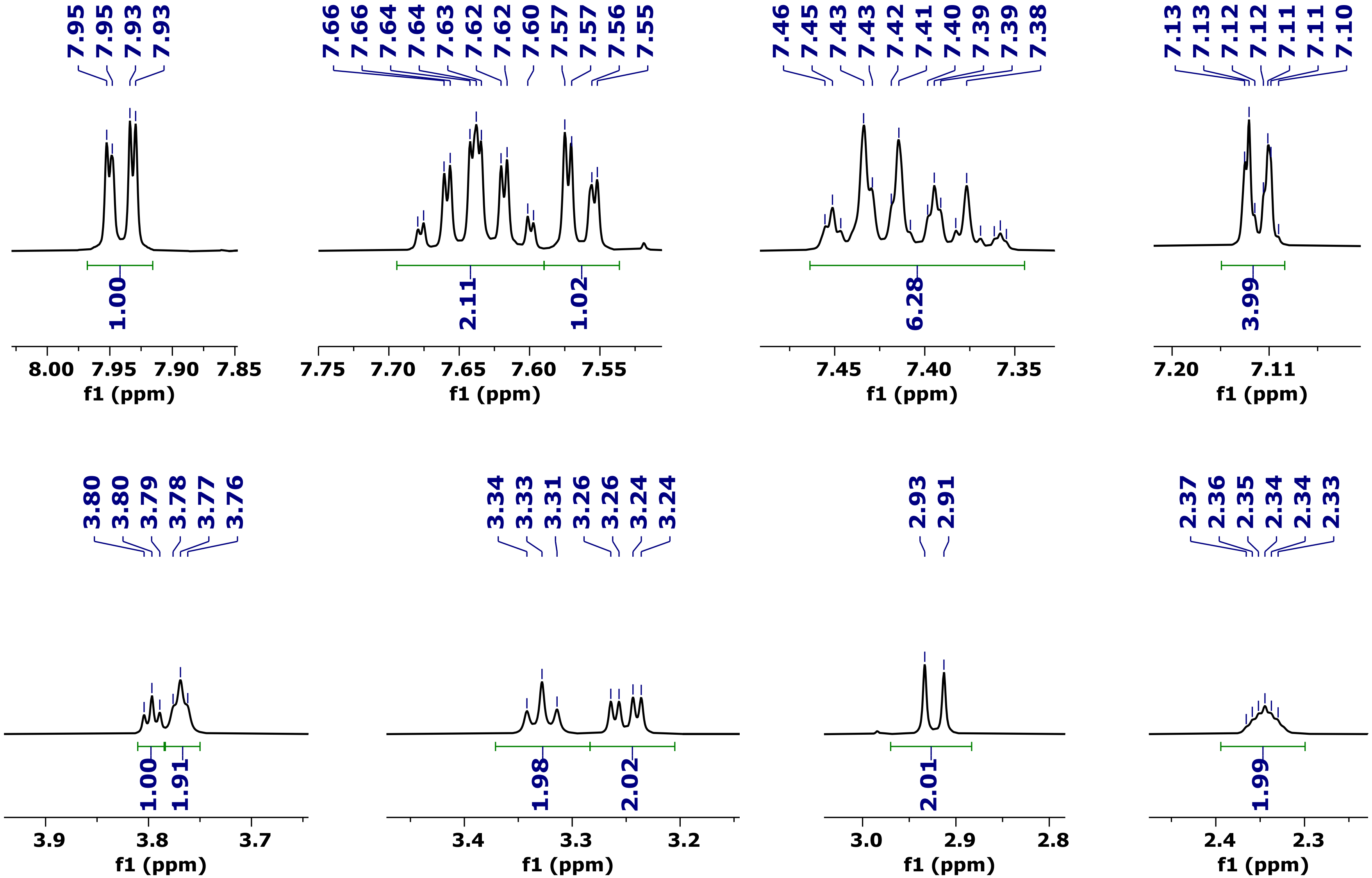
7.95 7.95 7.93 7.93
1.00
8.00 7.95 7.90 7.85
f1 (ppm)
7.66 7.66 7.64 7.64 7.63 7.62 7.62 7.60 7.57 7.57 7.56 7.55
2.11
1.02
7.75 7.70 7.65 7.60 7.55
f1 (ppm)
7.46 7.45 7.43 7.43 7.42 7.41 7.40 7.39 7.39 7.38
6.28
7.45 7.40 7.35
f1 (ppm)
7.13 7.13 7.12 7.12 7.11 7.11 7.10
3.99
7.20 7.11
f1 (ppm)
3.80 3.80 3.79 3.78 3.77 3.76
1.00
1.91
3.9 3.8 3.7
f1 (ppm)
3.34 3.33 3.31 3.26 3.26 3.24 3.24
1.98
2.02
3.4 3.3 3.2
f1 (ppm)
2.93 2.91
2.01
3.0 2.9 2.8
f1 (ppm)
2.37 2.36 2.35 2.34 2.34 2.33
1.99
2.4 2.3
f1 (ppm)

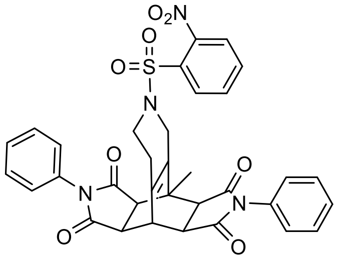

^{13}C NMR (101 MHz, CDCl3)

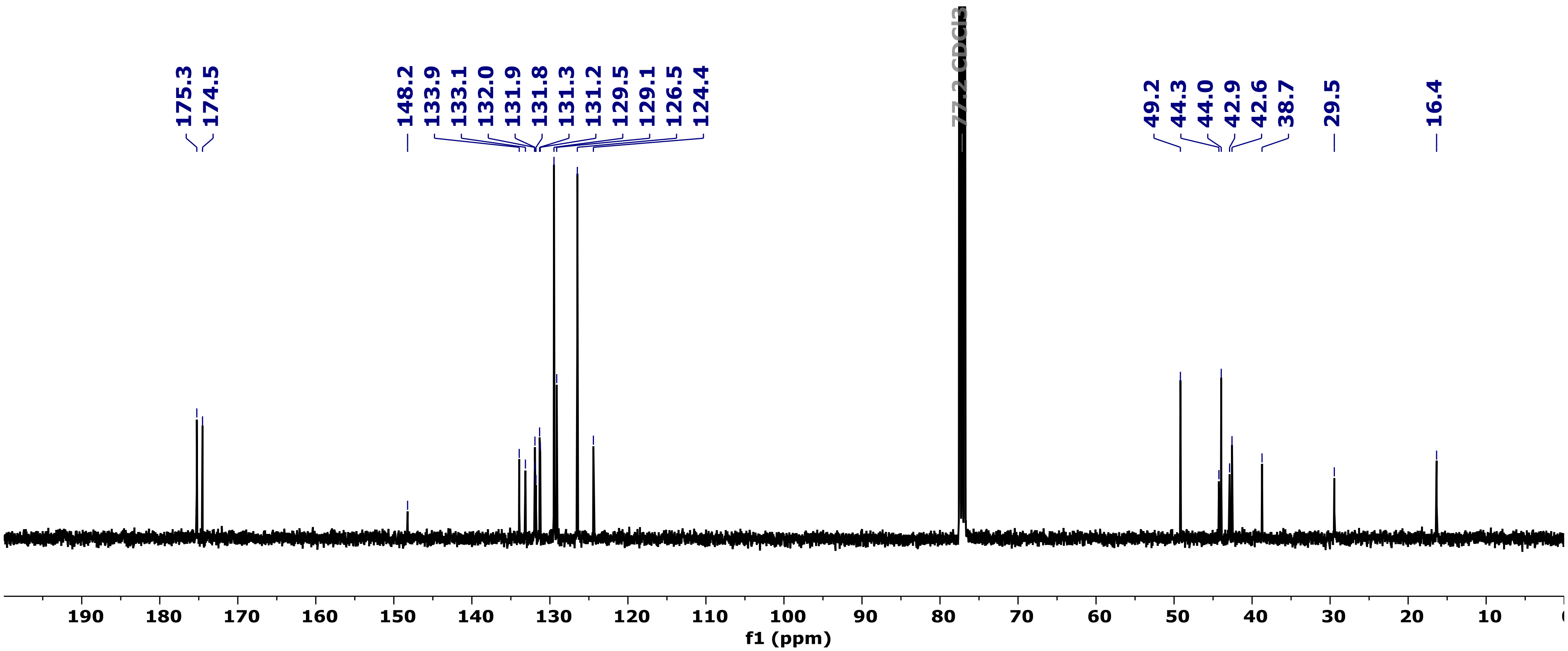

2D NMR HSQC

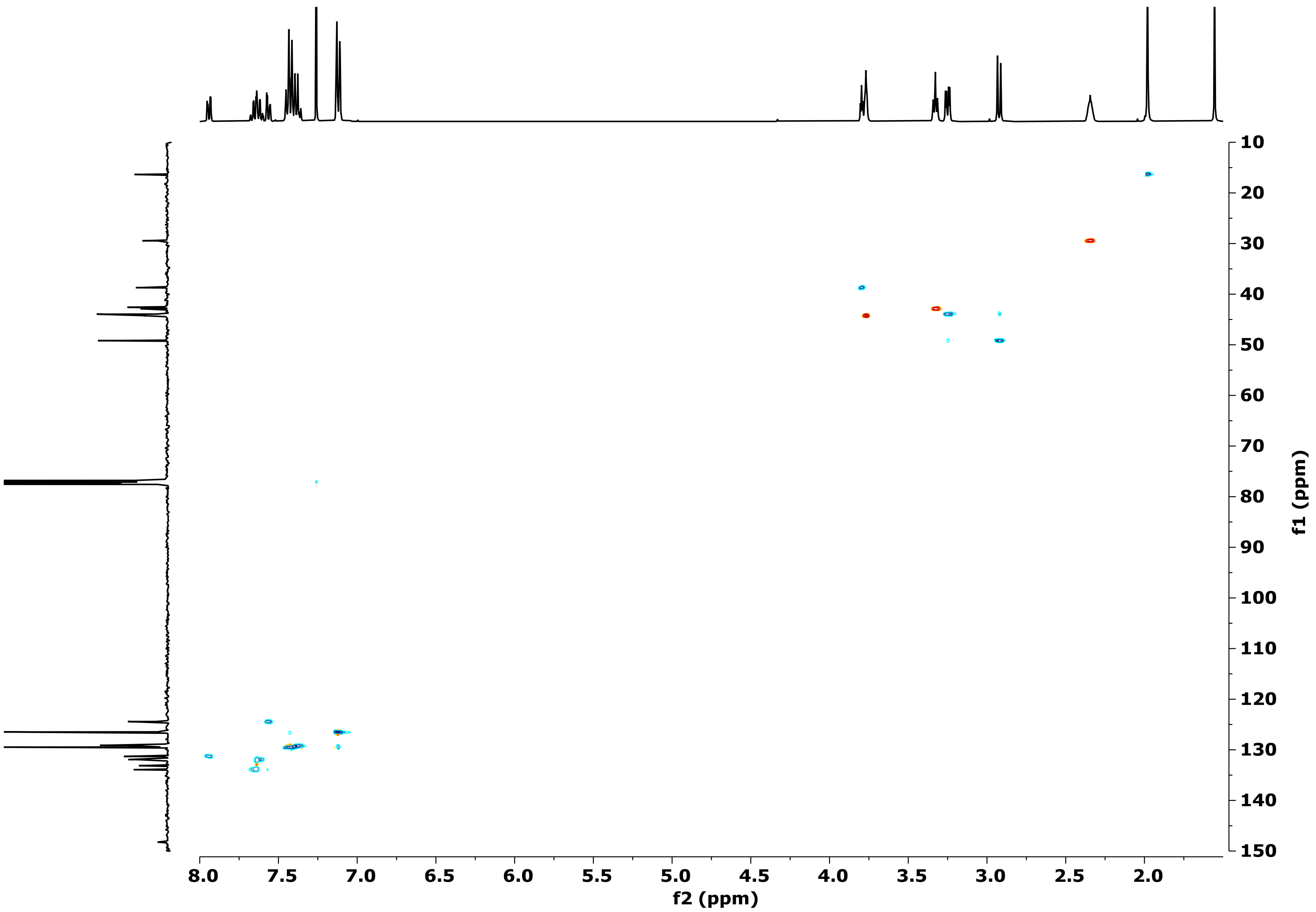

2D NMR COSY

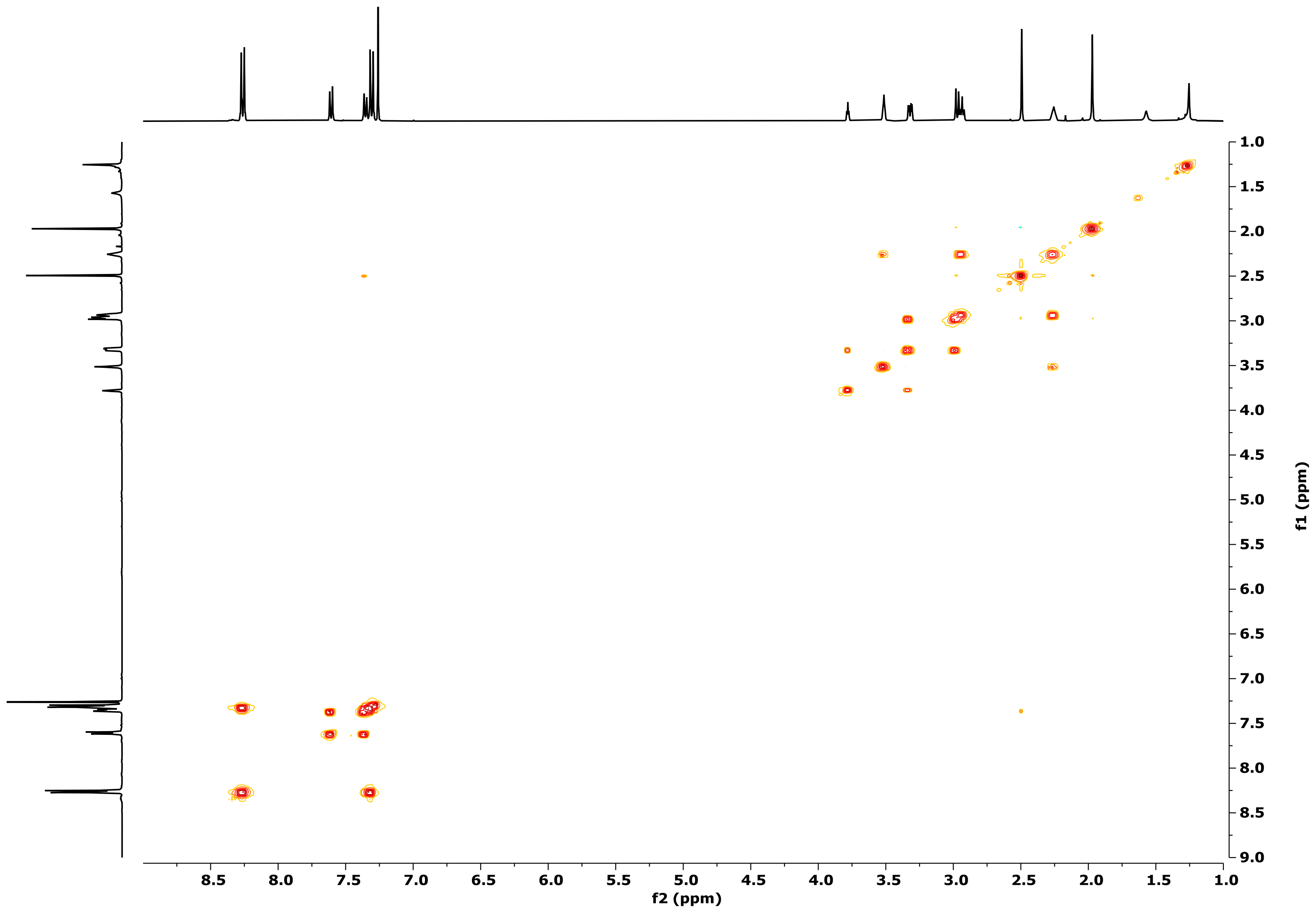

Compound 3o

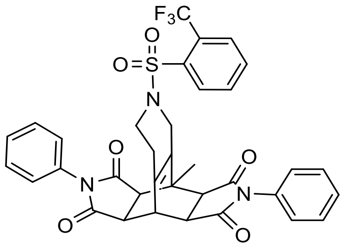

^{1}H NMR (400 MHz, CDCl$_3$)

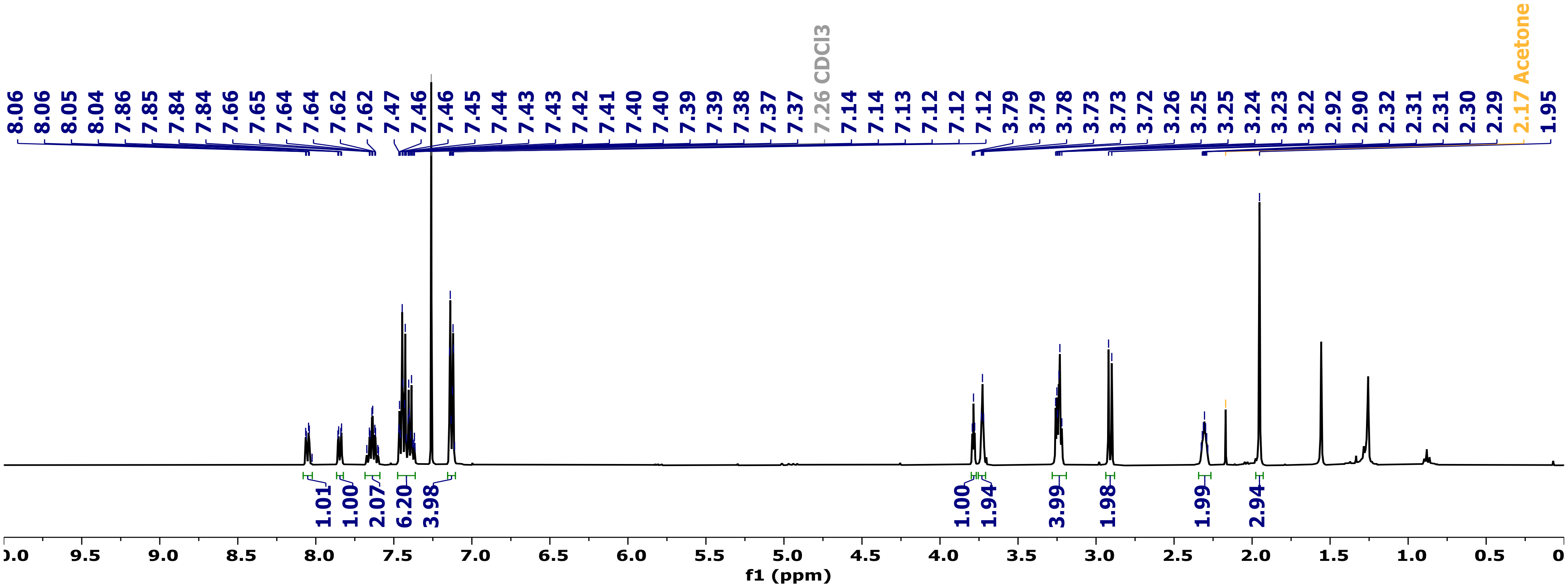

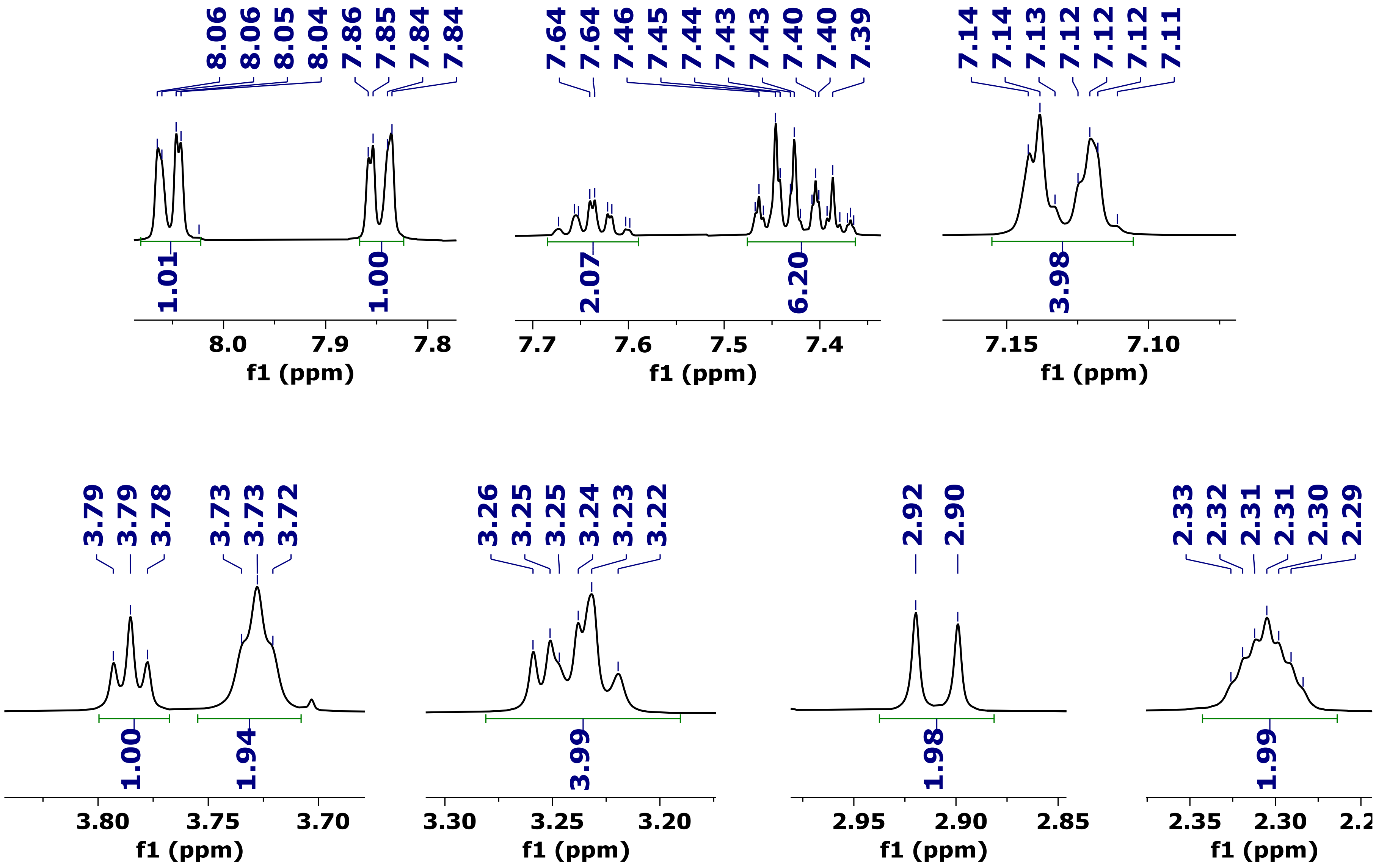
8.06 8.06 8.05 8.04 7.86 7.85 7.84 7.84
1.01 1.00
8.0 7.9 7.8
f1 (ppm)
7.64 7.64 7.46 7.45 7.44 7.43 7.43 7.40 7.40 7.39
2.07 6.20
7.7 7.6 7.5 7.4
f1 (ppm)
7.14 7.14 7.13 7.12 7.12 7.12 7.11
3.98
7.15 7.10
f1 (ppm)
3.79 3.79 3.78 3.73 3.73 3.72
1.00 1.94
3.80 3.75 3.70
f1 (ppm)
3.26 3.25 3.25 3.24 3.23 3.22
3.99
3.30 3.25 3.20
f1 (ppm)
2.92 2.90
1.98
2.95 2.90 2.85
f1 (ppm)
2.33 2.32 2.31 2.31 2.30 2.29
1.99
2.35 2.30
f1 (ppm)

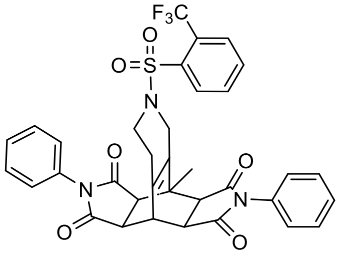

^{13}C NMR (101 MHz, CDCl$_3$)

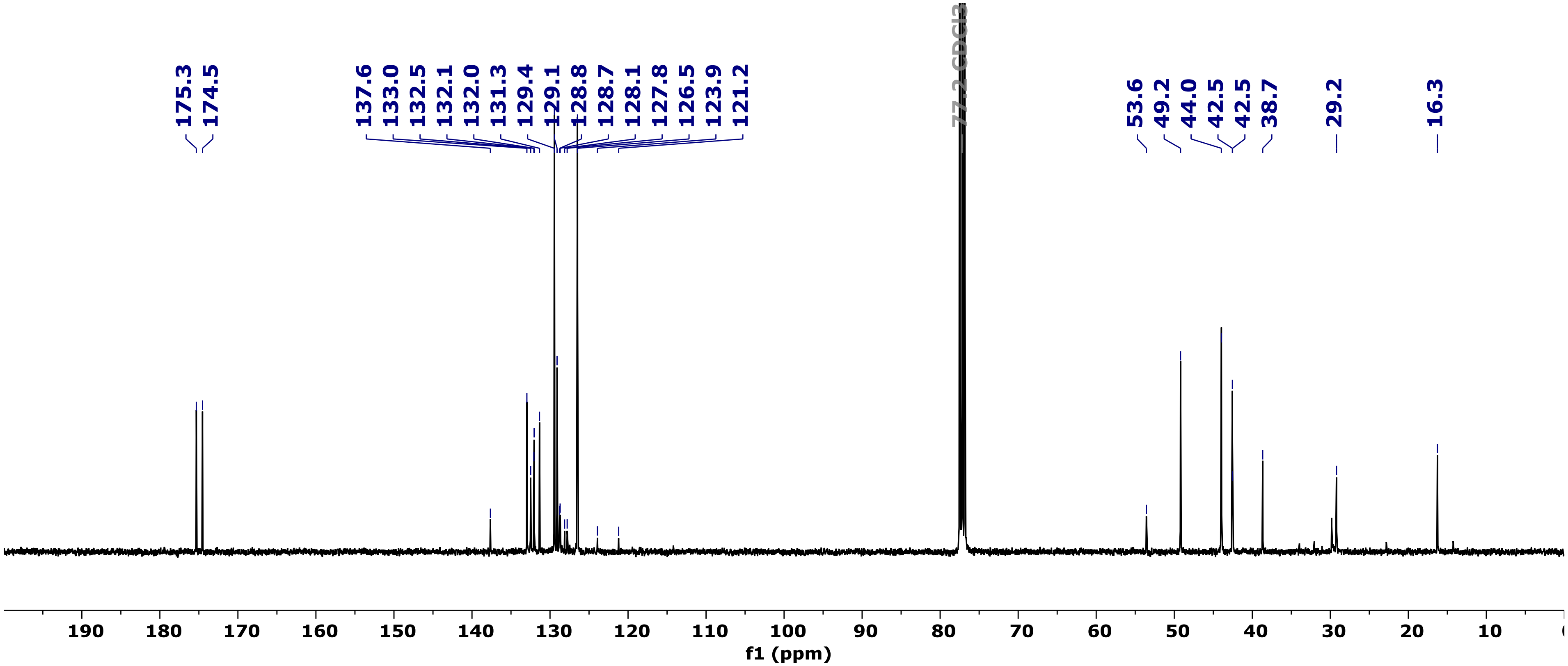

2D NMR HSQC

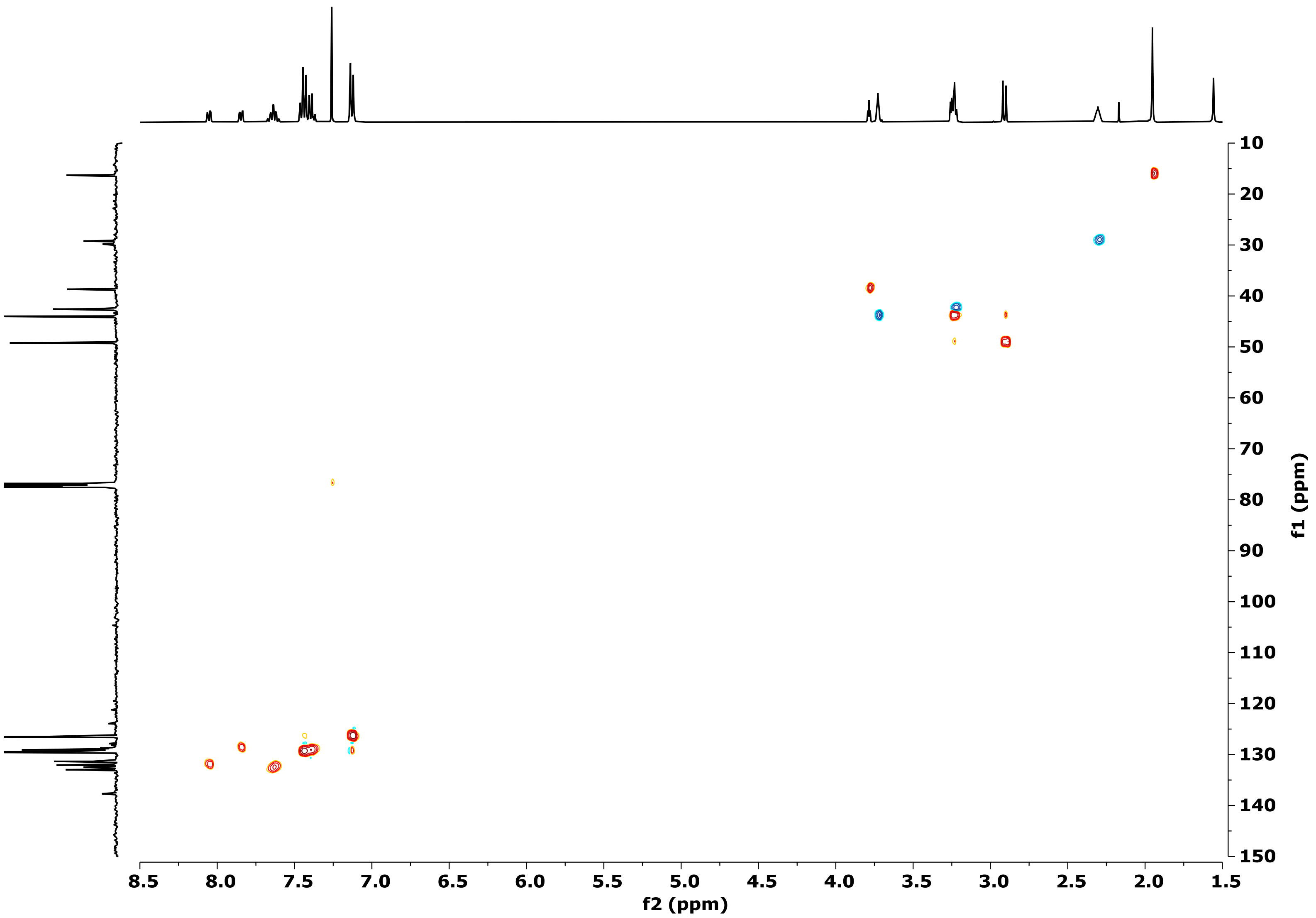

2D NMR COSY

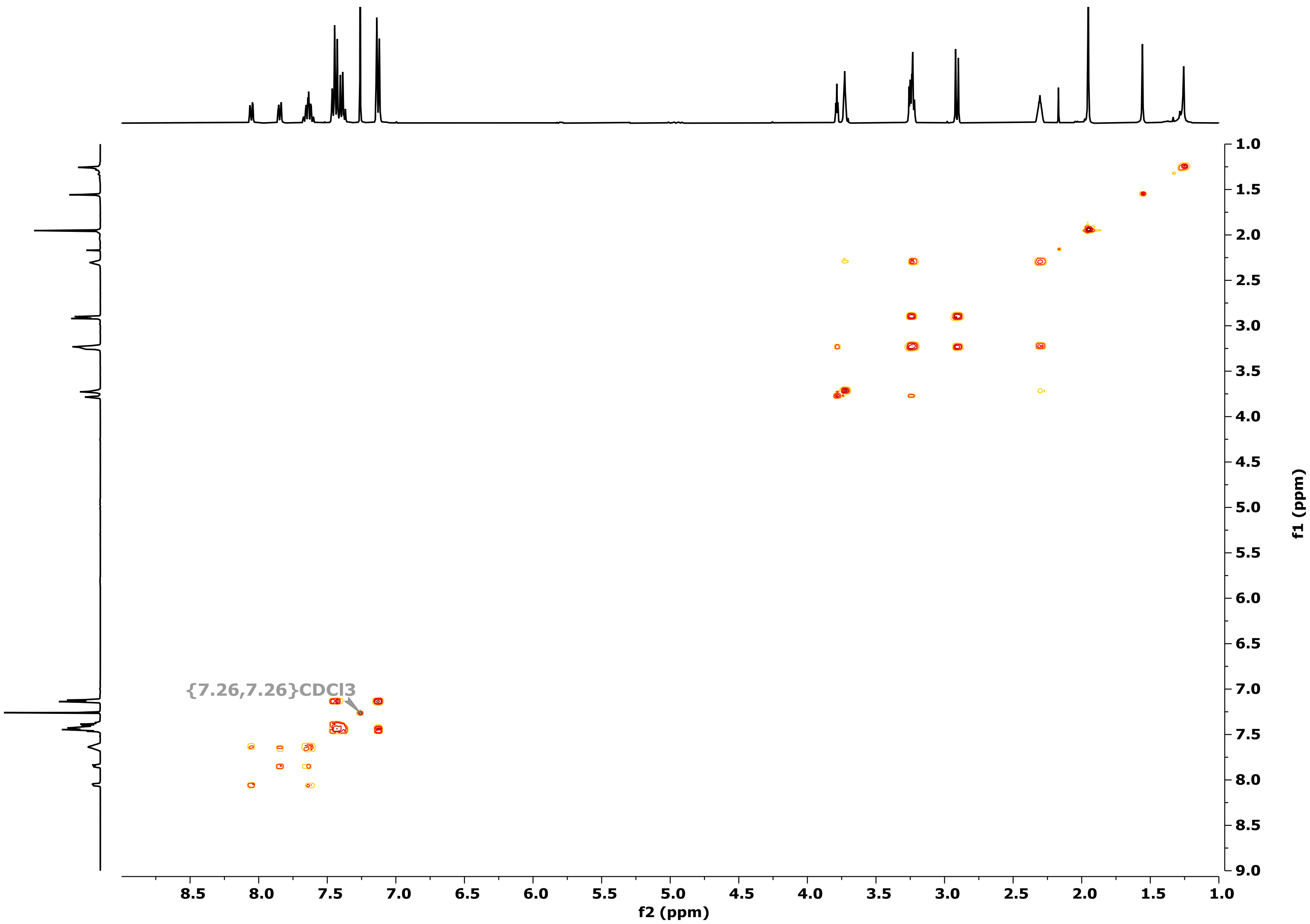
{7.26,7.26}CDCl3
f2 (ppm)
f1 (ppm)
8.5
8.0
7.5
7.0
6.5
6.0
5.5
5.0
4.5
4.0
3.5
3.0
2.5
2.0
1.5
1.0
9.0

Compound 3p

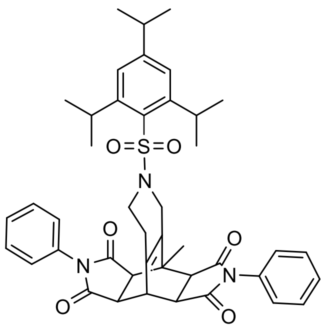

^{1}H NMR (400 MHz, $CDCl_3$)

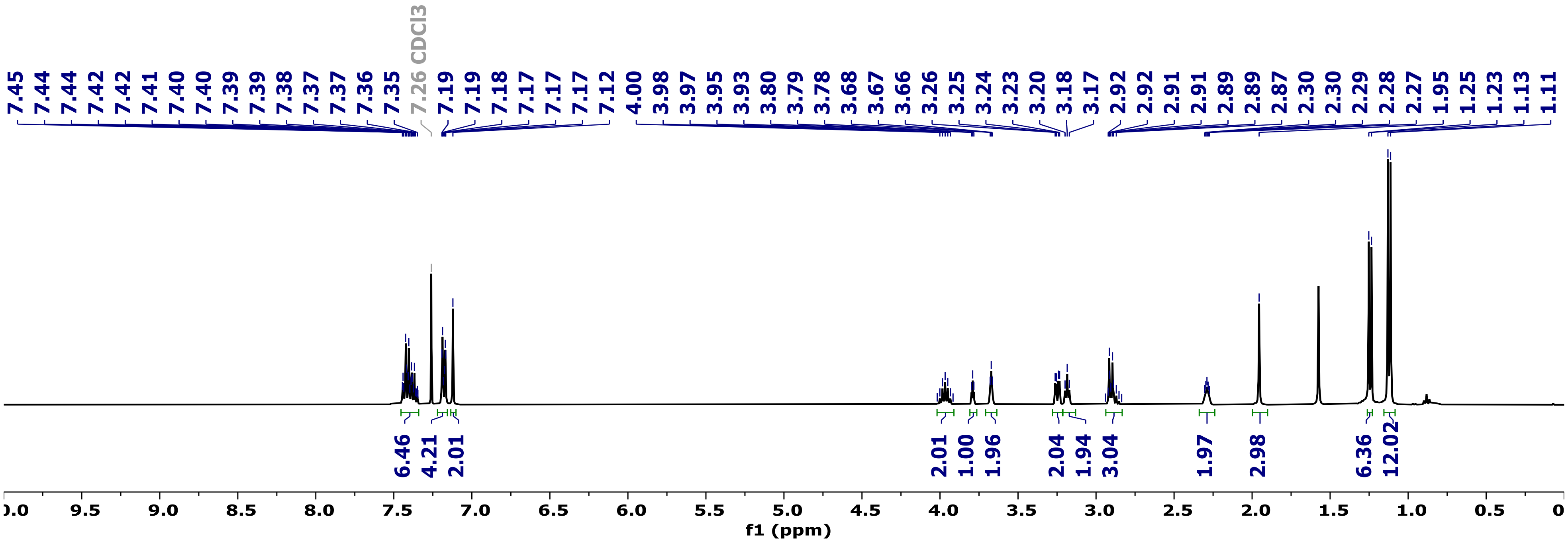

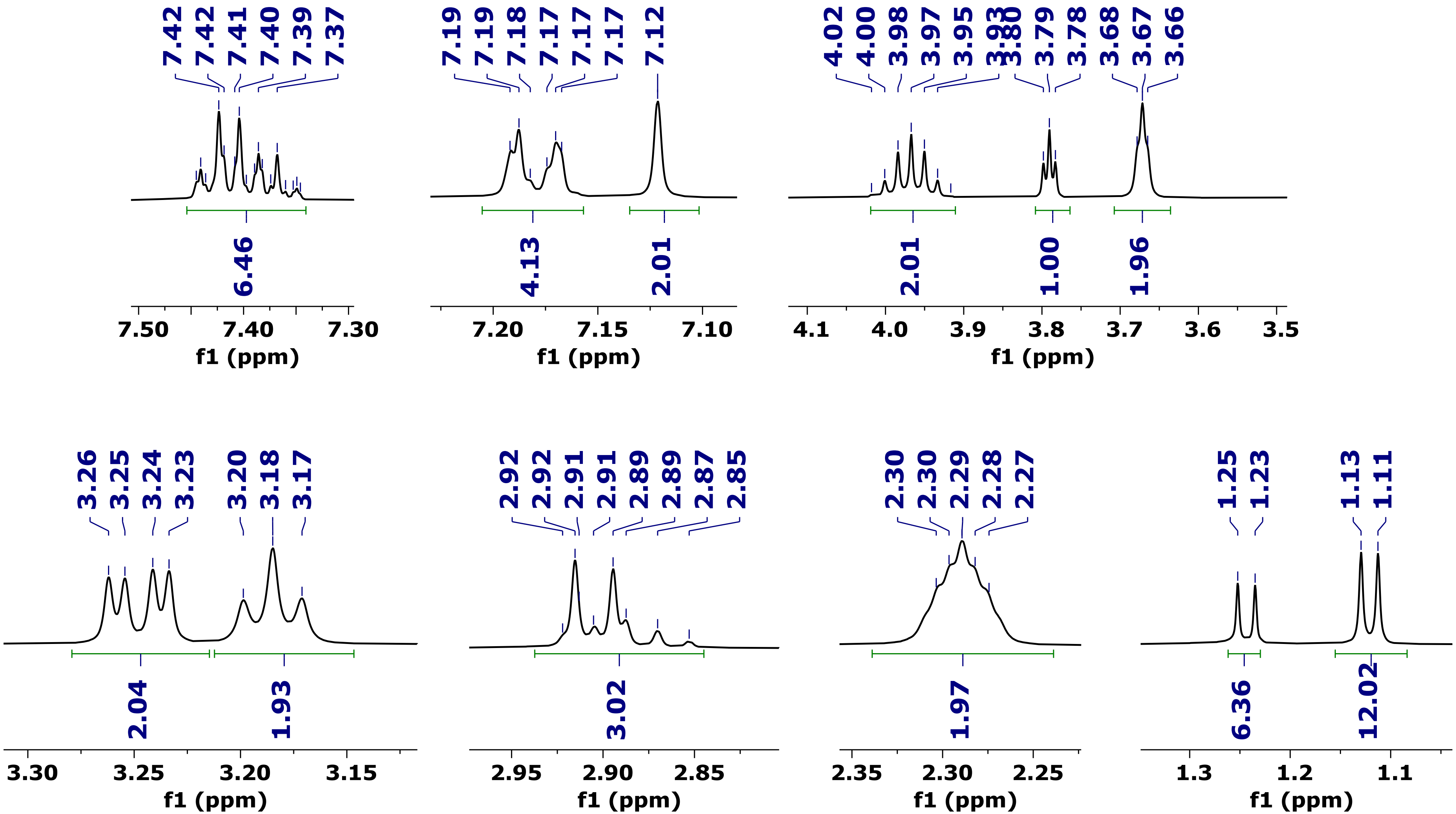

7.42
7.42
7.41
7.40
7.39
7.37
6.46
7.50
7.40
7.30
f1 (ppm)
7.19
7.19
7.18
7.17
7.17
7.17
7.12
4.13
2.01
7.20
7.15
7.10
f1 (ppm)
4.02
4.00
3.98
3.97
3.95
3.93
3.80
3.79
3.78
3.68
3.67
3.66
2.01
1.00
1.96
4.1
4.0
3.9
3.8
3.7
3.6
3.5
f1 (ppm)
3.26
3.25
3.24
3.23
3.20
3.18
3.17
2.04
1.93
3.30
3.25
3.20
3.15
f1 (ppm)
2.92
2.92
2.91
2.91
2.89
2.89
2.87
2.85
3.02
2.95
2.90
2.85
f1 (ppm)
2.30
2.30
2.29
2.28
2.27
1.97
2.35
2.30
2.25
f1 (ppm)
1.25
1.23
1.13
1.11
6.36
12.02
1.3
1.2
1.1
f1 (ppm)

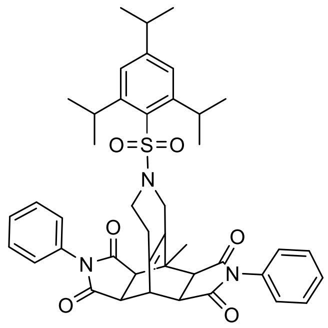

^{13}C NMR (101 MHz, $CDCl_3$)

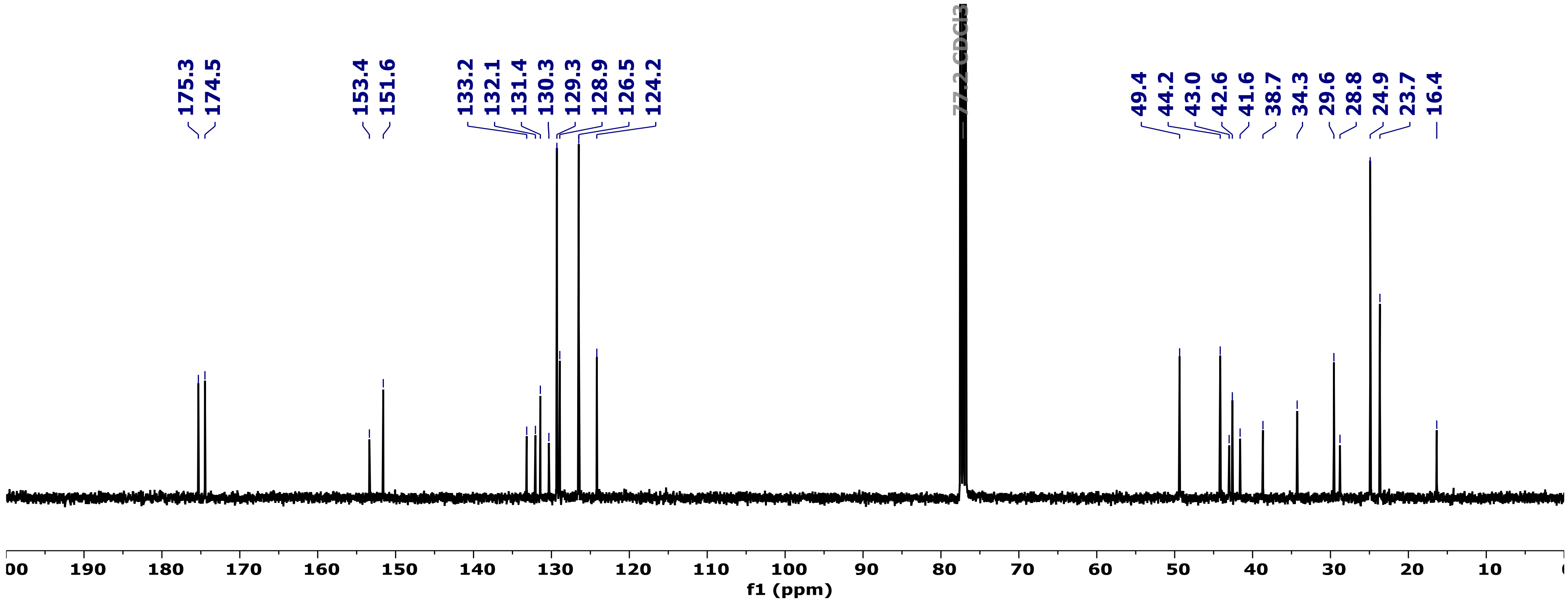

2D NMR HSQC

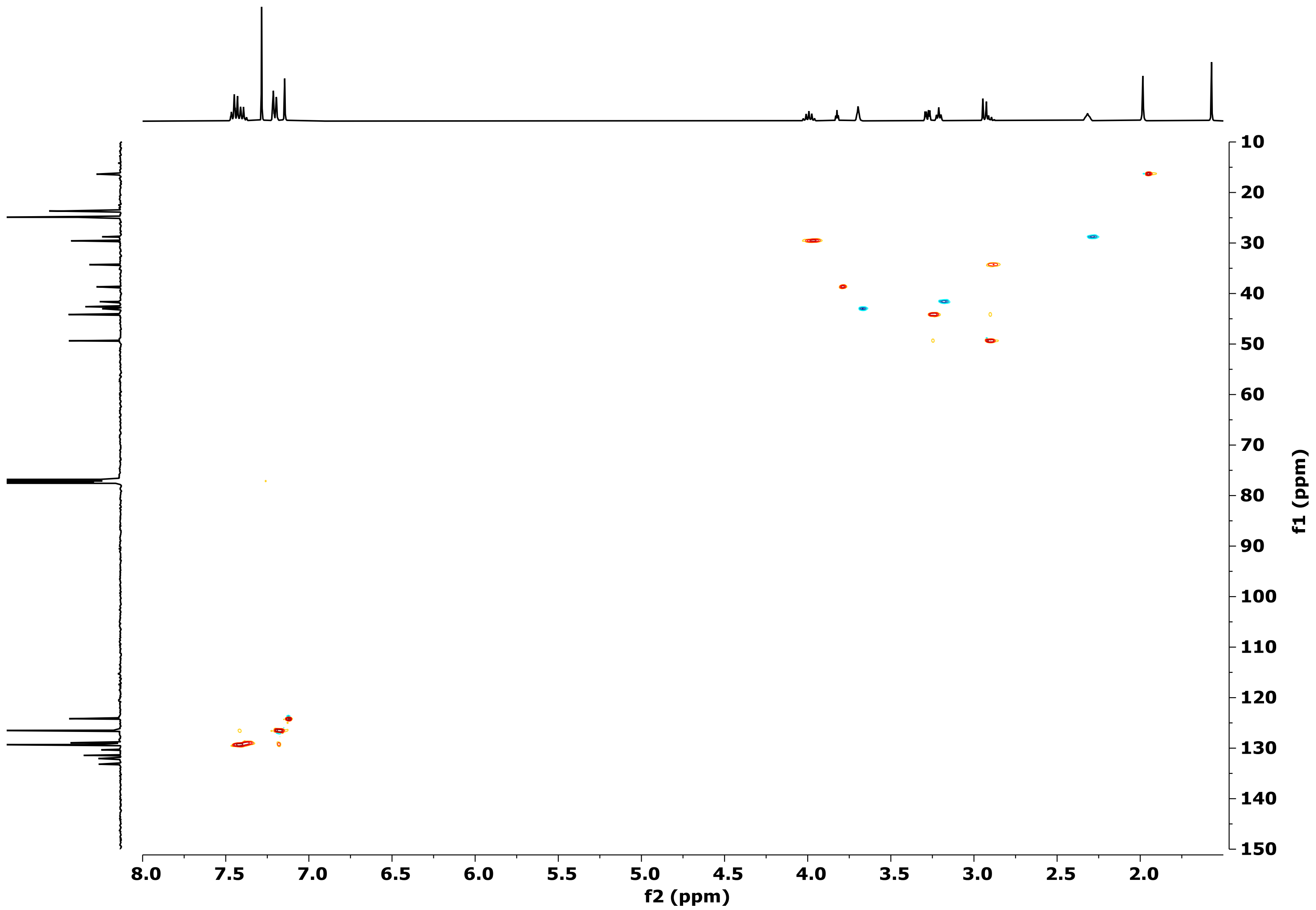

f2 (ppm)
f1 (ppm)
8.0
7.5
7.0
6.5
6.0
5.5
5.0
4.5
4.0
3.5
3.0
2.5
2.0
10
20
30
40
50
60
70
80
90
100
110
120
130
140
150

2D NMR COSY

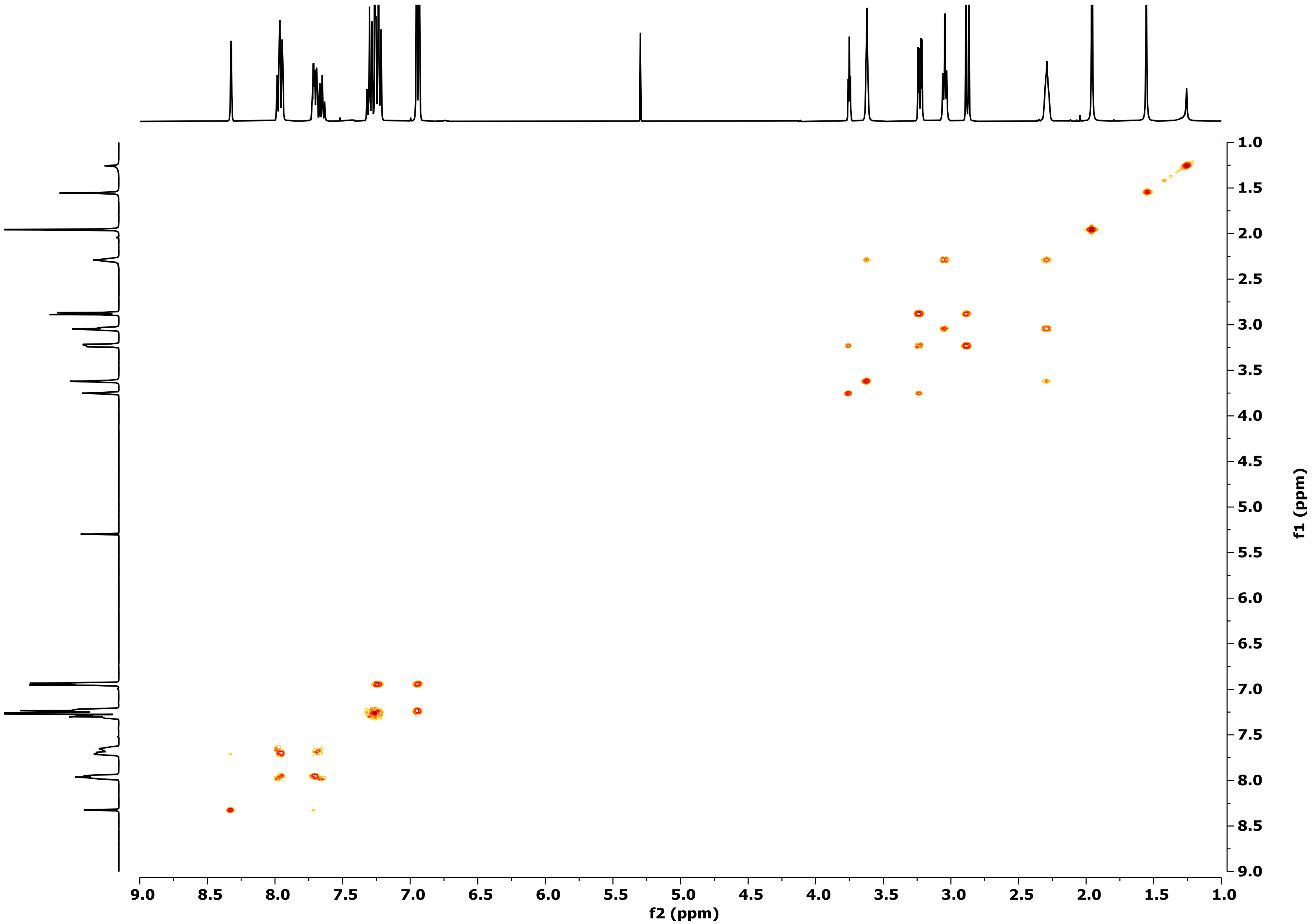

Compound 3q

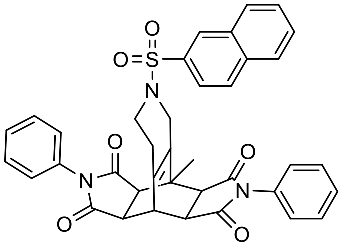

^{1}H NMR (400 MHz, CDCl$_3$)

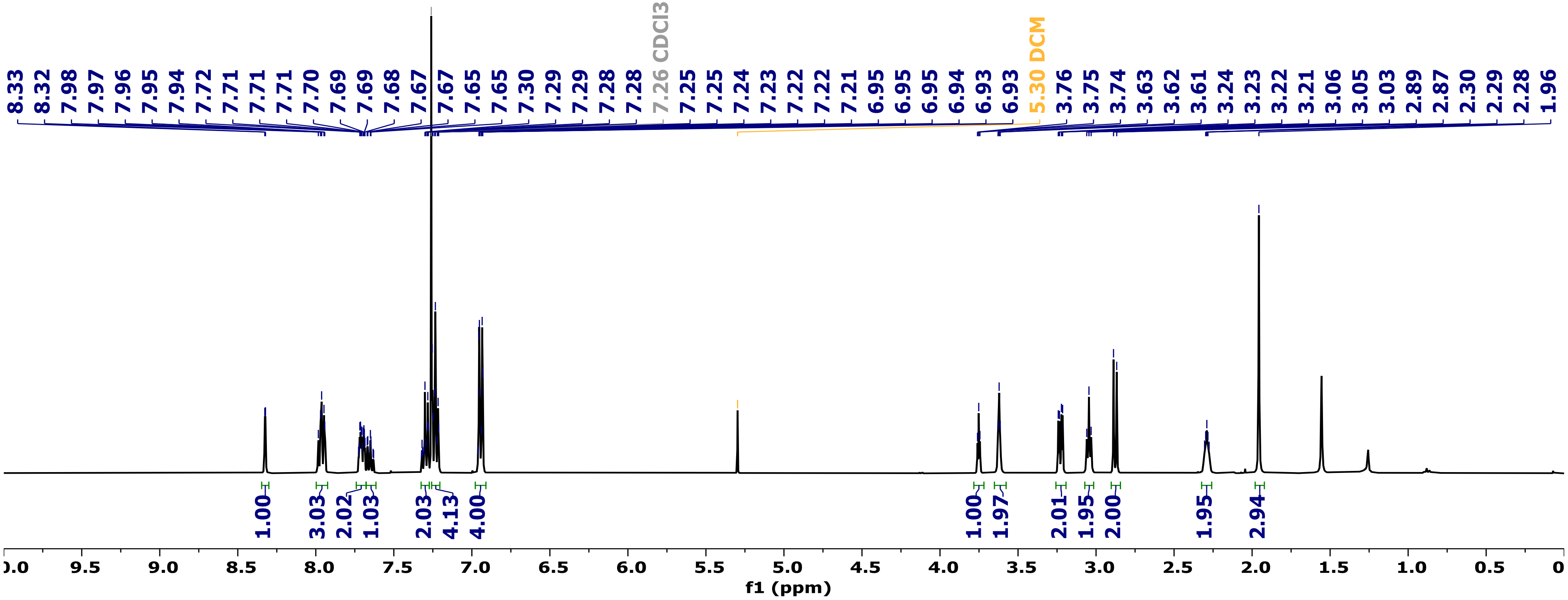

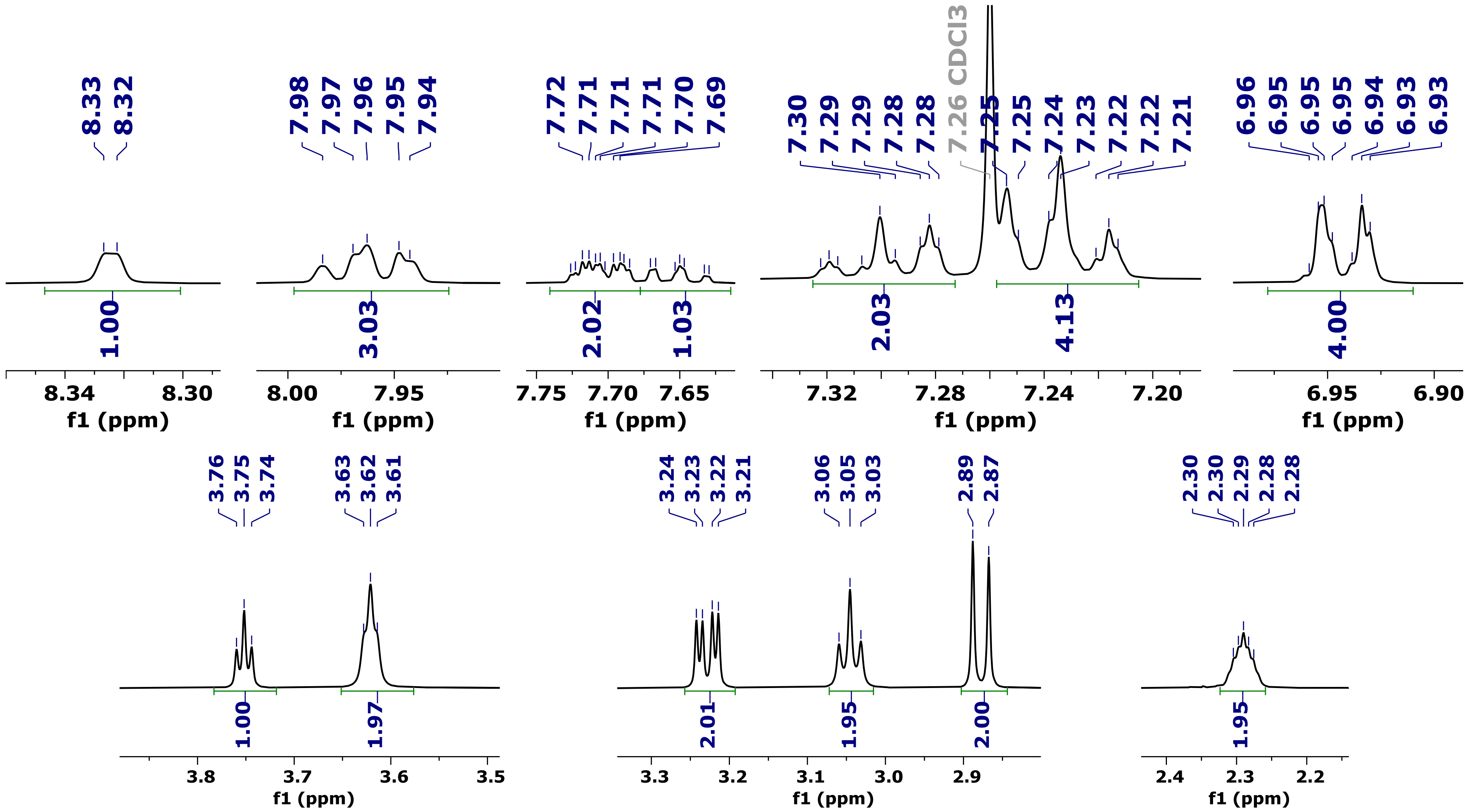
7.26 CDCl3
f1 (ppm)

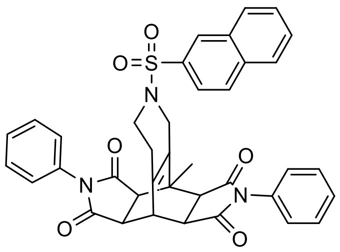

^{13}C NMR (101 MHz, $CDCl_3$)

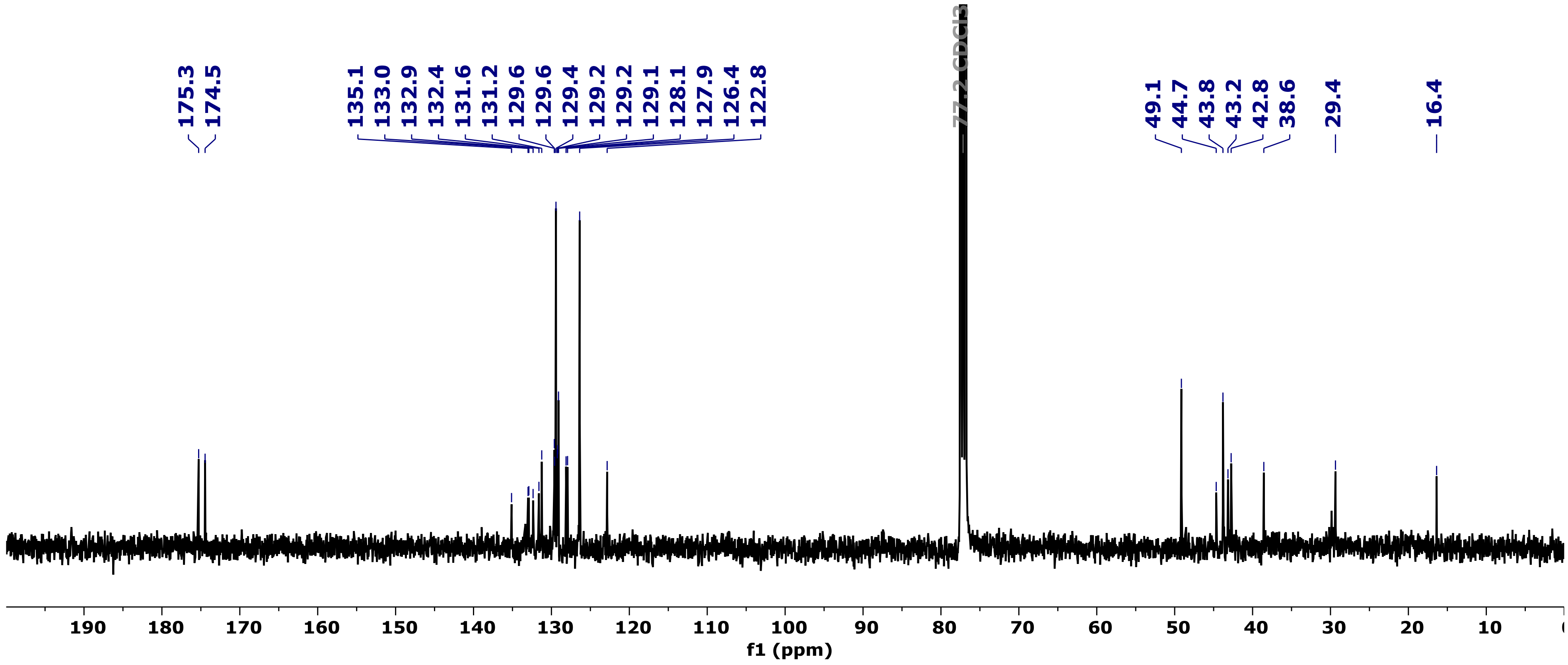

2D NMR HSQC

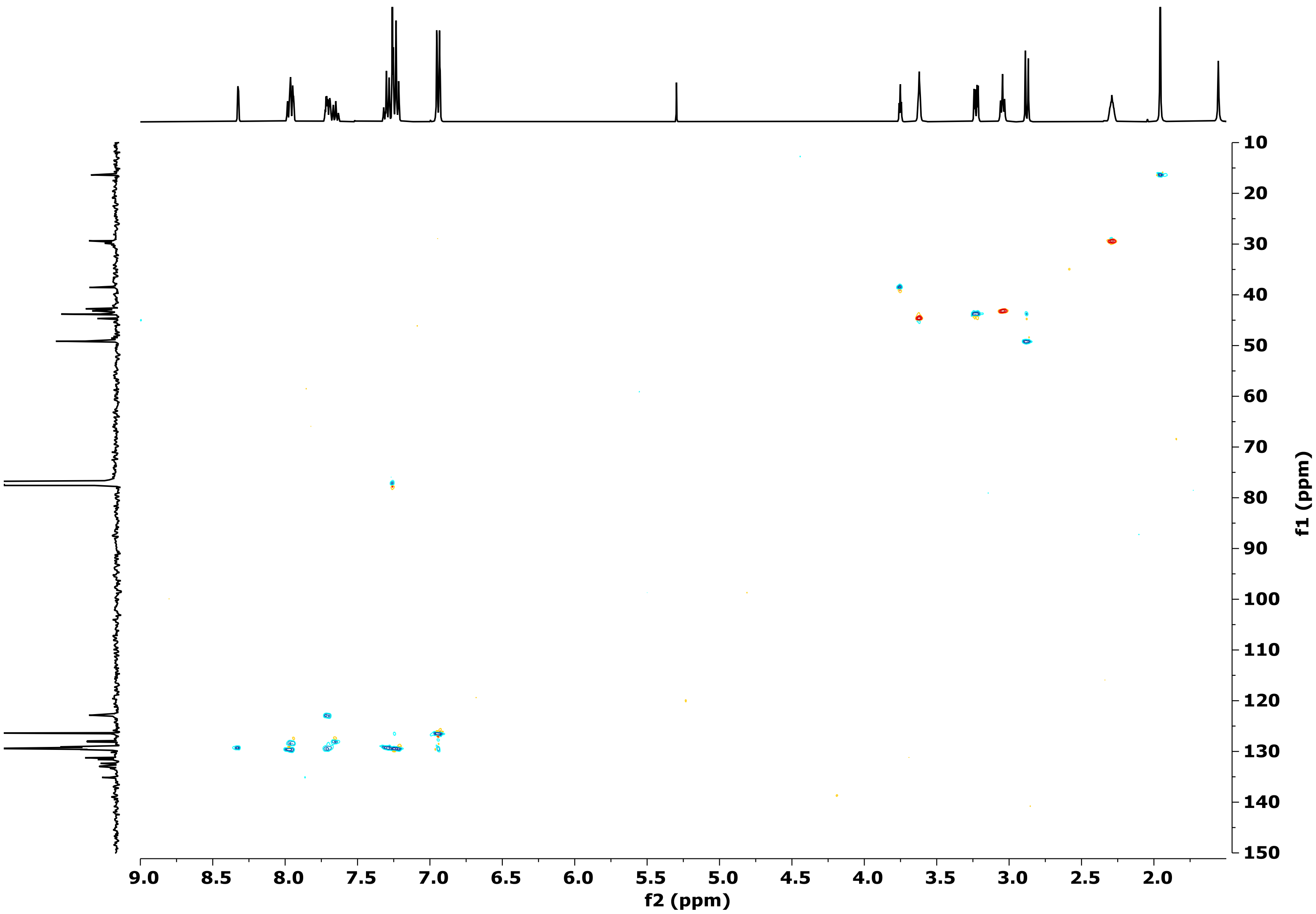

9.0
8.5
8.0
7.5
7.0
6.5
6.0
5.5
5.0
4.5
4.0
3.5
3.0
2.5
2.0
f2 (ppm)
10
20
30
40
50
60
70
80
90
100
110
120
130
140
150
f1 (ppm)

2D NMR COSY

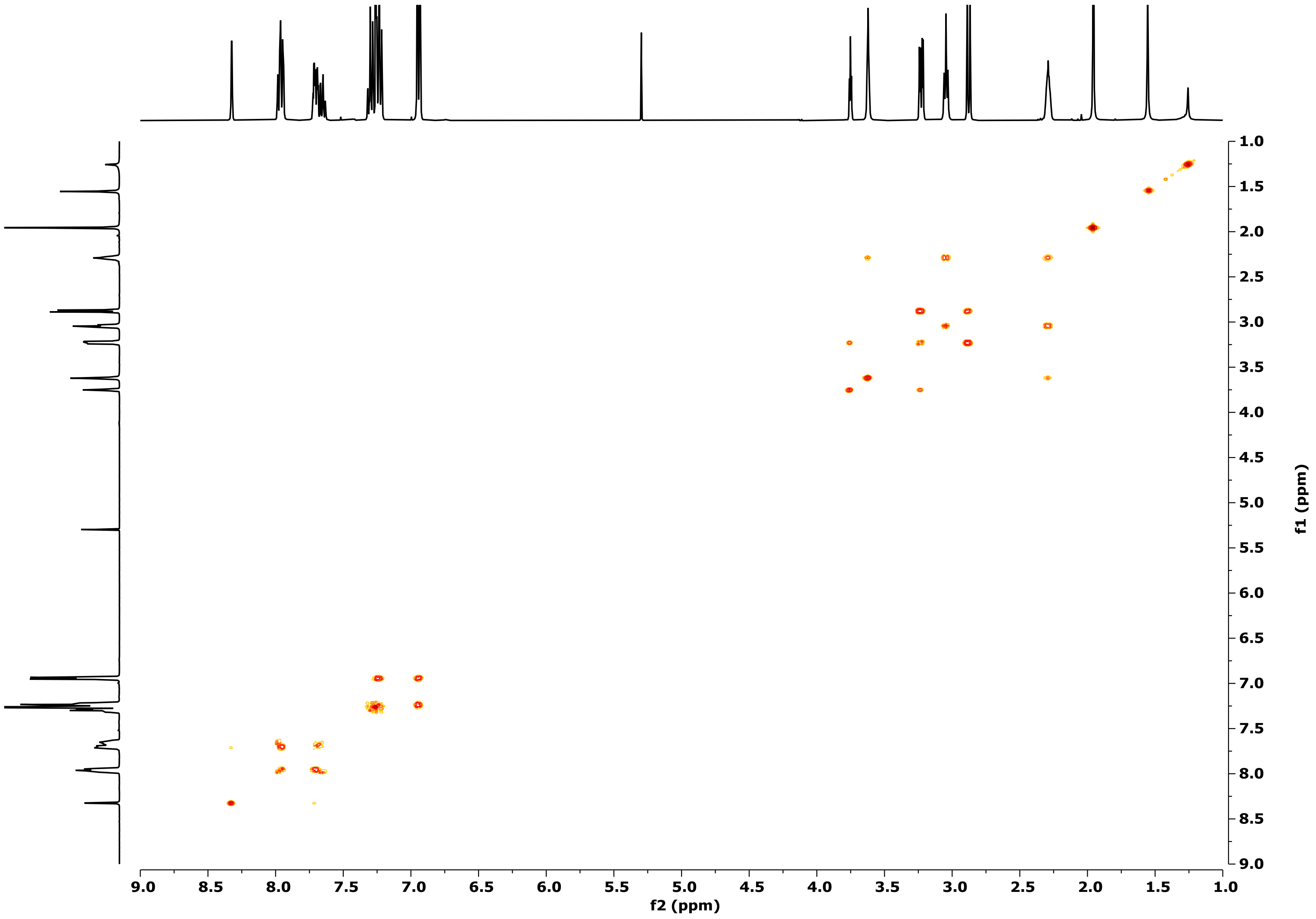

Compound 3r

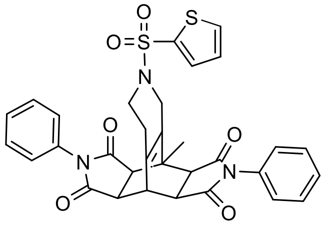

^{1}H NMR (400 MHz, $CDCl_3$)

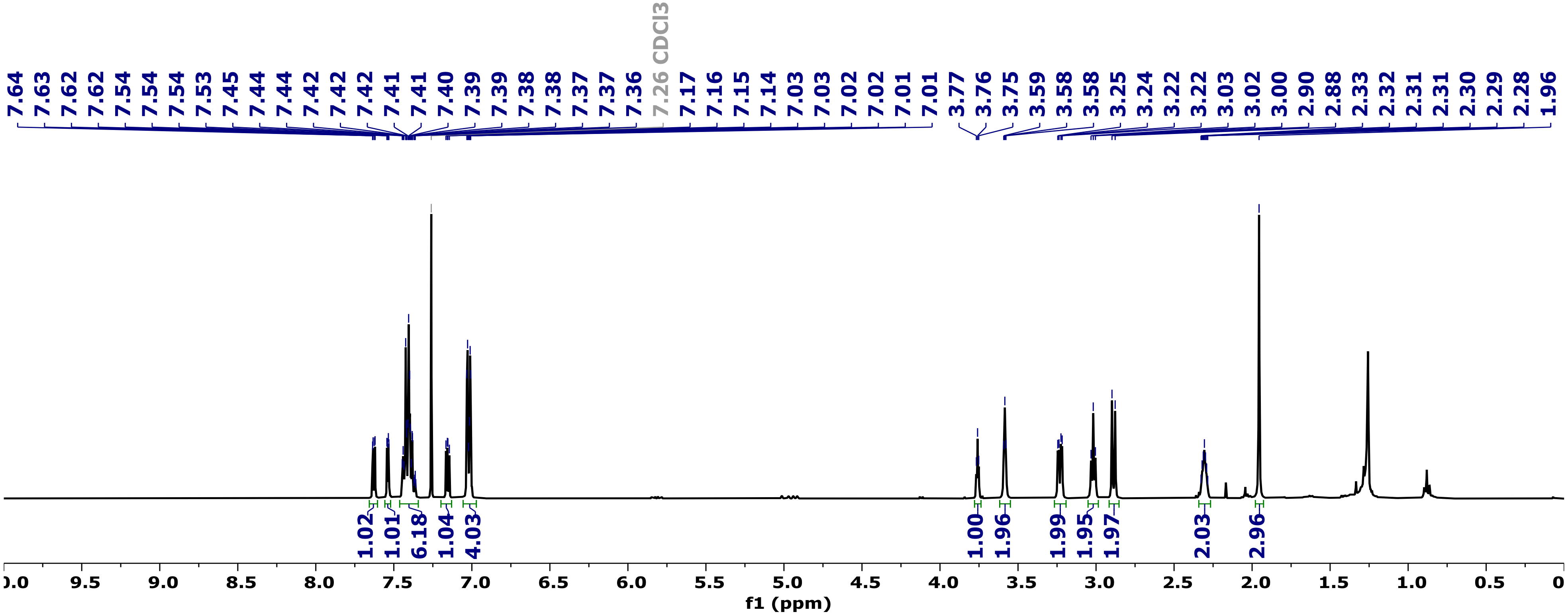

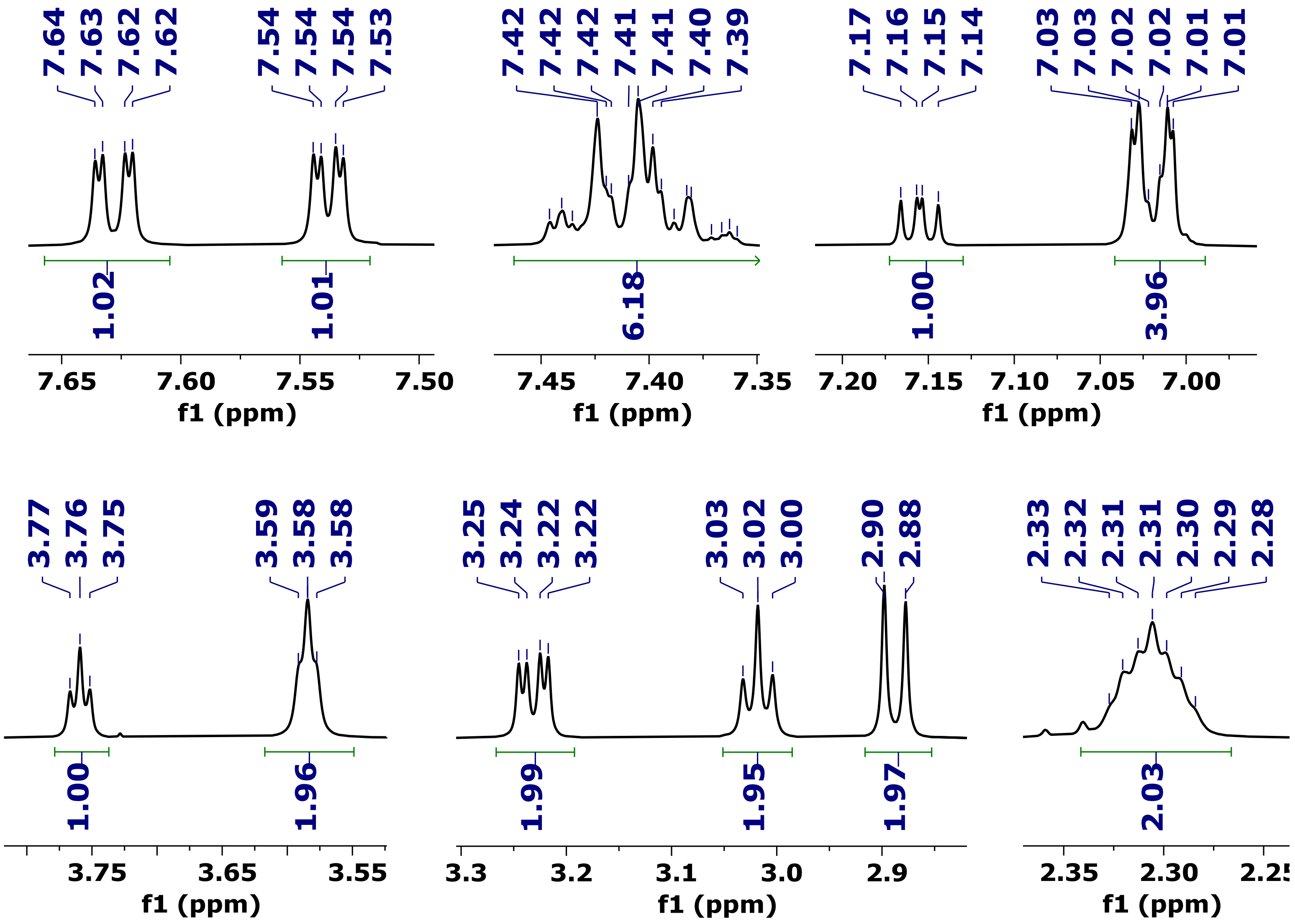
7.64 7.63 7.62 7.62 7.54 7.54 7.54 7.53
1.02 1.01
7.65 7.60 7.55 7.50
f1 (ppm)
7.42 7.42 7.42 7.41 7.41 7.40 7.39
6.18
7.45 7.40 7.35
f1 (ppm)
7.17 7.16 7.15 7.14 7.03 7.03 7.02 7.02 7.01 7.01
1.00 3.96
7.20 7.15 7.10 7.05 7.00
f1 (ppm)
3.77 3.76 3.75 3.59 3.58 3.58
1.00 1.96
3.75 3.65 3.55
f1 (ppm)
3.25 3.24 3.22 3.22 3.03 3.02 3.00 2.90 2.88
1.99 1.95 1.97
3.3 3.2 3.1 3.0 2.9
f1 (ppm)
2.33 2.32 2.31 2.31 2.30 2.29 2.28
2.03
2.35 2.30
f1 (ppm)

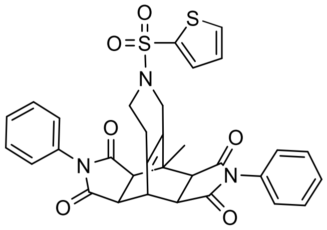

^{13}C NMR (101 MHz, $CDCl_3$)

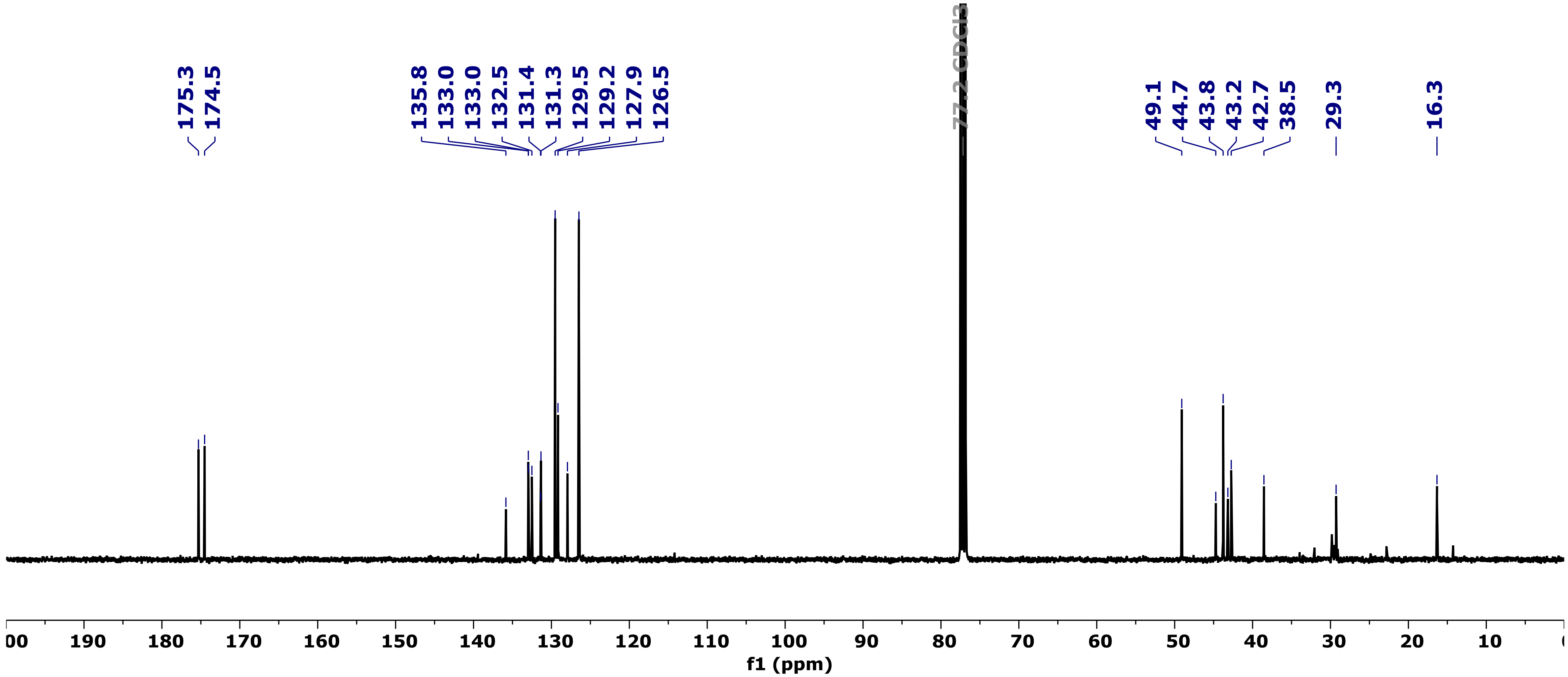

2D NMR HSQC

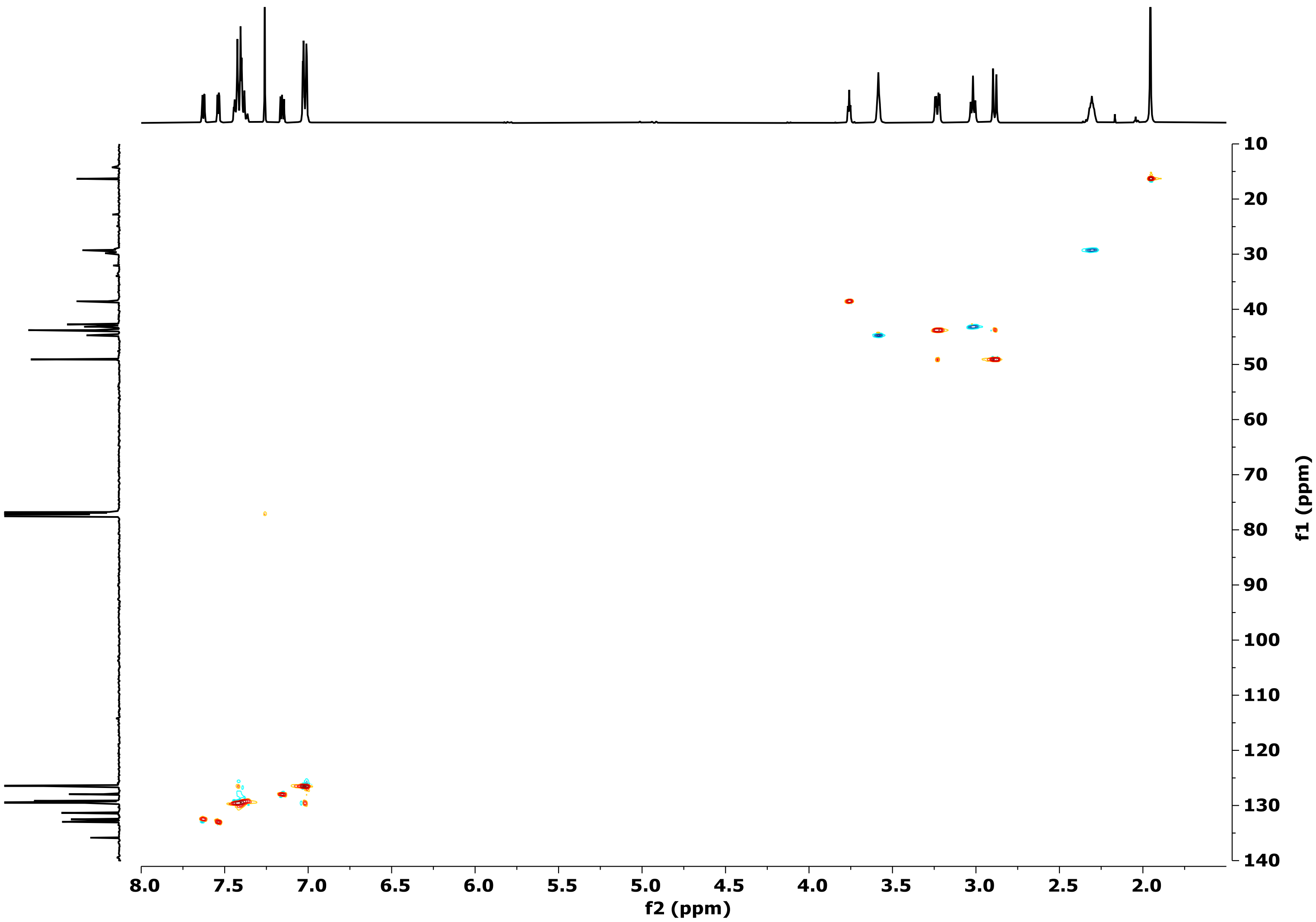

10
20
30
40
50
60
70
80
90
100
110
120
130
140
f1 (ppm)
8.0
7.5
7.0
6.5
6.0
5.5
5.0
4.5
4.0
3.5
3.0
2.5
2.0
f2 (ppm)

2D NMR COSY

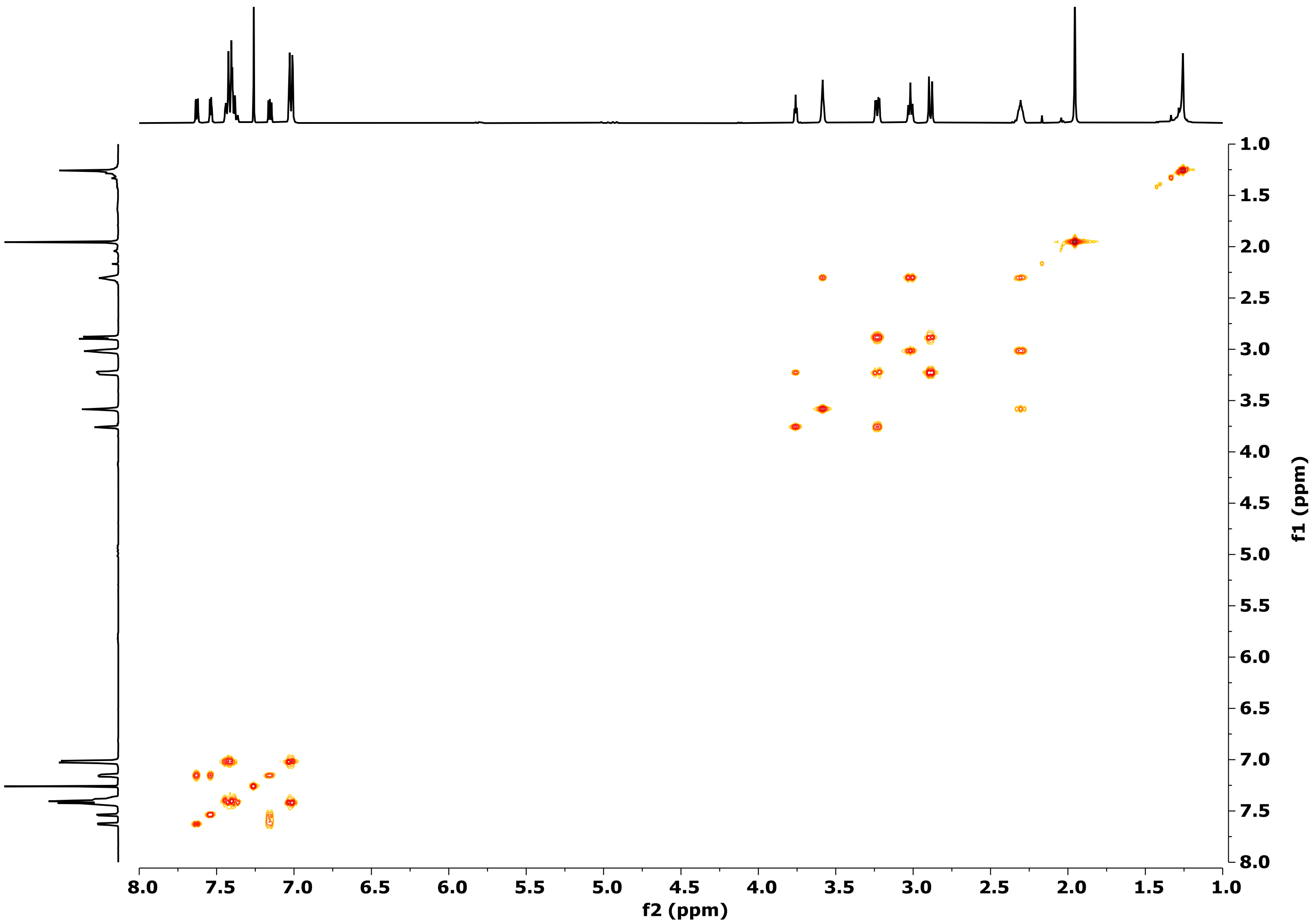

Compound 3s

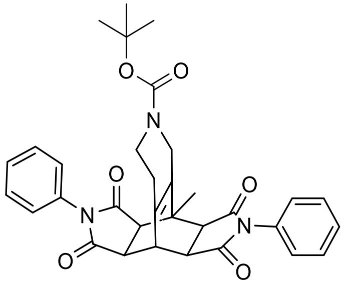

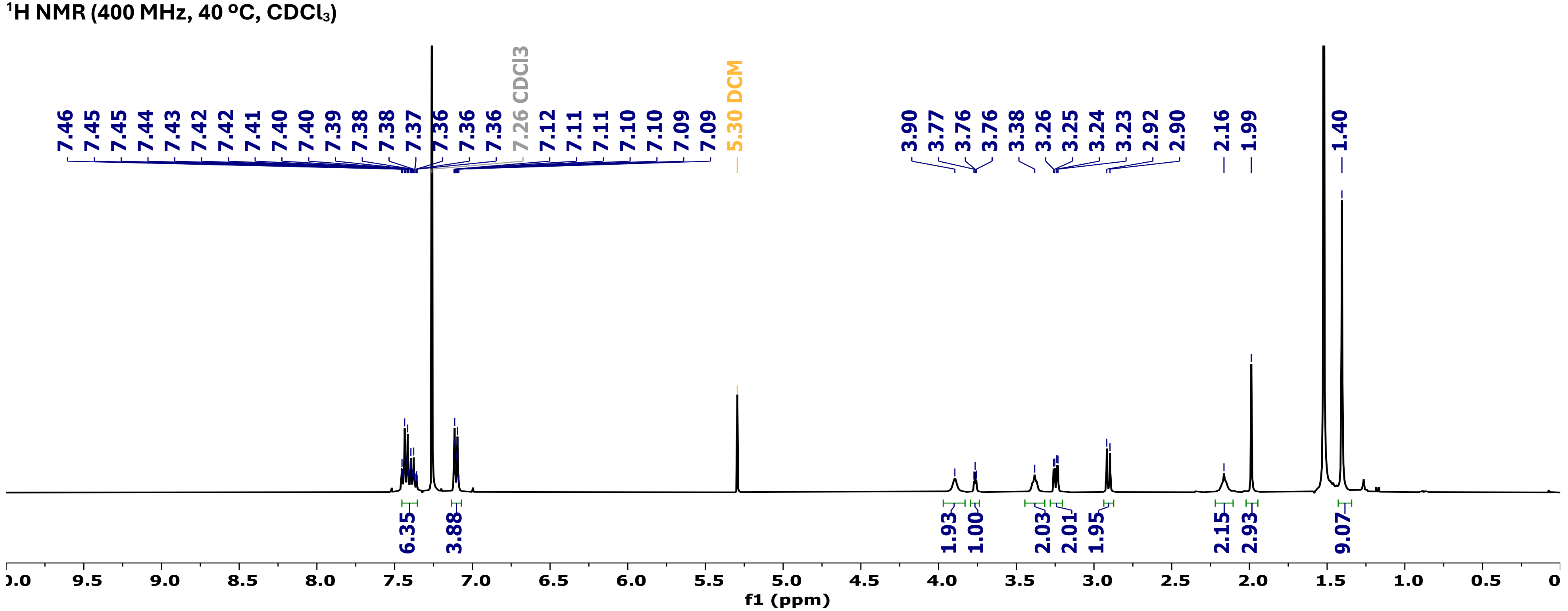
^{1}H NMR (400 MHz, 40 ºC, CDCl$_3$)
7.46
7.45
7.45
7.44
7.43
7.42
7.42
7.41
7.40
7.40
7.39
7.38
7.38
7.37
7.36
7.36
7.36
7.26 CDCl3
7.12
7.11
7.11
7.10
7.10
7.09
7.09
5.30 DCM
3.90
3.77
3.76
3.76
3.38
3.26
3.25
3.24
3.23
2.92
2.90
2.16
1.99
1.40
6.35
3.88
1.93
1.00
2.03
2.01
1.95
2.15
2.93
9.07
f1 (ppm)

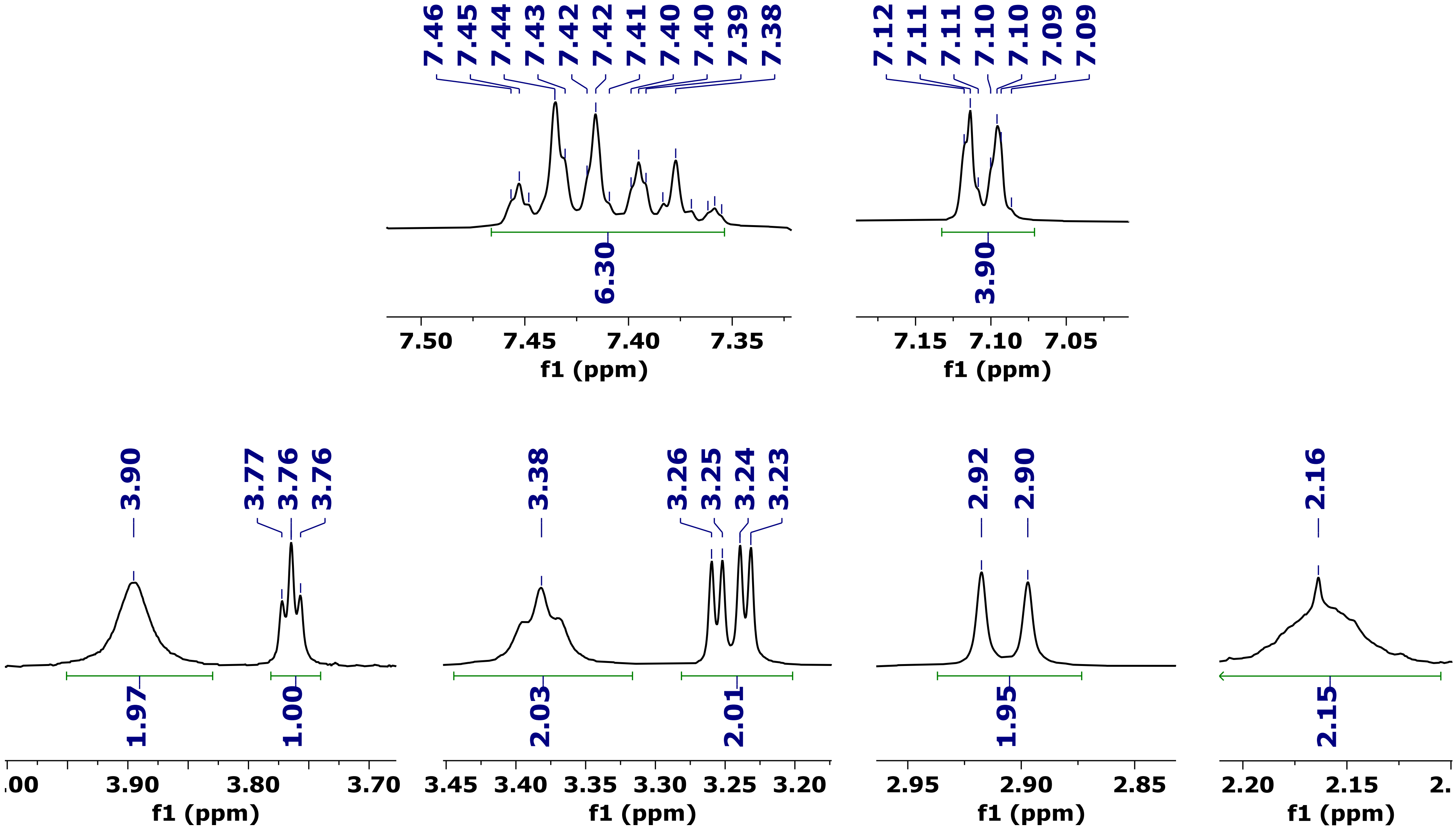
7.46 7.45 7.44 7.43 7.42 7.42 7.41 7.40 7.40 7.39 7.38
6.30
7.50 7.45 7.40 7.35
f1 (ppm)
7.12 7.11 7.11 7.10 7.10 7.09 7.09
3.90
7.15 7.10 7.05
f1 (ppm)
3.90
3.77 3.76 3.76
1.97
1.00
.00 3.90 3.80 3.70
f1 (ppm)
3.38
3.26 3.25 3.24 3.23
2.03
2.01
3.45 3.40 3.35 3.30 3.25 3.20
f1 (ppm)
2.92 2.90
1.95
2.95 2.90 2.85
f1 (ppm)
2.16
2.15
2.20 2.15 2.
f1 (ppm)

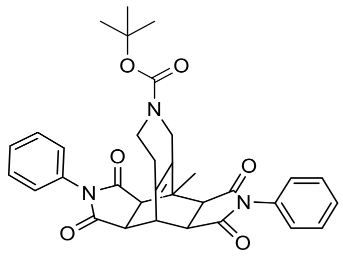

^{13}C NMR (101 MHz, $CDCl_3$)

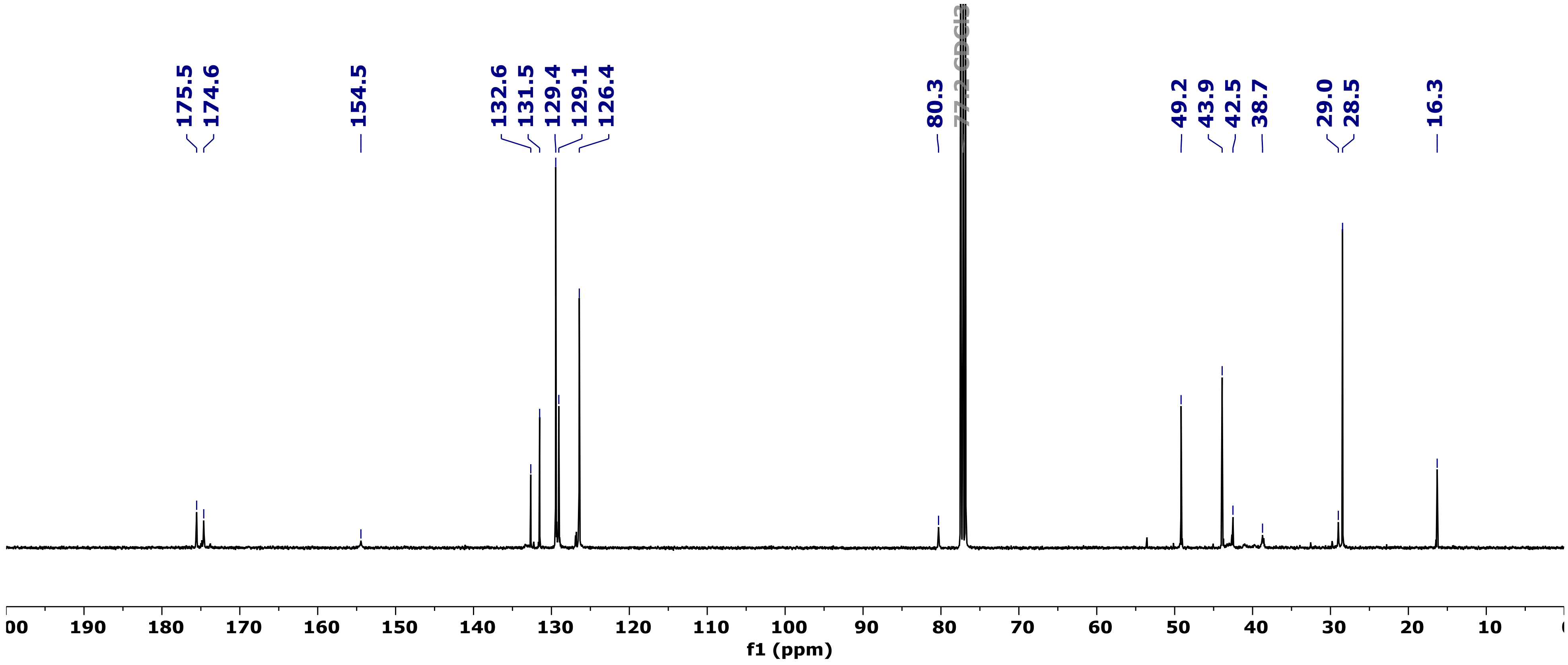

2D NMR HSQC

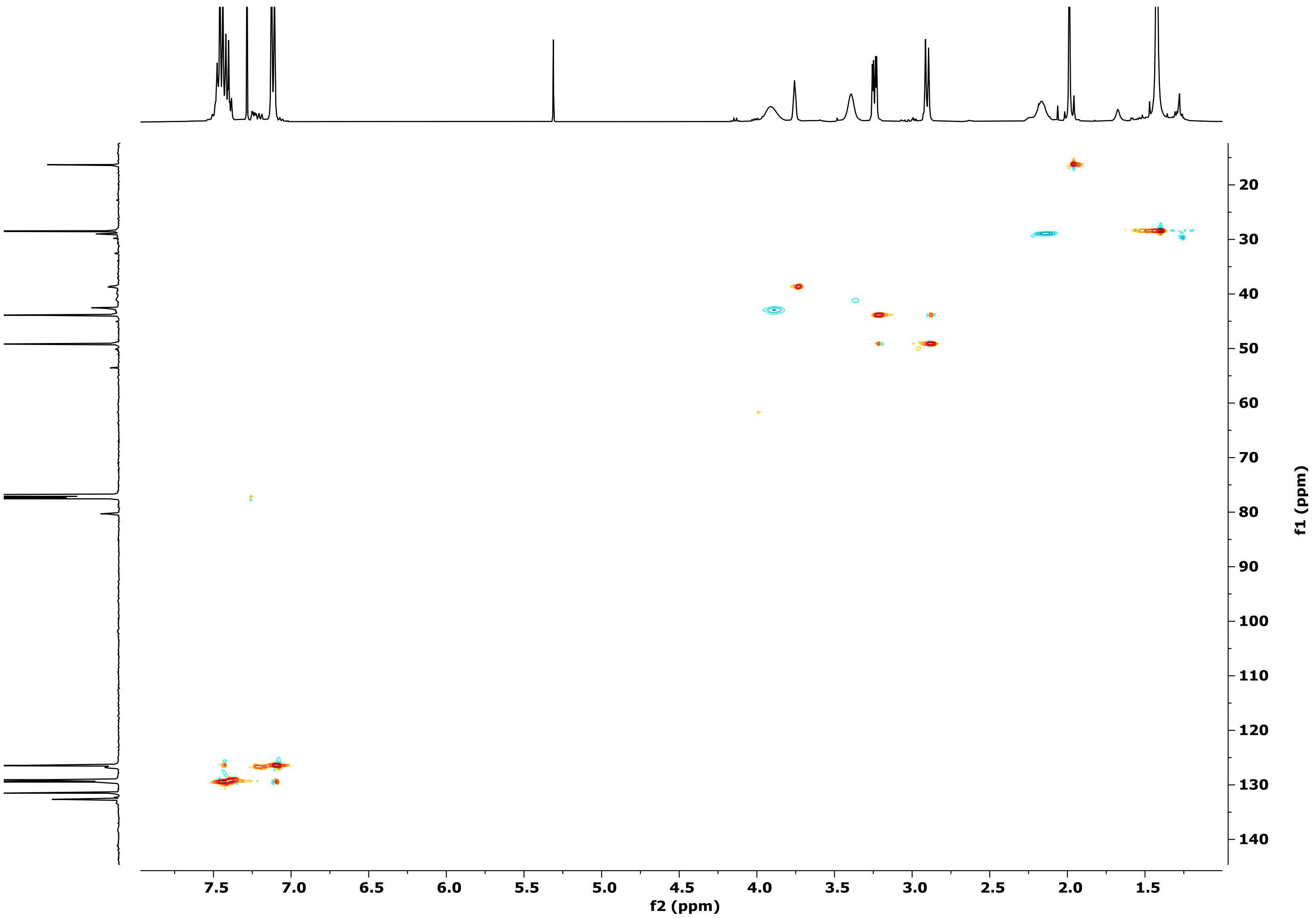

7.5
7.0
6.5
6.0
5.5
5.0
4.5
4.0
3.5
3.0
2.5
2.0
1.5
f2 (ppm)
20
30
40
50
60
70
80
90
100
110
120
130
140
f1 (ppm)

2D NMR COSY

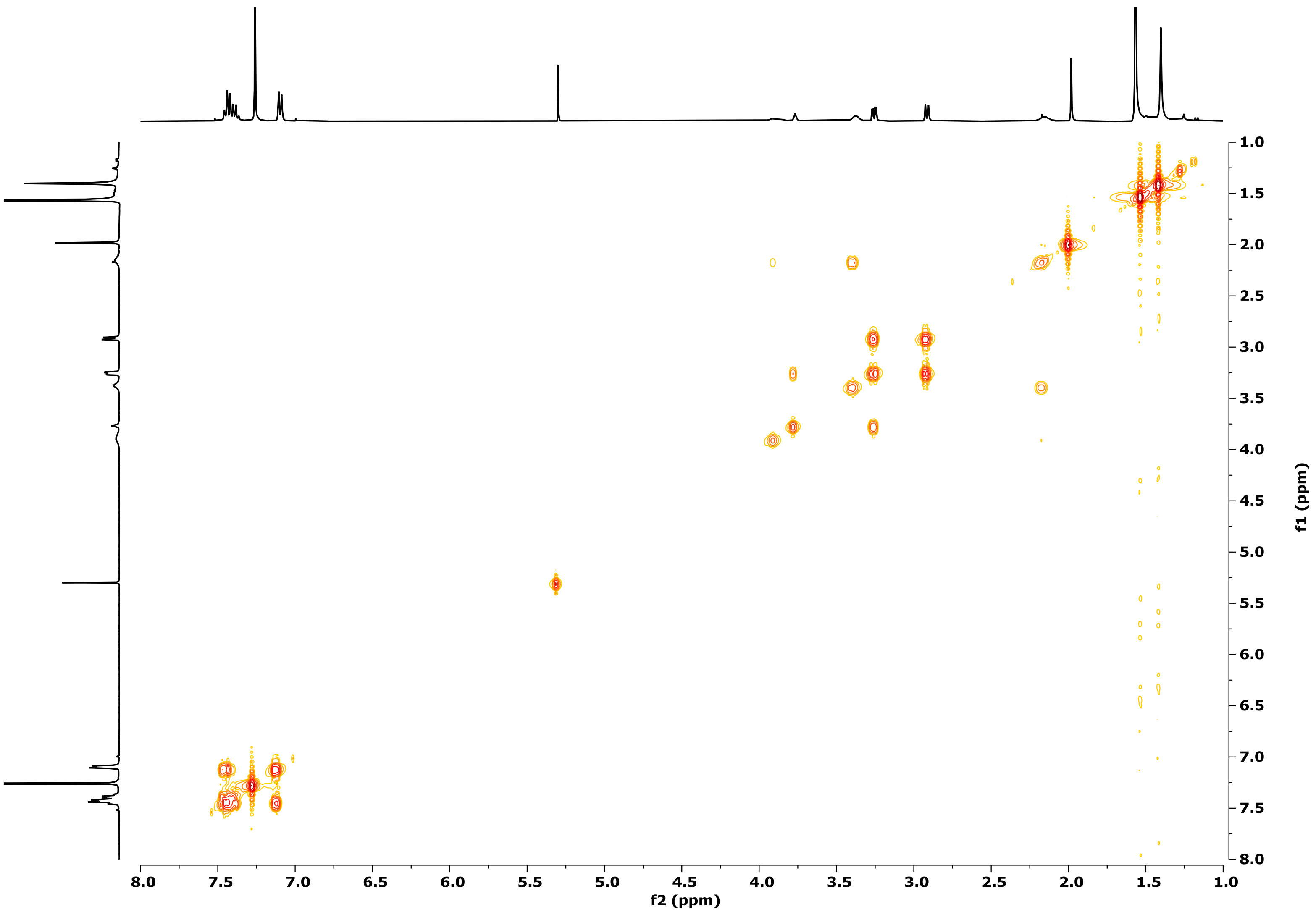

Compound 3t

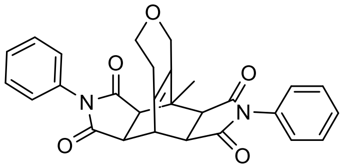

^{1}H NMR (400 MHz, CDCl$_3$)

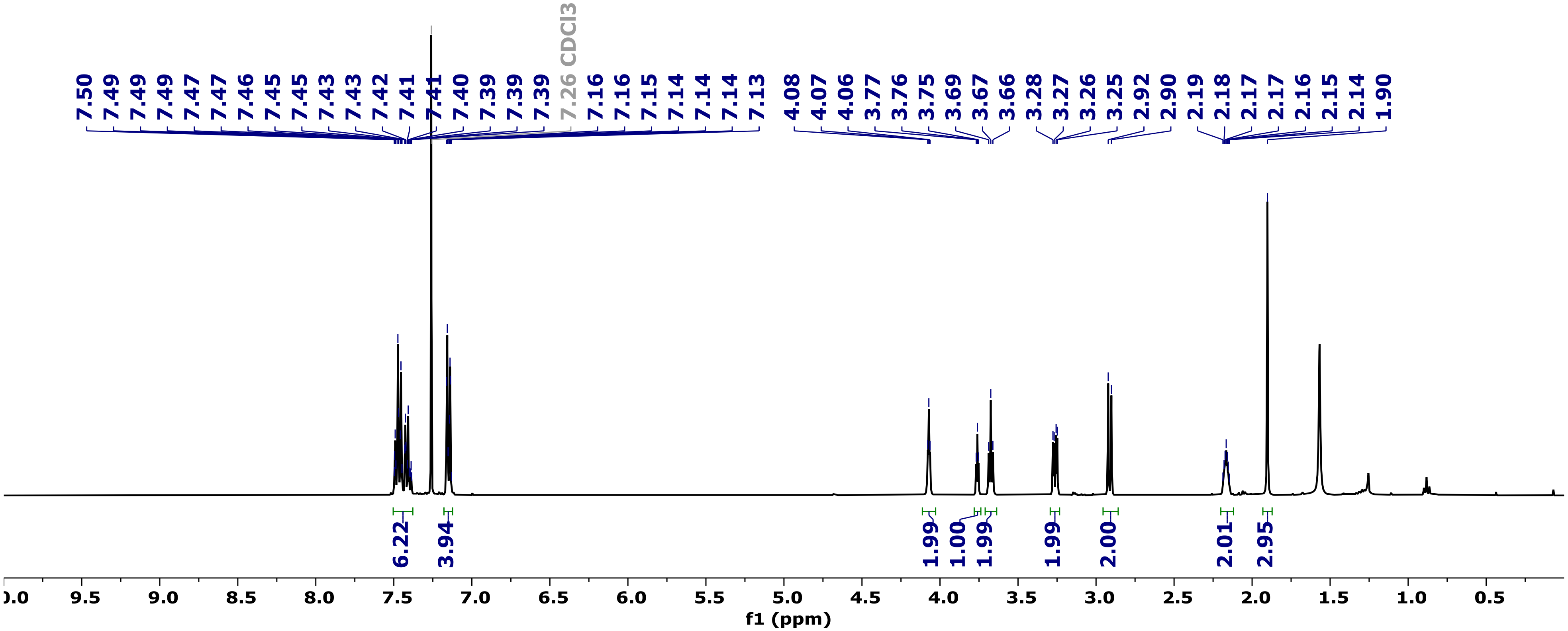

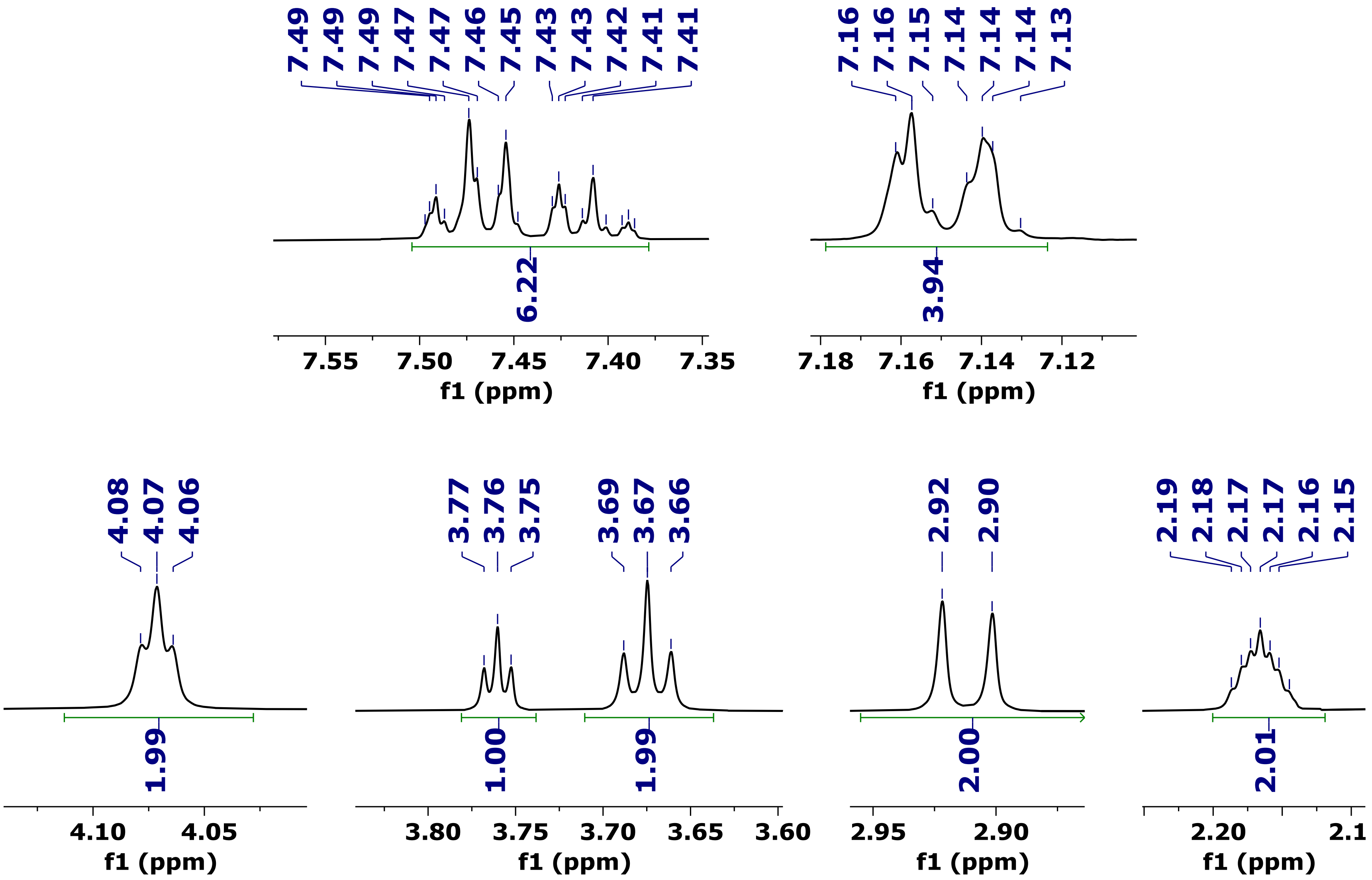

7.49 7.49 7.49 7.47 7.47 7.46 7.45 7.43 7.43 7.42 7.41 7.41
6.22
7.55 7.50 7.45 7.40 7.35
f1 (ppm)
7.16 7.16 7.15 7.14 7.14 7.14 7.13
3.94
7.18 7.16 7.14 7.12
f1 (ppm)
4.08 4.07 4.06
1.99
4.10 4.05
f1 (ppm)
3.77 3.76 3.75 3.69 3.67 3.66
1.00 1.99
3.80 3.75 3.70 3.65 3.60
f1 (ppm)
2.92 2.90
2.00
2.95 2.90
f1 (ppm)
2.19 2.18 2.17 2.17 2.16 2.15
2.01
2.20 2.1
f1 (ppm)

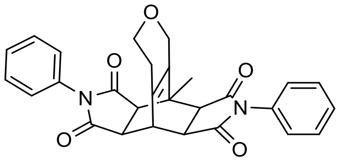

^{13}C NMR (101 MHz, $CDCl_3$)

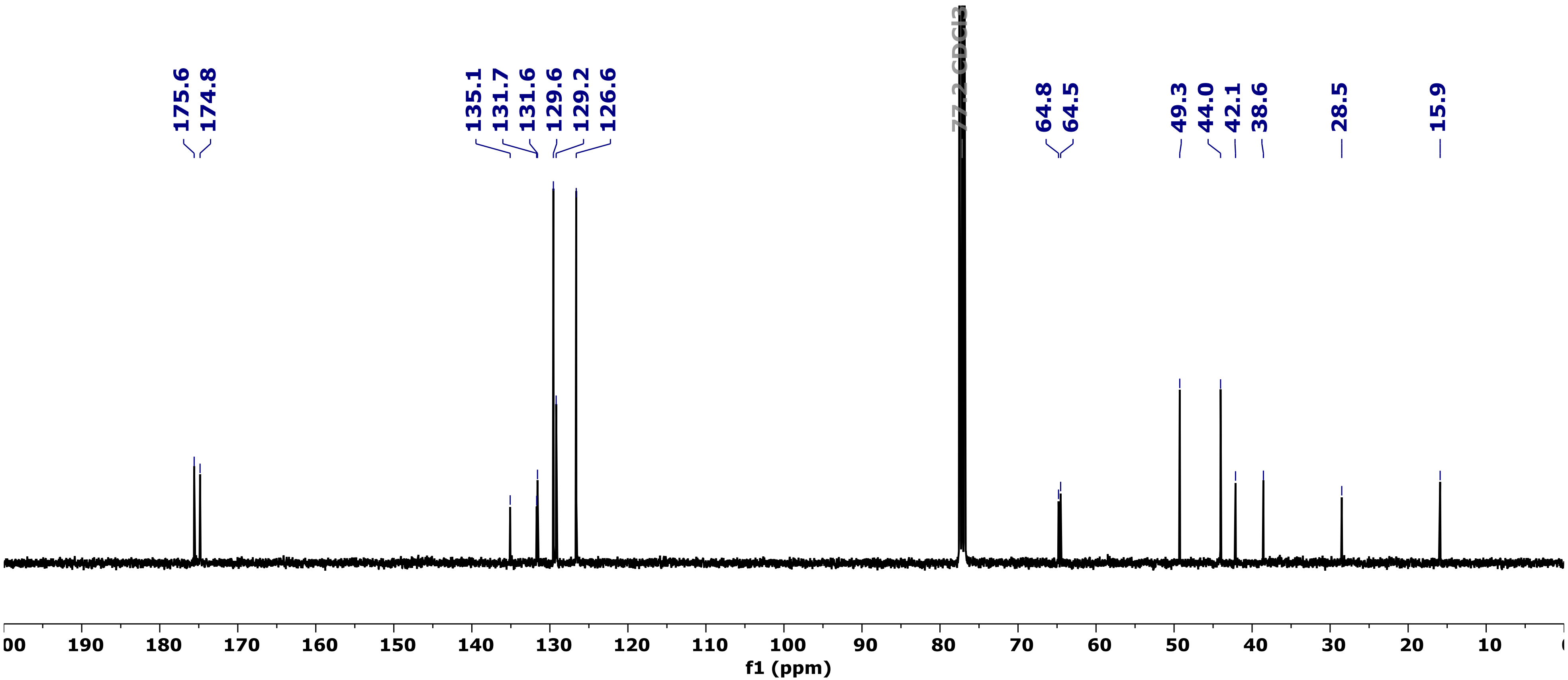

2D NMR HSQC

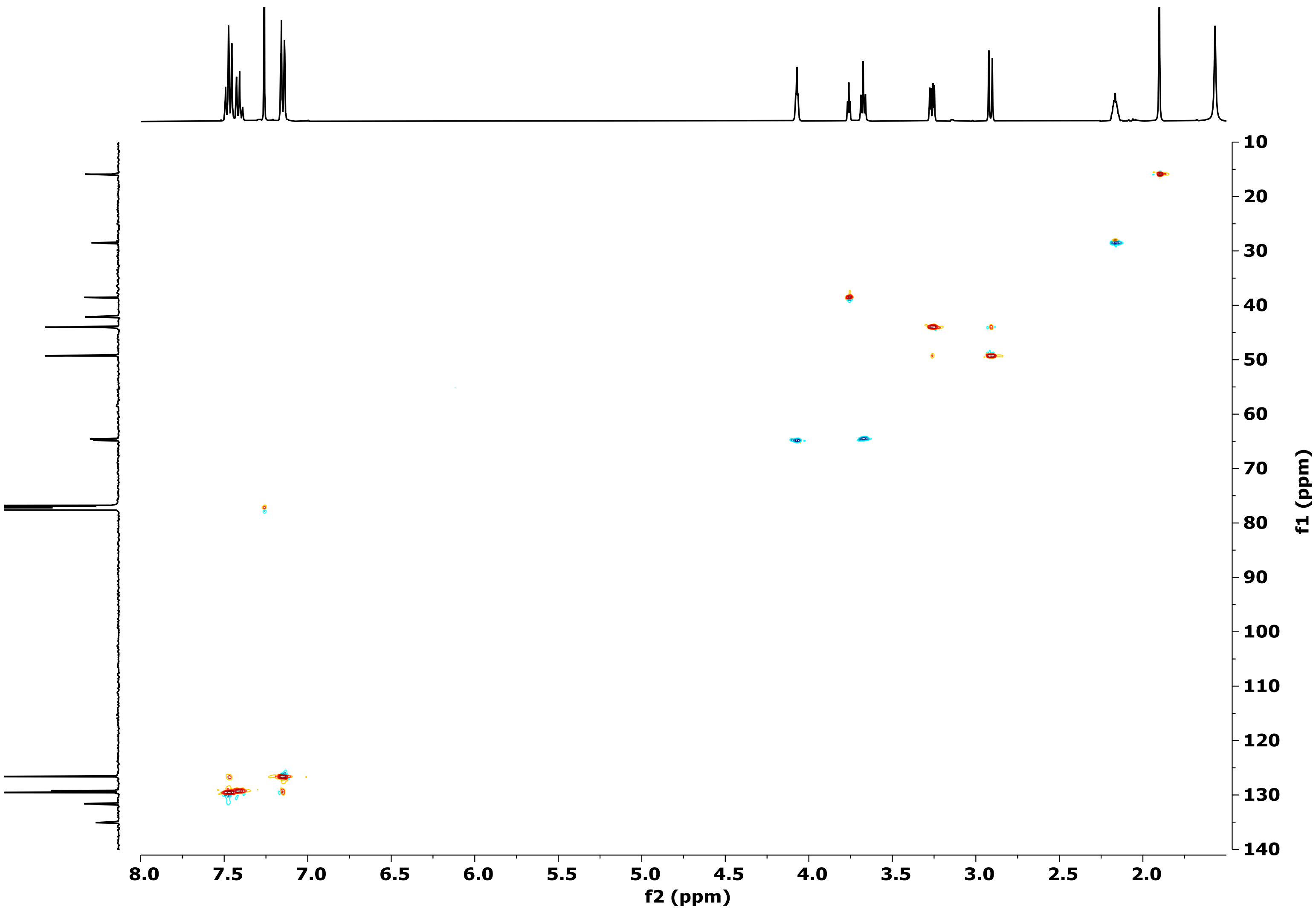

8.0
7.5
7.0
6.5
6.0
5.5
5.0
4.5
4.0
3.5
3.0
2.5
2.0
f2 (ppm)
10
20
30
40
50
60
70
80
90
100
110
120
130
140
f1 (ppm)

2D NMR COSY

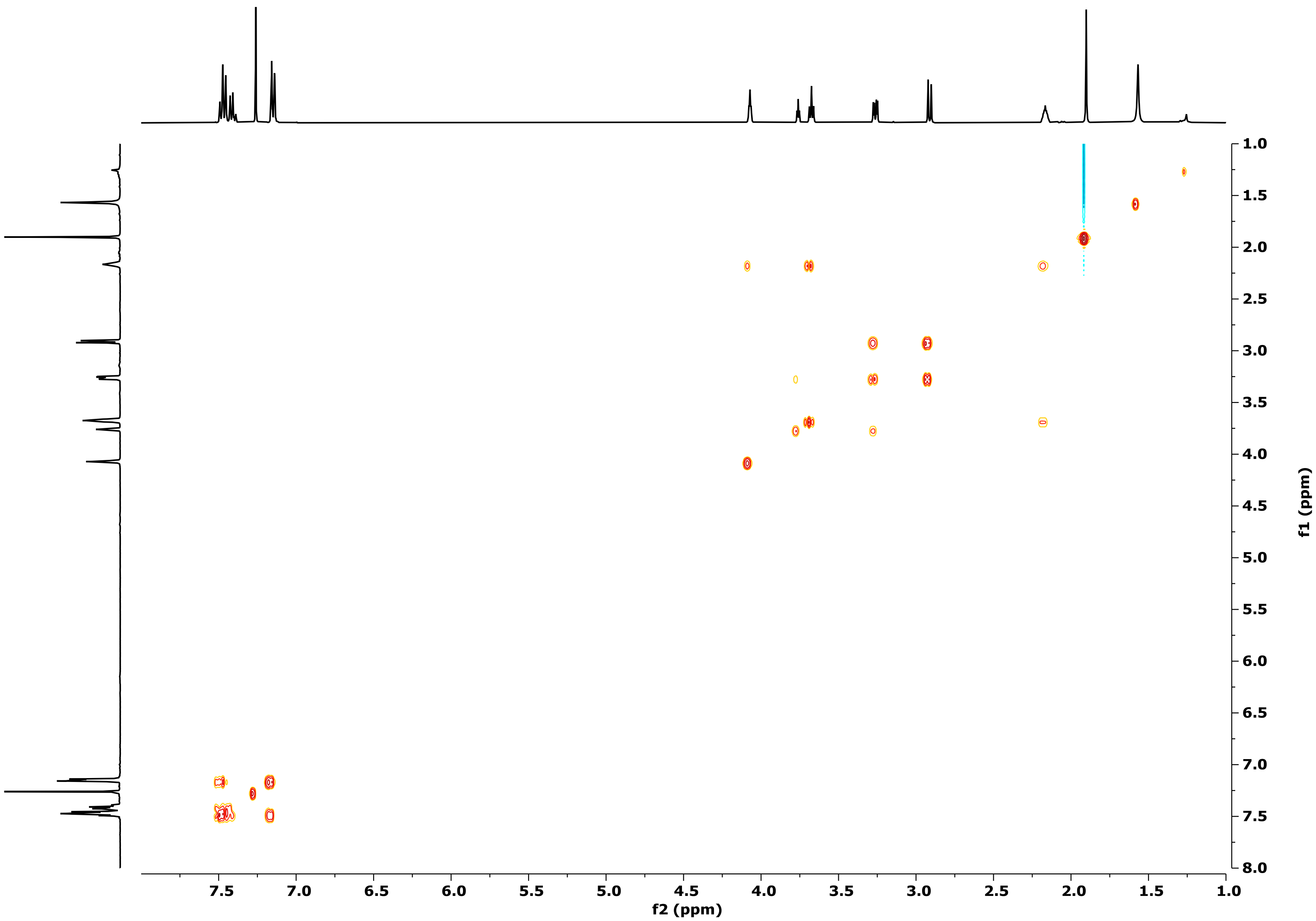

Compound 3u

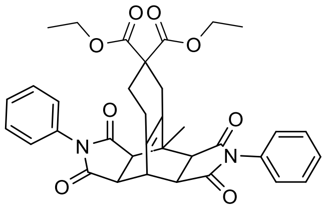

^{1}H NMR (400 MHz, $CDCl_3$)

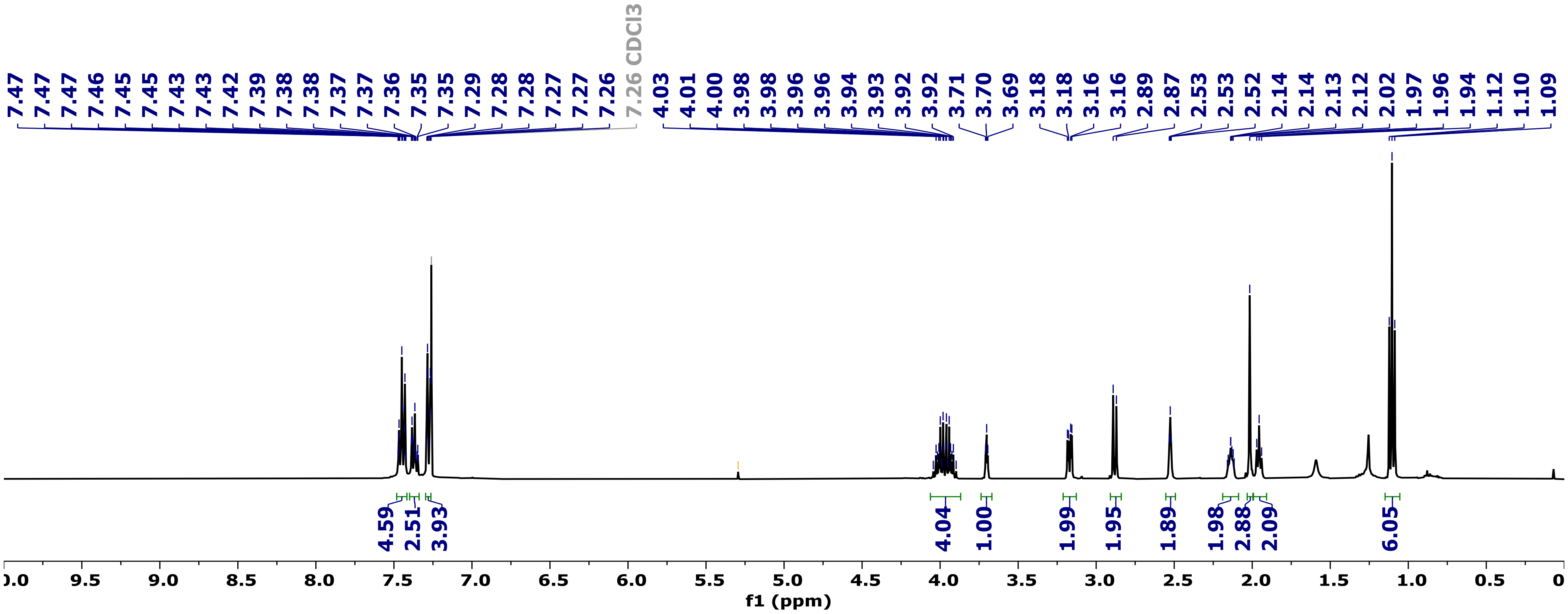

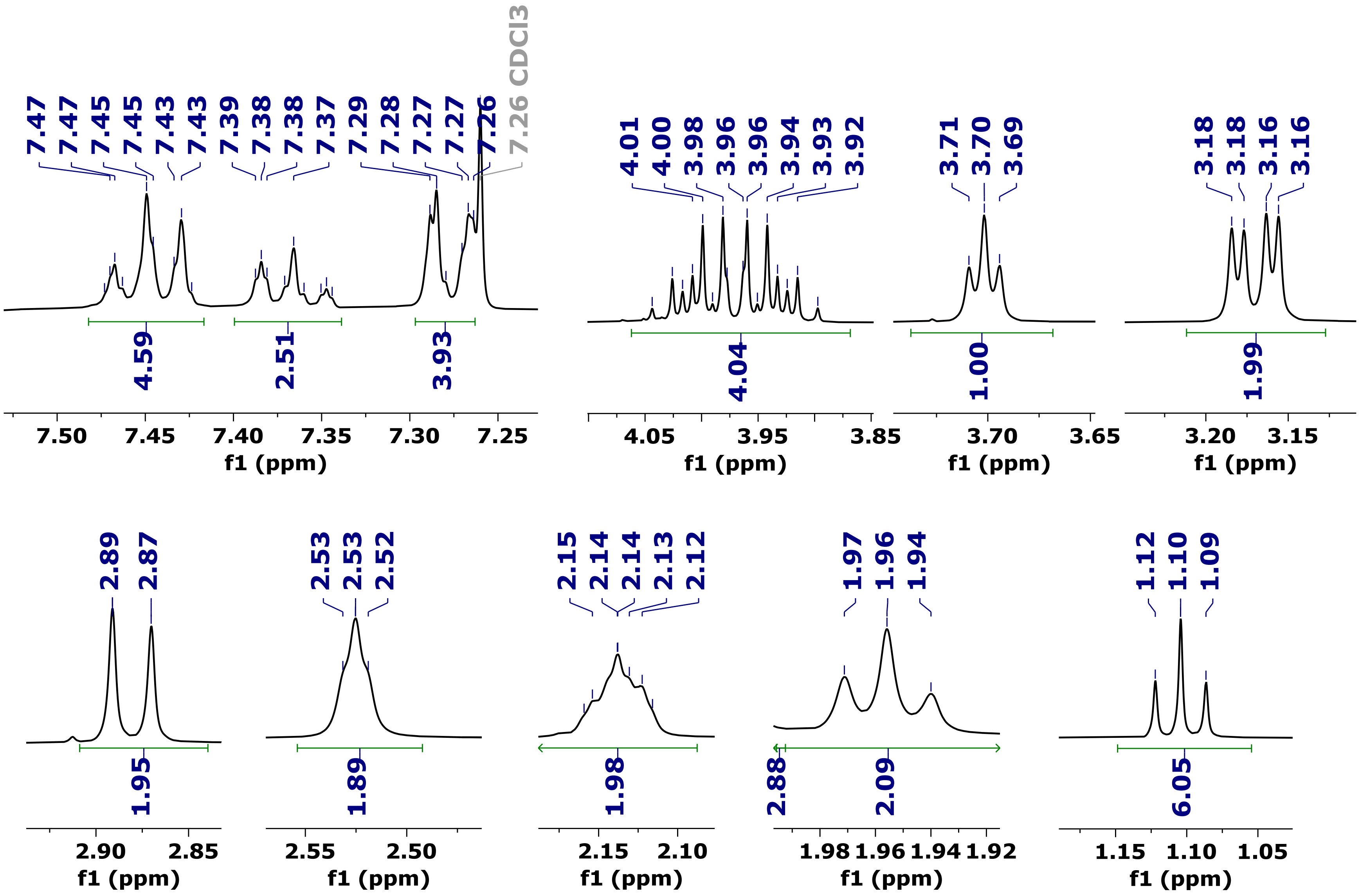
7.47
7.47
7.45
7.45
7.43
7.43
7.39
7.38
7.38
7.37
7.29
7.28
7.27
7.27
7.26
7.26 CDCl3
4.59
2.51
3.93
7.50 7.45 7.40 7.35 7.30 7.25
f1 (ppm)
4.01
4.00
3.98
3.96
3.96
3.94
3.93
3.92
4.04
4.05 3.95 3.85
f1 (ppm)
3.71
3.70
3.69
1.00
3.70 3.65
f1 (ppm)
3.18
3.18
3.16
3.16
1.99
3.20 3.15
f1 (ppm)
2.89
2.87
1.95
2.90 2.85
f1 (ppm)
2.53
2.53
2.52
1.89
2.55 2.50
f1 (ppm)
2.15
2.14
2.14
2.13
2.12
1.98
2.15 2.10
f1 (ppm)
1.97
1.96
1.94
2.88
2.09
1.98 1.96 1.94 1.92
f1 (ppm)
1.12
1.10
1.09
6.05
1.15 1.10 1.05
f1 (ppm)

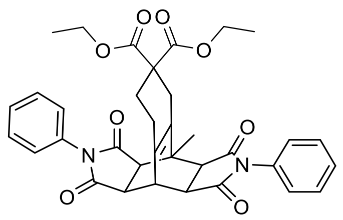

^{13}C NMR (101 MHz, $CDCl_3$)

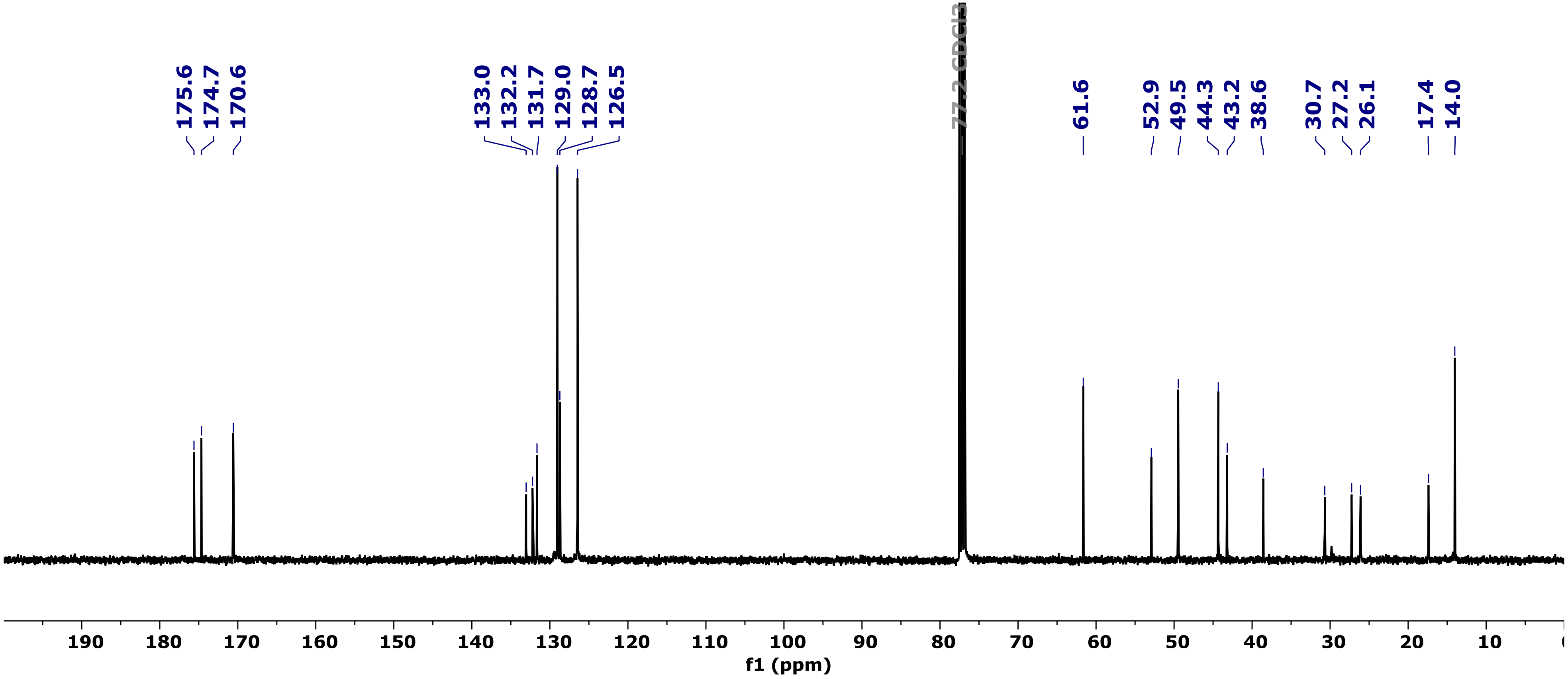

2D NMR HSQC

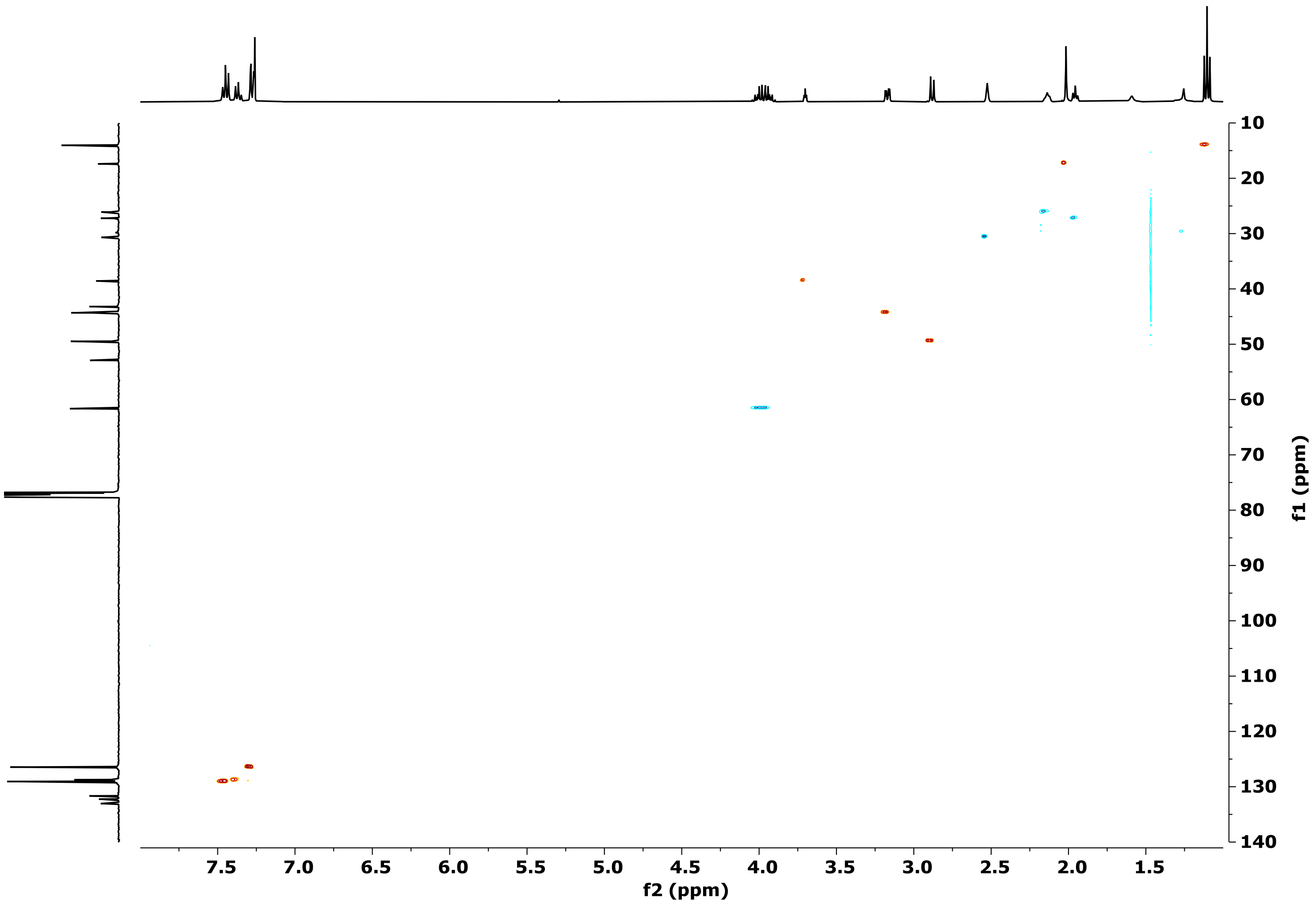

7.5
7.0
6.5
6.0
5.5
5.0
4.5
4.0
3.5
3.0
2.5
2.0
1.5
f2 (ppm)
10
20
30
40
50
60
70
80
90
100
110
120
130
140
f1 (ppm)

2D NMR COSY

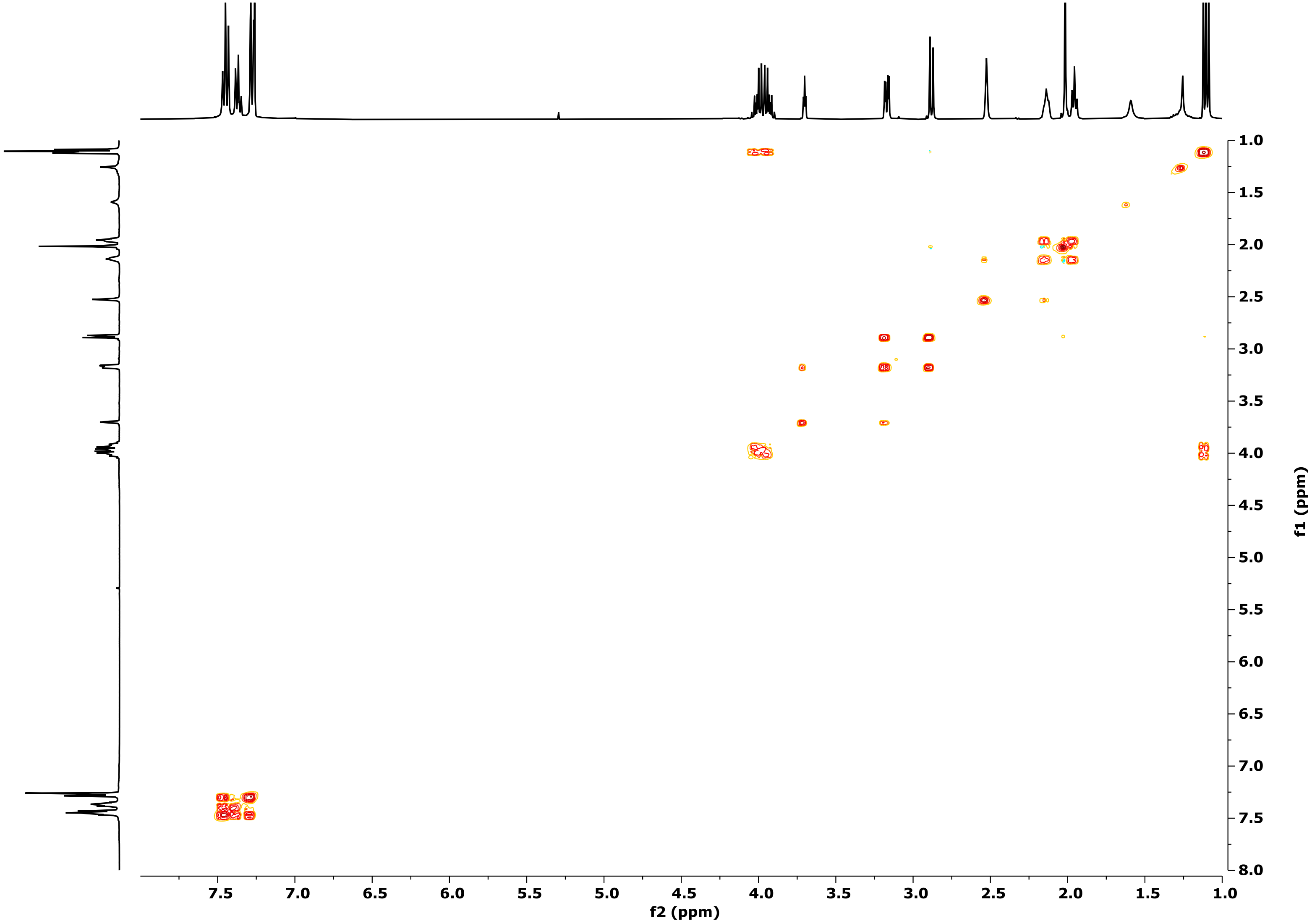

1.0
1.5
2.0
2.5
3.0
3.5
4.0
4.5
5.0
5.5
6.0
6.5
7.0
7.5
8.0
f1 (ppm)
7.5
7.0
6.5
6.0
5.5
5.0
4.5
4.0
3.5
3.0
2.5
2.0
1.5
1.0
f2 (ppm)

Compound 3v

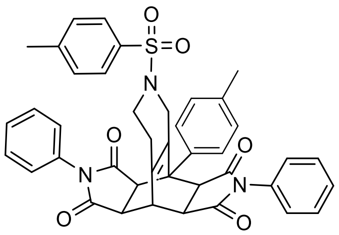

^{1}H NMR (400 MHz, $CDCl_3$)

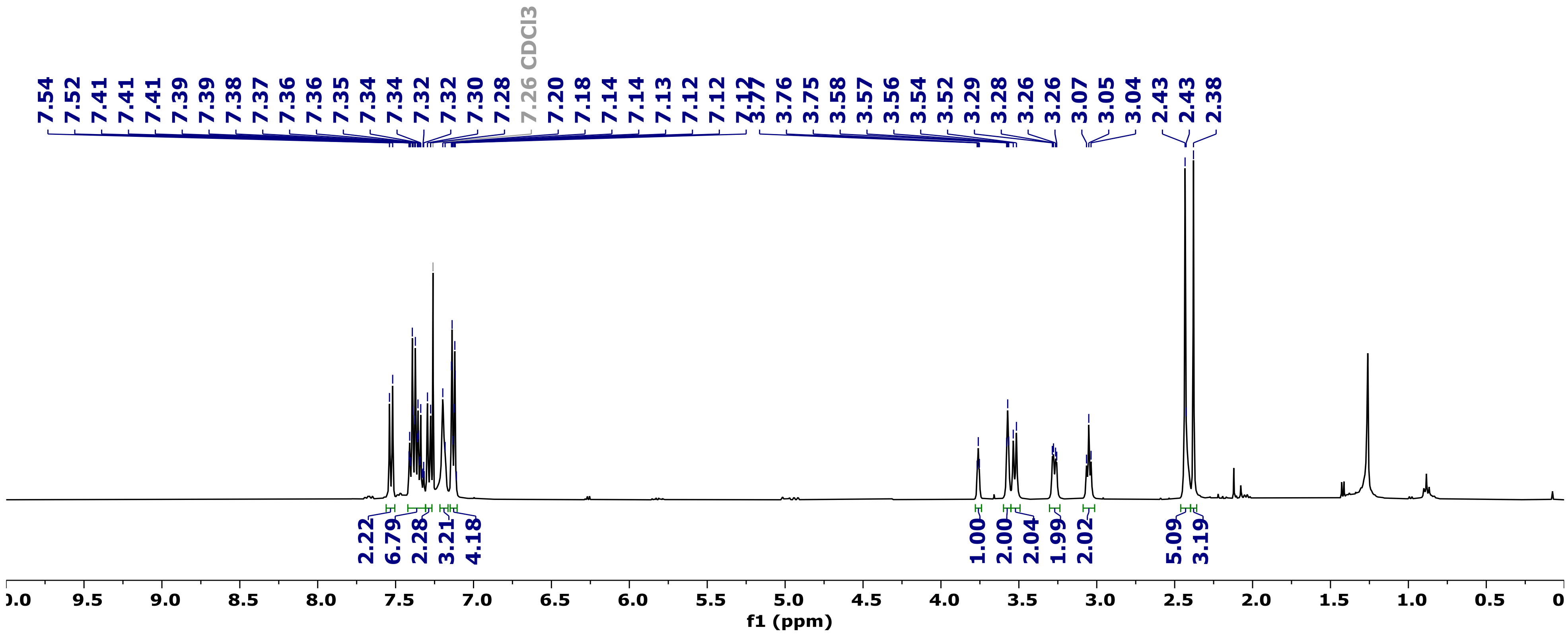

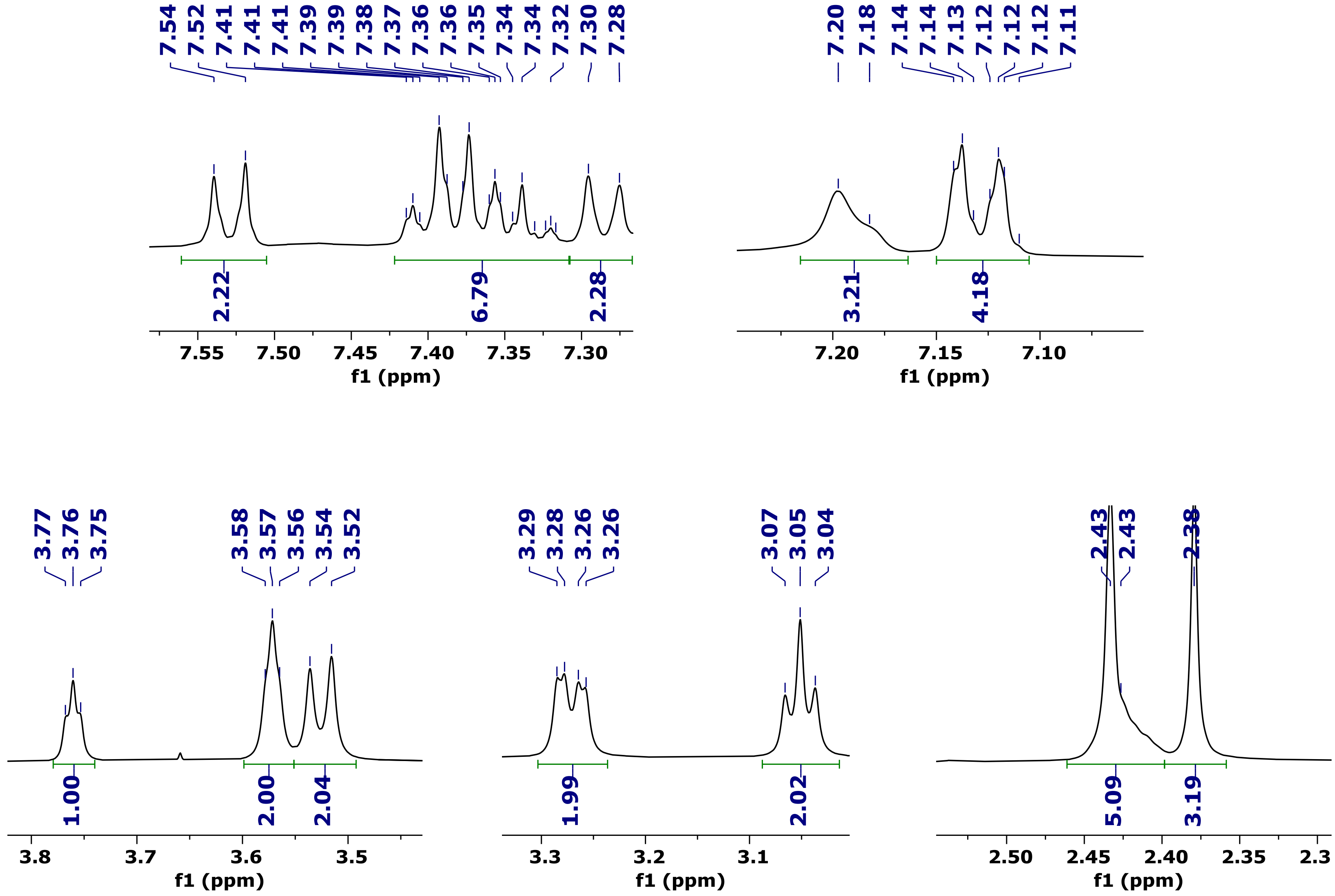
7.54 7.52 7.41 7.41 7.41 7.39 7.39 7.38 7.37 7.36 7.36 7.35 7.34 7.34 7.32 7.30 7.28
2.22 6.79 2.28
7.55 7.50 7.45 7.40 7.35 7.30
f1 (ppm)
7.20 7.18 7.14 7.14 7.13 7.12 7.12 7.12 7.11
3.21 4.18
7.20 7.15 7.10
f1 (ppm)
3.77 3.76 3.75 3.58 3.57 3.56 3.54 3.52
1.00 2.00 2.04
3.8 3.7 3.6 3.5
f1 (ppm)
3.29 3.28 3.26 3.26 3.07 3.05 3.04
1.99 2.02
3.3 3.2 3.1
f1 (ppm)
2.43 2.43 2.38
5.09 3.19
2.50 2.45 2.40 2.35 2.3
f1 (ppm)

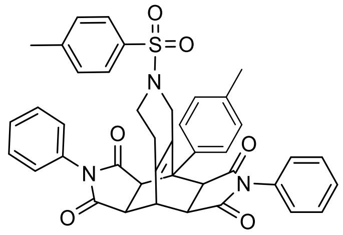

^{13}C NMR (101 MHz, $CDCl_3$)

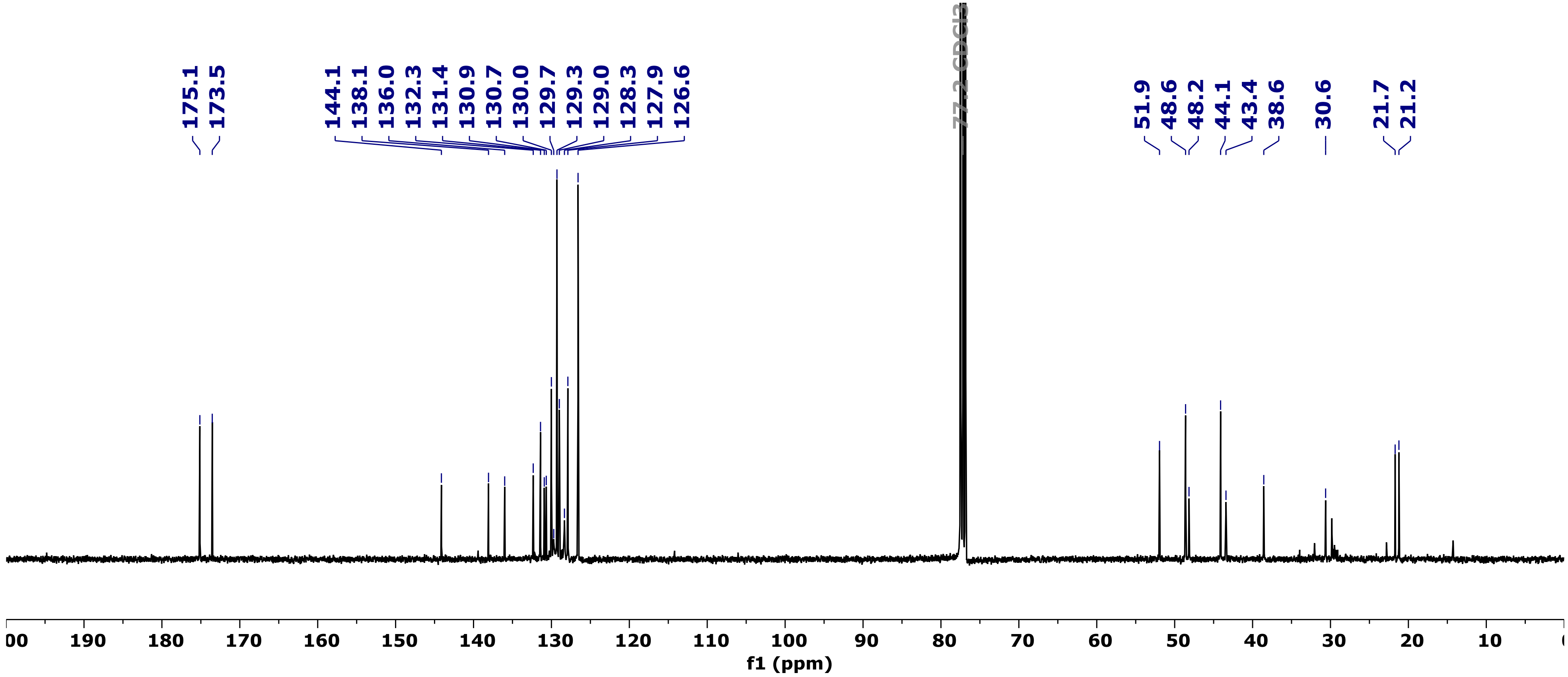

2D NMR HSQC

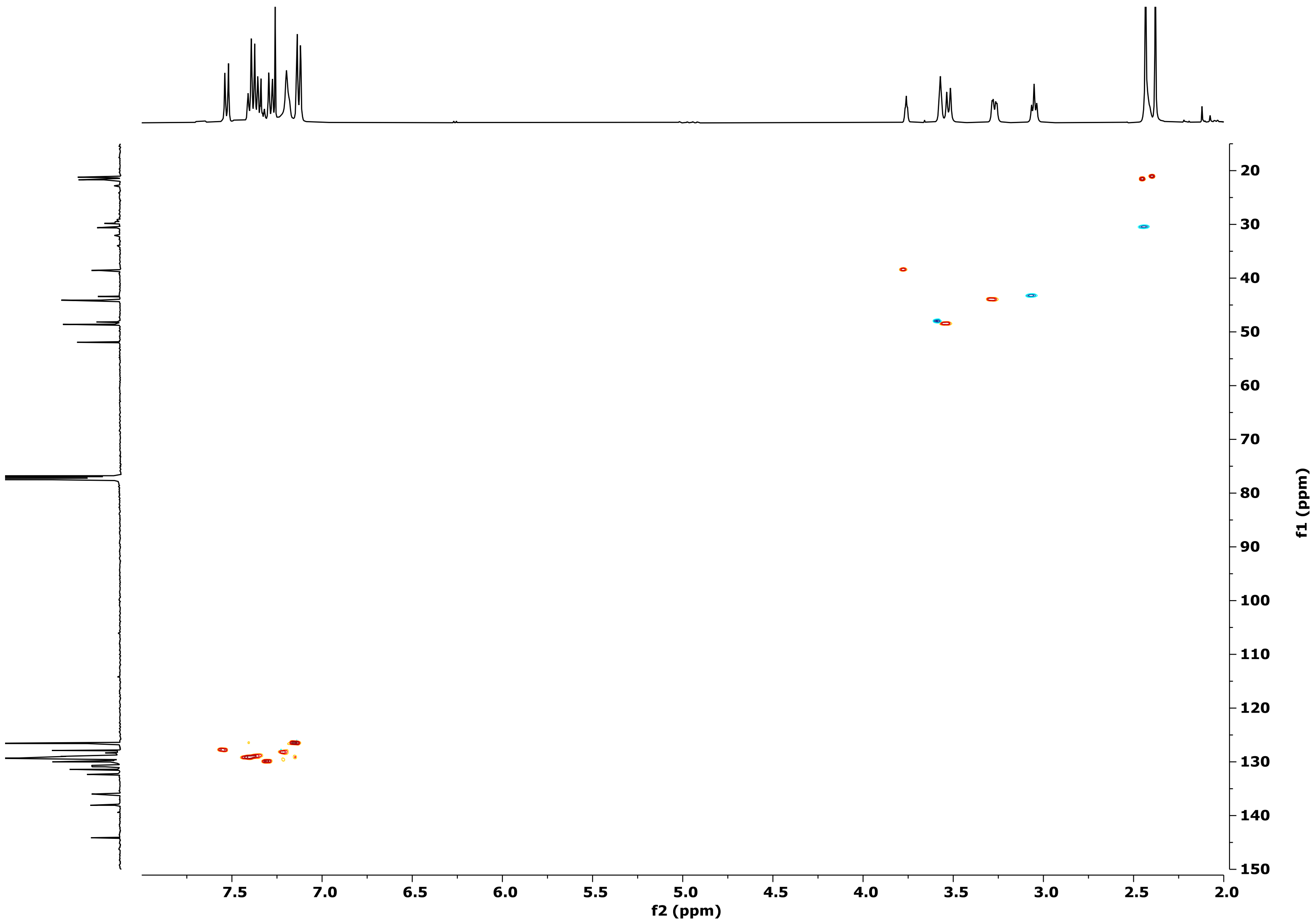

20
30
40
50
60
70
80
90
100
110
120
130
140
150
f1 (ppm)
7.5
7.0
6.5
6.0
5.5
5.0
4.5
4.0
3.5
3.0
2.5
2.0
f2 (ppm)

2D NMR COSY

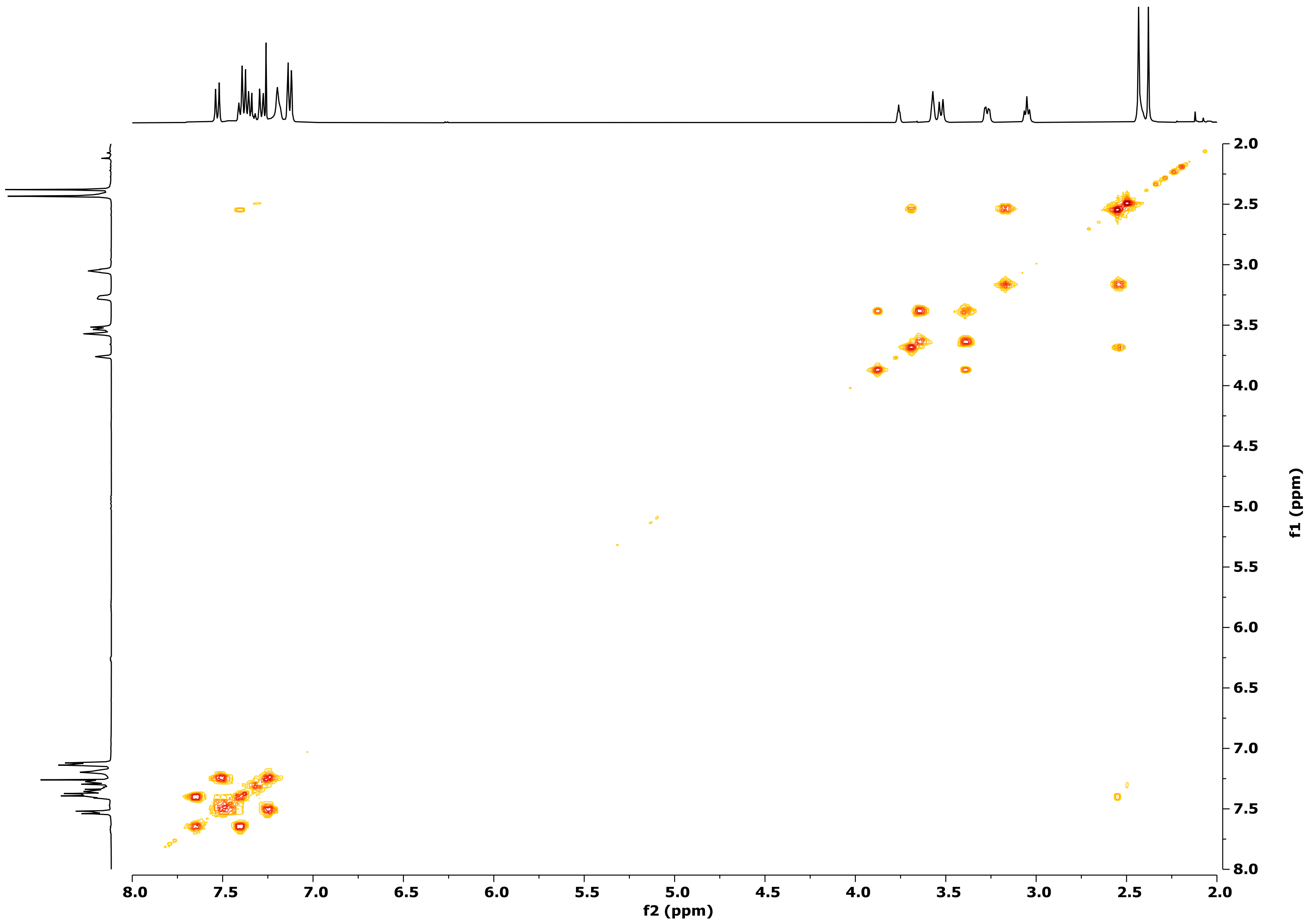

Compound 3w

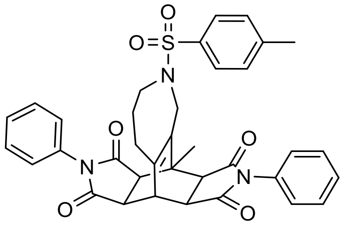

^{1}H NMR (400 MHz, $CDCl_3$)

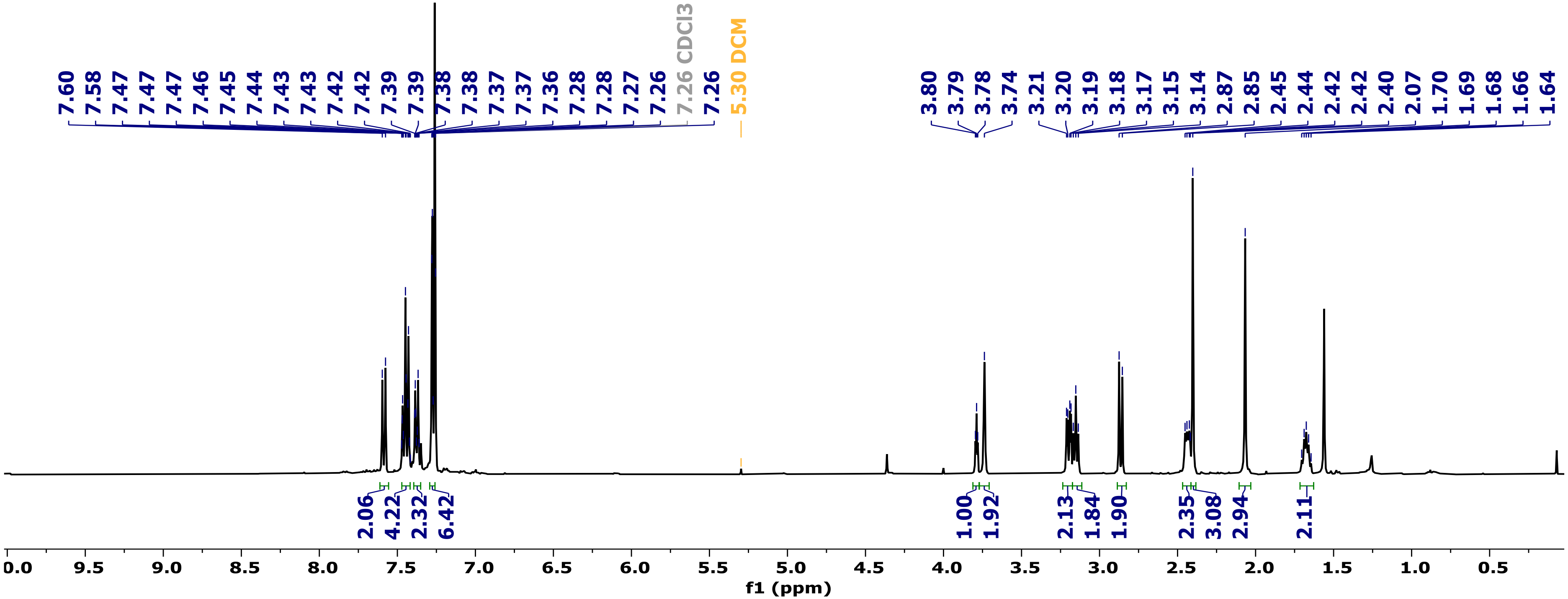

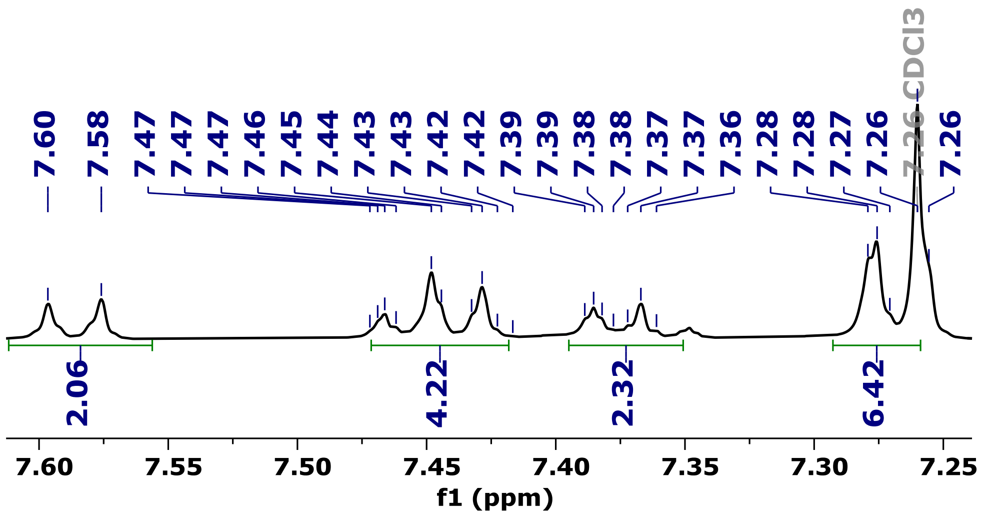
7.60
7.58
7.47
7.47
7.47
7.46
7.45
7.44
7.43
7.43
7.42
7.42
7.39
7.39
7.38
7.38
7.37
7.37
7.36
7.28
7.28
7.27
7.26
7.26 CDCl3
7.26
2.06
4.22
2.32
6.42
7.60
7.55
7.50
7.45
7.40
7.35
7.30
7.25
f1 (ppm)

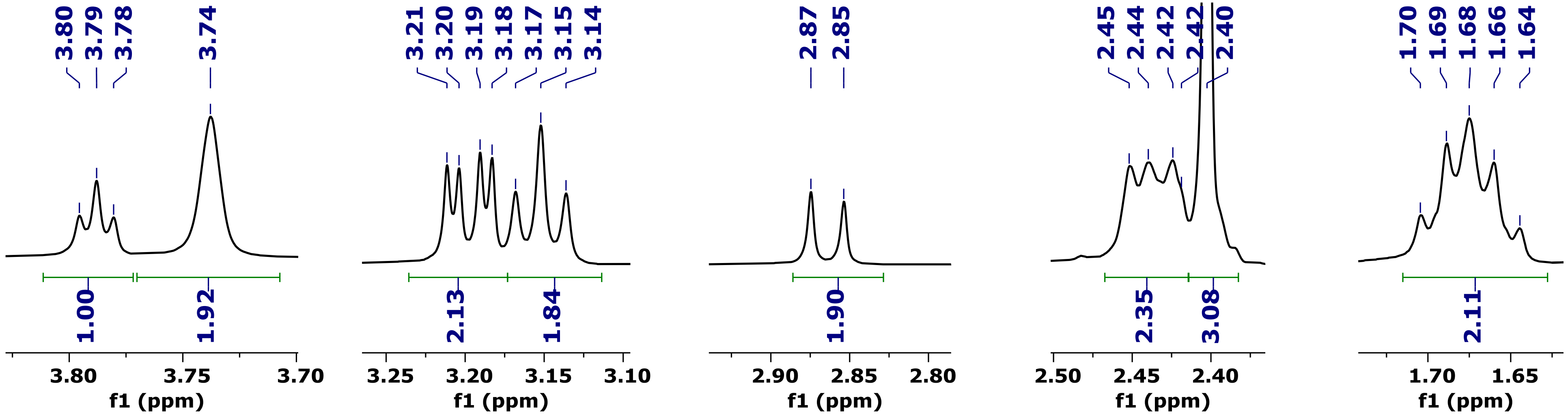
3.80
3.79
3.78
3.74
1.00
1.92
3.80
3.75
3.70
f1 (ppm)
3.21
3.20
3.19
3.18
3.17
3.15
3.14
2.13
1.84
3.25
3.20
3.15
3.10
f1 (ppm)
2.87
2.85
1.90
2.90
2.85
2.80
f1 (ppm)
2.45
2.44
2.42
2.42
2.40
2.35
3.08
2.50
2.45
2.40
f1 (ppm)
1.70
1.69
1.68
1.66
1.64
2.11
1.70
1.65
f1 (ppm)

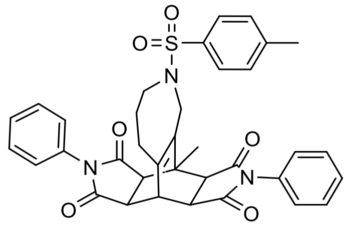

^{13}C NMR (101 MHz, CDCl$_3$)

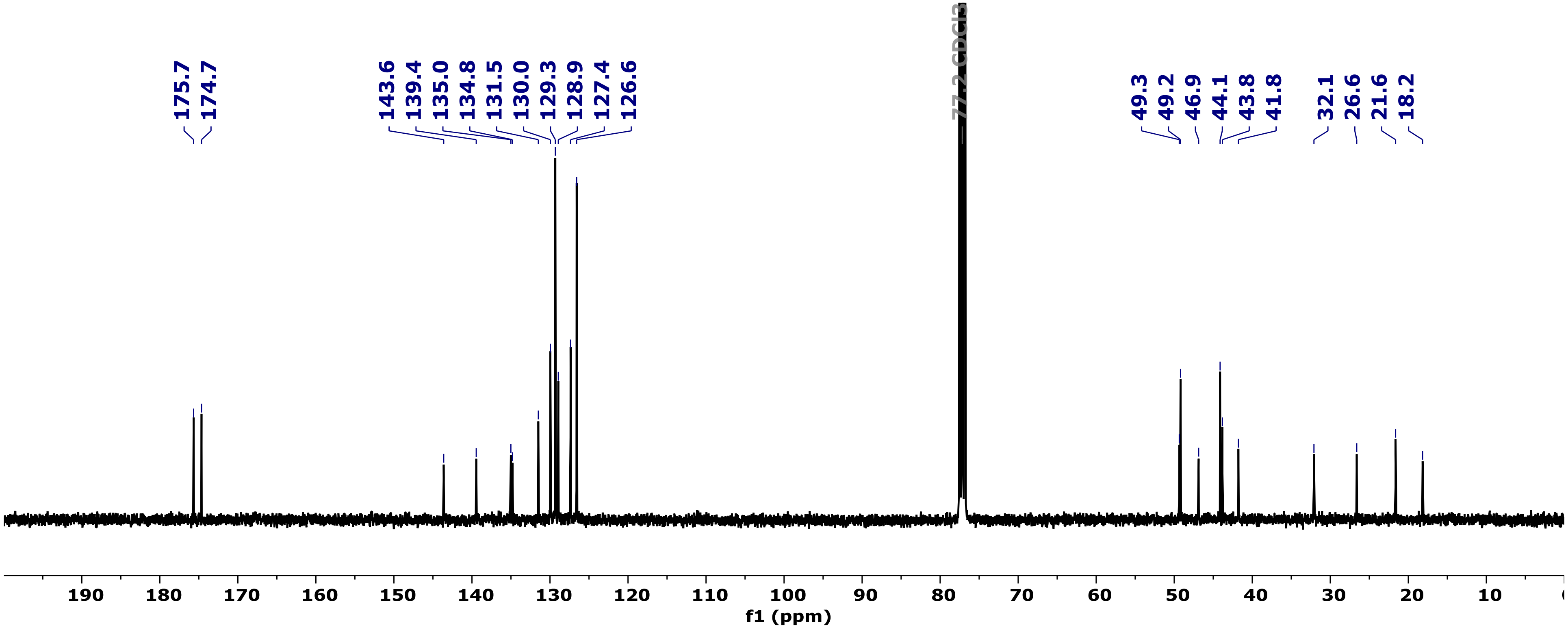

2D NMR HSQC

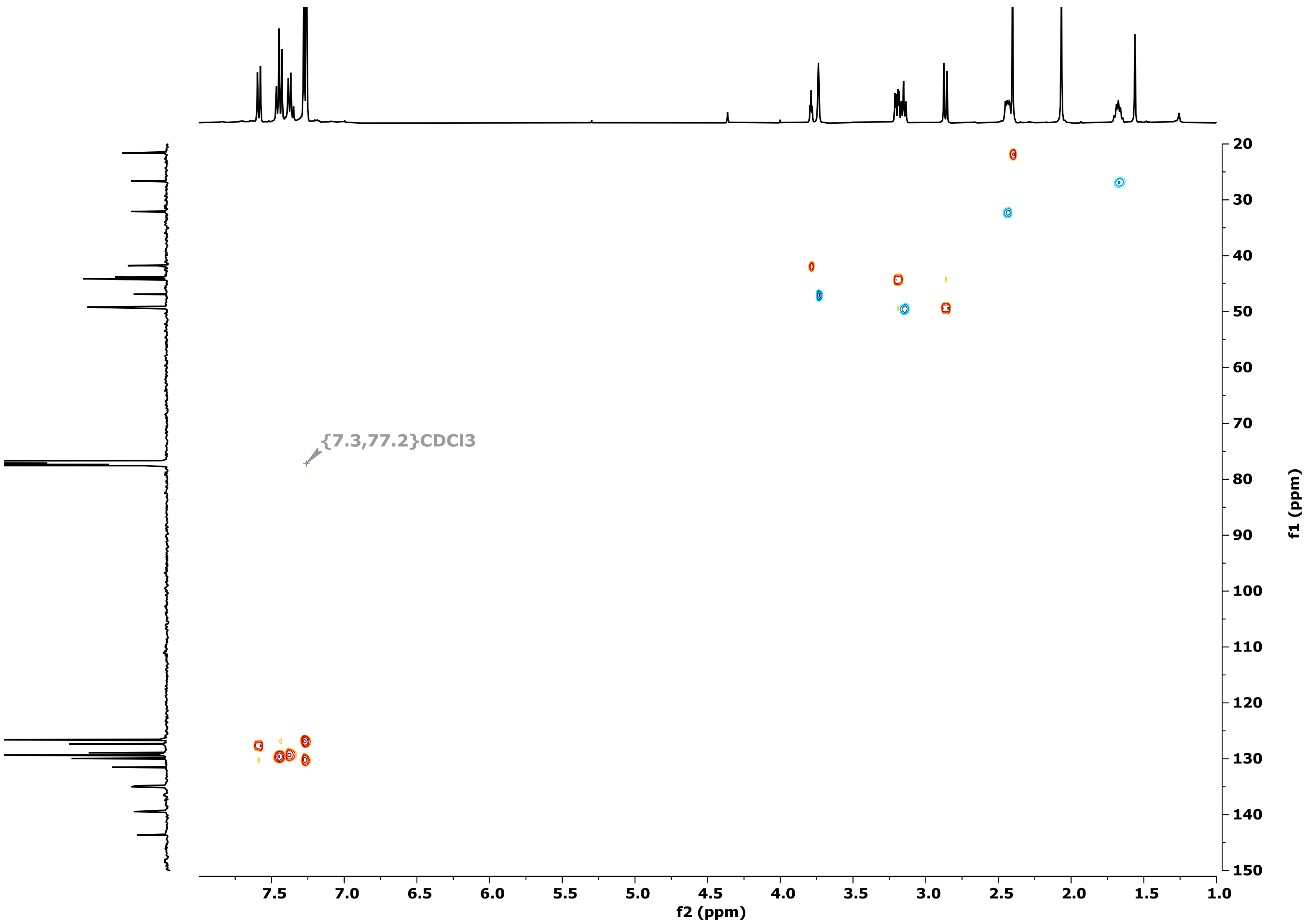

{7.3,77.2}CDCl3
f1 (ppm)
f2 (ppm)
20
30
40
50
60
70
80
90
100
110
120
130
140
150
7.5
7.0
6.5
6.0
5.5
5.0
4.5
4.0
3.5
3.0
2.5
2.0
1.5
1.0

2D NMR COSY

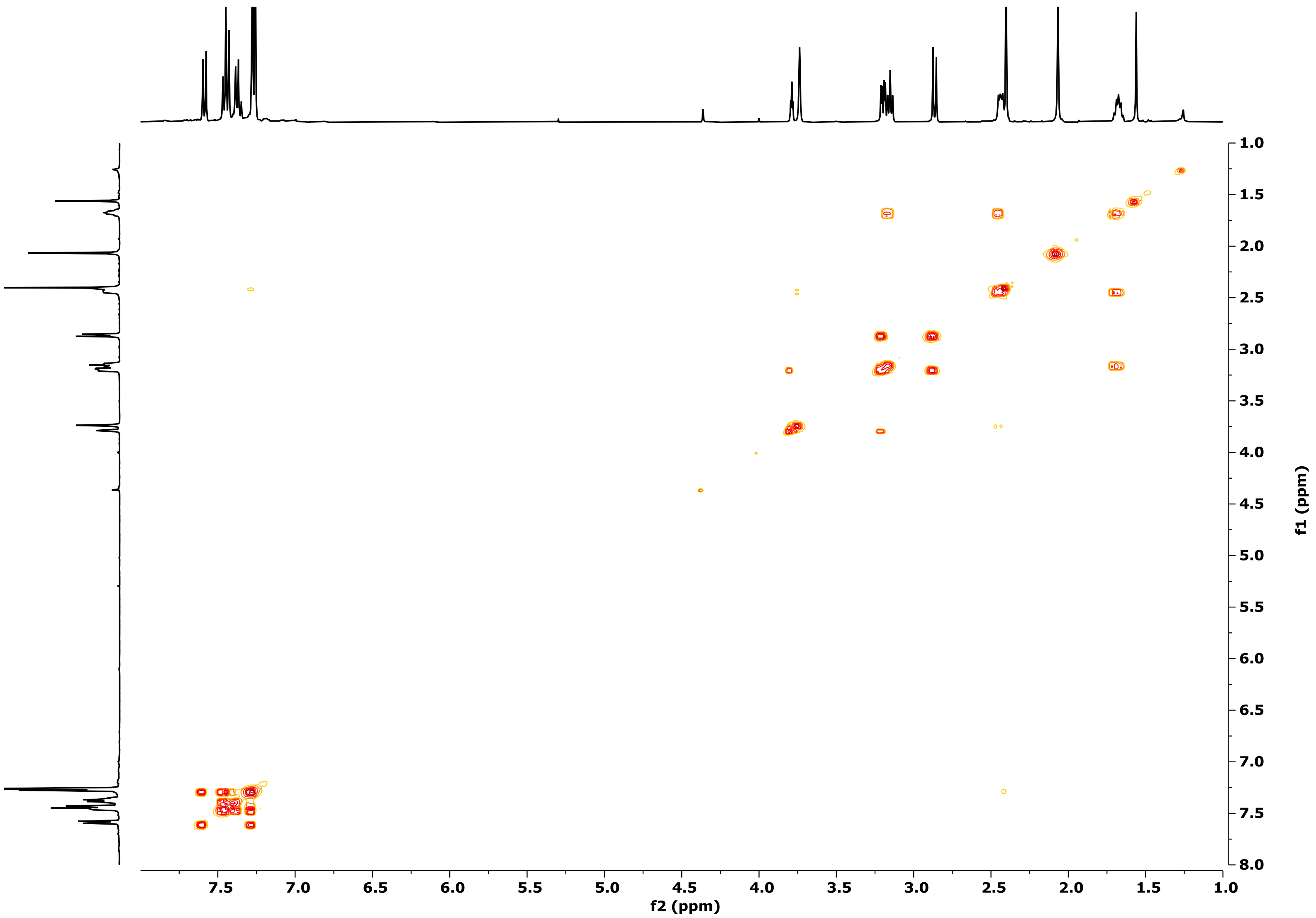

1.0
1.5
2.0
2.5
3.0
3.5
4.0
4.5
5.0
5.5
6.0
6.5
7.0
7.5
8.0
f1 (ppm)
7.5
7.0
6.5
6.0
5.5
5.0
4.5
4.0
3.5
3.0
2.5
2.0
1.5
1.0
f2 (ppm)

Compound 3x

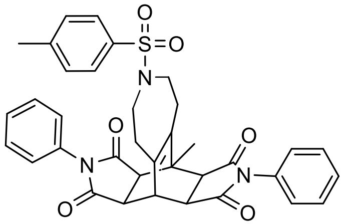

^{1}H NMR (400 MHz, $CDCl_3$)

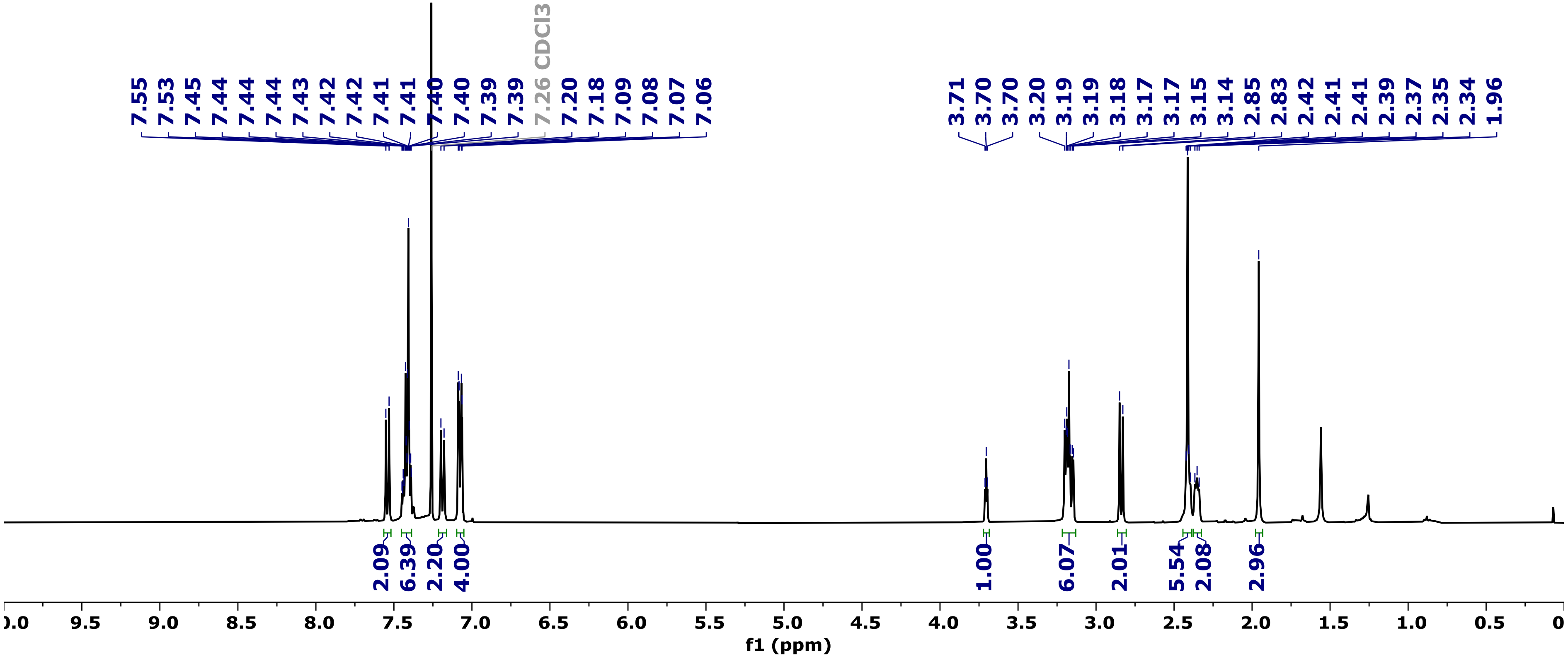

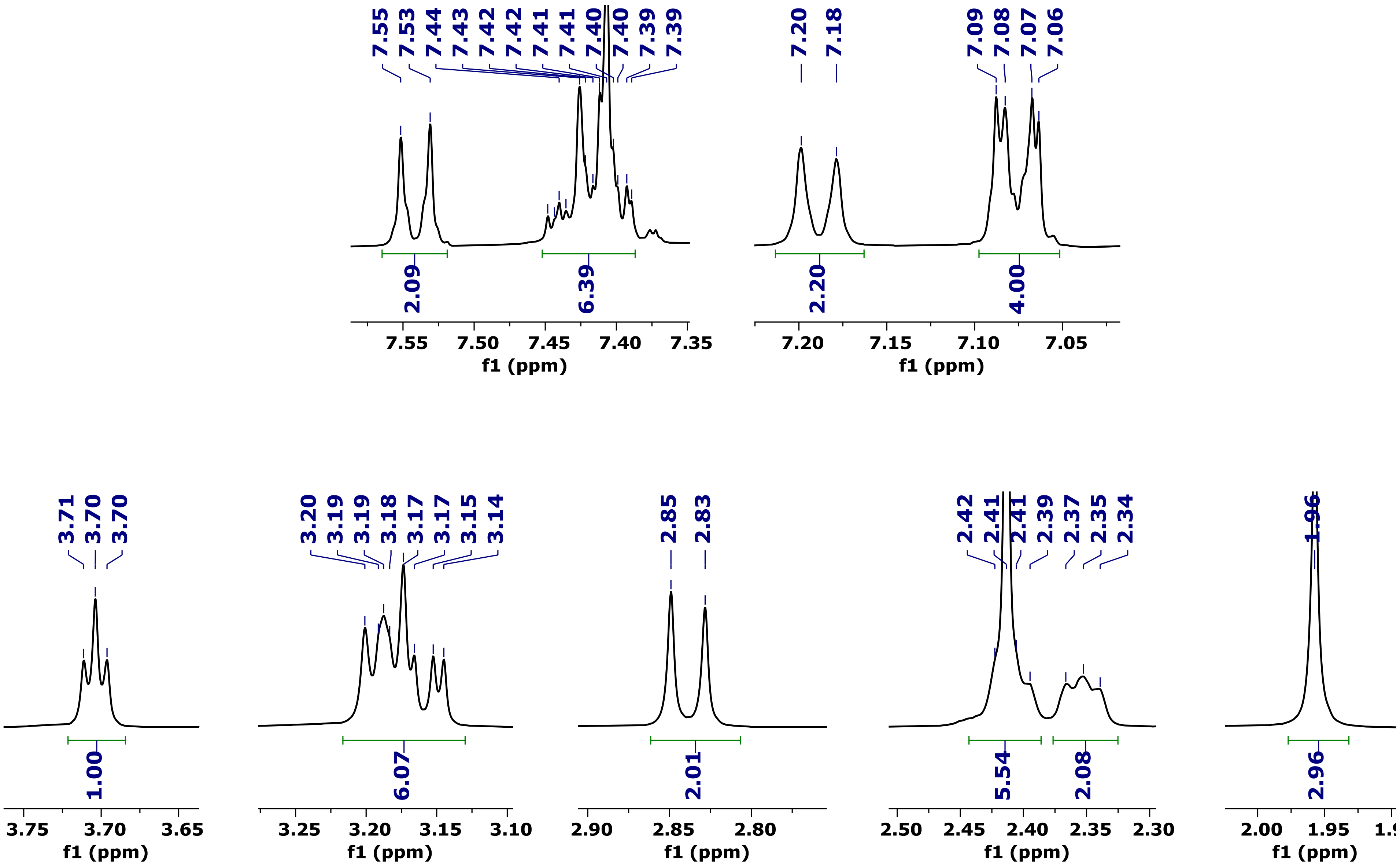
7.55 7.53 7.44 7.43 7.42 7.42 7.41 7.41 7.40 7.40 7.39 7.39
2.09
6.39
7.55 7.50 7.45 7.40 7.35
f1 (ppm)
7.20 7.18
7.09 7.08 7.07 7.06
2.20
4.00
7.20 7.15 7.10 7.05
f1 (ppm)
3.71 3.70 3.70
1.00
3.75 3.70 3.65
f1 (ppm)
3.20 3.19 3.19 3.18 3.17 3.17 3.15 3.14
6.07
3.25 3.20 3.15 3.10
f1 (ppm)
2.85 2.83
2.01
2.90 2.85 2.80
f1 (ppm)
2.42 2.41 2.41 2.39 2.37 2.35 2.34
5.54
2.08
2.50 2.45 2.40 2.35 2.30
f1 (ppm)
1.96
2.96
2.00 1.95
f1 (ppm)

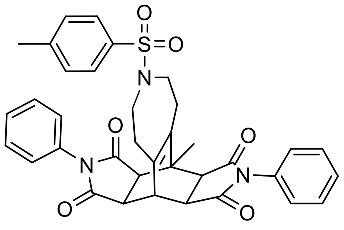

^{13}C NMR (101 MHz, $CDCl_3$)

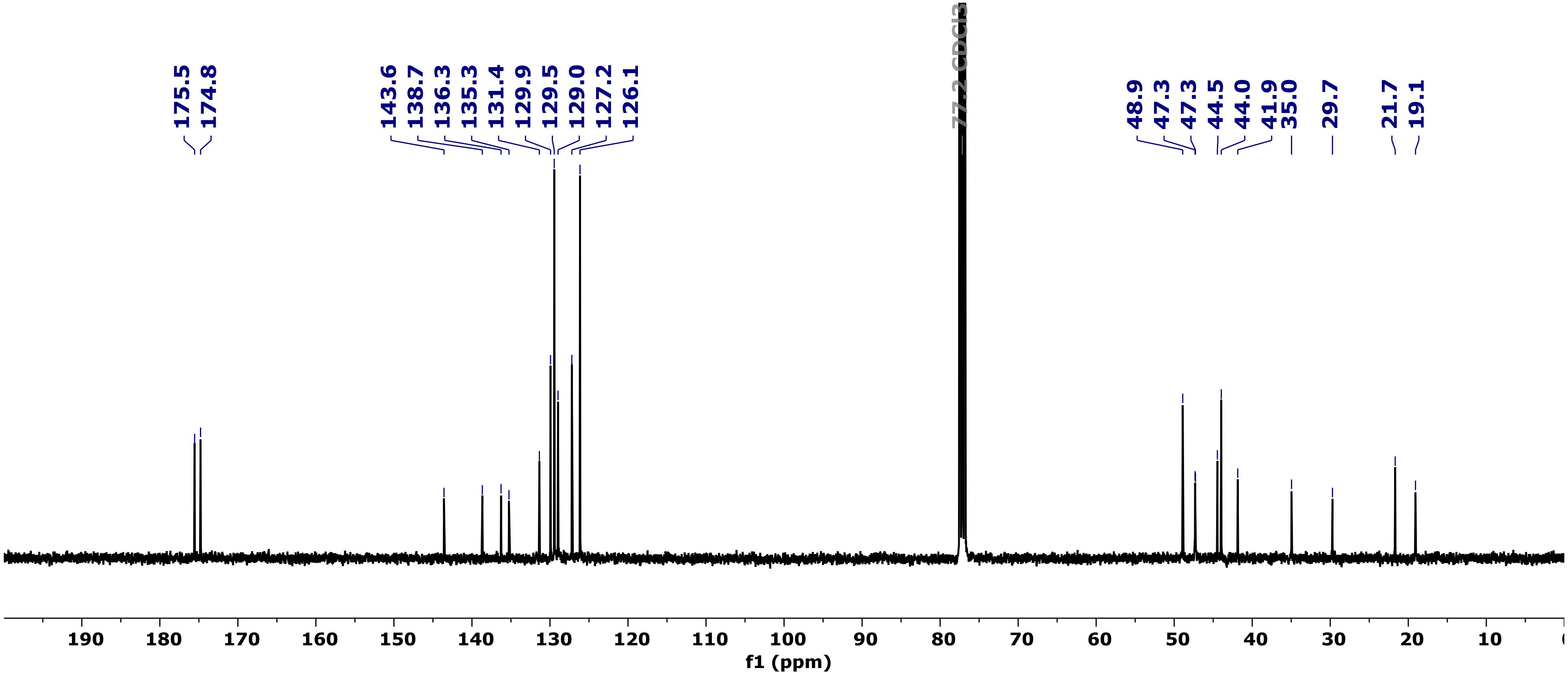

2D NMR HSQC

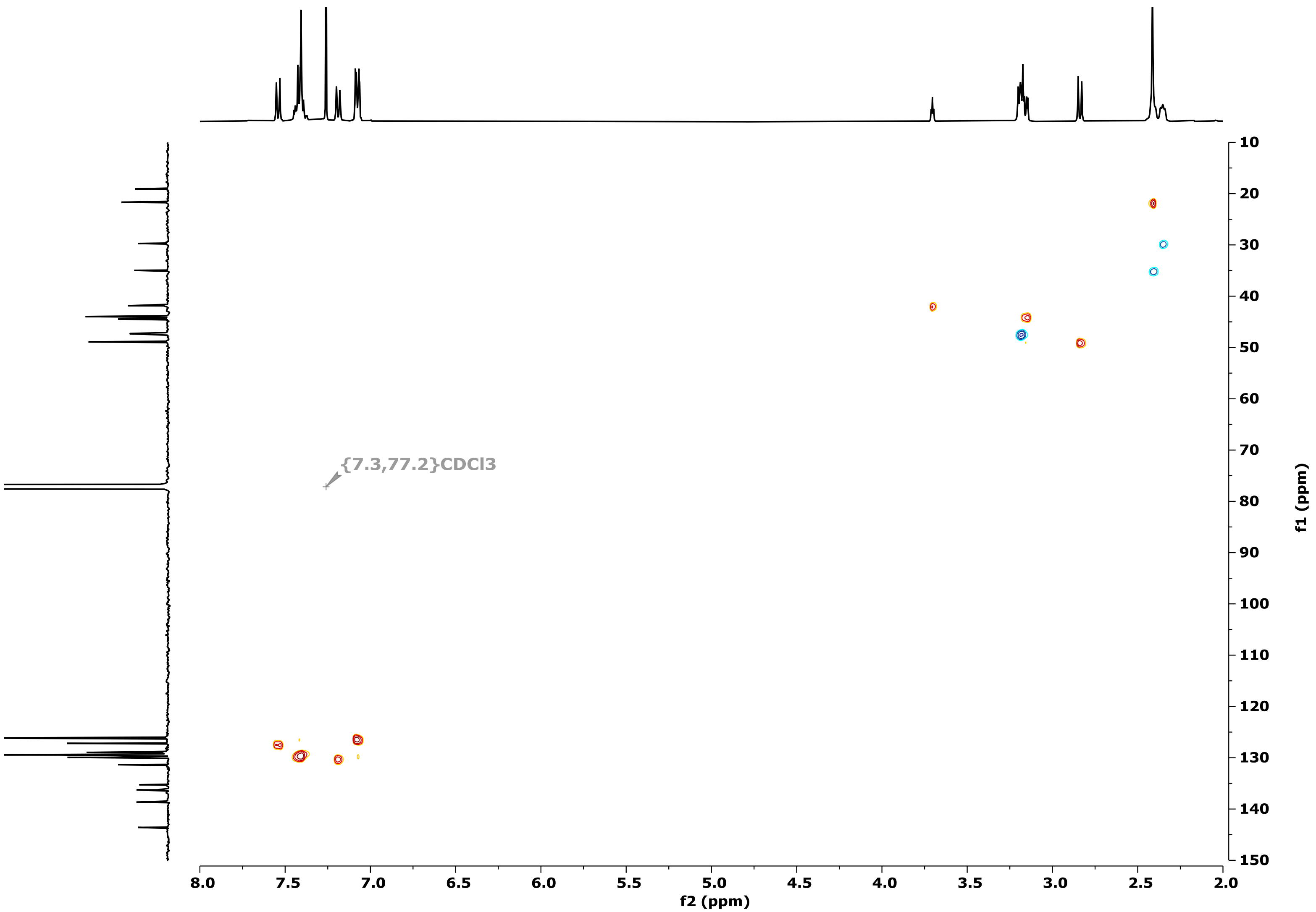
{7.3,77.2}CDCl3
f2 (ppm)
f1 (ppm)
8.0
7.5
7.0
6.5
6.0
5.5
5.0
4.5
4.0
3.5
3.0
2.5
2.0
10
20
30
40
50
60
70
80
90
100
110
120
130
140
150

2D NMR COSY

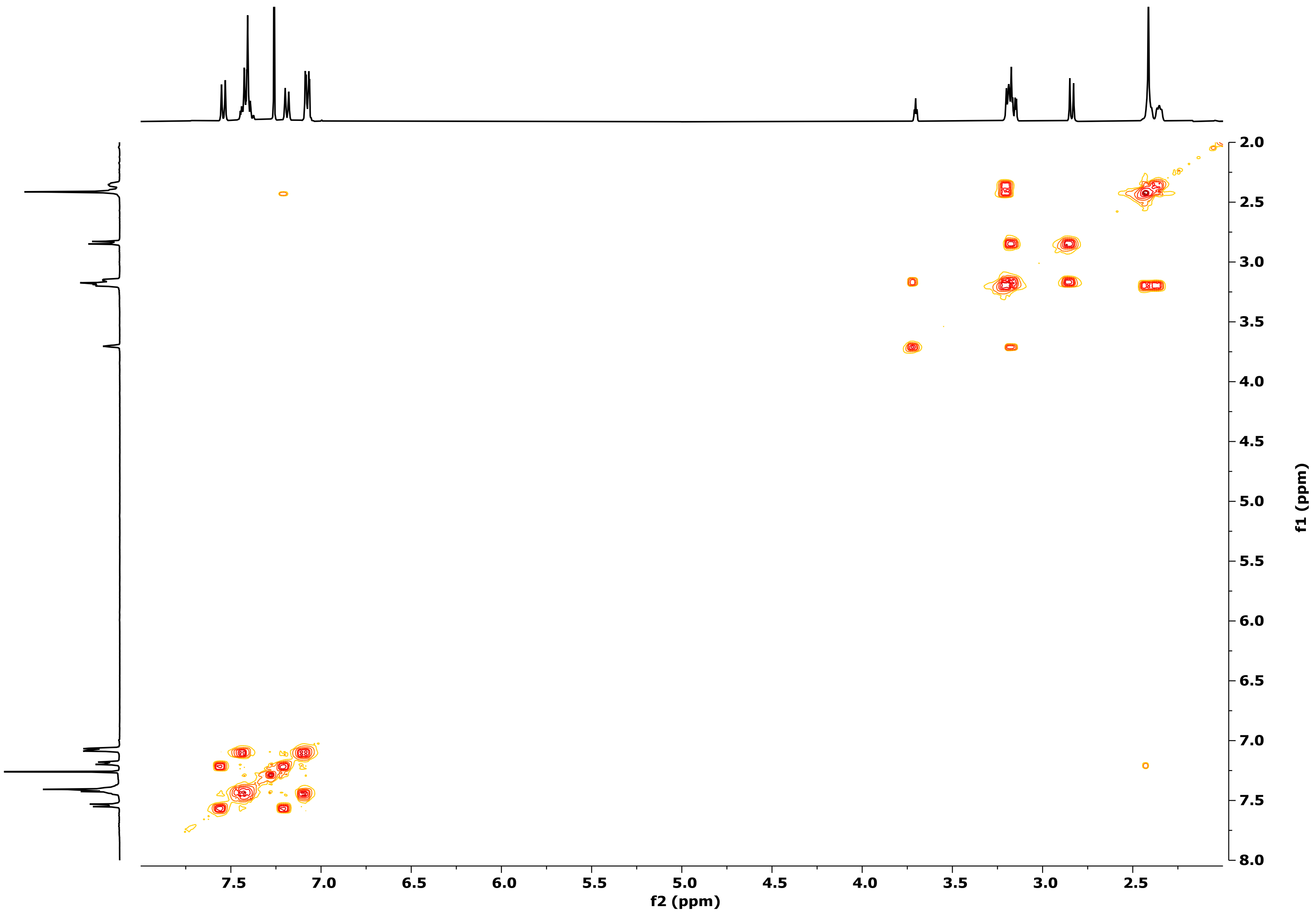

2.0
2.5
3.0
3.5
4.0
4.5
5.0
5.5
6.0
6.5
7.0
7.5
8.0
f1 (ppm)
7.5
7.0
6.5
6.0
5.5
5.0
4.5
4.0
3.5
3.0
2.5
f2 (ppm)

Compound 5

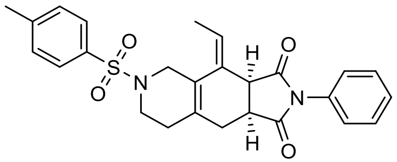

^{1}H NMR (400 MHz, $CDCl_3$)

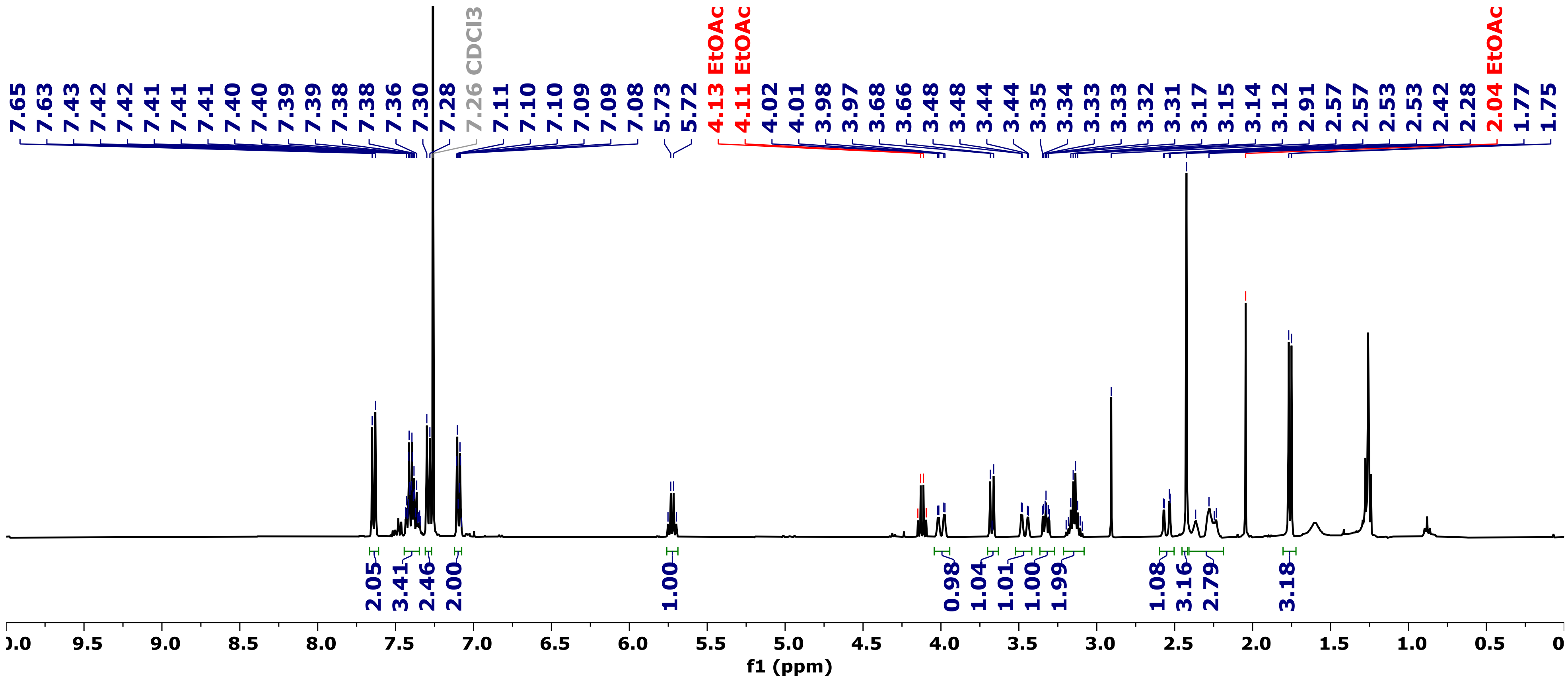

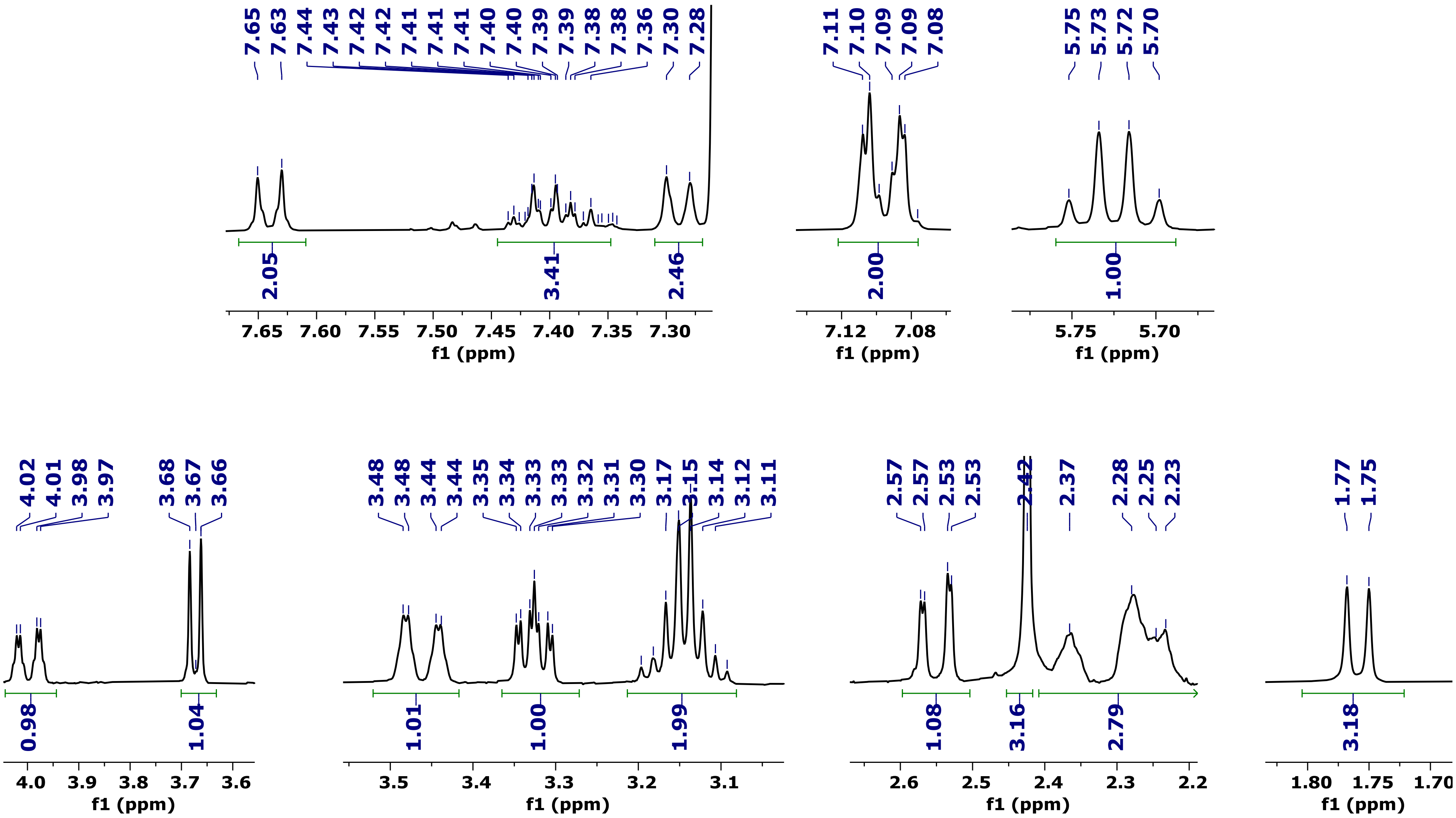
7.65 7.63 7.44 7.43 7.42 7.42 7.41 7.41 7.41 7.40 7.40 7.39 7.39 7.38 7.38 7.36 7.30 7.28
2.05 3.41 2.46
f1 (ppm)
7.11 7.10 7.09 7.09 7.08
2.00
f1 (ppm)
5.75 5.73 5.72 5.70
1.00
f1 (ppm)
4.02 4.01 3.98 3.97 3.68 3.67 3.66
0.98 1.04
f1 (ppm)
3.48 3.48 3.44 3.44 3.35 3.34 3.33 3.33 3.32 3.31 3.30 3.17 3.15 3.14 3.12 3.11
1.01 1.00 1.99
f1 (ppm)
2.57 2.57 2.53 2.53 2.42 2.37 2.28 2.25 2.23
1.08 3.16 2.79
f1 (ppm)
1.77 1.75
3.18
f1 (ppm)

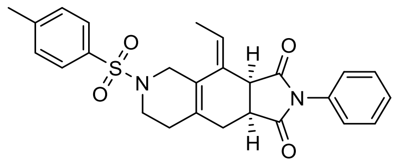

^{13}C NMR (101 MHz, $CDCl_3$)

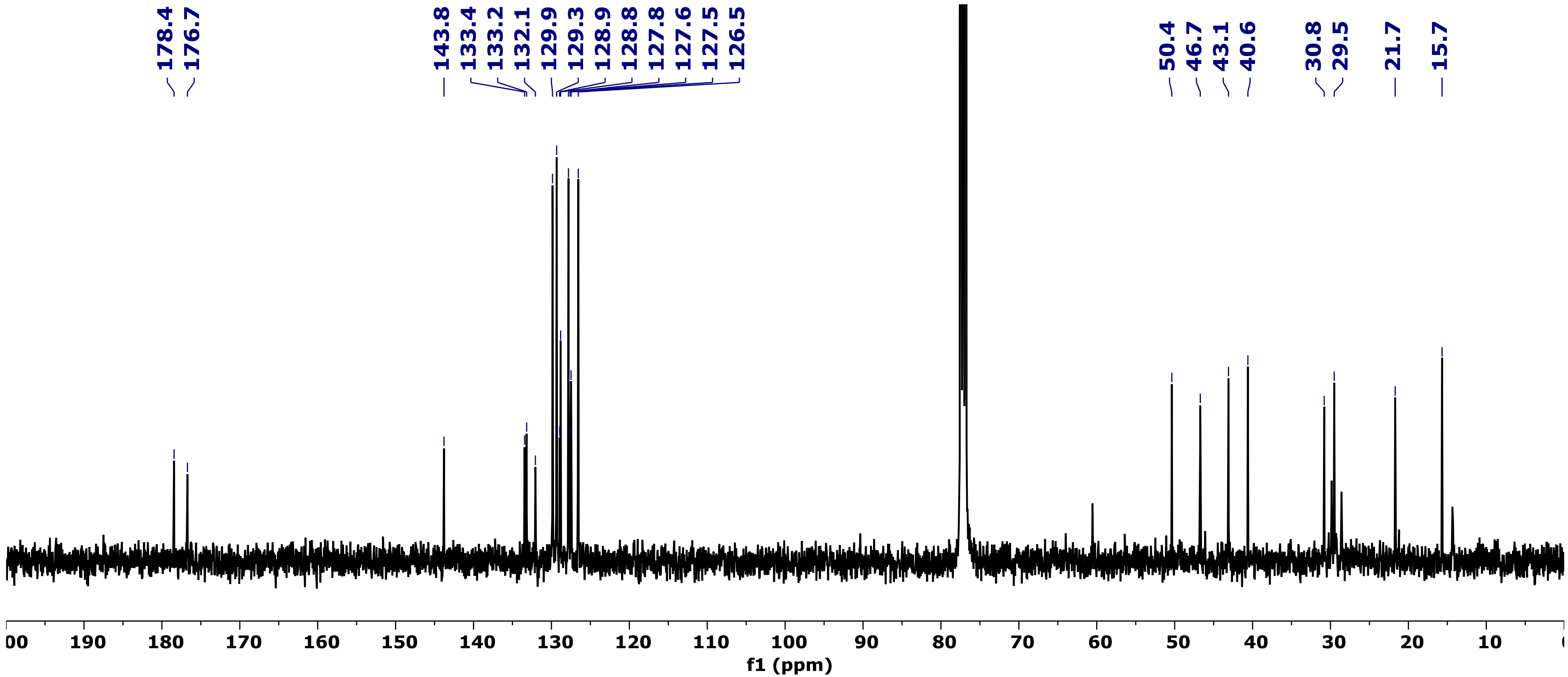

2D NMR HSQC

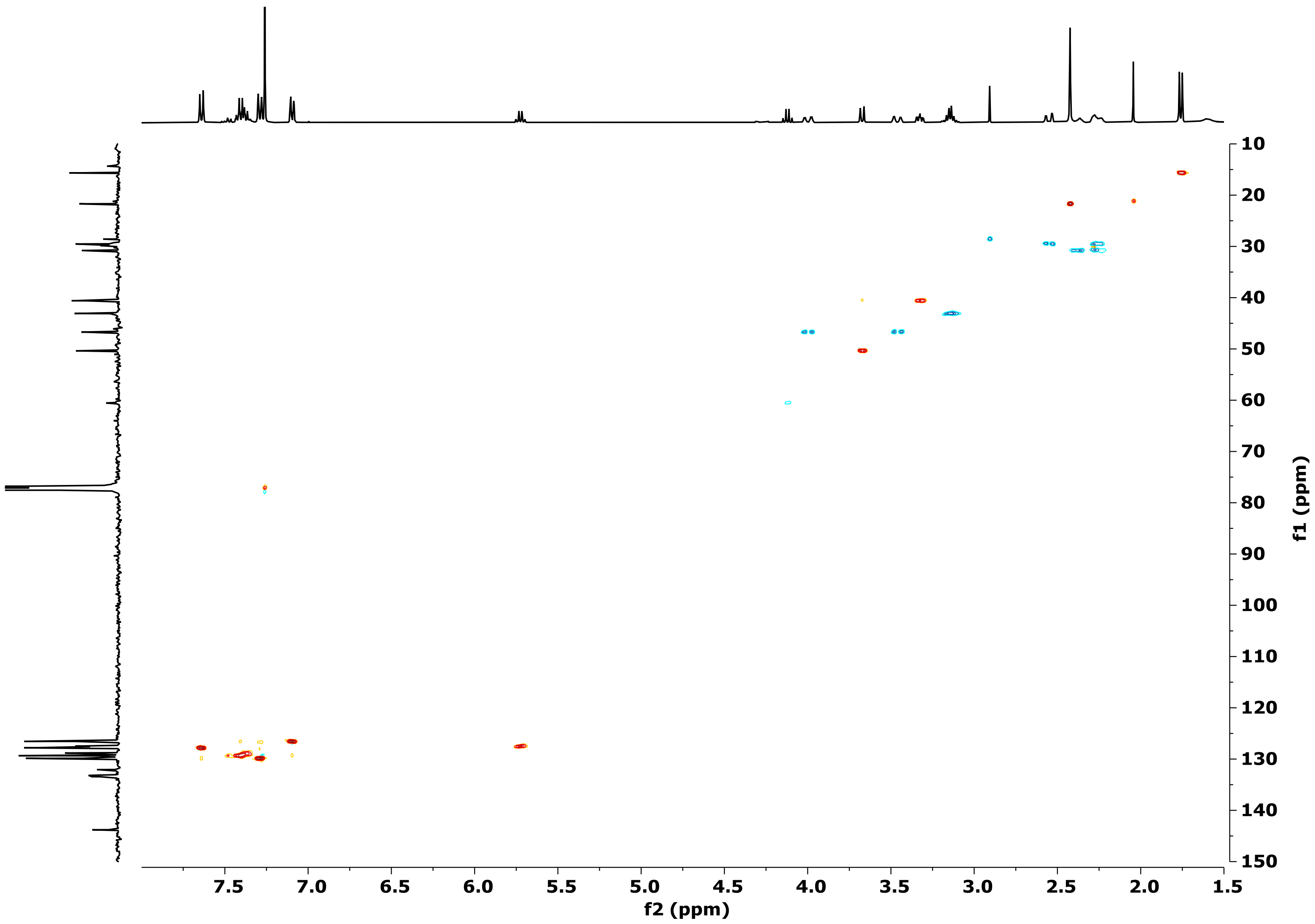

f2 (ppm)
f1 (ppm)
7.5
7.0
6.5
6.0
5.5
5.0
4.5
4.0
3.5
3.0
2.5
2.0
1.5
10
20
30
40
50
60
70
80
90
100
110
120
130
140
150

2D NMR COSY

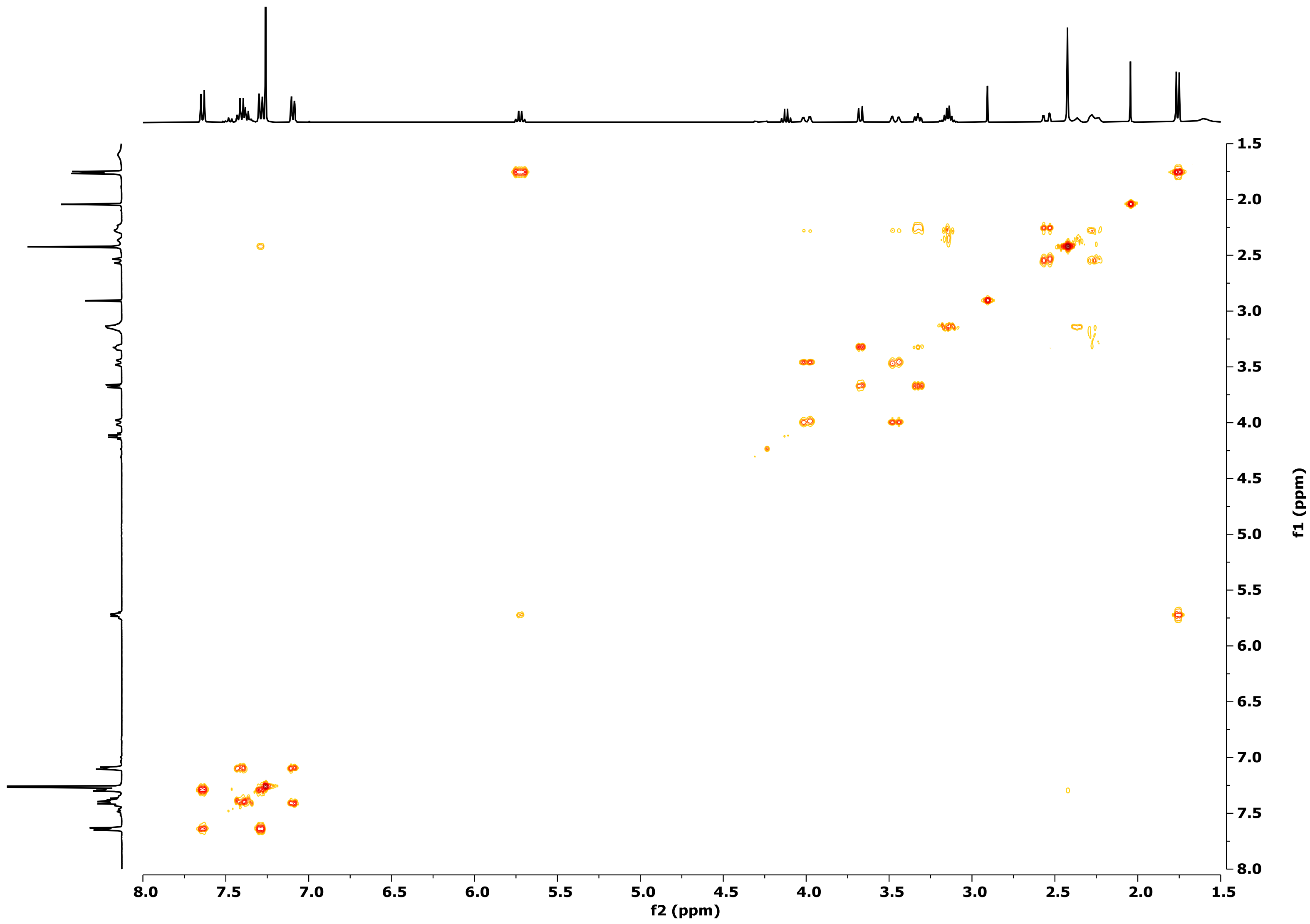

Compound 3ab

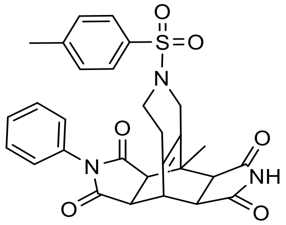

^{1}H NMR (400 MHz, DMSO)

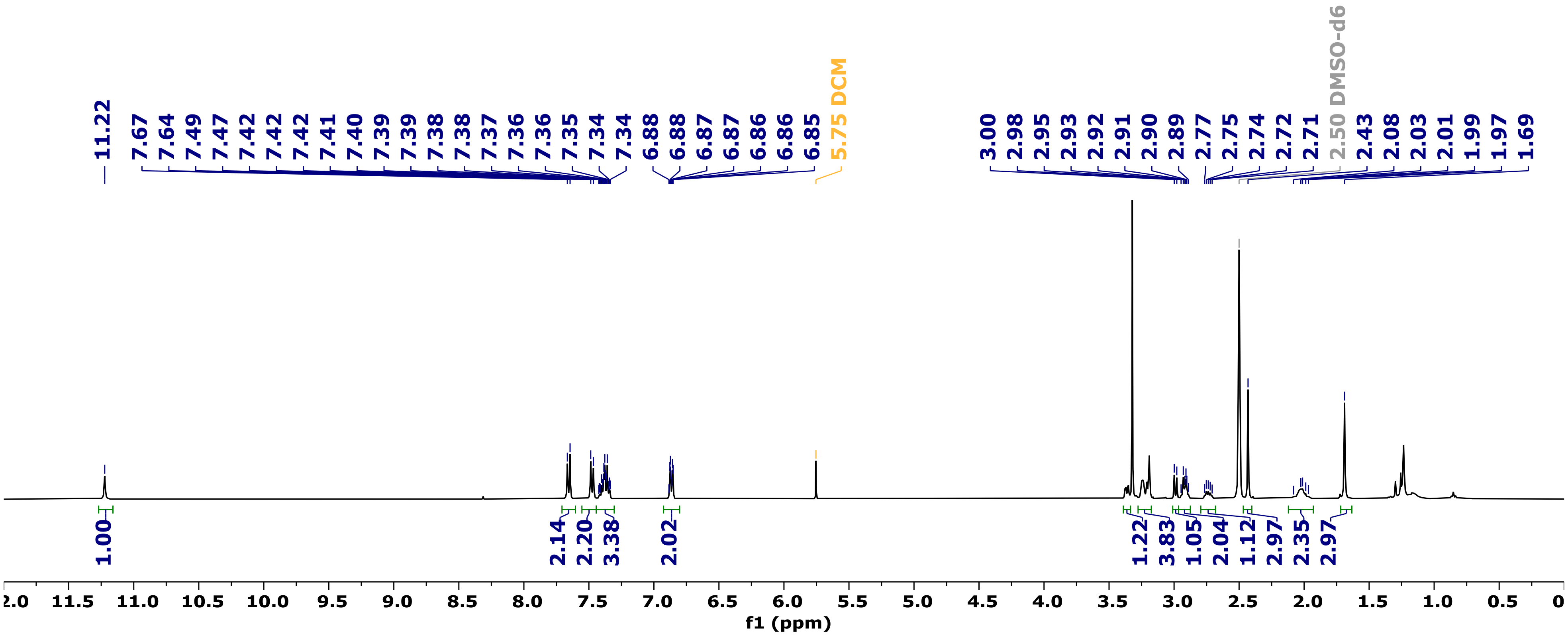

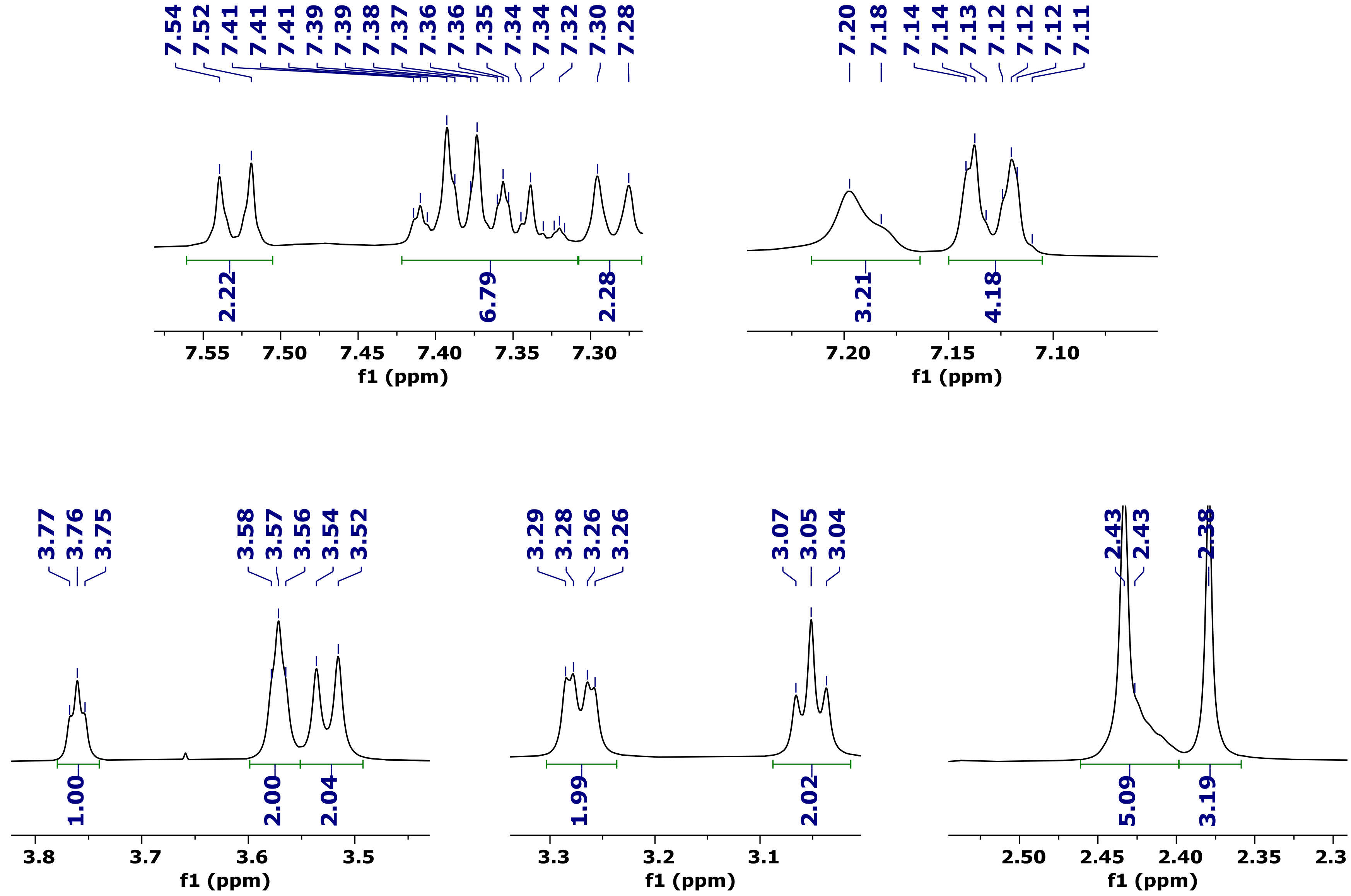
7.54 7.52 7.41 7.41 7.41 7.39 7.39 7.38 7.37 7.36 7.36 7.35 7.34 7.34 7.32 7.30 7.28
2.22
6.79
2.28
7.55 7.50 7.45 7.40 7.35 7.30
f1 (ppm)
7.20 7.18 7.14 7.14 7.13 7.12 7.12 7.12 7.11
3.21
4.18
7.20 7.15 7.10
f1 (ppm)
3.77 3.76 3.75
3.58 3.57 3.56 3.54 3.52
1.00
2.00
2.04
3.8 3.7 3.6 3.5
f1 (ppm)
3.29 3.28 3.26 3.26
3.07 3.05 3.04
1.99
2.02
3.3 3.2 3.1
f1 (ppm)
2.43 2.43
2.38
5.09
3.19
2.50 2.45 2.40 2.35 2.3
f1 (ppm)

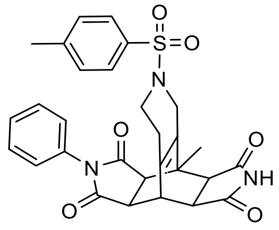

^{13}C NMR (101 MHz, DMSO)

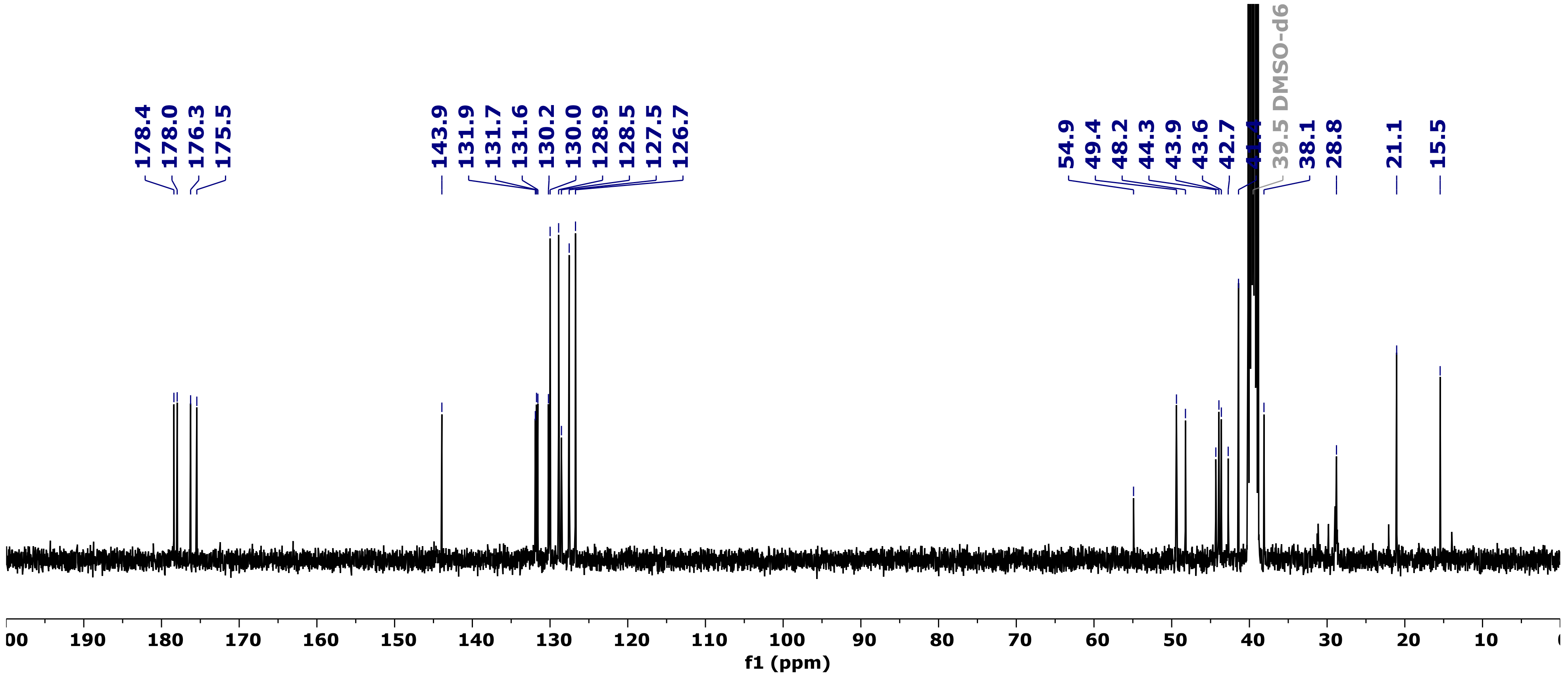

2D NMR HSQC

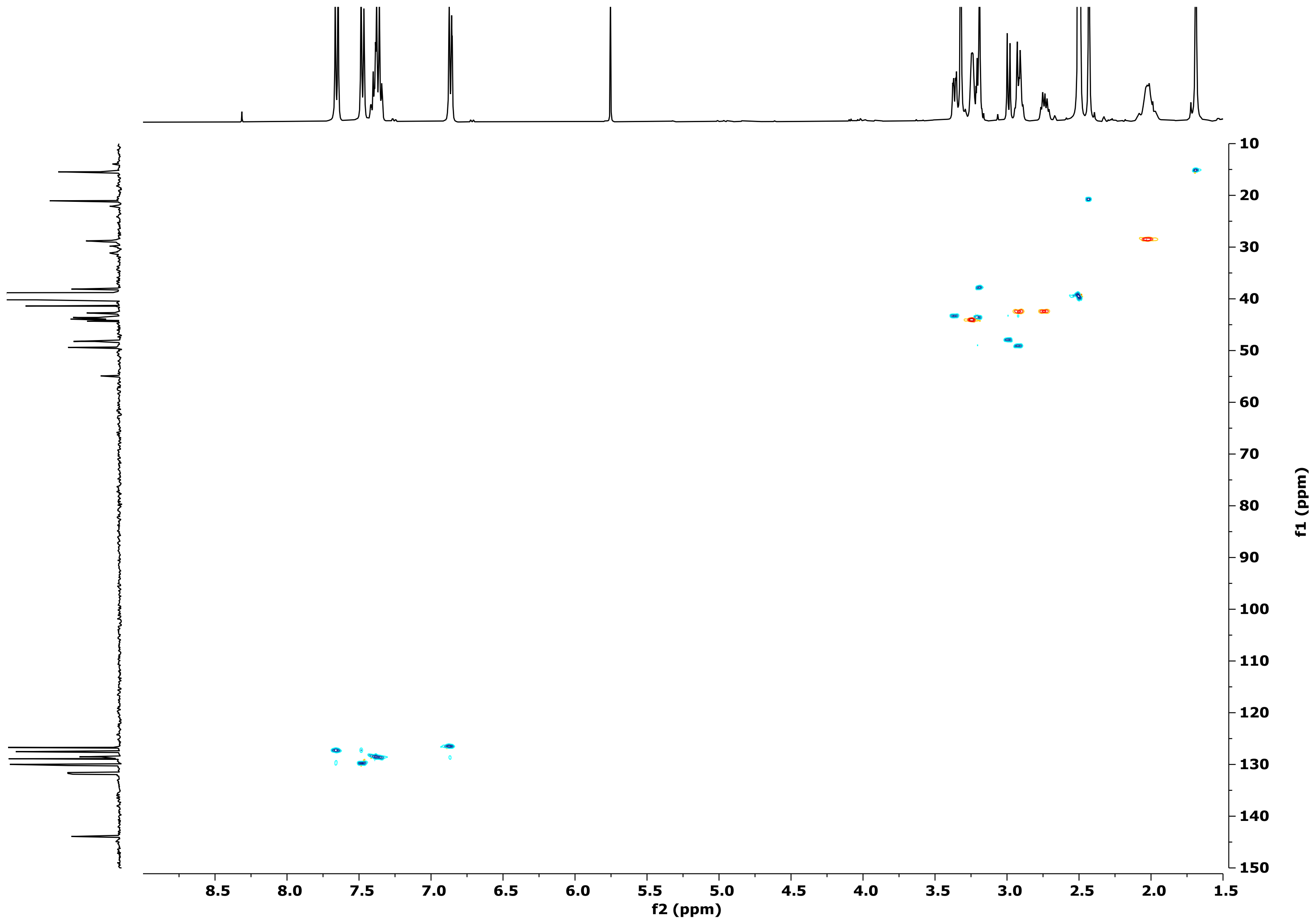

8.5
8.0
7.5
7.0
6.5
6.0
5.5
5.0
4.5
4.0
3.5
3.0
2.5
2.0
1.5
f2 (ppm)
10
20
30
40
50
60
70
80
90
100
110
120
130
140
150
f1 (ppm)

2D NMR COSY

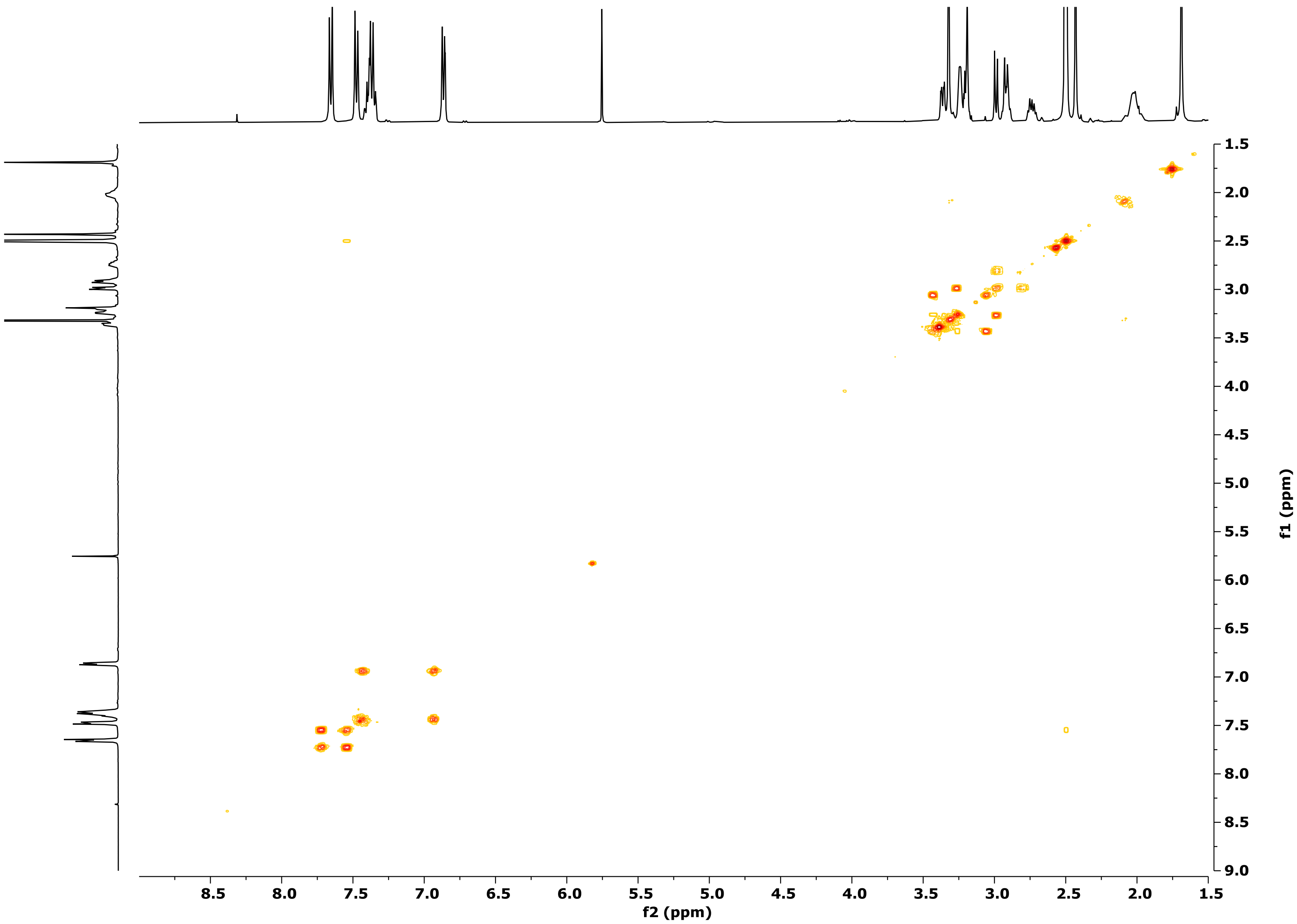

Compound 3ae

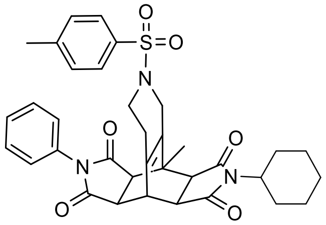

^{1}H NMR (400 MHz, $CDCl_3$)

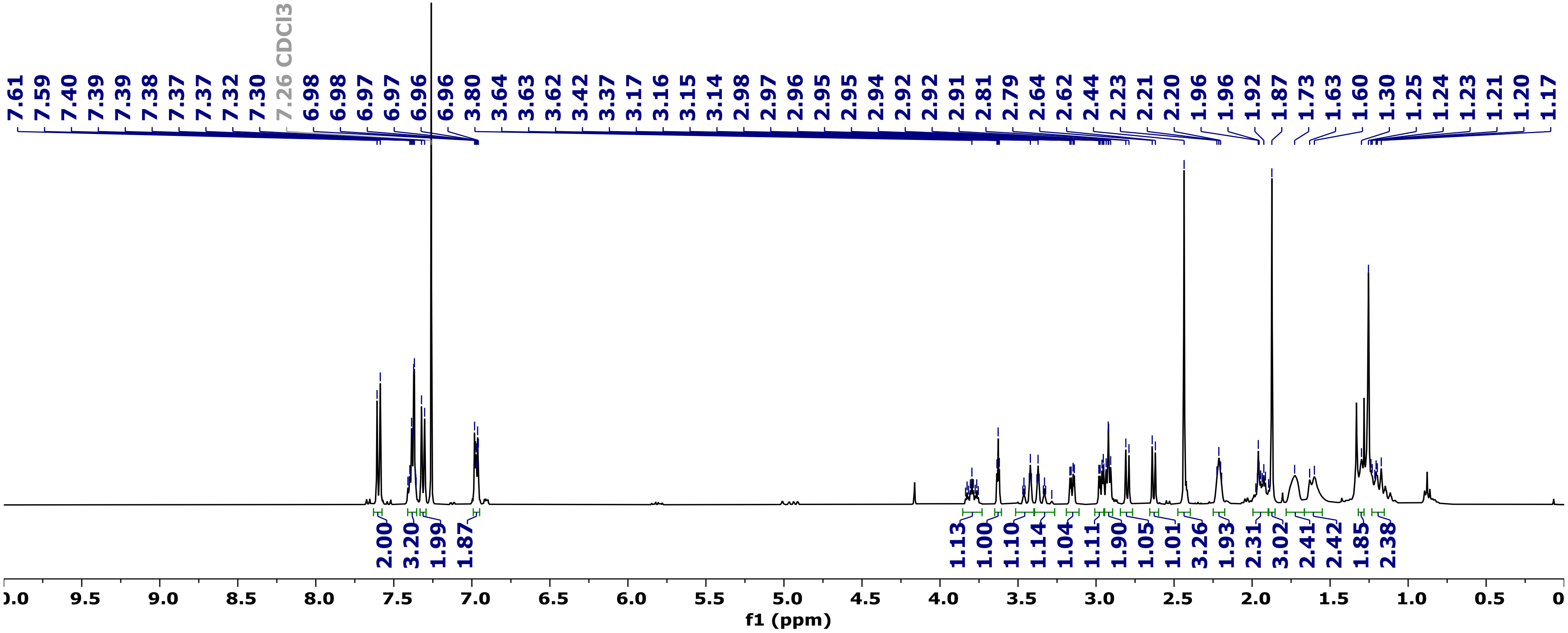

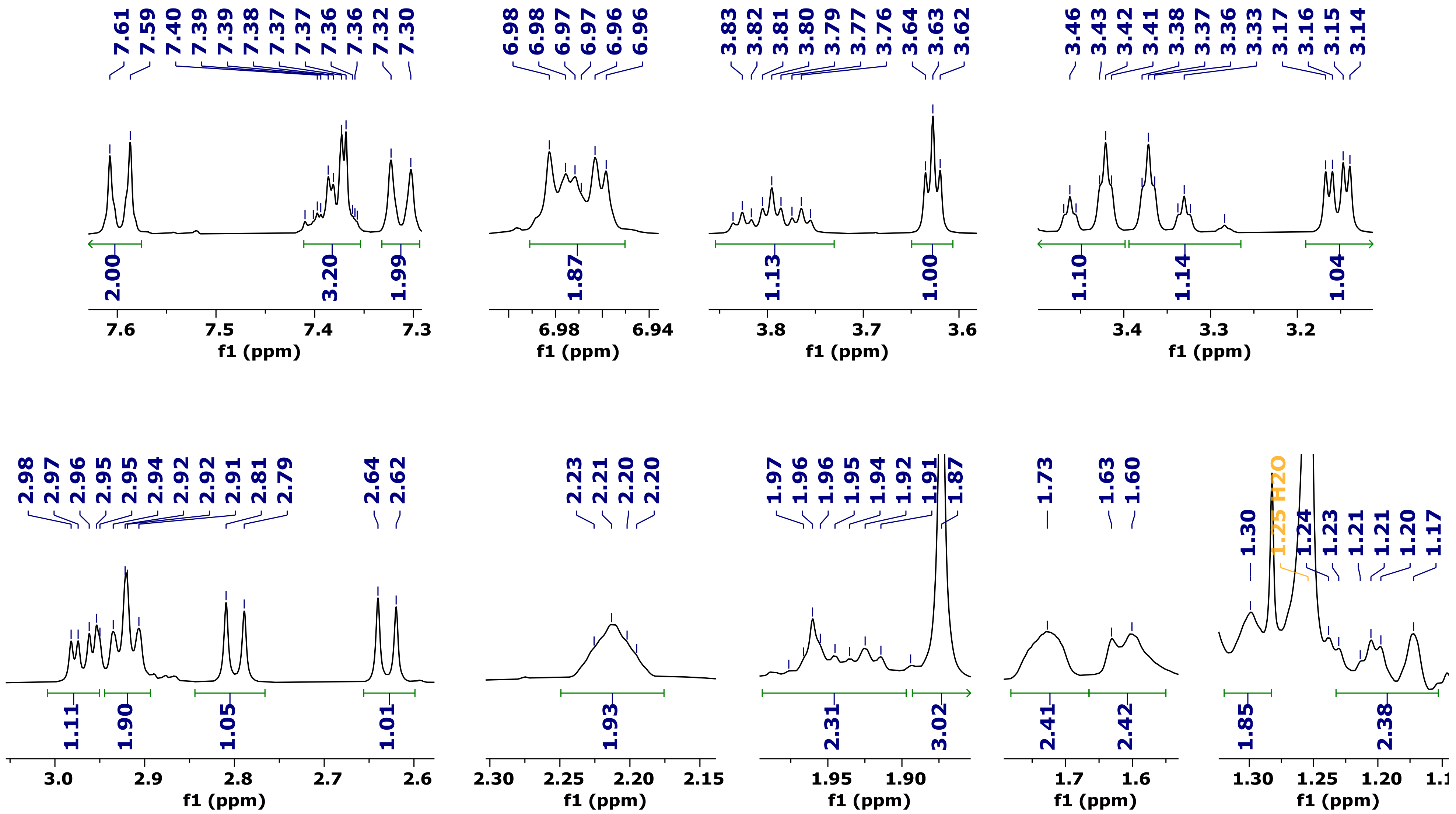
7.61 7.59 7.40 7.39 7.39 7.38 7.37 7.37 7.36 7.36 7.32 7.30
2.00 3.20 1.99
f1 (ppm)
6.98 6.98 6.97 6.97 6.96 6.96
1.87
f1 (ppm)
3.83 3.82 3.81 3.80 3.79 3.77 3.76 3.64 3.63 3.62
1.13 1.00
f1 (ppm)
3.46 3.43 3.42 3.41 3.38 3.37 3.36 3.33 3.17 3.16 3.15 3.14
1.10 1.14 1.04
f1 (ppm)
2.98 2.97 2.96 2.95 2.95 2.94 2.92 2.92 2.91 2.81 2.79 2.64 2.62
1.11 1.90 1.05 1.01
f1 (ppm)
2.23 2.21 2.20 2.20
1.93
f1 (ppm)
1.97 1.96 1.96 1.95 1.94 1.92 1.91 1.87
2.31 3.02
f1 (ppm)
1.73 1.63 1.60
2.41 2.42
f1 (ppm)
1.30 1.25 H2O 1.24 1.23 1.21 1.21 1.20 1.17
1.85 2.38
f1 (ppm)

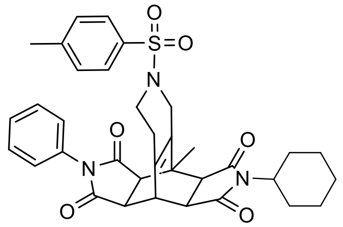

^{13}C NMR (101 MHz, $CDCl_3$)

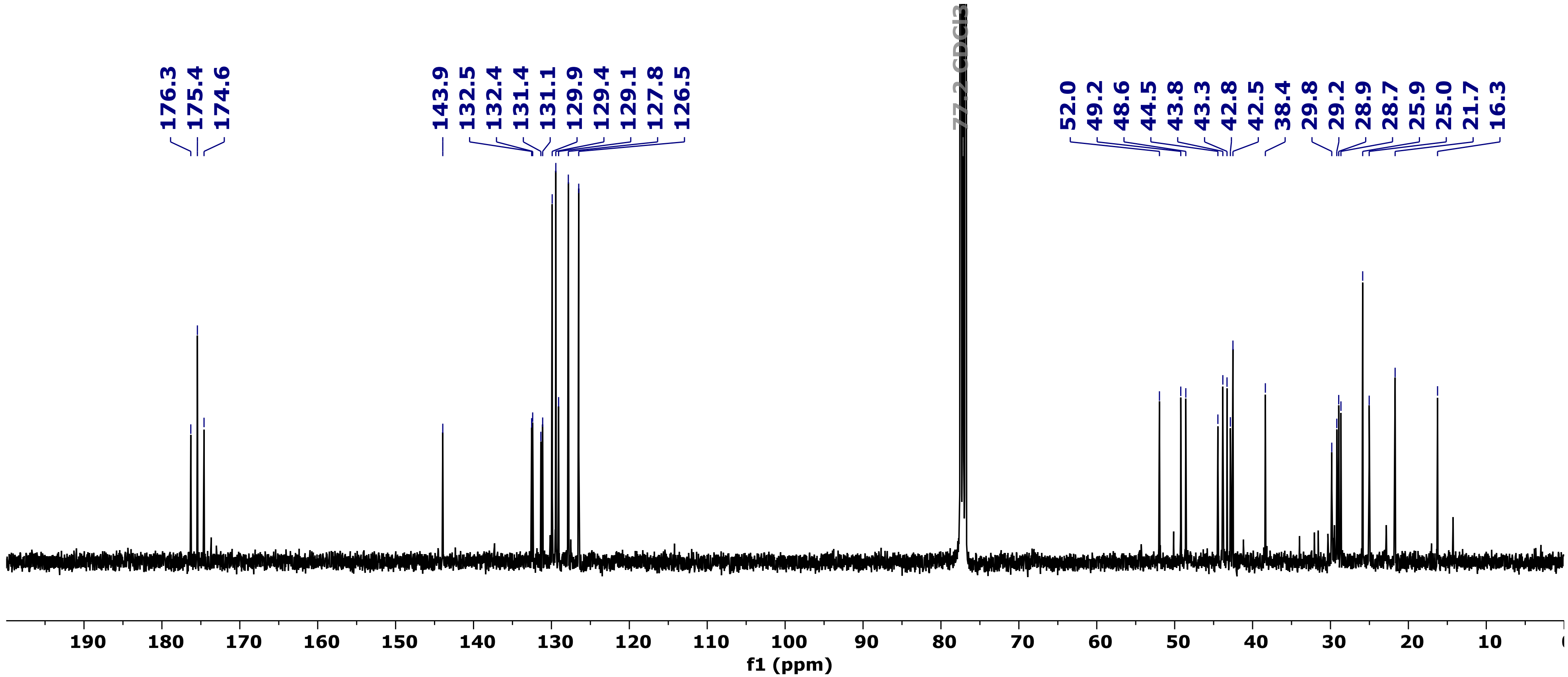

2D NMR HSQC

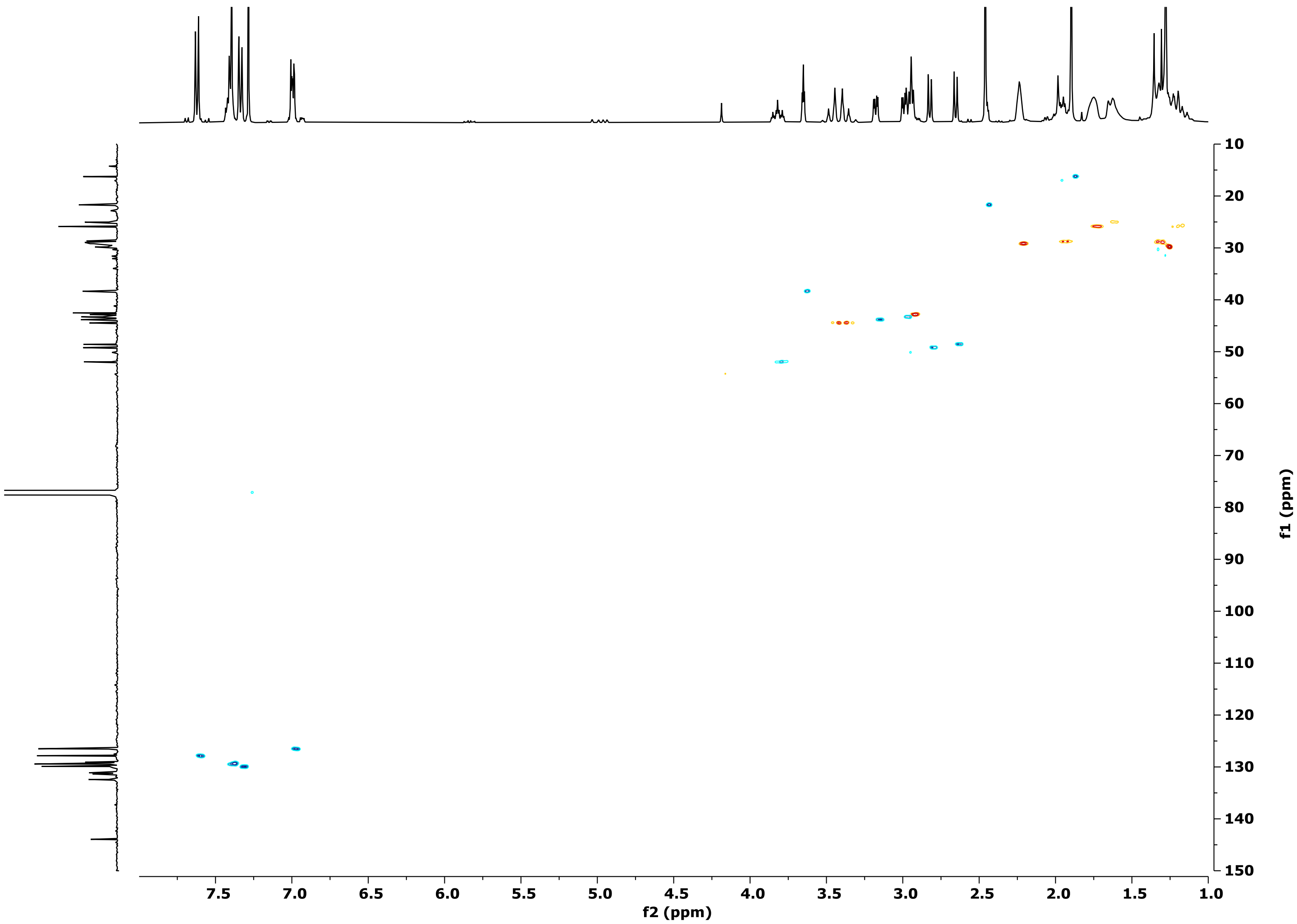

10
20
30
40
50
60
70
80
90
100
110
120
130
140
150
f1 (ppm)
7.5
7.0
6.5
6.0
5.5
5.0
4.5
4.0
3.5
3.0
2.5
2.0
1.5
1.0
f2 (ppm)

2D NMR COSY

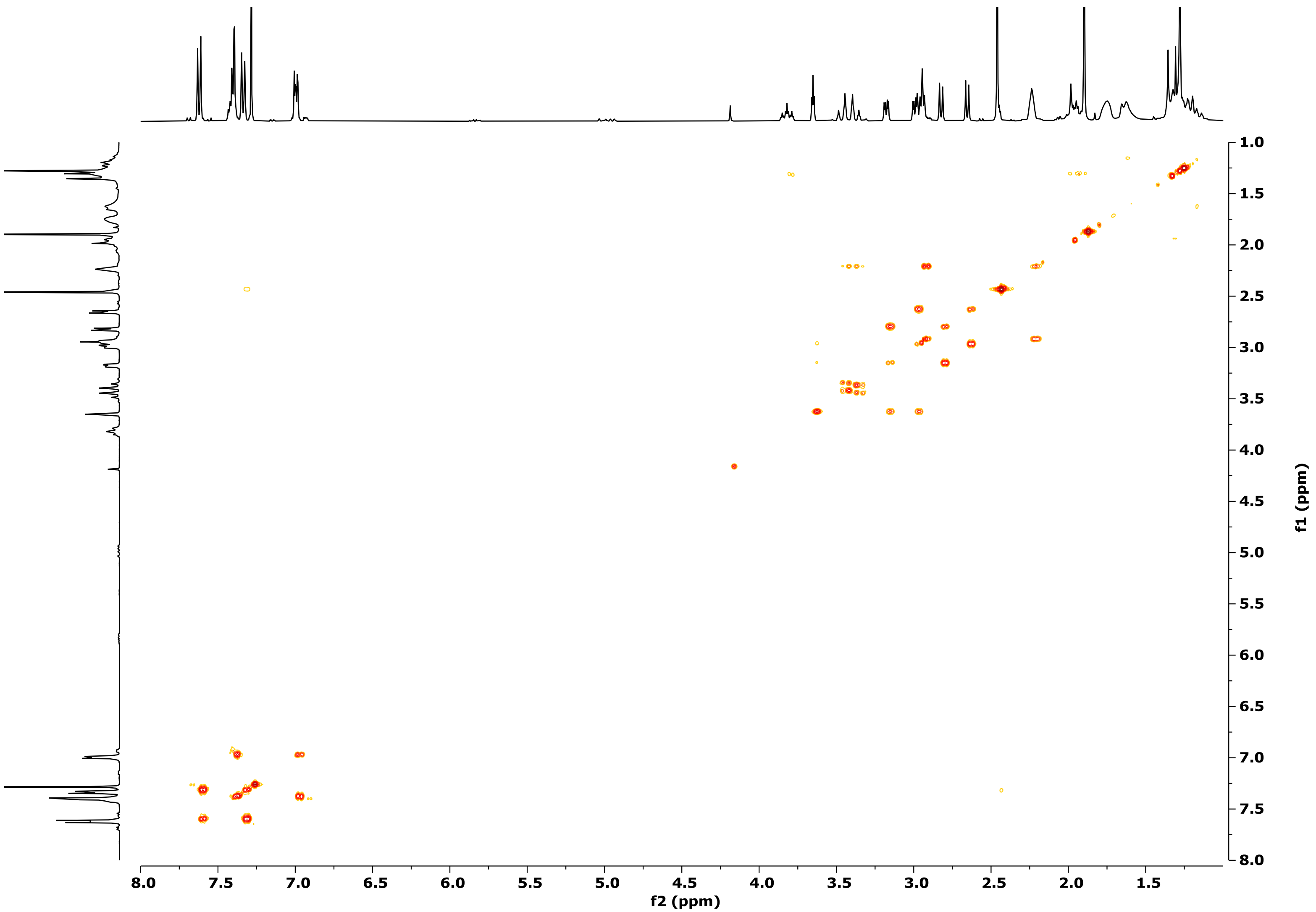

8.0
7.5
7.0
6.5
6.0
5.5
5.0
4.5
4.0
3.5
3.0
2.5
2.0
1.5
f2 (ppm)
1.0
1.5
2.0
2.5
3.0
3.5
4.0
4.5
5.0
5.5
6.0
6.5
7.0
7.5
8.0
f1 (ppm)

S5. Crystal structure of compound 3a

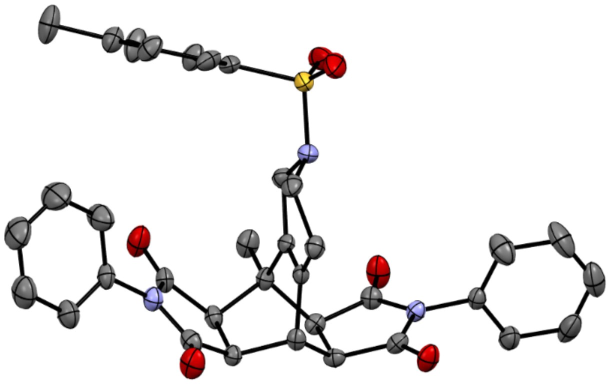

Figure S9. *ORTEP representation of compound* ***3a*** *with a probability level of 30%*

Colourless prism-like crystals of **3a** were grown at 2-6 °C using a layered solution approach. The bottom solution layer, contained within a long straight tube, consisted of the compound **3a** dissolved in CH_2Cl_2. Pentane was carefully placed on top using a syringe

A colorless, prism-like specimen of **3a**, approximate dimensions 0.080 mm x 0.120 mm x 0.130 mm, was used for the X-ray crystallographic analysis. The X-ray intensity data were measured on a D8 QUEST ECO three-circle diffractometer system equipped with a Ceramic x-ray tube (Mo Kα, λ = 0.71073 Å) and a doubly curved silicon crystal Bruker Triumph monochromator.

A total of 1385 frames were collected. The total exposure time was 11.54 hours. The frames were integrated with the Bruker SAINT software package using a narrow-frame algorithm. The integration of the data using a monoclinic unit cell yielded a total of 151065 reflections to a maximum θ angle of 26.37° (0.80 Å resolution), of which 14153 were independent (average redundancy 10.674, completeness = 99.8%, R_{int} = 9.77%, R_{sig} = 4.71%) and 10795 (76.27%) were greater than $2\sigma(F^2)$. The final cell constants of a = 10.3903(7) Å, b = 25.7826(16) Å, c = 12.9948(8) Å, β = 94.796(2)°, volume = 3469.0(4) $Å^3$, are based upon the refinement of the XYZ-centroids of 9791 reflections above 20 σ(I) with 5.079° < 2θ < 53.23°. Data were corrected for absorption effects using the Multi-Scan method (SADABS). The ratio of minimum to maximum apparent transmission was 0.929. The calculated minimum and maximum transmission coefficients (based on crystal size) are 0.9530 and 0.9710.

The structure was solved and refined using the Bruker SHELXTL Software Package, using the space group P 1 21 1, with Z = 4 for the formula unit, $C_{36}H_{32}Cl_3N_3O_6S$. The final anisotropic full-matrix least-squares refinement on F^2 with 920 variables converged at R1 = 7.91%, for the observed data and wR2 = 15.06% for all data. The goodness-of-fit was 1.124. The largest peak in the final difference electron density synthesis was 0.384 $e^-/Å^3$ and the largest hole

was -0.552 e^{-}/Å^{3} with an RMS deviation of 0.049 e^{-}/Å^{3}. On the basis of the final model, the calculated density was 1.419 g/cm^{3} and F(000), 1536 e^{-}.

Table S2. Sample and crystal data for NR_Ph_DDA.

**Identification code**	NR_Ph_DDA	
**Chemical formula**	$C_{36}H_{32}Cl_3N_3O_6S$	
**Formula weight**	741.05 g/mol	
**Temperature**	307(2) K	
**Wavelength**	0.71073 Å	
**Crystal size**	0.080 x 0.120 x 0.130 mm	
**Crystal habit**	colorless prism	
**Crystal system**	monoclinic	
**Space group**	P 1 21 1	
**Unit cell dimensions**	a = 10.3903(7) Å	α = 90°
	b = 25.7826(16) Å	β = 94.796(2)°
	c = 12.9948(8) Å	γ = 90°
**Volume**	3469.0(4) Å^{3}	
**Z**	4	
**Density (calculated)**	1.419 g/cm^{3}	
**Absorption coefficient**	0.375 mm^{-1}	
**F(000)**	1536	

Table S3. Data collection and structure refinement for NR_Ph_DDA.

**Diffractometer**	D8 QUEST ECO three-circle diffractometer
**Radiation source**	Ceramic x-ray tube (Mo Kα, λ = 0.71073 Å)
**Theta range for data collection**	2.41 to 26.37°
**Index ranges**	-12<=h<=12, -32<=k<=32, -16<=l<=16
**Reflections collected**	151065
**Independent reflections**	14153 [R(int) = 0.0977]
**Coverage of independent reflections**	99.8%

**Absorption correction**	Multi-Scan	
**Max. and min. transmission**	0.9710 and 0.9530	
**Structure solution technique**	direct methods	
**Structure solution program**	XT, VERSION 2018/2	
**Refinement method**	Full-matrix least-squares on F^2	
**Refinement program**	SHELXL-2019/1 (Sheldrick, 2019)	
**Function minimized**	$\Sigma w(F_o^2 - F_c^2)^2$	
**Data / restraints / parameters**	14153 / 1 / 920	
**Goodness-of-fit on F^2**	1.124	
**Final R indices**	10795 data; I>2σ(I)	R1 = 0.0791, wR2 = 0.1393
	all data	R1 = 0.1048, wR2 = 0.1506
**Weighting scheme**	$w=1/[\sigma^2(F_o^2)+(0.0373P)^2+3.9270P]$ where $P=(F_o^2+2F_c^2)/3$	
**Absolute structure parameter**	0.04(2)	
**Largest diff. peak and hole**	0.384 and -0.552 eÅ^{-3}	
**R.M.S. deviation from mean**	0.049 eÅ^{-3}	

Table S4. Atomic coordinates and equivalent isotropic atomic displacement parameters (Å2) for NR_Ph_DDA.

U(eq) is defined as one third of the trace of the orthogonalized U_{ij} tensor.

	x/a	y/b	z/c	U(eq)
S1	0.8388(2)	0.36592(9)	0.56938(17)	0.0569(6)
O2	0.8876(6)	0.3278(3)	0.6427(4)	0.078(2)
O3	0.9180(6)	0.4085(3)	0.5439(6)	0.087(2)
O23	0.3274(7)	0.2935(2)	0.5302(5)	0.0708(18)
O30	0.2016(6)	0.4617(2)	0.4928(4)	0.0648(16)
O31	0.5225(6)	0.3397(2)	0.9724(4)	0.0658(17)
O38	0.4647(6)	0.50887(19)	0.8857(4)	0.0544(14)
N4	0.7086(5)	0.3907(3)	0.6152(5)	0.0460(15)
N10	0.2578(6)	0.3755(2)	0.4862(4)	0.0457(14)
N19	0.5151(5)	0.4272(2)	0.9460(4)	0.0358(13)
C5	0.6207(7)	0.3528(3)	0.6552(6)	0.0441(17)

	x/a	y/b	z/c	U(eq)
C6	0.5027(6)	0.3791(3)	0.6891(5)	0.0348(15)
C7	0.3993(7)	0.3478(2)	0.7375(5)	0.0362(15)
C8	0.2737(7)	0.3544(3)	0.6645(5)	0.0390(16)
C9	0.2921(7)	0.3359(3)	0.5552(5)	0.0436(17)
C11	0.2287(7)	0.4209(3)	0.5342(5)	0.0440(18)
C12	0.2382(7)	0.4120(3)	0.6486(5)	0.0362(15)
C13	0.3465(7)	0.4454(2)	0.7043(5)	0.0354(15)
C14	0.4740(6)	0.4288(2)	0.6700(5)	0.0345(15)
C15	0.5558(7)	0.4631(3)	0.6112(5)	0.0432(17)
C16	0.6436(7)	0.4321(3)	0.5492(6)	0.0491(19)
C17	0.3693(7)	0.3762(2)	0.8411(5)	0.0332(15)
C18	0.4777(7)	0.3765(3)	0.9258(5)	0.0409(16)
C20	0.4461(7)	0.4626(3)	0.8844(5)	0.0406(16)
C21	0.3434(7)	0.4348(3)	0.8195(5)	0.0367(15)
C22	0.4302(9)	0.2906(3)	0.7591(6)	0.055(2)
C24	0.2543(7)	0.3701(3)	0.3757(5)	0.0439(16)
C25	0.3587(9)	0.3512(4)	0.3313(7)	0.076(3)
C26	0.3559(11)	0.3471(5)	0.2251(8)	0.099(4)
C27	0.2487(12)	0.3605(5)	0.1655(8)	0.090(3)
C28	0.1453(11)	0.3805(4)	0.2097(7)	0.082(3)
C29	0.1464(9)	0.3848(3)	0.3162(6)	0.062(2)
C32	0.6071(7)	0.4426(2)	0.0302(5)	0.0372(16)
C33	0.5636(8)	0.4516(3)	0.1244(6)	0.056(2)
C34	0.6504(12)	0.4698(4)	0.2049(7)	0.076(3)
C35	0.7756(12)	0.4778(4)	0.1879(9)	0.078(3)
C36	0.8183(10)	0.4686(4)	0.0935(9)	0.086(3)
C37	0.7333(9)	0.4509(4)	0.0138(7)	0.072(3)
C39	0.7870(7)	0.3340(3)	0.4532(6)	0.0460(18)
C40	0.7842(8)	0.3612(3)	0.3608(6)	0.051(2)
C41	0.7409(9)	0.3365(3)	0.2705(6)	0.058(2)

	x/a	y/b	z/c	U(eq)
C42	0.7014(9)	0.2852(3)	0.2712(6)	0.056(2)
C43	0.7035(10)	0.2604(4)	0.3648(7)	0.070(3)
C44	0.7457(9)	0.2837(3)	0.4554(6)	0.055(2)
C45	0.6584(12)	0.2583(4)	0.1701(7)	0.086(3)
Cl2N	0.6117(3)	0.71271(13)	0.0002(2)	0.1019(10)
Cl3N	0.6496(3)	0.60237(11)	0.9975(3)	0.0931(9)
Cl4N	0.8621(3)	0.66971(15)	0.9692(3)	0.1303(14)
C1N	0.7220(9)	0.6629(4)	0.0278(7)	0.076(3)
Cl2M	0.0699(14)	0.2900(4)	0.9216(16)	0.117(5)
Cl3M	0.8112(6)	0.3323(2)	0.9306(9)	0.070(3)
Cl2"	0.0706(10)	0.2847(4)	0.8613(8)	0.087(3)
Cl3"	0.8868(17)	0.3166(4)	0.9982(10)	0.146(7)
Cl4M	0.0252(3)	0.39579(11)	0.9003(3)	0.1050(11)
C1M	0.9579(9)	0.3338(4)	0.8810(8)	0.076(3)
S1A	0.8460(2)	0.62912(12)	0.33160(19)	0.0740(8)
O2A	0.7675(7)	0.5861(4)	0.3501(6)	0.113(3)
O3A	0.7985(6)	0.6678(4)	0.2583(5)	0.106(3)
O23A	0.3719(6)	0.7054(2)	0.3649(4)	0.0642(16)
O30A	0.4951(6)	0.5374(2)	0.4075(4)	0.0604(15)
O31A	0.1523(6)	0.6543(2)	0.9293(4)	0.0600(15)
O38A	0.2107(6)	0.48579(18)	0.0197(4)	0.0561(15)
N4A	0.9794(6)	0.6061(3)	0.2896(5)	0.0538(17)
N10A	0.4443(6)	0.6244(2)	0.4099(4)	0.0472(15)
N19A	0.1597(6)	0.5670(2)	0.9584(4)	0.0390(13)
C5A	0.0664(7)	0.6449(3)	0.2517(6)	0.0493(19)
C6A	0.1849(6)	0.6192(3)	0.2170(5)	0.0344(15)
C7A	0.2853(6)	0.6487(2)	0.1609(5)	0.0353(15)
C8A	0.4125(7)	0.6419(2)	0.2318(5)	0.0362(16)
C9A	0.4061(7)	0.6635(3)	0.3402(6)	0.0443(17)
C11A	0.4677(7)	0.5766(3)	0.3636(6)	0.0406(16)

	x/a	y/b	z/c	U(eq)
C12A	0.4477(7)	0.5842(2)	0.2485(5)	0.0357(15)
C13A	0.3364(7)	0.5497(3)	0.2003(5)	0.0378(16)
C14A	0.2120(7)	0.5697(3)	0.2360(5)	0.0366(15)
C15A	0.1301(8)	0.5338(3)	0.2935(6)	0.056(2)
C16A	0.0441(9)	0.5665(4)	0.3564(7)	0.068(2)
C17A	0.3094(6)	0.6181(3)	0.0621(5)	0.0340(15)
C18A	0.1990(7)	0.6183(3)	0.9771(5)	0.0354(15)
C20A	0.2308(7)	0.5312(3)	0.0205(5)	0.0390(16)
C21A	0.3355(7)	0.5606(3)	0.0834(5)	0.0383(16)
C22A	0.2543(9)	0.7054(3)	0.1379(7)	0.054(2)
C24A	0.4793(13)	0.6358(5)	0.5162(9)	0.055(3)
C25A	0.5572(10)	0.6747(4)	0.5481(9)	0.056(3)
C26A	0.5906(13)	0.6830(6)	0.6517(12)	0.080(4)
C27A	0.5382(19)	0.6502(7)	0.7206(11)	0.093(6)
C28A	0.4629(18)	0.6099(7)	0.6915(10)	0.094(5)
C29A	0.4274(13)	0.6031(5)	0.5858(8)	0.062(3)
C24'	0.425(2)	0.6224(9)	0.5258(12)	0.0439(16)
C25'	0.5401(16)	0.6182(12)	0.5878(17)	0.076(3)
C26'	0.5390(19)	0.6191(14)	0.6947(16)	0.099(4)
C27'	0.423(2)	0.6241(13)	0.7395(12)	0.090(3)
C28'	0.3076(18)	0.6283(10)	0.6774(15)	0.082(3)
C29'	0.3087(16)	0.6274(8)	0.5706(14)	0.063(9)
C32A	0.0686(7)	0.5508(3)	0.8749(6)	0.0440(18)
C33A	0.9418(9)	0.5458(4)	0.8883(7)	0.066(2)
C34A	0.8566(10)	0.5299(5)	0.8075(9)	0.087(3)
C35A	0.8999(11)	0.5178(4)	0.7163(8)	0.074(3)
C36A	0.0274(11)	0.5239(4)	0.7004(7)	0.074(3)
C37A	0.1123(8)	0.5410(4)	0.7815(6)	0.064(2)
C39A	0.8858(7)	0.6609(4)	0.4497(6)	0.053(2)
C40A	0.9127(9)	0.7134(4)	0.4519(7)	0.067(2)

	x/a	y/b	z/c	U(eq)
C41A	0.9426(9)	0.7369(3)	0.5462(7)	0.063(2)
C42A	0.9443(8)	0.7102(4)	0.6365(6)	0.058(2)
C43A	0.9196(10)	0.6587(4)	0.6323(7)	0.073(3)
C44A	0.8908(10)	0.6336(4)	0.5404(7)	0.073(3)
C45A	0.9709(12)	0.7377(4)	0.7387(8)	0.092(3)

S6. Crystal structure of compound 3b

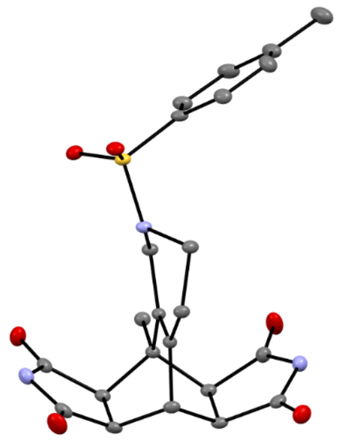

Figure S10. *ORTEP representation of compound **3b** with a probability level of 30%*

Colourless needle-like crystals of **3b** were grown at 2-6 °C using a layered solution approach. The bottom solution layer, contained within a long straight tube, consisted of the compound **3b** dissolved in CH_2Cl_2. Pentane was carefully placed on top using a syringe

A colorless, block-like specimen of **3b**, approximate dimensions 0.100 mm x 0.150 mm x 0.180 mm, was used for the X-ray crystallographic analysis. The X-ray intensity data were measured on a D8 QUEST ECO three-circle diffractometer system equipped with a Ceramic x-ray tube (Mo Kα, λ = 0.71073 Å) and a doubly curved silicon crystal Bruker Triumph monochromator.

A total of 733 frames were collected. The total exposure time was 2.04 hours. The frames were integrated with the Bruker SAINT software package using a narrow-frame algorithm. The integration of the data using a triclinic unit cell yielded a total of 72181 reflections to a maximum θ angle of 27.70° (0.76 Å resolution), of which 7172 were independent (average redundancy 10.064, completeness = 99.5%, R_{int} = 5.92%, R_{sig} = 3.10%) and 5757 (80.27%) were greater than 2σ(F^2). The final cell constants of a = 10.3852(5) Å, b = 12.1470(5) Å, c = 14.2346(5) Å, α = 109.6360(10)°, β = 93.9640(10)°, γ = 111.083(2)°, volume = 1540.72(11) $Å^3$, are based upon the refinement of the XYZ-centroids of 9409 reflections above 20 σ(I) with 5.776° < 2θ < 55.20°. Data were corrected for

absorption effects using the Multi-Scan method (SADABS). The ratio of minimum to maximum apparent transmission was 0.943. The calculated minimum and maximum transmission coefficients (based on crystal size) are 0.9710 and 0.9840.

The structure was solved and refined using the Bruker SHELXTL Software Package, using the space group P -1, with Z = 2 for the formula unit, $C_{29}H_{37}N_5O_8S$. The final anisotropic full-matrix least-squares refinement on F^2 with 442 variables converged at R1 = 4.53%, for the observed data and wR2 = 10.58% for all data. The goodness-of-fit was 1.065. The largest peak in the final difference electron density synthesis was 0.511 $e^-/Å^3$ and the largest hole was -0.444 $e^-/Å^3$ with an RMS deviation of 0.051 $e^-/Å^3$. On the basis of the final model, the calculated density was 1.327 g/cm^3 and F(000), 652 e^-.

Table S5. Sample and crystal data for ER058_F5.

**Identification code**	ER058_F5	
**Chemical formula**	$C_{29}H_{37}N_5O_8S$	
**Formula weight**	615.69 g/mol	
**Temperature**	100(2) K	
**Wavelength**	0.71073 Å	
**Crystal size**	0.100 x 0.150 x 0.180 mm	
**Crystal habit**	colorless block	
**Crystal system**	triclinic	
**Space group**	P -1	
**Unit cell dimensions**	a = 10.3852(5) Å	α = 109.6360(10)°
	b = 12.1470(5) Å	β = 93.9640(10)°
	c = 14.2346(5) Å	γ = 111.083(2)°
**Volume**	1540.72(11) $Å^3$	
**Z**	2	
**Density (calculated)**	1.327 g/cm^3	
**Absorption coefficient**	0.162 mm^{-1}	
**F(000)**	652	

Table S6. Data collection and structure refinement for ER058_F5.

**Diffractometer**	D8 QUEST ECO three-circle diffractometer	
**Radiation source**	Ceramic x-ray tube (Mo Kα, λ = 0.71073 Å)	
**Theta range for data collection**	2.15 to 27.70°	
**Index ranges**	-13<=h<=13, -15<=k<=15, -18<=l<=18	
**Reflections collected**	72181	
**Independent reflections**	7172 [R(int) = 0.0592]	
**Coverage of independent reflections**	99.5%	
**Absorption correction**	Multi-Scan	
**Max. and min. transmission**	0.9840 and 0.9710	
**Structure solution technique**	direct methods	
**Structure solution program**	XT, VERSION 2018/2	
**Refinement method**	Full-matrix least-squares on F^2	
**Refinement program**	SHELXL-2019/1 (Sheldrick, 2019)	
**Function minimized**	Σ w(F_o^2 - F_c^2)^2	
**Data / restraints / parameters**	7172 / 0 / 442	
**Goodness-of-fit on F^2**	1.065	
**Final R indices**	5757 data; I>2σ(I)	R1 = 0.0453, wR2 = 0.0961
	all data	R1 = 0.0637, wR2 = 0.1058
**Weighting scheme**	w=1/[$\sigma^2(F_o^2)$+(0.0347P)^2+1.1566P]<br>where P=(F_o^2+2F_c^2)/3	
**Largest diff. peak and hole**	0.511 and -0.444 eÅ^{-3}	
**R.M.S. deviation from mean**	0.051 eÅ^{-3}	

Table S7. Atomic coordinates and equivalent isotropic atomic displacement parameters (Å^2) for ER058_F5.

U(eq) is defined as one third of the trace of the orthogonalized U_{ij} tensor.

	x/a	y/b	z/c	U(eq)
S23	0.19019(4)	0.45374(4)	0.20676(3)	0.01823(10)
O19	0.82836(14)	0.51176(14)	0.48872(9)	0.0297(3)
O20	0.87027(17)	0.73431(13)	0.28316(10)	0.0373(4)
O21	0.45604(16)	0.04631(13)	0.16120(11)	0.0348(3)
O22	0.48599(14)	0.24438(13)	0.93836(9)	0.0286(3)
O24	0.16258(13)	0.41894(12)	0.09854(9)	0.0240(3)
O25	0.08573(13)	0.38712(12)	0.25157(9)	0.0225(3)
N1	0.85590(16)	0.64668(15)	0.40368(11)	0.0232(3)
N9	0.44433(16)	0.12546(14)	0.03689(11)	0.0230(3)
N15	0.33202(15)	0.43179(14)	0.23563(10)	0.0175(3)
C2	0.83324(17)	0.53157(18)	0.41062(13)	0.0215(4)
C3	0.81313(18)	0.43434(17)	0.30484(12)	0.0191(3)
C4	0.82050(17)	0.50696(16)	0.23369(12)	0.0183(3)
C5	0.85163(19)	0.64280(18)	0.30553(13)	0.0237(4)
C6	0.66704(18)	0.32182(16)	0.27348(12)	0.0189(3)
C7	0.65362(19)	0.23263(17)	0.16287(13)	0.0208(3)
C8	0.5090(2)	0.12310(17)	0.12299(14)	0.0245(4)
C10	0.52426(19)	0.22581(16)	0.01150(13)	0.0210(4)
C11	0.66248(18)	0.30491(16)	0.09097(12)	0.0186(3)
C12	0.67968(17)	0.44480(16)	0.15205(12)	0.0166(3)
C13	0.55982(17)	0.43423(15)	0.20914(12)	0.0157(3)
C14	0.45140(17)	0.48377(17)	0.19044(13)	0.0183(3)
C16	0.37634(19)	0.44980(18)	0.34250(12)	0.0205(3)
C17	0.45264(18)	0.36345(17)	0.34176(12)	0.0186(3)
C18	0.55583(17)	0.37443(16)	0.27292(12)	0.0165(3)
C26	0.23272(18)	0.61836(17)	0.26579(13)	0.0203(3)
C27	0.21329(19)	0.66983(18)	0.36465(14)	0.0246(4)
C28	0.2527(2)	0.80053(19)	0.41000(15)	0.0304(4)

	x/a	y/b	z/c	U(eq)
C29	0.3133(2)	0.88166(18)	0.36032(16)	0.0291(4)
C30	0.3326(2)	0.82829(19)	0.26267(16)	0.0296(4)
C31	0.29255(19)	0.69746(18)	0.21483(14)	0.0253(4)
C32	0.3569(2)	0.0244(2)	0.41192(19)	0.0407(5)
C33	0.68651(18)	0.51824(16)	0.08234(12)	0.0195(3)
O1N	0.91814(16)	0.88183(14)	0.55747(11)	0.0372(4)
N3N	0.13503(17)	0.93978(15)	0.65087(11)	0.0256(3)
C2N	0.0397(2)	0.96147(19)	0.60211(14)	0.0304(4)
C4N	0.1017(2)	0.8159(2)	0.65756(17)	0.0378(5)
C5N	0.2769(2)	0.0379(2)	0.69939(18)	0.0431(5)
O1M	0.1681(3)	0.9857(3)	0.9227(2)	0.0319(7)
N3M	0.9924(3)	0.0398(3)	0.8787(2)	0.0277(8)
C2M	0.1200(4)	0.0675(4)	0.9232(3)	0.0308(9)
C4M	0.8888(5)	0.9105(4)	0.8232(4)	0.0331(10)
C5M	0.9446(6)	0.1419(4)	0.8835(4)	0.0438(11)
O1'	0.8209(4)	0.0556(4)	0.0536(3)	0.0321(10)
N3'	0.9269(5)	0.0640(4)	0.9185(3)	0.0352(11)
C2'	0.9036(5)	0.1134(5)	0.0107(4)	0.0344(12)
C4'	0.0298(11)	0.1439(7)	0.8768(6)	0.072(3)
C5'	0.8488(8)	0.9321(7)	0.8534(5)	0.0482(18)

S7. Computational study

S7.1. Computational details

Geometries of all stationary points were optimized without symmetry constraint with the Gaussian 16 program[3] using the DFT B3LYP hybrid exchange-correlation functional.[4] The all-electron cc-pVDZ basis set[5] was employed for non-metal atoms and the cc-pVDZ-PP basis set containing an effective core relativistic pseudopotential for Rh.[6] The electronic energy was improved by performing single point energy calculations with the cc-pVTZ (cc-pVTZ-PP for Rh) basis set and the M06L functional[7] and including solvent effects corrections computed with the

solvent model based on density (SMD) continuum solvation.[8] To mimic the experimental solvent mixture with a molar fraction ratio of 79:21 of dichloroethane:ethanol, the values of the solvent descriptors used in the SMD solvation model were re-defined on the basis of a linear behavior with the molar fraction. Using the “Solvent=(Generic,Read)” options of the Gaussian09 SCRF keyword, the solvent mixture was defined employing the following solvent descriptors: Dynamic Dielectric Constant=9.24; Static Dielectric Constant=1.427; Abraham’s hydrogen bond acidity=0.157; Abraham’s hydrogen bond basicity=0.189; Surface Tension=42.82; Carbon Aromaticity=0; Electronegativity Halogenicity=0.394. The D3 Grimme energy corrections for dispersion[9] with the original damping function were added in all B3LYP/cc-pVDZ-PP and M06L/cc-pVTZ-PP calculations. Analytical Hessians were computed to determine the nature of stationary points (one and zero imaginary frequencies for TSs and minima, respectively) and to calculate unscaled zero-point energies (ZPEs) as well as thermal corrections and entropy effects using the standard statistical-mechanics relationships for an ideal gas.[10] These two latter terms were computed at 353.15 K and 1 atm to provide the reported relative Gibbs energies. As a summary, the reported Gibbs energies contain electronic energies including solvent effects calculated at the M06L-D3/cc-pVTZ-PP//B3LYP-D3/cc-pVDZ-PP level together with gas phase thermal and entropic contributions computed at 313.15 K and 1 atm with the B3LYP-D3/cc-pVDZ-PP method. All computational data concerning reaction mechanisms was uploaded to the ioChem-BD repository[11] and is available through the following link: https://iochem.udg.edu/browse/handle/100/7462.

S7.2. Reaction mechanism

To get full insight into the chemoselectivity of the process, we simulated the Rh(DPEphos)-catalyzed reaction between substrate **1a** and methyl maleimide at the M06L-D3/cc-pVTZ-PP/SMD(79% Dichloroethane, 21% Ethanol)//B3LYP-D3/cc-pVDZ-PP level of at 353 K. The results obtained are summarized in **Scheme 3** and **Figures S12-14**.

As reported previously,[1] the reaction starts with coordination of the Rh(DPEphos) complex to allenyne **1a** to forming Rh(**1a**), which is taken as the reference point ($\Delta G = 0.0$ kcal mol^{-1}) in **Figure S12**. Oxidative coupling through **TS1** ($\Delta G^{\neq}$=14.7 kcal mol^{-1}) leads to rhodacyclopentene intermediate **I**, releasing 19.4 kcal·mol^{-1}. This intermediate can coordinate to two TFA molecules present in the reaction medium, thus delivering intermediate **I**(TFA)$_2$, an octahedral 18-electron intermediate. Subsequently, one of the coordinated TFA ligands transfers its proton, yielding Rh(III) intermediate **II**(TFA)$_2$, which lies 9.0 kcal·mmol^{-1} higher in the Gibbs energy surface (ΔG=-10.4 kcal mol^{-1}). Overall, coordination and proton transfer *via* **TS2** require an activation free energy of 11.4 kcal.mol^{-1}. This same process was estimated to have a much higher Gibbs energy barrier of 28.4 kcal·mol^{-1} when H_2O was considered as the proton source,[1] consistent with the greater acidity of TFA. From intermediate **II**(TFA)$_2$, the resulting trifluoroacetate anion can be reprotonated to regenerate TFA through proton abstraction at two different positions, ultimately leading to the formation of products **3c** or **4c**. Abstraction from the methylene group vicinal to the Rh center (**TS 3**) demands only 1.0 kcal·mol^{-1} and produces rhodacyclopentadiene intermediate **III**(TFA)$_2$ in a slightly endergonic process. Importantly, the reversibility of the proton shifts accounts for the observed deuterium scrambling in the labelling experiments, as an equilibrium is established that allows hydrogen migration in both directions. Proton abstraction is rapidly followed by exergonic loss of one TFA ligand and a barrierless Rh-mediated [4+2] cycloaddition with maleimide (See **Figure S14**). The latter step takes place selectively with an *endo* approach, favored by steric congestion from the phosphine ligand. Overall, these three steps from **II**(TFA)$_2$ to cycloadduct **IV**(TFA) release 17.3 kcal·mol^{-1}. Finally, reductive elimination (**TS4**) coupled with TFA loss through a low Gibbs energy barrier of 3.5 kcal·mol^{-1} results in intermediate Rh(**RD**) (ΔG = -64.7 kcal mol^{-1}). Displacement with a new molecule of **1a** and release of the initially postulated diene intermediate **RD** closes the catalytic cycle. An additional uncatalyzed Diels-Alder cycloaddition with methyl maleimide

(**TS5**, $\Delta G^{\neq}$= 21.3 kcal·mol^{-1}), which again takes place selectively through *endo* approximation ultimately leads to product **3c**.

Alternatively, from **II**$(TFA)_2$, a barrierless proton abstraction at the methyl group (*alt* **TS3**) can from intermediate *alt* **II**$(TFA)_2$, which readily releases its two TFA ligands, delivering *alt* **III**. This intermediate is located slightly lower than **IV**(TFA) on the Gibbs energy surface (-28.8 kcal·mol^{-1}). However, reductive elimination from *alt* **III** (*alt* **TS 4**) has a much higher activation energy of 14.6 kcal·mol^{-1} and, moreover, it is endergonic by 9.9 kcal·mol^{-1}. Such a difference accounts for the chemoselective formation of product **3c** under the reaction conditions employed. Finally, substitution of the vinylallene intermediate **VA** by a new molecule of **1a** closes the catalytic cycle. **VA** participates in a Diels-Alder cycloaddition with maleimide analogously to **RD** to finally form product **4c** in a process that demands 25.6 kcal·mol^{-1} and that is exergonic by -9.2 kcal·mol^{-1}.

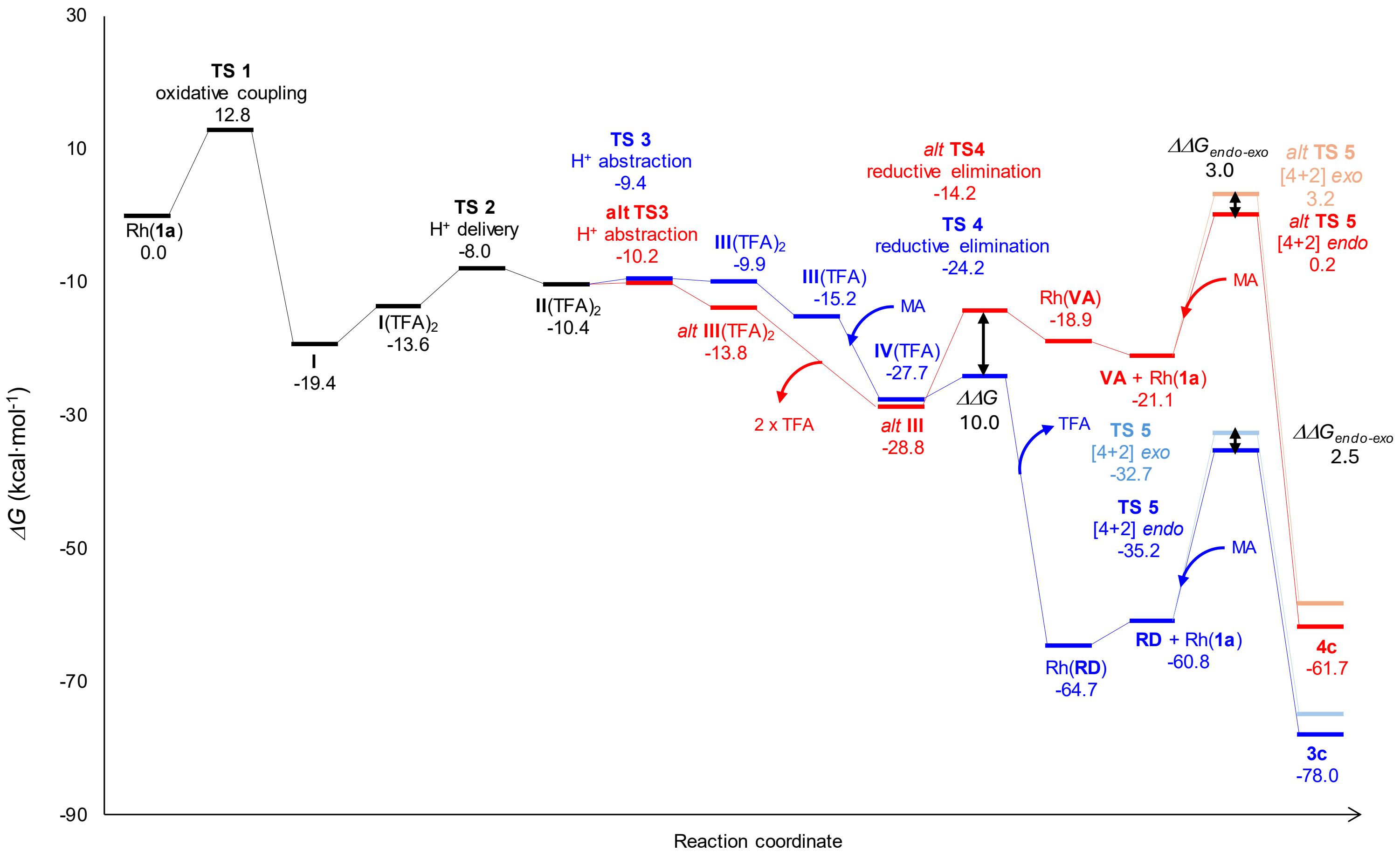

Figure S12. M06L-D3/cc-pVTZ-PP/SMD(79% Dichloroethane, 21% Ethanol)//B3LYP-D3/cc-pVDZ-PP (353 K) of the Rh(DPE)-catalyzed reaction between allenyne **1a** and maleimide. MA = maleimide; TFA = trifuloroacetic acid; VA = vinylallene.

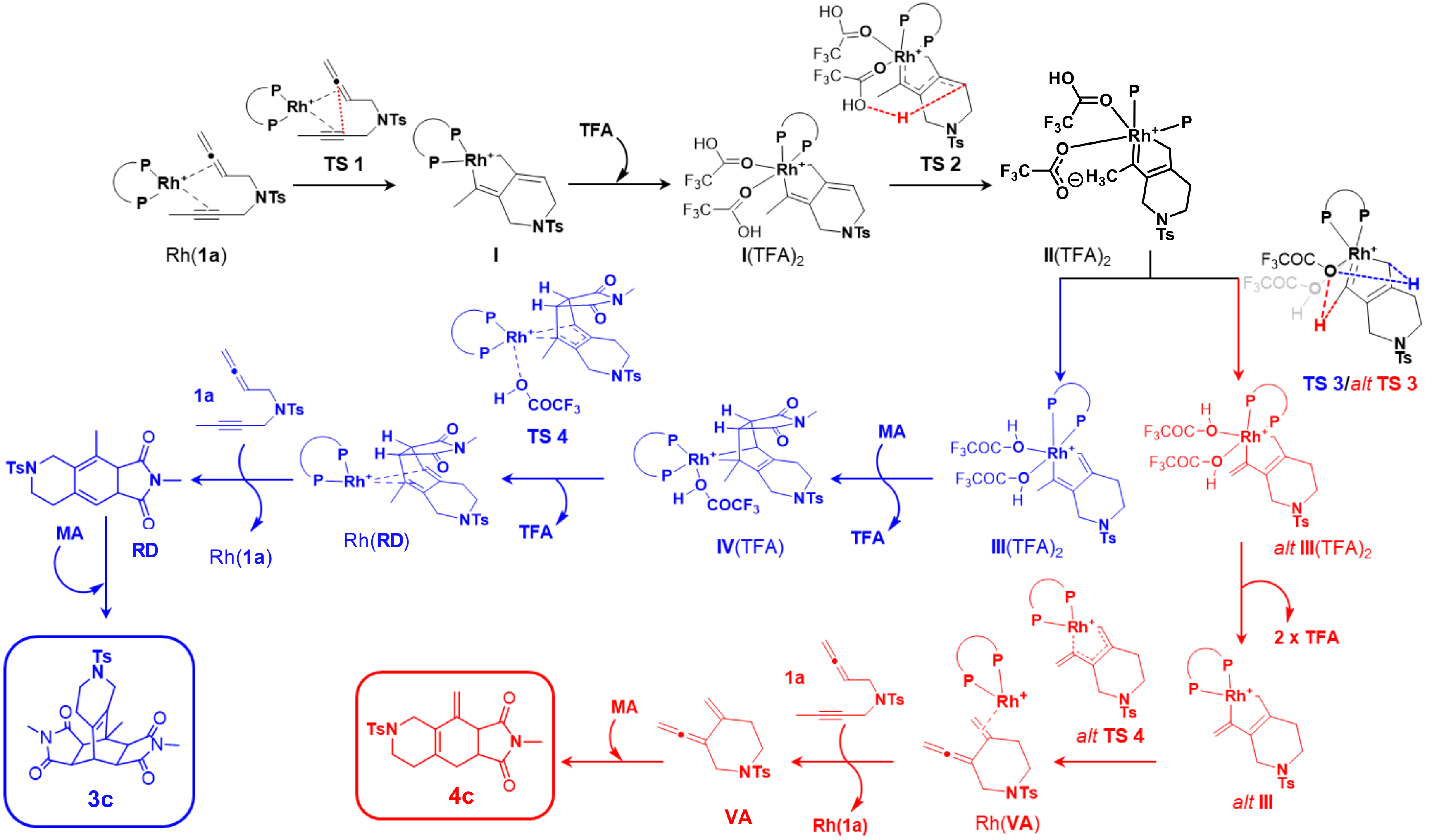

Figure S13. *Chemical structure of the intermediates and transition states of the Rh(DPE)-catalyzed reaction between allenyne **1a** and maleimide. MA = maleimide; TFA = trifuloroacetic acid; VA = vinylallene; RD = reactive diene.*

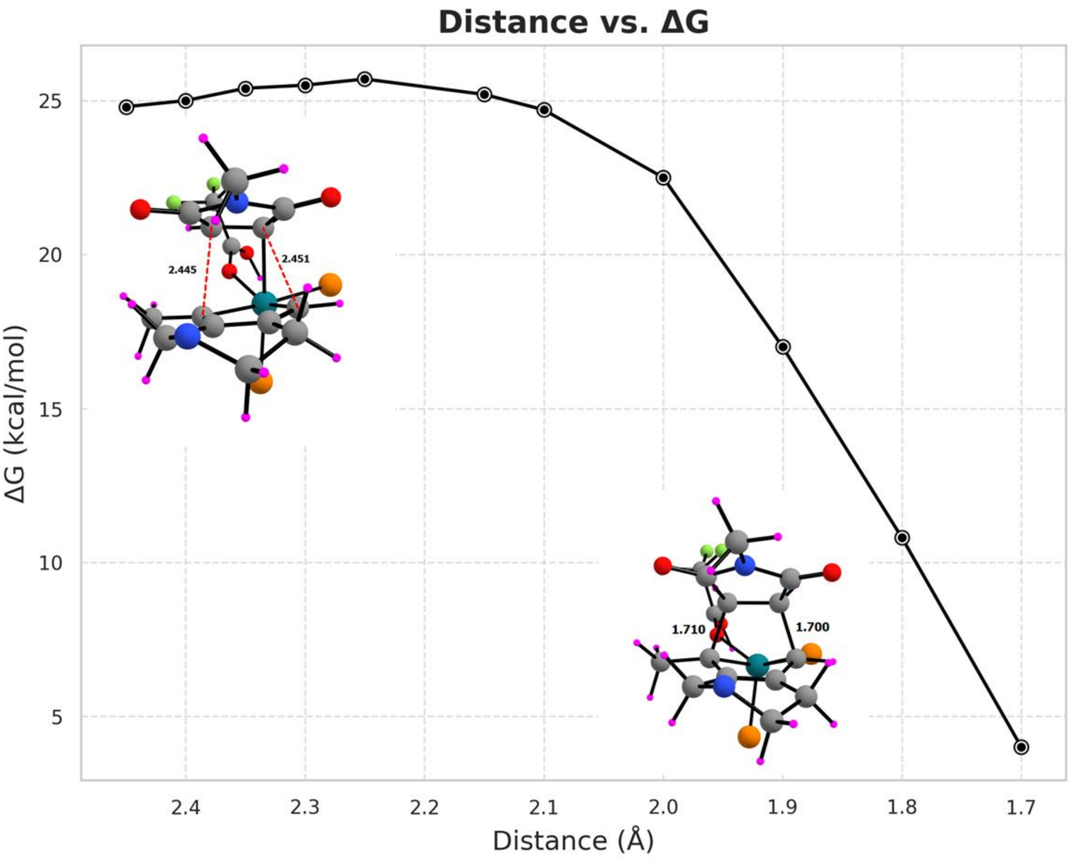

Figure S14. *BP86/cc-pVDZ relaxed scan between intermediates **III**(TFA) and **IV**(TFA). Selected substituents have been omitted for clarity.*

(a) (b)

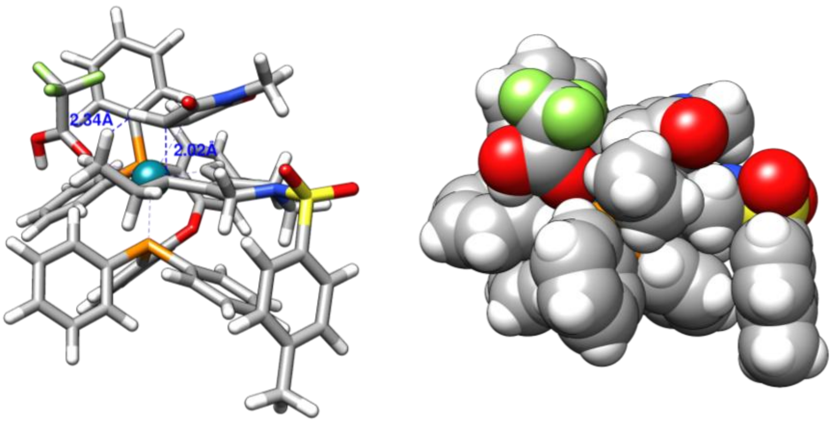

Figure S15. *(a) Ball and stick molecular model of the approximation between MA and intermediate **III(TFA)** considering **1k** as the allenyne substrate indicating key distances between the terminal methyl group and the maleimide and the rhodacyclopentadiene core and the maleimide. (b) Van der Waals molecular model fo the aproximation between MA and intermediate **III(TFA)** considering **1k** as the allenyne substrate showing the steric hindrance caused by the increased bulkyness of the ethyl group. The geometry was obtained via frozen optimization at the BP86-D3/cc-pVDZ-PP level of theory fixing the distance between the maleimide and the rhodacyclopentadiene core at 2.02 Å with the Gaussian16 keyword opt(modredundant,loose).*

References:

[1] a) Castanyer, C.; Artigas, A.; Solà, M.; Pla-Quintana, A. and Roglans, A., *Adv. Synth. Catal.* **2025**, *367*, e202401163. b) Deng, X.; Shi, L.Y.; Lan, J.; Guan, Y.Q.; Zhang, X.; Lv, H.; Chung, L.W. and Zhang, X. *Nat. Commun.* **2019**,10, 949 – 959. c) Park, J.H.; Kim, S.Y.; Kim, S.M.; Lee, S.I.; Chung, Y.K. *Synlett.* **2007**, 3, 453 – 459.

[2] a) Mandal, T.; Das, S.; Sarkar, S. D., *Adv. Synth. Catal.* **2019**, *361*, 3200 – 3209. b) Kumar, R.; Kumar, R.; Chandra, D.; Sharma, U. *J. Org. Chem.* **2019,** 84, 1542 – 1552. c) Mandal, T.; Das, S.; Maji, R.; De Sarkar, S. *Org. Lett.* **2023**, *25*, 7727-7732.

[3] M. J. Frisch, G. W. Trucks, H. B. Schlegel, G. E. Scuseria, M. a. Robb, J. R. Cheeseman, G. Scalmani, V. Barone, G. a. Petersson, H. Nakatsuji, X. Li, M. Caricato, a. V. Marenich, J. Bloino, B. G. Janesko, R. Gomperts, B. Mennucci, H. P. Hratchian, J. V. Ortiz, a. F. Izmaylov, J. L. Sonnenberg, Williams, F. Ding, F. Lipparini, F. Egidi, J. Goings, B. Peng, A. Petrone, T. Henderson, D. Ranasinghe, V. G. Zakrzewski, J. Gao, N. Rega, G. Zheng, W. Liang, M. Hada, M. Ehara, K. Toyota, R. Fukuda, J. Hasegawa, M. Ishida, T. Nakajima, Y. Honda, O. Kitao, H. Nakai, T. Vreven, K. Throssell, J. a. Montgomery Jr., J. E. Peralta, F. Ogliaro, M. J. Bearpark, J. J. Heyd, E. N. Brothers, K. N. Kudin, V. N. Staroverov, T. a. Keith, R. Kobayashi, J. Normand, K. Raghavachari, a. P. Rendell, J. C. Burant, S. S. Iyengar, J. Tomasi, M. Cossi, J. M. Millam, M. Klene, C. Adamo, R. Cammi, J. W. Ochterski, R. L. Martin, K. Morokuma, O. Farkas, J. B. Foresman, D. J. Fox, **2016**, Gaussian 16, Revision A.03, Gaussian, Inc., Wallingford.

[4] (a) Becke, A. D. *J Chem Phys.* **1993**, *98*, 5648–5652. (b) Lee, C.; Yang, W. Parr, R.G. *Phys. Rev. B.* **1988**, *37*, 785–789.

[5] (a) T.H. Dunning. *J. Chem. Phys.* **1989**, 90, 1007–1023. (b) Woon, D.E.; Dunning, T.H. *J. Chem. Phys.* **1993**, *98*, 1358–1371.

[6] Peterson, K. A.; Figgen, D.; Dolg, M.; Stoll, H. *J. Chem. Phys.* **2007**, *126*, 124101.

[7] Zhao, Y., Truhlar, D. G.. *J. Chem. Phys.* **2006**, *125*, 194101.

[8] Marenich, A. V.; Cramer, C. J.; Truhlar, D. G. *J. Phys. Chem. B.* **2009**, *113*, 6378–6396.

[9] Grimme, S.; Antony, J.; Ehrlich, S.; Krieg, H. *J. Chem. Phys.* **2010**, *132*, 154104.

[10] P. Atkins, J. De Paula, *The Elements of Physical Chemistry*, Oxford University Press, Oxford, **2006**.

[11] Álvarez-Moreno, M.; de Graaf, C.; López, N.; Maseras, F.; Poblet, J.M.; Bo, C. *J. Chem. Inf. Model.* **2015**, *55*, 95-103.
